# Peptidylprolyl Isomerases as In Vivo Carriers for Drugs That Target Various Intracellular Entities

**DOI:** 10.3390/biom7040072

**Published:** 2017-09-29

**Authors:** Andrzej Galat

**Affiliations:** Service d’Ingénierie Moléculaire des Protéines (SIMOPRO), CEA, Université Paris-Saclay, F-91191 Gif/Yvette, France; galat@dsvidf.cea.fr; Fax: +33-169089137

**Keywords:** PPIase, FKBP, cyclophilin, rapamycin, FK506

## Abstract

Analyses of sequences and structures of the cyclosporine A (CsA)-binding proteins (cyclophilins) and the immunosuppressive macrolide FK506-binding proteins (FKBPs) have revealed that they exhibit peculiar spatial distributions of charges, their overall hydrophobicity indexes vary within a considerable level whereas their points isoelectric (pIs) are contained from 4 to 11. These two families of peptidylprolyl *cis*/*trans* isomerases (PPIases) have several distinct functional attributes such as: (1) high affinity binding to some pharmacologically-useful hydrophobic macrocyclic drugs; (2) diversified binding epitopes to proteins that may induce transient manifolds with altered flexibility and functional fitness; and (3) electrostatic interactions between positively charged segments of PPIases and negatively charged intracellular entities that support their spatial integration. These three attributes enhance binding of PPIase/pharmacophore complexes to diverse intracellular entities, some of which perturb signalization pathways causing immunosuppression and other system-altering phenomena in humans.

## 1. Introduction

**Figure 1:**
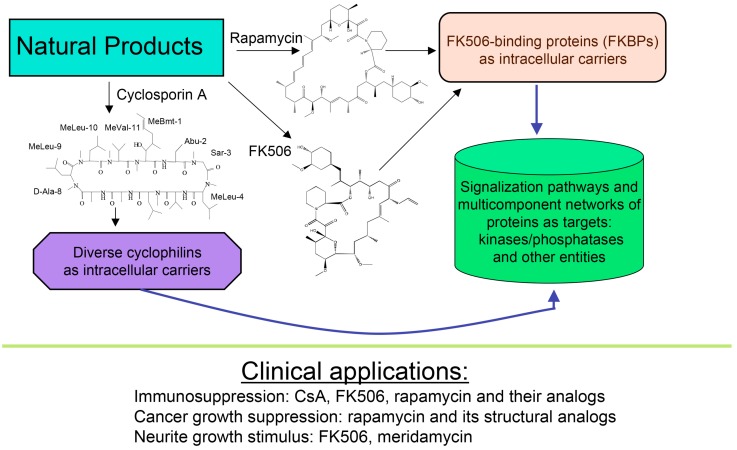
Scheme depicting two polyketides (FK506 and rapamycin) and cyclic peptide cyclosporine A (CsA) with a brief summary of their intracellular targets whose blocking by immunophilin/(immunosuppressive drug) complex causes diverse clinically useful effects.

About forty years ago, two different types of macrocyclic molecules were isolated and shown to possess immunosuppressive activities, such as the cyclic peptide containing non-standard amino acid residues (AAs) cyclosporine A (CsA) and its homologues [1], and two polyketides having l-pipecolic acid ring, namely immunosuppressive macrolide FK506 [2] and rapamycin [3,4]. Rapamycin and its different natural and synthetic derivatives such as everolimus, temsirolimus (CCI-779) or zotarolimus (ABT-578) have been used as anticancer drugs [5,6,7]. Some natural homologues of CsA, FK506 and rapamycin are devoid of immunosuppressive activity [7,8]. All those compounds have been found in soil samples coming from three different regions of Earth, namely CsA and its derivatives were purified from the ascomycete fungus *Tolypocladium inflatum* found in a soil sample from Norway [1], FK506 (tacrolimus) was isolated from a bacterial culture of *Streptomyces tsukubaensis* found in a soil sample from Japan [2] whereas rapamycin (sirolimus) was recuperated from a filament-forming bacterium, *Streptomyces hygroscopicus* found in a soil sample of Rapa Nui (Easter Island) [3,4], respectively. Several other natural polyketides have some structural similarity to FK506, namely meridamycin from *Streptomyces hydroscopicus* that was found in a soil sample from Venezuela [9], nocardiopsins isolated from a marine sediment sample found off the cost of Brisbane [10], or antascomicins purified from *Micromonospora* species found in a Chinese soil sample [11]. Even if they bind to 12 kDa FK506-binding protein (FKBP12), curiously these molecules are not immunosuppressive but instead they antagonize the actions caused by FK506 [11]. Moreover, non-immunosuppressive meridamycin [9] has neuroprotective activity [12,13]. The macrolide sanglifehrin-A and its natural homologues bind to cyclophilin A (CyPA) and perturb some immune responses [14], but its mode of action is different from that of the CsA-driven immunosuppression [15]. These different effects are depicted in Figure 1.

**Figure 2:**
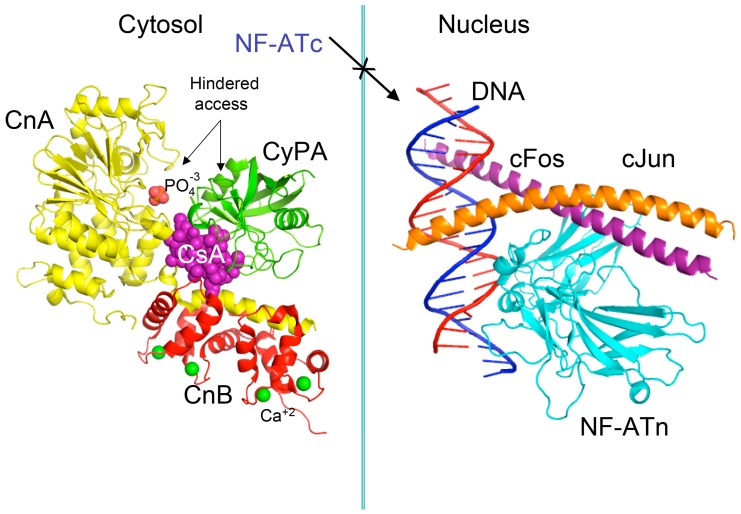
Scheme of the X-ray structure of a complex between human cyclophilin (hCyPA) (green ribbon) bound to cyclosporine A (CsA) (violet spheres) and the calcineurin heterodimer, calcineurin A (CnA), yellow ribbon), calcineurin B (CnB) (red ribbon) with four Ca^+2^ ions shown as green spheres. The cyclophilin A (CyPA)/CsA complex hinders access to phosphatase activity site (shown as bound phosphate ion in light red spheres) (1mf8.pdb [29]). Similar action causes the FK506/FKBP12a complex if it is bound to CnA–CnB (data not shown). Different phosphorylated components of transcription machinery rest immobilized in the cytoplasm, namely cytoplasmic form of transcription factor NF-ATc. On the left panel is shown the X-ray structure (1s9k.pdb) of a complex between human interleukin-2 (IL-2) ARRE1 promoter element (blue/red ribbon) and the fragments of the activator protein (AP-1) transcription complex, namely nuclear form of NF-ATn (cyan), cFos (violet) and cJun (orange) [30].

Pioneer works leading to isolation and characterization of several natural isoforms of peptidylprolyl *cis*/*trans* isomerase (PPIases) [16] such as CyPA [17], CyPB, CyPD, CyP40 (reviewed in [8]), and FKBP12a [18], FKBP13, FKBP25 and FKBP52 (reviewed in [7]) revealed that these immunophilins have sizeable expression levels in diverse organs (reviewed in [19]). The principal intracellular binders of CsA are: (1) CyPA (cytoplasm) [17]; (2) cyclophilin B (CyPB, endoplasmic reticulum (ER)) [20], and (3) cyclophilin D (CyPD, mitochondrial membrane) (reviewed in [8]) whereas abundantly expressed heat-shock protein-associated CyP40 binds weakly to CsA [21]. FKBP12a (cytoplasm) [18], FKBP25 (cytoplasm, diverse organelles and nucleus) [22], FKBP13 (ER-protein), or tetratricopeptide domain (TPR)-containing FKBP51 and FKBP52 (reviewed in [7,19]) bind to FK506 or rapamycin and their structural analogues [23]. It has been shown that either of the following two complexes, namely CyPA/CsA or FKBP12a/FK506 hinders the access to phosphatase activity site of calcineurin heterodimer [24]. In consequence, the phosphorylated pool of the nuclear factor of activated T cells transcription factor (NF-ATc) remains in the cytoplasm (see Figure 2) [25,26]. FKBP12/rapamycin binds to serine-threonine kinase mTOR and it hinders the access to its kinase activity site [27,28], which causes anergy of T cells. These hypotheses imply that under physiological conditions the intracellular contents of calcineurin heterodimer and mTOR in T cells are small, thus their enzymatic activities could be effectively blocked by the immunophilin/(immunosuppressive drug) complexes.

Recent analyses of a human interactome have shown that the expression level of CyPA is roughly equal to that of the core histones whereas FKBP52, FKBP25 and FKBP13 have expression levels smaller by about two orders of magnitude [31]. It would imply that under physiologic conditions, PPIase activity of some immunophilins could not be fully inhibited by the immunosuppressive drugs or their non-immunosuppressive analogues. For example, it has been suggested that the mitochondrial permeability transition pore is under the control of CyPD. Inhibition of the latter by CsA should shut down the pore that in turn could protect the organelle from oxidative stress. Due to high expression levels of the cytoplasmic CyPA and ER-retained CyPB, under physiological conditions these two PPIases should capture the entire content of CsA, which would leave little chance for a residual quantity of it to be bound to CyPD. This may in part explain why CsA did not improve clinical outcome of reperfusion therapy in patients with myocardial infarction [32].

In this review, we have described analyses of physical-chemical attributes, structures and some functional aspects of the cyclophilins and FKBPs. Some of those immunophilins are carriers for hydrophobic small molecular mass pharmacophores that affect several different intracellular signaling pathways [25,26,27,28]. We also have made analyses of physical-chemical and structural attributes of human kinases, some of which interact with different immunophilins. Analyses of recent literature indicate for more and more complex networks of proteins and other intracellular entities that are under the control of diverse PPIases [33,34,35,36,37,38,39,40,41]. Some intracellular supramacromolecular complexes containing charged moieties such as RNAs or DNAs interact with various PPIases [42,43,44,45,46,47,48,49,50,51]. Thus, some of the clinically useful immunosuppressive molecules (pharmacophores) may influence other signalization networks than the well-described scenario for CsA-, FK506-, or rapamycin-induced immunosuppression [24,25,26,27,28].

## 2. Strategy for Analyses of Sequences and Structures Used in This Review

### 2.1. Analyses of Sequences

**Figure 3:**
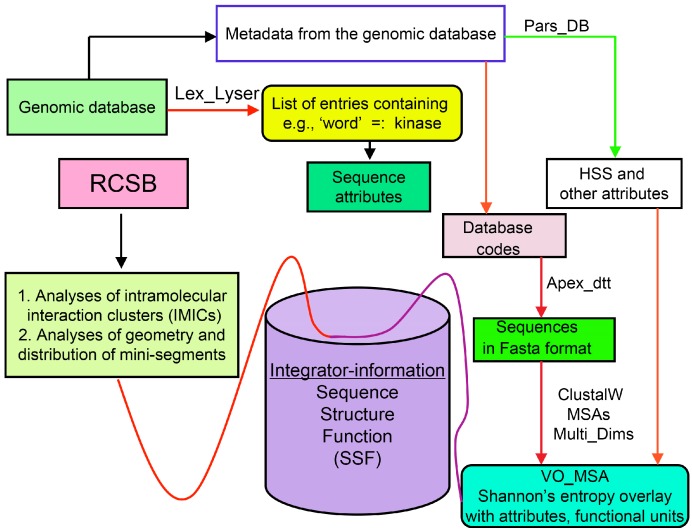
Scheme of different modules applied for analyses of genomic databases, sequences and structures of proteins. Abbreviations: HSS, hydrophobic sequence space; VO, vertically-oriented; MSAs, multiple sequence alignments; RCSB, Research Collaboratory for Structural Bioinformatics Protein Data Bank.

Analyses of genomic sequences and X-ray structures were schematically depicted in Figure 3. We used the BLAST program [52] for searching diverse genomic databases with sequences of FKBP12a, CyPA and the kinase domain of TOR as input templates. Human genomic database, which was downloaded from the PubMed server at the National Centre of Biotechnology Information (NCBI) (ftp.ncbi.nlm.nih.gov) [53], was converted into diverse sets of metadata using the Lex_Lyser program (written by A.G.), which also allowed to pull out functionally-related sets of proteins using specific keywords. For example, searching human genomic database with the keywords ‘kinase’ or ‘phosphatase’, the program extracted all sequence entries of both super-families of proteins. We also used the recently described package of programs for analyses of hydrophobic sequence space (HSS) and diverse attributes of sequences such as the theoretical points isoelectric (pIs), overall hydrophobicity indexes (HIs), distribution of charges along the polypeptide chain and their clusters [54]. HIs ≤ 25% are for hydrophilic proteins whereas HIs ≥ 40% are for hydrophobic proteins. Sequences were aligned with the ClustalW program [55]. Multiple sequence alignments (MSAs) of proteins were consecutively processed by the Multi_Dims program (written by AG), which created vertically oriented (VO) compressed forms of MSA (VO_MSA). For example, Section 3 shows the VO_MSA that was made from 22 different MSAs containing 576 sequences of FKBPs from various species. It illustrates a matrix of human sequences of the FK506-like binding domains (FKBDs) formatted as a VO_MSA, which contains Shannon’s entropy values in its third dimension [54]. The VO_MSA was enriched with information on secondary structure and van der Waals distances calculated from the X-ray data of several ternary complexes containing immunophilins bound to different immunosuppressive drugs. Macintosh versions of Lex_Lyser and Multi_Dims and their source codes written in Fortran 77 are available upon request. Also instructions on how to compile the above-mentioned programs using GNU C++ and Fortran compilers are available from the author.

### 2.2. Analyses of Structural Data

Intramolecular interaction clusters (IMICs) [56] that are shown on two-dimensional (2D) maps were computed from the X-ray structures of proteins whose coordinates were downloaded from the Research Collaboratory for Structural Bioinformatics (RCSB; Worldwide Protein Data Bank (wwPDB); http://www.rcsb.org) [57]. The IMICs that are close to the diagonal of the distance matrix are due to α-helical structures (1–5 interactions in α-helix), antiparallel β-sheets are perpendicularly oriented whereas parallel β-sheets are co-linearly oriented to the diagonal. The distance from the diagonal to given IMIC on the 2D map is proportional to the distance between the polypeptide segments in linear sequence. Graphical forms of the X-ray data were made with PyMOL [58]. Intermolecular distances were calculated from several X-ray structures of complexes of some immunophilins bound to their ligands [56].

## 3. Physical-Chemical Attributes of Human Cyclophilins and FKBPs

### 3.1. Hydrophobicity versus Charge Distribution

**Figure 4:**
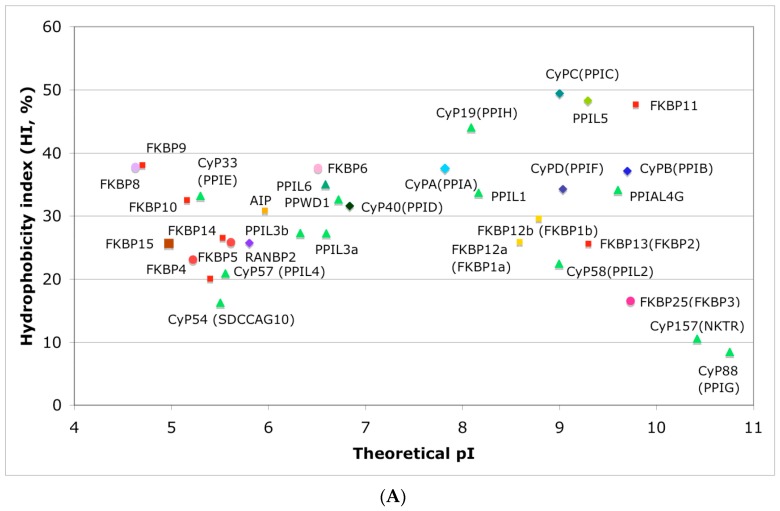

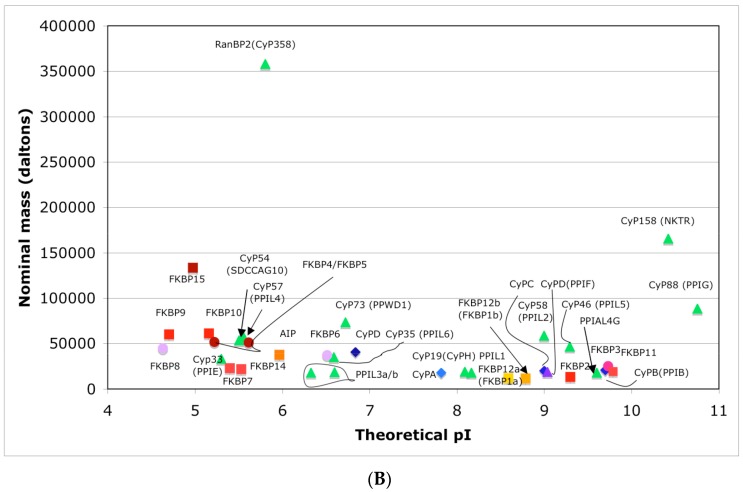
(**A**) Distribution of the overall hydrophobicity indexes (HIs) versus the theoretical points isoelectric (pIs) of human cyclophilins [8] (triangles and diamonds) and FKBPs [7] (circles and squares). The FKBPs and CyPs are shown with most often used abbreviation that is followed by the gene encoding each of them. Archetypal CyPA (light blue diamond); spliceosomal cyclophilins (light green triangles); tetratricopeptide repeat (TPR)-containing CyP40 (deep-blue diamond); large Ran-binding protein CyP358 (RANBP2, violet diamond); two isoforms of archetypal FKBP12 (yellow squares, genes *FKBP1a* and *FKBP1b*); endoplasmic reticulum (ER)-retained FKBPs (red squares); aryl-receptor associated (AIP) (FKBP37, deep yellow square); TPR-containing FKBPs (violet and light red circles); RNA- and DNA-binding FKBP25 (*FKBP3*; red circle); other abbreviations used for the FKBPs (gene in parentheses) are as follow: FKBP13 (*FKBP2*), FKBP52 (*FKBP4*), FKBP51 (*FKBP5*), FKBP36 (*FKBP6*), FKBP22 (*FKBP7*), FKBP38 (*FKBP8*), FKBP63 (*FKBP9*), FKBP65 (*FKBP10*), FKBP19 (*FKBP11*), FKBP22 (*FKBP14*), FKBP135 (*FKBP15*). (**B**) Distribution of the nominal masses versus the theoretical pIs of human cyclophilins and FKBPs (the same motifs were used as in panel A; the names of the genes (in parentheses) coding for peptidyl-prolyl cis/transe isomerases (PPIases) were used on both panels.

In Figure 4A is shown a distribution of the overall HIs of twenty human cyclophilins and fifteen FKBPs versus the theoretical pIs whereas in Figure 4B is shown a distribution of the nominal masses versus the pIs. Human CyPA (hCyPA) has a nominal mass of about 18 kDa and its multiple small size paralogues are expressed in diverse cells. For example, the archetypal hCyPA (blue diamond) has at least twelve small size paralogues, which are the components of spliceosomal complexes (light green triangles) [8,48]. Large cyclophilins (30 to 360 kDa) are fusion proteins that contain one PPIase domain and various combinations of other domains such as RNA-recognition domain (RRM), TPR, serine/arginine (SR)-rich domain, WD40 domain, leucine-rich (LR) domain, etc. (reviewed in [8]). Three cyclophilins are very hydrophobic proteins (blue circles), which reside in the ER (CyPB) [20], membranes (CyPC), and mitochondrial membranes (CyPD) [8].

**Figure 5:**
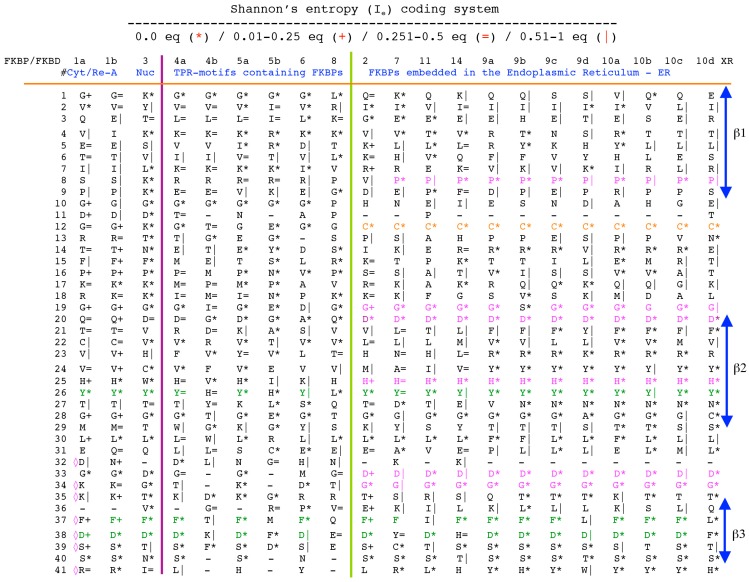

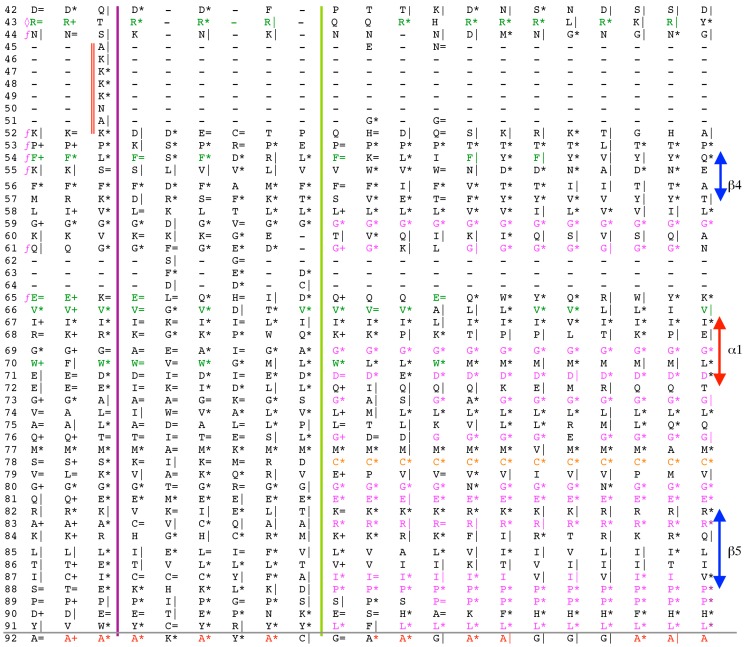

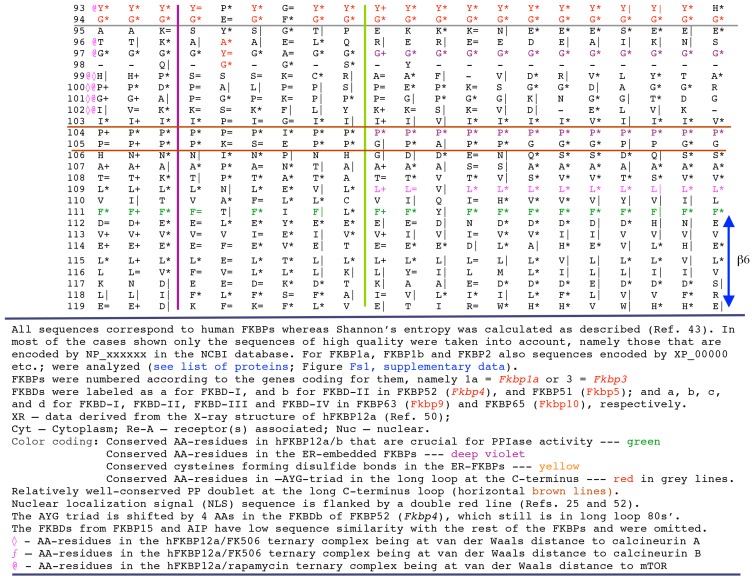
Vertical sequence alignment of the FKBDs from human FKBPs with Shannon’s entropy. Twenty-one FKBDs from human FKBPs formatted as a VO_MSA. The FKBDs from hFKBP15 and AIP were excluded from this MSA since they have a poor overlap (outliers) with the ensemble of shown sequences.

The archetypal FK506-binding protein (hFKBP12a) has at least one small paralogue (hFKBP12b; yellow squares) [7]. Large FKBPs are fusion proteins containing from one to four FKBDs and several different sequence motifs such as TPRs or calmodulin-binding domain (CBD). Six of the FKBPs (red squares) reside in the ER where each of the FKBDs has one disulfide bonding (Figure 5 and Figure S1 in Supplementary Materials). The positively charged hFKBP25 that is encoded by the *Fkbp3* gene (red circle) has the theoretical pI similar to those of the two large SR-rich spliceosome-associated cyclophilins [8,48]. The protein has several phosphorylation sites, which in fact diminish its experimentally established pIs [36]. Similar remark applies to the other nuclear PPIases, which may be phosphorylated and displaying several different isoforms with lower pIs than the theoretical one. The FKBDs of the FKBPs have relatively well-conserved features. For example, the average sequence similarity score ID_ave_ = 87% for 148 sequences of the FKBDs in a series of the FKBP13s, which are encoded by the *Fkbp2* gene. Likewise, similar levels of conservation retain the sequences of the FKBP12a and FKBP12b, namely the ID_ave_ = 90 and 85%, respectively (Figure 5).

### 3.2. Spatial Hydrophobicity *versus* Polarity in the X-ray Structures of CyPA and FKBP12a

**Figure 6:**
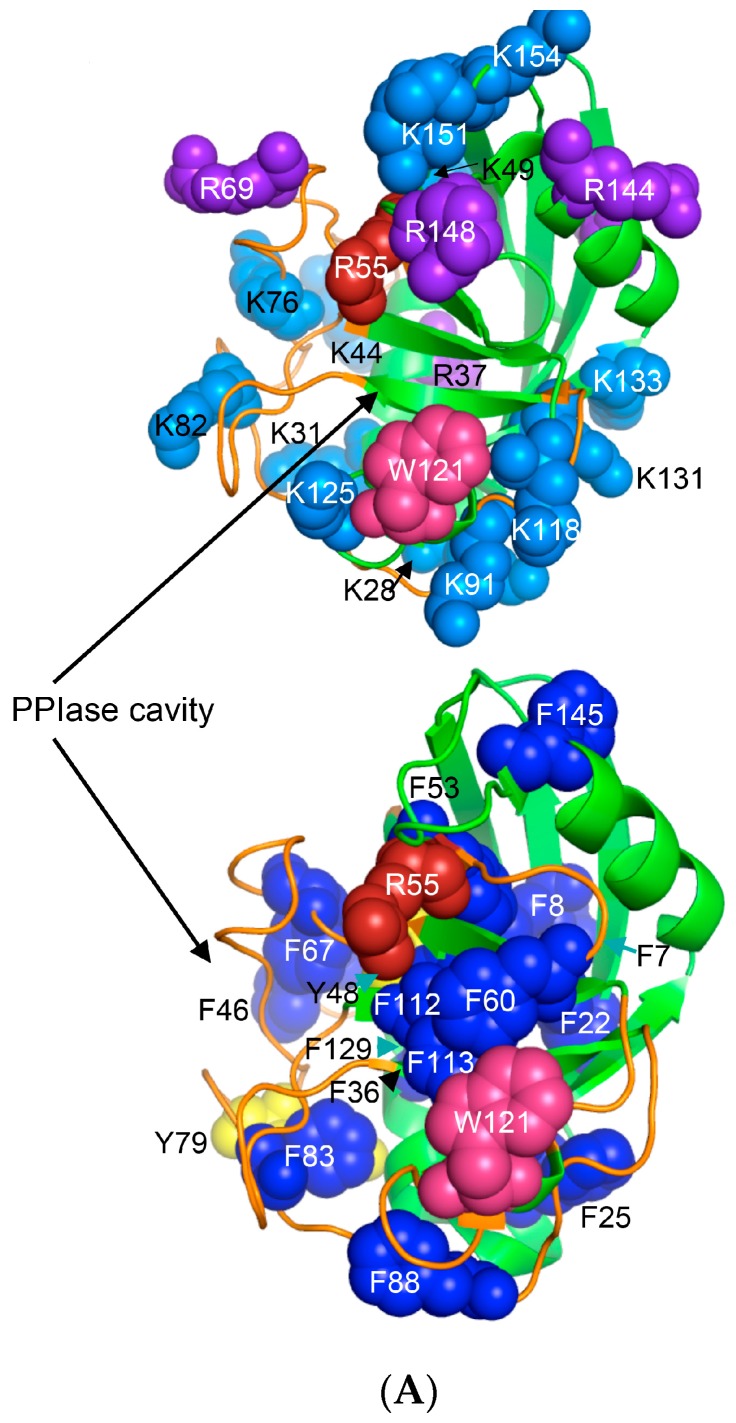

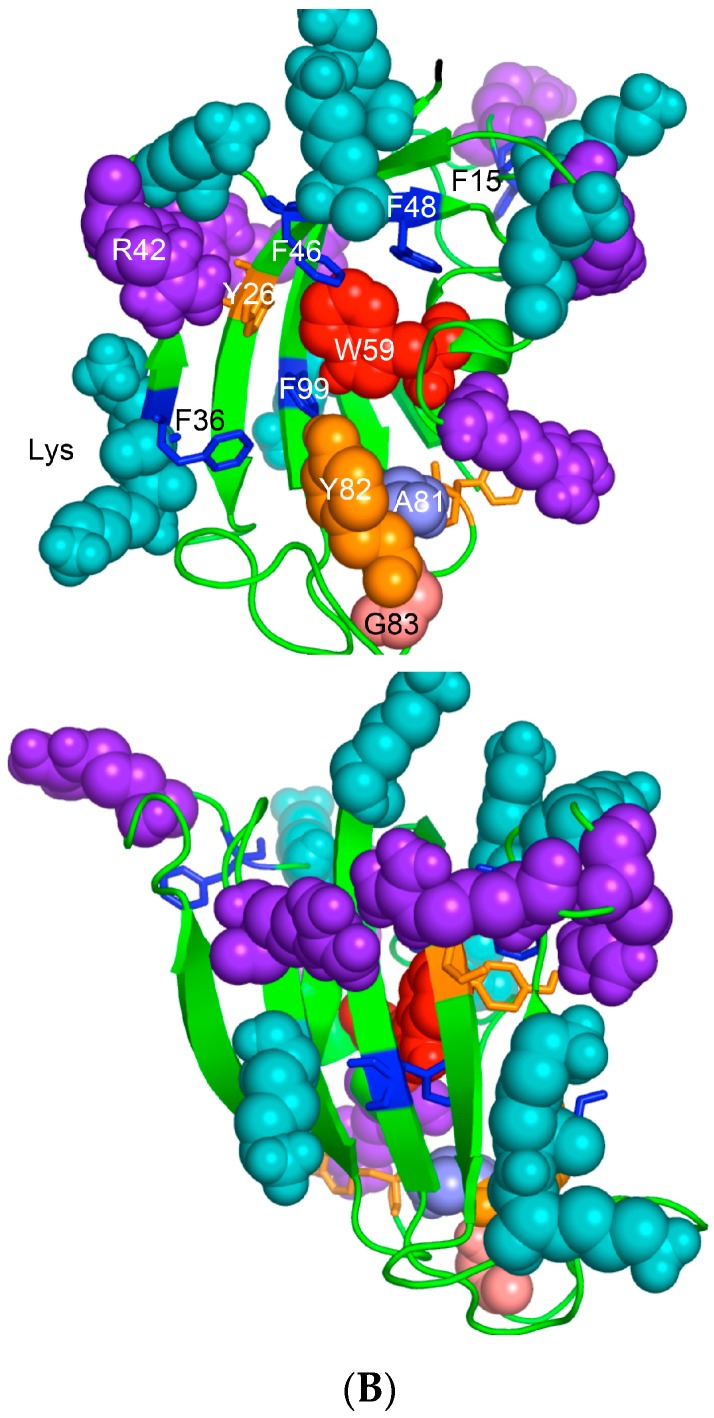
(**A**) X-ray structure of hCyPA (2CPL.pdb) [59] with explicitly shown K (deep teal) and R residues (violet). Upper and lower panel show PPIase cavity with R55 (deep red) that participates in *cis*/*trans* isomerisation of X-Pro epitopes and W121 (raspberry) being at the lower part of the cavity. The polypeptide backbone that makes up PPIase cavity is in orange whereas the structure rich in α-helices and β-strands is in green. At the lower panel is shown an extensive interaction network between fifteen F residues (blue spheres) and two Y residues (yellow spheres), which form a tight hydrophobic core of the cyclophilin fold [8,60]. F7, F129 and Y48 (cyan arrows) are not visible since they are on the opposite side of the structure; (**B**) Distribution of positively charged amino acid residues on the surface of hFKBP12a (the X-ray structure of hFKBP12a/rapamycin complex, 1FKB.pdb).

Human CyPA (NP_066953) has fifteen F residues (9%), which if added to the content of the other hydrophobic AAs suggest that it is a hydrophobic protein (HI = 37.6%). hCyPA and its complex with the very hydrophobic macrocycle CsA are soluble in aqueous solution [59]. hCyPA contains fourteen K (8.5%) and six R residues (3.5%) versus seven D (4.3%) and twelve E residues (7.3%), which implies that it is a basic protein (pI 7.8). In Figure 6A are shown two drawings of the X-ray structure of hCyPA [59]. hCyPA and the other cyclophilin-like domains (CLDs) have a tightly packed part made up with β-strands and α-helices (shown at the back of the drawing) and a loosely packed part made up with long loops, which accommodate PPIase cavity. CyPA is a PPIase, which possesses a large hydrophobic cavity that has a high affinity to CsA and its natural and synthetic analogues (Figure S2 Supplementary Materials). The structural features are highly conserved in the cyclophilin family of proteins [8,17,59,60].

On the upper panel are shown positively charged AAs such as R (violet) and K (deep teal), which are distributed on its surface at quasi-equidistant spatial positions. hFKBP12a is less hydrophobic than hCyPA but it also has highly charged surface [61] (Figure 6B). Complexes of CsA and its nonimmunosuppressive derivatives with hCyPA or other cyclophilins, as well as those of FK506 or rapamycin bound to hFKBP12a or the other FKBPs have about 50% of the hydrophobic surface of the drug that remains solvent exposed (effector’s domain). The binding of a pharmacophore to the shallow PPIase activity site of CyPA rigidifies the flexible loops forming the binding cavity while the effector’s domain of the pharmacophore acquires a high congruency to the docking surface on several different intracellular entities whose functional profiles become abrogated, which in consequence causes immunosuppression [26,27] and other system-altering phenomena (reviewed in [7,8,18,26]).

Several splicesome-associated cyclophilins are basic proteins [48,60] that have a good capacity to bind to RNAs whereas CyPB can bind to a double-stranded DNA [20]. Moreover, it has been shown that CyPA facilitated the transport of viral RNA [43] and it participated in the translocation of some proteins from the cytoplasm to the nucleus [44,45]. In Figure S2 are shown hydrophilic and hydrophobic patches on CyPA. It illustrates the positively charged patches that could interact with negatively charged biopolymers such as segments of DNA, diverse RNAs (mRNAs, tRNAs, microRNA etc.). Those patches are far from the CsA-binding cleft. Moreover, FKBP25 (pI 9.75) binds to single and double-stranded DNAs [49,50] structurally diversified RNAs [49,51], RNA granules [42] and polyribosomes [47] or phospholipid-membrane anchored receptors [41]. Thus, it could be envisioned that novel either natural or synthetic hydrophobic pharmacophores bound to CyPs or FKBPs could become effective blockers of various pivotal intracellular signalization pathways. Such complexes could become useful as tumor growth suppression agents and of some other systemic diseases.

## 4. Some Reflections on the Mechanism of Action Induced by CsA, Tacrolimus and Sirolimus

Both, the cyclophilins and the FKBPs have a shallow binding pocket (PPIase activity site), which can accommodate medium size ligands (MSL), such as the polyketides whose nominal mass remains within 800 to 1000 Da. Some of the cyclophilin/MSL or FKBP/MSL complexes bind to diverse intracellular targets in which interactions between the exposed hydrophobic epitopes of the ligand are strengthened by auxiliary docking of the side chains of interacting proteins. For example, analyses of the X-ray structures of calcineurin A/calcineurin B (1mf8.pdb) bound to CyPA/CsA (Figure 2) [29,62] or FKBP12a/FK506 [63], and the X-ray structure of the rapamycin-binding domain (RBD) of mTOR bound to FKBP12a/rapamycin [28] revealed the following intermolecular interaction networks: (1) the (CnA–CnB)/(hFKBP12a–FK506; 1TCO.pdb) [63] ternary complex is stabilized by 105 interactions (distances ≤4.5 Å) between CnA/CnB and FK506, 175 interactions between hFKBP12a and FK506, and 210 interactions between CnA/CnB and hFKBP12a; (2) the hFKBP12a/rapamycin complex bound to the RBD of mTOR (4FAP.pdb) [28] is stabilized by 177 inter-molecular interactions (distances ≤4.5 Å) between rapamycin and hFKBP12a in addition to 106 and 77 interactions between rapamycin–RBD and hFKBP12a–RBD, respectively. This suggests that the docking surface of the hFKBP12a/rapamycin complex to the RBD has as many geometrical constraints as that for docking of rapamycin inside PPIase cavity of hFKBP12a. Analyses show that two different segments of the hFKBP12a interact with CnA and CnB whereas only the C-terminus of hFKBP12a interacts with mTOR (Figure 5). It is worth mentioning that the sequence consisting the RBD and kinase domain of TOR remained highly conserved in the organisms ranging from *Saccharomyces cerevisiae* to disparate mammals (for 9 sequences ID_ave_ = 76%; Figure S3 Supplementary Materials). Moreover, data shown in Supplementary Figure S3a indicate for a remarkable conservation of the global sequence attributes of TORs in organisms ranging from *S. cerevisiae* to *Homo sapiens*. Likewise, physical-chemical attributes of the AAs in the RBDs that interact with the hFKBP12a/rapamycin complex are well conserved (Figure S4). mTOR is a part of at least two different assemblies of proteins, known as mTORC1 and mTORC2 [64,65]. Both entities are accompanied by a relatively small and hydrophobic protein known as target of rapamycin-complex subunit lethal with SEC13 protein 8 (LST8) (NP_07176) whose X-ray structure (4JVS.pdb) [66] is shown in Section 5. Several other factors regulate activity of mTORC1 and mTORC2, some of which are large hydrophobic proteins (Table S1). 

## 5. Analyses of Sequence Attributes of Human Kinases and Phosphatases

**Figure 7:**
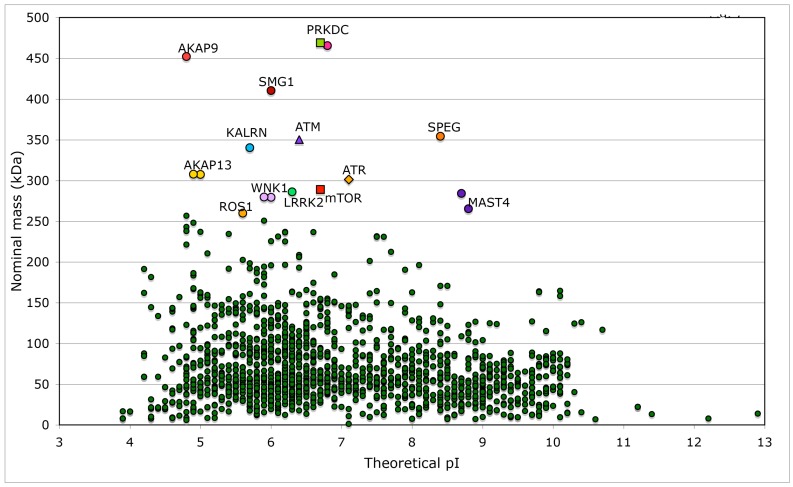
Distribution of nominal masses versus theoretical pI of 1908 proteins, which have the keyword ‘kinase’ encoded in the analyzed human genomic database. Sequences of five large size serine/threonine-protein kinases have some common features with that of mTOR (NP_004949), namely SMG1 (NP_055907) and its multiple isoforms that are involved in nonsense-mediated mRNA decay; ATR (NP_001175) that is essential for DNA damage repair and phosphorylation of several kinases essential in cell cycle; ATM (NP_000042), which is a close homologue of ATR that controls genome stability and phosphorylation of diverse proteins in response to DNA damage signaling; PRKDC, two isoforms of DNA-dependent protein kinase catalytic subunit (NP_008835 and NP_001075109) that are implicated in double-strand DNA repair and in telomere stability through interaction with telomere length regulated protein (TEL2, NP_057195.2; GI:225545550) and two isoforms of transformation/transcription domain-associated protein (TRRAP; NP_003487 and NP_001231509). Few other kinases (KALRN, SPEG, WNK1, LRRK2, MAST4, and ROS1) and several proteins interacting with kinases such as A-kinase anchor protein AKAP9 (two isoforms, red circles), AKAP13 (yellow circles), and AKAP6 (violet circle), as well as unconventional myosin-IXa (MYO9A; blue square), several isoforms of tyrosine-protein phosphatase non-receptor type 13 (PTPN13; blue circles), and extracellular matrix protein FRAS1 (brown triangle) have nominal masses greater than 250 kDa (Table S2). FRAS1 was found because it has several Furin-like repeats; Furin is a serine-kinase dependent subtilisin-like proprotein convertase, which may cleave and activate different growth hormones and related proteins.

We made several analyses of human genomic database and extracted physical-chemical attributes of kinases and phosphatases, some of which interact with different immunophilins. Searches of the database with the keywords ‘kinase’ or ‘phosphatase’ supplied long lists of proteins belonging to these two super-families and their cofactors. For example, the outputs generated by Lex_Lyser list 1908 entries for ‘kinase’ (Table S2) and 833 entries for ‘phosphatase’ (data not shown), which constitute about 7.7% of the analyzed human genomic database. In Figure 7 is shown a distribution of the nominal masses of human kinases versus the theoretical pIs. Several kinases are large multi-domain proteins (≥250 kDa), which include the transformation/transcription domain associated protein (TRRAP) [67], the ataxia telangiectasia and Rad3-related protein (ATR) [68], the ataxia telangiectasia related protein (ATM) [69] and mTOR [29]. Kinase domain of nonsense mediated mRNA decay associated PI3K related kinase (SMG1) [70] has about 33% sequence similarity to its counterpart in mTOR. Sequences of TRRAP, ATM, ATR and mTOR have similar modular organization (Figure S4 Supplementary Materials) but their overall sequence similarity is low (the IDs from 12 to 14%). Kinase domains of these proteins have somewhat better sequence similarity (the IDs from 22 to 24%).

**Figure 8:**
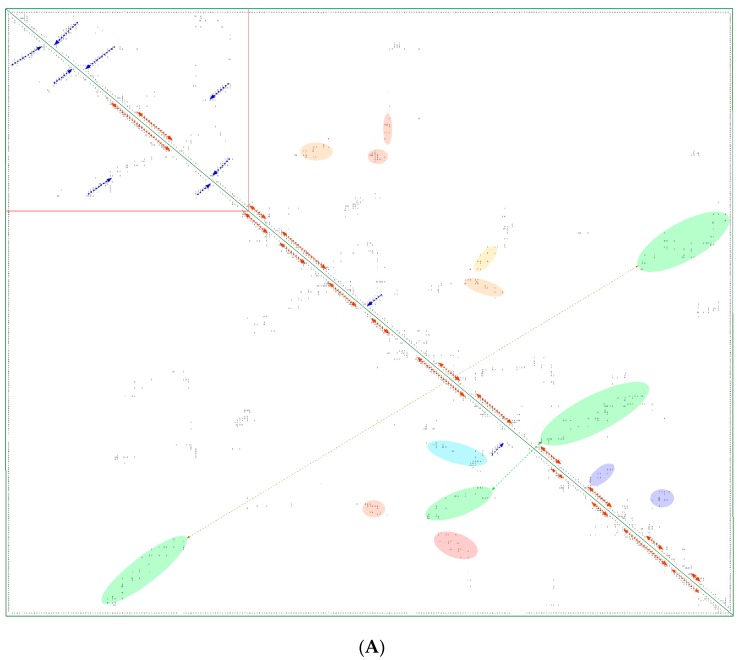

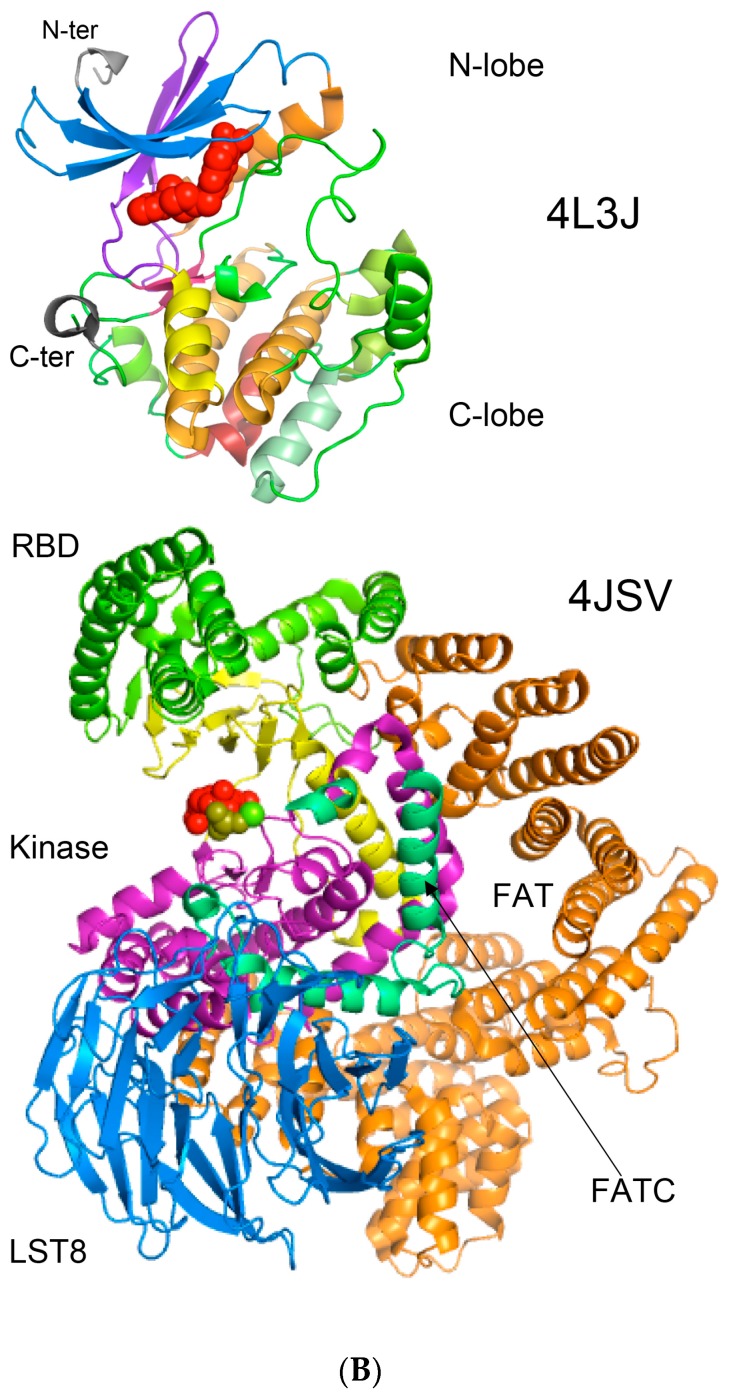
(**A**) Two-dimensional map of the intramolecular interaction clusters (IMICs) calculated from the X-ray structures of kinase domains in ribosomal protein S6 kinase β1 isoform (S6K1; 4L3J.pdb [71]; NP_003152; upper triangle) and mTOR (4JSV.pdb) [66]; AAs from 1376 to 2549; lower triangle) containing rapamycin-binding domain (RBD), kinase and phosphatidylinositol kinase (PIK)-related kinase (FATC) domains, which is bound to mammalian lethal with SEC13 protein 8 (LST8) bound to adenosine-5'-diphosphate (ADP; red-green spheres). (**B**) X-ray structure of S6K1 bound to 2-{[4-(5-ethylpyrimidin-4-yl)piperazin-1-yl]methyl}-5-(trifluoromethyl)-1h-benzimidazole (red spheres; upper panel); N-lobe kinase of the kinase domain has β-sheets (blue/violet) linked via an α-helix (orange) whereas its C-terminal counterpart is rich in α-helical segments (different colors); X-ray structure of mTOR (lower panel) bound to rich in β-strands LST8 (blue ribbon); the RBD is in green ribbon; N-lobe is in light yellow whereas its C-lobe (raspberry ribbon) is terminated with α-helical FATC domain (deep green); rich in α-helical segments FAT domain is at the N-terminus of the RBD (orange ribbon).

In Figure 8A is shown a 2D map of the IMICs calculated from the X-ray structures of the kinase domains from ribosomal protein S6 kinase beta-1 (S6K1) [71] (upper triangle) and mTOR [66] (lower triangle). In Figure 8B are shown the X-ray structures of S6K1 (upper panel) and a large fragment of mTOR containing rapamycin-binding domain (RBD; lower panel). Both proteins belong to AGC family but their sequences are highly dissimilar (ID = 6%). S6K1 (aka STKc-p70S6k) is one of the downstream components interacting with mTOR. Despite low sequence similarity, several IMICs have similar distributions in both triangles of the 2D map, which indicates that the major spatial features are well conserved in both domains. For example, the IMICs in the N-terminal lobes, which are shown at the left upper corner of the figure that was designated in the red box, correspond to two sets of anti-parallel β-sheets (blue arrows) that are followed by a long α-helix (orange arrow) and two sets of anti-parallel β-sheets. Even if the alignment of the α-helices in the C-lobes is imperfect, several groups of the IMICs, which were colored as green/blue ellipsoids linked via dotted arrows, have similar positions in both triangles. Several groups of the IMICs that are indicated as red and violet ellipsoids however are unique spatial features of each domain. A large-size format of Figure 8A is given in Supplementary Materials.

## 6. Conclusions

About thirty years ago it was shown that the principal cytoplasmic binder of CsA is cyclophilin A [17]. Shortly after that several other PPIases were isolated whose activity could be fully inhibited by FK506 or rapamycin [18,19]. However, due to relatively high content of PPIases in T cells and other cell lines, it has been excluded from the beginning that a direct inhibition of PPIases activity may have any relation with immunosuppressive actions of the drugs. This assumption has led to several seminal discoveries [24,25,26,27,28], namely blocking cellular activities of ternary or higher order complexes being at a low expression level caused by PPIase/immunosuppressive drug was responsible for beneficial effects in clinical applications of CsA, FK506, rapamycin and their structural analogues.

However, more than 50% of the PPIases are large proteins that possess various domains and sequence motifs (reviewed in [7,8,19,60]). Some of the PPIases bind to transcription factors [33,34,35,46], cause PPIase-driven protein folding [72], can modify certain structural features of diversified forms of RNAs [42,43,47,48,49,51], and are often associated to factors crucial for maintaining homeostasis pathways [73]. PPIases may also bind to some intrinsically disordered segments and epitopes of proteins [74] and supramacromolecular entities [40,64,65], which alter physical-chemical, spatial and functional attributes of such complexes. Binding between given PPIase and diverse intracellular moieties should give rise to novel type of a manifold, whose overall properties such as physical-chemical attributes, local structures and functional features could be altered. Diverse PPIase/protein, PPIase/RNA, PPIase/DNA complexes may form transient manifolds whose synergy induces a fine functional adequateness, which is a pivotal part of high-fidelity PPIase-driven processes in biological systems [7,8,21]. It is worth mentioning that disturbance of multicomponent entities in vivo may inadvertently alter diverse vital processes [75]. Formation and functional aspects of the above-mentioned assemblies of macromolecules was probably acquired via a process driven by self-organized criticality (SOC) phenomenon. For example, transformation of sequence information constraints into a functional structured protein [76,77] via build up of clusters of hydrophobic amino acids was elaborated using some aspects of the SOC theorem [77,78]. This theorem could have been one of crucial factors that had modulated evolution and adaptation of species. For example, the question whether some evolutionary events created a remarkable congruency between the docking surfaces of diverse immunophilin/rapamycin complexes and the RBD of mTOR or were it rather due to a serendipitous event remains without answer. It could be postulated however that an ensemble of geometrical determinants of the effector’s domain of rapamycin plus some side-chains of the immunophilin resemble the docking surface of substrates, or inhibitors, or regulatory factors of mTORC1 and mTORC2. However, it remains puzzling whether only a microorganism on Easter Island generated rapamycin or other microorganisms in different parts of the globe produce similar types of metabolites? It would be also interesting to investigate if interruption of ternary or higher order complexes is typical for these several microbial metabolites described in this review or such ‘defense mechanism’ is a common feature of other products produced by disparate microorganisms.

It has been shown that in some cell lines, rapamycin actions were not correlated with the inhibition of mTORC1 and mTORC2 [79,80]. A prolonged clinical use of rapamycin leads to dysfunction of glucose homeostasis [81]. Moreover, different combinations of immunophilin/rapamycin complexes [82] including hFKBP25/rapamycin [83] bind to the RBD and hinder the access to kinase activity site of mTOR. These data suggest that mTOR has a sizeable allostery, which tunes up the interactions between the RBD and diverse immunophilin/pharmacophore complexes. Whether rapamycin and its analogues, which utilize immunophilins as intracellular carriers, could modulate functional features of other entities than mTORC1 or mTORC2 requires some explorations. 

Diversified physical-chemical attributes, domain structure, retention within specific intracellular compartments and abundant expression levels of some PPIases in various cells suggest that they would be good carriers for different natural or synthetic hydrophobic polyketides that could target disparate intracellular processes and signalization networks [7,8,21,27,28,29,30,63,64,82], some of which might control human longevity [84,85,86,87,88]. Novel and sophisticated strategies however would have to be invented [89,90,91,92] that could allow unraveling functional inputs of each of the immunophilins and their complexes with different pharmacophores [7,8] to multidimensional networks made up of myriads of feedback loops, which in part rely upon disparate components listed in Table S2. Moreover, vigorous searches must be made on isolation of novel strains producing polyketides, hydrophobic cyclic peptides or other small molecular mass compounds that bind to different PPIases, thereby such complexes could interfere with some crucial signalization intracellular pathways but also in controlling networks of cells.

## Supplementary Materials

The following are available online at www.mdpi.com/2218-273X/7/4/72/s1, Figure S1: List of aligned FKBDs with the sequence of human FKBP13 (*Fkbp2*) as an arbitrary chosen top reference and the MSA containing the sequence of hFKBP13 and its orthologs expressed in disparate species, Figure S2: Three images of the X-ray structure of human CyPA (2CPL.pdb), Figure S3: MSA of nine sequences of TOR from several organisms, Figure S4: Alignment of full sequences of human ATR-ATM-mTOR proteins, Table S1: Sequence attributes of several proteins that contribute to the formation of TORC1 and TORC2 complexes, Table S2: Human kinases and their co-factors that were found by Lex_Lyser, a large-size format of Figure 8A.
