# Supplementary material for: Peptidylprolyl Isomerases as In Vivo Carriers for Drugs That Target Various Intracellular Entities
Source: Biomolecules. 2017 Sep 29;7(4):72. doi: 10.3390/biom7040072 (PMC5745455; doi:10.3390/biom7040072)
Supplement: Supplementary file 1 [file biomolecules-07-00072-s001.zip › Supplementary/biomolecules-224310-supplementary.pdf]

Supplementary materials

Figure S1. List of aligned FKBDs with the sequence of human FKBP13 (*Fkbp2*) as an arbitrary chosen top reference (pp. 2-6) and the MSA containing the sequence of hFKBP13 and its orthologs expressed in disparate species (pp. 7-12), which was used as one of the input files to the Multi-Dims program that has created the VO_MSA (Figure 5).

Figure S2. Three images of the X-ray structure of human CyPA (2CPL.pdb) shown as spheres with the following color usage: Lys (K) marine blue; Arg (R) violet; His (H) black; Asp (D) and Glu (E) chartreuse; Asn (N), Gln (Q), Gly (G), Thr (T), Ser (S), Pro (P) are in orange whereas hydrophobic AAs are in firebrick (outside CsA-binding cleft) and different shades of pink in the binding cleft. Amino acid residues forming cyclosporin A binding site were labeled. Image 1 – CsA-binding cleft; Image 2 – side-view of the binding cleft; Image 3 – the opposite side of the binding cleft with Val (V1) and Glu (E165) explicitly colored in light sand color (pp. 13-15).

Figure S3. MSA of nine sequences of TOR from several organisms, namely 1) *Homo sapiens*; 2) *Mus musculus*; 3) *Rattus norvegicus*; 4) *Ovis aries*; 5) *Capra hircus*; 6-7) two isoforms from *Drosophila melanogaster*; 8-9) two isoforms from *Saccharomyces cerevisiae* (TOR1 and TOR2). Sequence attributes of the aligned sequences are given in Figure S3A (pp. 16-19).

Figure S4. Alignment of full sequences of human ATR-ATM-mTOR proteins (pp. 20-24).

Table S1. Sequence attributes of several proteins that contribute to the formation of TORC1 and TORC2 complexes (p. 25).

Table S2. Human kinases and their co-factors that were found by Lex_Lyser (pp. 26-57).

Figure S1 - List of aligned sequences using human sequence of FKBP13 (*Fkbp2*) as arbitrary chosen reference.

1	NP_004461	142AAs	32- 137/Alg = 106AA	[Homo sapiens].
2	NP_001032558	140AAs	30- 135/Alg = 106AA	[Bos taurus].
3	NP_032046	140AAs	30- 135/Alg = 106AA	[Mus musculus].
4	NP_001253744	142AAs	32- 137/Alg = 106AA	[Macaca mulatta].
5	NP_001127900	140AAs	30- 135/Alg = 106AA	[Rattus norvegicus].
6	NP_001107353	141AAs	31- 136/Alg = 106AA	[Xenopus tropicalis].
7	NP_001004677	138AAs	28- 133/Alg = 106AA	[Danio rerio].
8	NP_001158699	137AAs	27- 132/Alg = 106AA	[Oncorhynchus mykiss].
9	NP_001188070	138AAs	28- 133/Alg = 106AA	[Ictalurus punctatus].
10	NP_001079493	141AAs	31- 136/Alg = 106AA	[Xenopus laevis].
11	NP_001299353	145AAs	35- 140/Alg = 106AA	[Papilio xuthus].
12	NP_010807	135AAs	24- 132/Alg = 109AA	[Saccharomyces cerevisiae S288c]
13	JAO69915	138AAs	28- 133/Alg = 106AA	[Poeciliopsis prolifica].
14	XP_016084356	138AAs	28- 133/Alg = 106AA	[Sinocyclocheilus graham
15	XP_016071878	140AAs	30- 135/Alg = 106AA	[Miniopterus natalensis]
16	XP_016041686	139AAs	29- 134/Alg = 106AA	[Erinaceus europaeus].
17	XP_015974039	140AAs	30- 135/Alg = 106AA	[Rousettus ae
18	XP_006980788	140AAs	30- 135/Alg = 106AA	[Peromyscus m
19	XP_015826586	138AAs	28- 133/Alg = 106AA	[Nothobranchius furzeri]
20	XP_015744729	141AAs	31- 136/Alg = 106AA	[Python bivittatus].
21	XP_015683908	141AAs	31- 136/Alg = 106AA	[Protobothrops mucrosqua
22	XP_007244563	138AAs	28- 133/Alg = 106AA	[Astyanax mexicanus].
23	XP_006911221	140AAs	30- 135/Alg = 106AA	[Pteropus ale
24	XP_006771685	140AAs	30- 135/Alg = 106AA	[Myotis davidii].
25	XP_015397493	140AAs	30- 135/Alg = 106AA	[Panthera tigris altaica
26	XP_015333657	140AAs	30- 135/Alg = 106AA	[Marmota marmota marmota
27	XP_005577499	142AAs	32- 137/Alg = 106AA	[Macaca fascicularis].
28	XP_015272310	141AAs	31- 136/Alg = 106AA	[Gekko japoni
29	XP_015248799	139AAs	29- 134/Alg = 106AA	[Cyprinodon variegatus].
30	XP_015105405	140AAs	30- 135/Alg = 106AA	[Vicugna paco

31	XP_014969226	142AAs	32- 137/Alg = 106AA	[Macaca mulat
32	XP_011957484	140AAs	30- 135/Alg = 106AA	[Ovis aries].
33	XP_014856097	138AAs	28- 133/Alg = 106AA	[Poecilia mex
34	XP_014935343	140AAs	30- 135/Alg = 106AA	[Acinonyx jubatus].
35	XP_014748502	138AAs	28- 133/Alg = 106AA	[Sturnus vulgaris].
36	XP_014700413	140AAs	30- 135/Alg = 106AA	[Equus asinus
37	XP_006149888	140AAs	30- 135/Alg = 106AA	[Tupaia chine
38	XP_005864247	140AAs	30- 135/Alg = 106AA	[Myotis brandtii].
39	XP_006176941	140AAs	30- 135/Alg = 106AA	[Camelus ferus].
40	XP_006129809	144AAs	31- 136/Alg = 106AA	[Pelodiscus s
41	XP_006036922	142AAs	32- 137/Alg = 106AA	[Alligator sinensis].
42	XP_005988896	141AAs	31- 136/Alg = 106AA	[Latimeria ch
43	XP_005894189	140AAs	30- 135/Alg = 106AA	[Bos mutus].
44	XP_005808854	138AAs	28- 133/Alg = 106AA	[Xiphophorus maculatus].
45	XP_006106614	140AAs	30- 135/Alg = 106AA	[Myotis lucifugus].
46	XP_008952214	142AAs	32- 137/Alg = 106AA	[Pan paniscus].
47	XP_013976757	140AAs	30- 135/Alg = 106AA	[Canis lupus
48	XP_003828606	142AAs	32- 137/Alg = 106AA	[Pan paniscus].
49	XP_013873214	138AAs	28- 133/Alg = 106AA	[Austrofundulus limnaeus
50	XP_005660807	140AAs	30- 135/Alg = 106AA	[Sus scrofa].
51	XP_005699908	140AAs	30- 135/Alg = 106AA	[Capra hircus
52	XP_005736999	138AAs	28- 133/Alg = 106AA	[Pundamilia nyererei].
53	XP_005384688	140AAs	30- 135/Alg = 106AA	[Chinchilla l
54	XP_005333562	140AAs	30- 135/Alg = 106AA	[Ictidomys tridecemlinea
55	XP_003452386	138AAs	28- 133/Alg = 106AA	[Oreochromis niloticus].
56	XP_005351906	140AAs	30- 135/Alg = 106AA	[Microtus och
57	XP_013006176	140AAs	30- 135/Alg = 106AA	[Cavia porcellus].
58	JAI39202	139AAs	28- 133/Alg = 106AA	[Bactrocera latifrons].
59	XP_012975627	140AAs	30- 135/Alg = 106AA	[Mesocricetus
60	XP_010898827	137AAs	27- 132/Alg = 106AA	[Esox lucius].
61	XP_004852587	138AAs	28- 133/Alg = 106AA	[Heterocephal

62 XP_004770232 140AAs | 30- 135/Alg = 106AA ||[Mustela puto

63 XP_012876646 140AAs | 30- 135/Alg = 106AA ||[Dipodomys ordii].

64 XP_012816015 141AAs | 31- 136/Alg = 106AA ||[Xenopus trop

65 XP_004656659 140AAs | 30- 135/Alg = 106AA ||[Jaculus jacu

66 XP_004618432 140AAs | 30- 135/Alg = 106AA ||[Sorex araneu

67 XP_012666155 140AAs | 30- 135/Alg = 106AA ||[Otolemur gar

68 XP_004596785 139AAs | 29- 134/Alg = 106AA ||[Ochotona princeps].

69 XP_012581059 140AAs | 30- 135/Alg = 106AA ||[Condylura cr

70 XP_012633386 140AAs | 30- 135/Alg = 106AA ||[Microcebus m

71 XP_012520835 140AAs | 30- 135/Alg = 106AA ||[Propithecus coquereli].

72 XP_004394068 140AAs | 30- 135/Alg = 106AA ||[Odobenus ros

73 XP_012408303 139AAs | 29- 134/Alg = 106AA ||[Sarcophilus harrisii].

74 XP_004264331 140AAs | 30- 135/Alg = 106AA ||[Orcinus orca

75 XP_012351963 142AAs | 32- 137/Alg = 106AA ||[Nomascus leucogenys].

76 XP_004643825 140AAs | 30- 135/Alg = 106AA ||[Octodon degus].

77 XP_004643824 140AAs | 30- 135/Alg = 106AA ||[Octodon degus].

78 XP_004479918 140AAs | 30- 135/Alg = 106AA ||[Dasypus novemcinctus].

79 XP_003274202 142AAs | 32- 137/Alg = 106AA ||[Nomascus leucogenys].

80 XP_004479917 140AAs | 30- 135/Alg = 106AA ||[Dasypus novemcinctus].

81 XP_012318520 142AAs | 32- 137/Alg = 106AA ||[Ao

82 XP_011718910 142AAs | 32- 137/Alg = 106AA ||[Macaca nemestrina].

83 XP_011897294 142AAs | 32- 137/Alg = 106AA ||[Cercocebus atys].

84 XP_011848866 142AAs | 32- 137/Alg = 106AA ||[Mandrillus l

85 XP_011797983 142AAs | 32- 137/Alg = 106AA ||[Colobus angolensis pall

86 XP_003974414 138AAs | 28- 133/Alg = 106AA ||[Takifugu rubripes].

87 XP_004068088 138AAs | 28- 133/Alg = 106AA ||[Oryzias latipes].

88 XP_011367027 140AAs | 30- 135/Alg = 106AA ||[Pteropus vam

89 XP_006937524 184AAs | 74- 179/Alg = 106AA ||[Felis catus]

90 XP_011219931 140AAs | 30- 135/Alg = 106AA ||[Ailuropoda m

91 XP_010993050 140AAs | 30- 135/Alg = 106AA ||[Camelus drom

92 XP_010836289 140AAs | 30- 135/Alg = 106AA ||[Bison bison

93 XP_010770745 138AAs | 28- 133/Alg = 106AA ||[Notothenia coriiceps].

```
--------------------------------------------------------------------------------
 94 XP_010752642   138AAs | 28- 133/Alg = 106AA ||[Larimichthys crocea].
--------------------------------------------------------------------------------
 95 XP_010597383   140AAs | 30- 135/Alg = 106AA ||[Loxodonta af
--------------------------------------------------------------------------------
 96 XP_010634398   140AAs | 30- 135/Alg = 106AA ||[Fukomys dama
--------------------------------------------------------------------------------
 97 XP_010346544   142AAs | 32- 137/Alg = 106AA ||[Saimiri boliviensis bol
--------------------------------------------------------------------------------
 98 XP_010375362   142AAs | 32- 137/Alg = 106AA ||[Rhinopithecus roxellana
--------------------------------------------------------------------------------
 99 XP_010375348   142AAs | 32- 137/Alg = 106AA ||[Rhinopithecus roxellana
--------------------------------------------------------------------------------
100 XP_009421628   142AAs | 32- 137/Alg = 106AA ||[Pan troglodytes].
--------------------------------------------------------------------------------
101 XP_009244471   142AAs | 32- 137/Alg = 106AA ||[Pongo abelii].
--------------------------------------------------------------------------------
102 XP_003909669   142AAs | 32- 137/Alg = 106AA ||[Papio anubis].
--------------------------------------------------------------------------------
103 XP_008837174   140AAs | 30- 135/Alg = 106AA ||[Nannospalax
--------------------------------------------------------------------------------
104 XP_008707188   140AAs | 30- 135/Alg = 106AA ||[Ursus mariti
--------------------------------------------------------------------------------
105 XP_008565641   140AAs | 30- 135/Alg = 106AA ||[Galeopterus variegatus]
--------------------------------------------------------------------------------
106 XP_008304826   138AAs | 28- 133/Alg = 106AA ||[Stegastes partitus].
--------------------------------------------------------------------------------
107 XP_008255497   182AAs | 29- 134/Alg = 106AA ||[Or
--------------------------------------------------------------------------------
108 XP_005305586   141AAs | 31- 136/Alg = 106AA ||[Chrysemys picta bellii]
--------------------------------------------------------------------------------
109 XP_008047615   140AAs | 30- 135/Alg = 106AA ||[Tarsius syrichta].
--------------------------------------------------------------------------------
110 XP_007992347   142AAs | 32- 137/Alg = 106AA ||[Chlorocebus
--------------------------------------------------------------------------------
111 XP_007943021   140AAs | 30- 135/Alg = 106AA ||[Orycteropus
--------------------------------------------------------------------------------
112 XP_007608803   140AAs | 30- 135/Alg = 106AA ||[Cricetulus g
--------------------------------------------------------------------------------
113 JAC52726       139AAs | 28- 133/Alg = 106AA ||[Bactrocera dorsalis].
--------------------------------------------------------------------------------
114 XP_007462141   140AAs | 30- 135/Alg = 106AA ||[Lipotes vexi
--------------------------------------------------------------------------------
115 XP_007174388   140AAs | 30- 135/Alg = 106AA ||[Balaenoptera
--------------------------------------------------------------------------------
116 XP_006050940   140AAs | 30- 135/Alg = 106AA ||[Bubalus buba
--------------------------------------------------------------------------------
117 XP_007070374   141AAs | 31- 136/Alg = 106AA ||[Chelonia mydas].
--------------------------------------------------------------------------------
118 XP_007102658   140AAs | 30- 135/Alg = 106AA ||[Physeter cat
--------------------------------------------------------------------------------
119 XP_006901562   140AAs | 30- 135/Alg = 106AA ||[Elephantulus
--------------------------------------------------------------------------------
120 XP_006746300   140AAs | 30- 135/Alg = 106AA ||[Leptonychote
--------------------------------------------------------------------------------
121 JAC05186       138AAs | 27- 132/Alg = 106AA ||[Ceratitis capitata].
--------------------------------------------------------------------------------
122 XP_005983463   140AAs | 30- 135/Alg = 106AA ||[Pantholops h
--------------------------------------------------------------------------------
123 JAB39795       142AAs | 32- 137/Alg = 106AA ||[Callithrix jacchus].
--------------------------------------------------------------------------------
124 XP_004051480   142AAs | 32- 137/Alg = 106AA ||[Gorilla goril
--------------------------------------------------------------------------------
```

125	AEJ84311	140AAs	30- 135/Alg = 106AA	[Capra hircus].
126	Q54SR7	133AAs	28- 133/Alg = 106AA	
127	XP_014452900	132AAs	32- 128/Alg = 97AA	[Alligator mi
128	XP_014354594	145AAs	35- 140/Alg = 106AA	[Papilio machaon].
129	JAN41653	138AAs	28- 133/Alg = 106AA	[Daphnia magna].
130	XP_014287548	143AAs	33- 138/Alg = 106AA	[Halyomorpha halys].
131	XP_014211126	151AAs	30- 135/Alg = 106AA	[Copidosoma floridanum].
132	XP_014090010	139AAs	28- 133/Alg = 106AA	[Bactrocera oleae].
133	XP_013186102	146AAs	36- 141/Alg = 106AA	[Amyelois transitella].
134	XP_004383846	140AAs	30- 135/Alg = 106AA	[Trichechus m
135	XP_004931279	146AAs	36- 141/Alg = 106AA	[Bombyx mori].
136	XP_012254607	150AAs	28- 133/Alg = 106AA	[Athalia rosae].
137	XP_004536978	138AAs	27- 132/Alg = 106AA	[Ceratitis capitata].
138	XP_785999	142AAs	30- 135/Alg = 106AA	[Strongylocentrotus purp
139	XP_011560028	137AAs	27- 132/Alg = 106AA	[Plutella xylostella].
140	XP_011211190	139AAs	28- 133/Alg = 106AA	[Bactrocera dorsalis].
141	KFO28453	140AAs	30- 135/Alg = 106AA	[Fukomys damarensis].
142	XP_001600159	147AAs	26- 131/Alg = 106AA	[Nasonia vitripennis].
143	JAB53650	141AAs	31- 136/Alg = 106AA	[Micrurus fulvius].
144	KTG33021	138AAs	28- 133/Alg = 106AA	[Cyprinus carpio].
145	JAI11984	141AAs	31- 136/Alg = 106AA	[Crotalus adamanteus].
146	JAA96089	141AAs	31- 136/Alg = 106AA	[Crotalus horridus].

Figure S1A. MSA of hFKBP13 (Fkbp2) and some of its orthologues expressed in miscellaneous species (see Fig. S1).

```
NP_004461         QIGVKKRV--DHCPIKSRKGDVLHMHYTG-KLEDGTEFDSSLPQNQPFVFSLGTGQVIKG
NP_001032558      QIGVKKRV--DHCPIKSRKGDVLHMHYTG-KLEDGTEFDSSLPQNQPFVFSLGTGQVIKG
NP_032046         QIGVKKRV--DHCPIKSRKGDVLHMHYTG-KLEDGTEFDSSLPQNQPFVFSLGTGQVIKG
NP_001253744      QIGVKKRV--DHCPIKSRKGDVLHMHYTG-KLEDGTEFDSSLPQNQPFVFSLGTGQVIKG
NP_001127900      QIGVKKRV--DHCPIKSRKGDVLHMHYTG-KLEDGTEFDSSLPQNQPFVFSLGTGQVIKG
NP_001107353      QIGVKKRV--ENCLVKSRKGDTLHMHYTG-KLEDGTEFDSSIPRNQAFTFTLGTGQVIKG
NP_001004677      QIGIKKRV--DNCPIKSRKGDVLNMHYTG-KLEDGTEFDSSIPRNQPFTFTLGTGQVIKG
NP_001158699      QIGIKKRV--DNCPIKSRKGDVLNMHYTG-KLEDGTEFDSSIPRNQPFTFTLGTGQVIKG
NP_001188070      QIGIKKRV--ENCPIKSRKGDVLNMHYTG-RLEDGTEFDSSIPRNQPFTFTLGTGQVIKG
NP_001079493      QIGVKKRV--ENCPVKSRKGDTLHMHYTG-KLEDGTEFDSSIPRNQPFTFTLGTGQVIKG
NP_001299353      QIGVKKRP--SECPIKSRKGDLLHMHYTG-TLEDGTEFDSSIPRGNPLTFTLGSGQVIKG
NP_010807         EIGIIKRIPVEDCLIKAMPGDKVKVHYTGSLLESGTVFDSSYSRGSPIAFELGVGRVIKG
JAO69915          QIGIKKRV--DNCPIKSRKGDVLNMHYTG-RLEDGTEFDSSIPRGQPFTFTLGTGQVIKG
XP_016084356      QIGIKKRV--DNCPIKSRKGDVLNMHYTG-KLEDGTEFDSSIPRNQPFTFTLGTGQVIKG
XP_016071878      QIGVKKRV--EHCPIKSRKGDVLHMHYTG-KLEDGTEFDSSLPQNQPFVFSLGTGQVIKG
XP_016041686      QIGVKKRV--DHCPIKSRKGDVLHMHYTG-KLEDGTEFDSSLPQNQPFVFSLGTGQVIKG
XP_015974039      QIGVKKRV--DHCPIKSRKGDVLHMHYTG-KLEDGTEFDSSLPQNQPFVFSLGTGQVIKG
XP_006980788      QIGVKKRV--DHCPIKSRKGDVLHMHYTG-KLEDGTEFDSSLPQNQPFVFSLGTGQVIKG
XP_015826586      QIGIKKRV--DNCPIKSRKGDVLNMHYTG-KLEDGTEFDSSIPRDRPFTFTLGTGQVIKG
XP_015744729      QIGVKKRV--DNCPIKSRKGDVLHMHYTG-KLEDGTEFDSSLTRHQPFIFSLGTGQVIKG
XP_015683908      QIGVKKRV--DNCPVKSRKGDVLHMHYTG-KLEDGTEFDSSLTRDQPFIFSLGTGQVIKG
XP_007244563      QIGIKKRV--DNCPIKSRKGDVLNMHYTG-KLEDGTEFDSSIPRNQPFTFTLGTGQVIKG
XP_006911221      QIGVKKRV--DHCPIKSRKGDVLHMHYTG-KLEDGTEFDSSLPQNQPFVFSLGTGQVIKG
XP_006771685      QIGVKKRV--DHCPIKSRKGDVLHMHYTG-KLEDGTEFDSSLPQNQPFVFSLGTGQVIKG
XP_015397493      QIGVKKRV--DHCPIKSRKGDVLHMHYTG-KLEDGTEFDSSLPQNQPFVFSLGTGQVIKG
XP_015333657      QIGVKKRV--DHCPIKSRKGDVLHMHYTG-KLEDGTEFDSSLPQNQPFVFSLGTGQVIKG
XP_005577499      QIGVKKRV--DHCPIKSRKGDVLHMHYTG-KLEDGTEFDSSLPQNQPFVFSLGTGQVIKG
XP_015272310      QIGVKKRV--DNCPIKSRKGDVLHMHYTG-KLEDGTEFDSSLTRDQPFVFTLGTGQVIKG
XP_015248799      QIGIKKRV--DNCPIKSRKGDVLNMHYTG-KLEDGTEFDSSISRDRPFTFTLGTGQVIKG
XP_015105405      QIGVKKRV--DHCPIKSRKGDVLHMHYTG-KLEDGTEFDSSLPQNQPFVFSLGTGQVIKG
XP_014969226      QIGVKKRV--DHCPIKSRKGDVLHMHYTG-KLEDGTEFDSSLPQNQPFVFSLGTGQVIKG
XP_011957484      QIGVKKRV--DHCPIKSRKGDVLHMHYTG-KLEDGTEFDSSLPQNQPFVFSLGTGQVIKG
XP_014856097      QIGIKKRV--DNCPIKSRKGDVLNMHYTG-KLEDGTEFDSSIPRGQPFTFTLGTGQVIKG
XP_014935343      QIGVKKRV--DHCPIKSRKGDVLHMHYTG-KLEDGTEFDSSLPQNQPFVFSLGTGQVIKG
XP_014748502      QIGVRRRP--ERCGDRSRRGDVLTLHYSG-TLEDGTLFDSSLSREQPFVFSLGTGQVIKG
XP_014700413      QIGVKKRV--DHCPIKSRKGDVLHMHYTG-KLEDGTEFDSSLPQNQPFVFSLGTGQVIKG
XP_006149888      QIGVKKRV--DHCPIKSRKGDVLHMHYTG-KLEDGTEFDSSLPQNQPFVFSLGTGQVIKG
XP_005864247      QIGVKKRV--DHCPIKSRKGDVLHMHYTG-KLEDGTEFDSSLPQNQPFVFSLGTGQVIKG
XP_006176941      QIGVKKRV--DHCPIKSRKGDVLHMHYTG-KLEDGTEFDSSLPQNQPFVFSLGTGQVIKG
XP_006129809      QIGVKKRV--ENCPIKSRKGDVLHMHYTG-KLEDGTEFDSSIPRDQPFIFSLGTGQVIKG
XP_006036922      QIGVKRRV--ENCGLRSRKGDVLTMHYTG-RLEDGSEFDSSLGRAQPFVFSLGTGQVIKG
XP_005988896      QIGVKKRV--DNCLVRSRKGDILHMHYTG-MLEDGTEFDSSIPRNQPFTFTLGTGQVIKG
XP_005894189      QIGVKKRV--DHCPIKSRKGDVLHMHYTG-KLEDGTEFDSSLPQNQPFVFSLGTGQVIKG
XP_005808854      QIGIKKRV--DNCPIKSRKGDVLNMHYTG-KLEDGTEFDSSIPRDRPFTFTLGTGQVIKG
XP_006106614      QIGVKKRV--DHCPIKSRKGDVLHMHYTG-KLEDGTEFDSSLPQNQPFVFSLGTGQVIKG
XP_008952214      QIGVKKRV--DHCPIKSRKGDVLHMHYTG-KLEDGTEFDSSLPQNQPFVFSLGTGQVIKG
XP_013976757      QIGVKKRV--DHCPIKSRKGDVLHMHYTG-KLEDGTEFDSSLPQNQPFVFSLGTGQVIKG
XP_003828606      QIGVKKRV--DHCPIKSRKGDVLHMHYTG-KLEDGTEFDSSLPQNQPFVFSLGTGQVIKG
XP_013873214      QIGIKKRV--DNCPIKSRKGDVLNMHYTG-KLEDGTEFDSSIPRDRPFTFTLGTGQVIKG
XP_005660807      QIGVKKRV--DHCPIKSRKGDVLHMHYTG-KLEDGTEFDSSLPQNQPFVFSLGTGQVIKG
XP_005699908      QIGVKKRV--DHCPIKSRKGDVLHMHYTG-KLEDGTEFDSSLPQNQPFVFSLGTGQVIKG
XP_005736999      QIGIKKRV--DNCPIKSRKGDVLNMHYTG-KLEDGTEFDSSIPRDRPFTFTLGTGQVIKG
XP_005384688      QIGVKKRV--DHCPIKSRKGDVLHMHYTG-KLEDGTEFDSSLPQNQPFVFSLGTGQVIKG
XP_005333562      QIGVKKRV--DHCPIKSRKGDVLHMHYTG-KLEDGTEFDSSLPQNQPFVFSLGTGQVIKG
XP_003452386      QIGIKKRV--DNCPIKSRKGDVLNMHYTG-KLEDGTEFDSSIPRDRPFTFTLGTGQVIKG
XP_005351906      QIGVKKRV--DHCPIKSRKGDVLHMHYTG-KLEDGTEFDSSLPKNQPFVFSLGTGQVIKG
XP_013006176      QIGVKKRV--DHCPIKSRKGDVLHMHYTG-KLEDGTEFDSSLPQNQPFVFSLGTGQVIKG
```

```
JAI39202        KIGIKKRA--ENCEVKARKGDTIHVHYRG-TLKDGTEFDNSYDRNKAFSVRLGAGQVIKG
XP_012975627    QIGVKKRV--DHCPIKSRKGDVLHMHYTG-KLEDGTEFDSSLPQNQPFVFSLGTGQVIKG
XP_010898827    QIGIKKRV--DNCPIKSRKGDVLNMHYTG-KLEDGTEFDSSIPRNQPFTFTLGTGQVIKG
XP_004852587    QIGVKKRV--DHCPIKSRKGDVLHMHYTG-KLEDGTEFDSSLPQNQPFVFSLGTGQVIKG
XP_004770232    QIGVKKRV--DHCPIKSRKGDVLHMHYTG-KLEDGTEFDSSLPQNQPFVFSLGTGQVIKG
XP_012876646    QIGVKKRV--DHCPIKSRKGDVLHMHYTG-KLEDGTEFDSSLPQNQPFVFSLGTGQVIKG
XP_012816015    QIGVKKRV--ENCLVKSRKGDTLHMHYTG-KLEDGTEFDSSIPRNQAFTFTLGTGQVIKG
XP_004656659    QIGVKKRV--DHCPIKSRKGDVLHMHYTG-KLEDGTEFDSSLPQNQPFVFSLGTGQVIKG
XP_004618432    QIGVKKRV--DHCPIKSRKGDVLHMHYTG-KLEDGTEFDSSLPKNQPFVFSLGTGQVIKG
XP_012666155    QIGVKKRV--DHCPMKSRKGDVLHMHYTG-KLEDGTEFDSSLPQNQPFVFSLGTGQVIKG
XP_004596785    QIGVKKRV--EHCPVKSRKGDVLHMHYTG-KLEDGTEFDSSLPQNQPFVFSLGTGQVIKG
XP_012581059    QIGVKKRV--DHCPIKSRKGDVLHMHYTG-KLEDGTEFDSSLPQNQPFVFSLGTGQVIKG
XP_012633386    QIGVKKRV--DHCPIKSRKGDVLHMHYTG-KLEDGTEFDSSLPQNQPFVFSLGTGQVIKG
XP_012520835    QIGVKKRV--DHCPIKSRKGDVLHMHYTG-KLEDGTEFDSSLPQNQPFVFSLGTGQVIKG
XP_004394068    QIGVKKRV--DHCPIKSRKGDVLHMHYTG-KLEDGTEFDSSLPQNQPFVFSLGTGQVIKG
XP_012408303    QIGVKKRV--DHCPLKSRKGDVLHIHYTG-RLEDGTEFDSSLHRDQPFVFSLGTGQVIKG
XP_004264331    QIGVKKRV--DHCPIKSRKGDVLHMHYTG-KLEDGTEFDSSLPQNQPFVFSLGTGQVIKG
XP_012351963    QIGVKKRV--DHCPIKSRKGDVLHMHYTG-KLEDGTEFDSSLPQNQPFVFSLGTGQVIKG
XP_004643825    QIGVKKRV--DHCPIKSRKGDVLHMHYTG-KLEDGTEFDSSLPQNQPFVFSLGTGQVIKG
XP_004643824    QIGVKKRV--DHCPIKSRKGDVLHMHYTG-KLEDGTEFDSSLPQNQPFVFSLGTGQVIKG
XP_004479918    QIGVKKRV--DHCPIKSRKGDVLHMHYTG-KLEDGTEFDSSLPQNQPFVFSLGTGQVIKG
XP_003274202    QIGVKKRV--DHCPIKSRKGDVLHMHYTG-KLEDGTEFDSSLPQNQPFVFSLGTGQVIKG
XP_004479917    QIGVKKRV--DHCPIKSRKGDVLHMHYTG-KLEDGTEFDSSLPQNQPFVFSLGTGQVIKG
XP_012318520    QIGVKKRV--DHCPIKSHKWDVLHMHYTG-KLEDGTEFDSSLPQNQPFVFSLGTGQVIKG
XP_011718910    QIGVKKRV--DHCPIKSRKGDVLHMHYTG-KLEDGTEFDSSLPQNQPFVFSLGTGQVIKG
XP_011897294    QIGVKKRV--DHCPIKSRKGDVLHMHYTG-KLEDGTEFDSSLPQNQPFVFSLGTGQVIKG
XP_011848866    QIGVKKRV--DHCPIKSRKGDVLHMHYTG-KLEDGTEFDSSLPQNQPFVFSLGTGQVIKG
XP_011797983    QIGVKKRV--DHCPIKSRKGDVLHMHYTG-KLEDGTEFDSSLPQNQPFVFSLGTGQVIKG
XP_003974414    QIGIKKRV--DHCTTKSRKGDVLNMHYTG-KLEDGTEFDSSISRDKPFTFTLGKGQVIKG
XP_004068088    QIGIKKRV--DNCPIKSRKGDVLNMHYTG-KLEDGTEFDSSIPRDRPFTFTLGTGQVIKG
XP_011367027    QIGVKKRV--DHCPIKSRKGDVLHMHYTG-KLEDGTEFDSSLPQNQPFVFSLGTGQVIKG
XP_006937524    QIGVKKRV--DHCPIKSRKGDVLHMHYTG-KLEDGTEFDSSLPQNQPFVFSLGTGQVIKG
XP_011219931    QIGVKKRV--DHCPIKSRKGDVLHMHYTG-KLEDGTEFDSSLPQNQPFVFSLGTGQVIKG
XP_010993050    QIGVKKRV--DHCPIKSRKGDVLHMHYTG-KLEDGTEFDSSLPQNQPFVFSLGTGQVIKG
XP_010836289    QIGVKKRV--DHCPIKSRKGDVLHMHYTG-KLEDGTEFDSSLPQNQPFVFSLGTGQVIKG
XP_010770745    QIGIKKRV--DNCPIKSRKGDVLNMHYTG-KLEDGTEFDSSIPRDRPFTFTLGTGQVIKG
XP_010752642    QIGIKKRV--DNCPMKSRKGDVLNMHYTG-KLEDGTEFDSSIPRDRPFTFTLGTGQVIKG
XP_010597383    QIGVKKRV--DHCPIKSRKGDVLHMHYTG-KLEDGTEFDSSLPQNQPFVFSLGTGQVIKG
XP_010634398    QIGVKKRV--DHCPIKSRKGDVLHMHYTG-KLEDGTEFDSSLPQNQPFVFSLGTGQVIKG
XP_010346544    QIGVKKRV--DHCPIKSRKGDVLHMHYTG-KLEDGTEFDSSLPQNQPFVFSLGTGQVIKG
XP_010375362    QIGVKKRV--DHCPIKSRKGDVLHMHYTG-KLEDGTEFDSSLPQNQPFVFSLGTGQVIKG
XP_010375348    QIGVKKRV--DHCPIKSRKGDVLHMHYTG-KLEDGTEFDSSLPQNQPFVFSLGTGQVIKG
XP_009421628    QIGVKKRV--DHCPIKSRKGDVLHMHYTG-KLEDGTEFDSSLPQNQPFVFSLGTGQVIKG
XP_009244471    QIGVKKRV--DHCPIKSRKGDVLHMHYTG-KLEDGTEFDSSLPQNQPFVFSLGTGQVIKG
XP_003909669    QIGVKKRV--DHCPIKSRKGDVLHMHYTG-KLEDGTEFDSSLPQNQPFVFSLGTGQVIKG
XP_008837174    QIGVKKRV--DHCPIKSRKGDVLHMHYTG-KLEDGTEFDSSLPQNQPFVFSLGTGQVIKG
XP_008707188    QIGVKKRV--DHCPIKSRKGDVLHMHYTG-KLEDGTEFDSSLPQNQPFVFSLGTGQVIKG
XP_008565641    QIGVKKRV--DHCPIKSRKGDVLHMHYTG-KLEDGTEFDSSLPQNQPFVFSLGTGQVIKG
XP_008304826    QIGIKKRV--DNCPIKSRKGDVLNMHYTG-KLEDGTEFDSSIPRDRPFTFTLGTGQVIKG
XP_008255497    QIGVKKXV--DHCPIRSRKGDVLHMHYTG-KLEDGTEFDSSLPQKQPFVFSLGTGQVIKG
XP_005305586    QIGVKKRV--ENCPIKSRKGDVLHMHYTG-KLEDGTEFDSSIPRDQPFIFSLGTGQVIKG
XP_008047615    QIGVKKRV--DHCPVKSRKGDVLHMHYTG-KLEDGTEFDSSLPQNQPFVFSLGTGQVIKG
XP_007992347    QIGVKKRV--DHCPIKSRKGDVLHMHYTG-KLEDGTEFDSSLPQNQPFVFSLGTGQVIKG
XP_007943021    QIGVKKRV--DHCPIKSRKGDVLHMHYTG-KLEDGTEFDSSLPQNQPFVFSLGTGQVIKG
XP_007608803    QIGVKKRV--DHCPIKSRKGDVLHMHYTG-KLEDGTEFDSSLPQNQPFVFTLGTGQVIKG
JAC52726        KIGIKKRA--ENCEVKARKGDTIHVHYRG-TLKDGTEFDSSYDRNKAFSVRLGAGQVIKG
XP_007462141    QIGVKKRV--DHCPIKSRKGDVLHMHYTG-KLEDGTEFDSSLPQNQPFVFSLGTGQVIKG
XP_007174388    QIGVKKRV--DHCPIKSRKGDVLHMHYTG-KLEDGTEFDSSLPQNQPFVFSLGTGQVIKG
XP_006050940    QIGVKKRV--DHCPIKSRKGDVLHMHYTG-KLEDGTEFDSSLPQNQPFVFSLGTGQVIKG
XP_007070374    QIGVKKRV--ENCPIKSRKGDVLHMHYTG-KLEDGTEFDSSIPRDQPFIFSLGTGQVIKG
```

```
XP_007102658        QIGVKKRV--DHCPIKSRKGDVLHMHYTG-KLEDGTEFDSSLPQNQPFVFSLGTGQVIKG
XP_006901562        QIGVKKRV--DHCPIKSRKGDVLHMHYTG-KLEDGTEFDSSLPQNQPFVFSLGTGQVIKG
XP_006746300        QIGVKKRV--DHCPIKSRKGDVLHMHYTG-KLEDGTEFDSSLPQNQPFVFSLGTGQVIKG
JAC05186            RIGIKKRA--ENCEIKARKGDTIHVHYRG-TLKDGTEFDSSYGRNKAFSVRLGAGQVIKG
XP_005983463        QIGVKKRV--DHCPIKSRKGDVLHMHYTG-KLEDGTEFDSSLPQNQPFVFSLGTGQVIKG
JAB39795            QIGVKKRV--DHCPIKSRKGDVLHMHYTG-KLEDGTEFDSSLPQNQPFVFSLGTGQVIKG
XP_004051480        QIGVKKRV--DHCPIKSRKGDVLHMHYTG-KLEDGTEFDSSLPQNQPFVFSLGTGQVIKG
AEJ84311            QIGVKKRV--DHCPIKSRKGDVLHMHYTG-SLEDGTEFDSSLPQNQPFVFSLGTGQVIKG
Q54SR7              QIGVKYRP--DECTVKTKSGDKLKIHYTG-TLLNGDKFDSSVDRGTPFEFKIGVGQVIKG
XP_014452900        QIGVKRRV--ENCGLRSRKGDVLTMHYTG-RLEDGSEFDSSLGRAQPFVFSLGTGQVIKG
XP_014354594        QIGVKKRP--SECPIKSRKGDLLHMHYTG-TLEDGTEFDSSLPRGNPLTFTLGSGQVIKG
JAN41653            QIGIKKRV--DNCTVKSKRGDLLHMHYTG-TLEDGTEFDSSIPRGQPLTFTLGSGQVIKG
XP_014287548        QIGIKKRI--DNCTKKSVKGDLLHMHYTG-KLEDGTVFDSSYDRKQPLTFTLGFGQVIRG
XP_014211126        QIGVKKRV--DNCTLRSKRGDTLVVNYVG-TLEDGTEFDSSSNYDDSFFITLGYGQVIKG
XP_014090010        KIGIKKRV--ENCEVKARKGDTIHVHYRG-TLKDGTEFDSSYDRNKAFSVRLGAGQVIKG
XP_013186102        QIGVKKRP--AECPIKSRKGDLLHMHYTG-TLEDGTEFDSSIPRGNPLTFTLGSGQVIKG
XP_004383846        QIGVKKRV--DHCPIKSRKGDVLHMHYTG-KLEDGTEFDSSLPQNQPFVFSLGTGQVIKG
XP_004931279        QIGIKTRP--EDCSIKSRKGDLLHMHYTG-TLDDGTEFDSSIPRGNPLTFKLGSGQVIKG
XP_012254607        QIGIKKRV--EKCNVRSKKGDTLYVHYVG-TLEDGTEIDSSSIHGEPLPVTLGYRQVIKG
XP_004536978        RIGIKKRA--ENCEIKARKGDTIHVHYRG-TLKDGTEFDSSYGRNKAFSVRLGAGQVIKG
XP_785999           QIGIKKKV--EDCTTRSRTGDRLSMHYTG-KLEDGTEFDSSIPRKQTFDFTLGAGQVIKG
XP_011560028        QIGIKKRP--AECSMKTRKGDLLHIHYTG-TLVDGTEFDSSYSRKMPITFTLGSGQVIKG
XP_011211190        KIGIKKRA--ENCEVKARKGDTIHVHYRG-TLKDGTEFDSSYDRNKAFSVRLGAGQVIKG
KFO28453            QIGVKKRV--DHCPIKSRKGDVLHMHYTG-KLEDGTEFDSSLPQNQPFVFSLGTGQVIKG
XP_001600159        QIGIKKRV--DNCTLKSKRGDTLFVNYVG-TLEDGTEFDKSSNYEDSFLVTLGYGQVIKG
JAB53650            QIGVKKRV--DNCPIKSRKGDVLHMHYTG-KLEDGTEFDSSLTRDQPFIFSLGTGQVIKG
KTG33021            QIGIKKRV--DNCPIKSRKGDVLNMHYTG-KLEDGTEFDSSIPRNQPFTFTLGTGQVIKG
JAI11984            QIGVKKRV--DNCPVKSRKGDVLHMHYTG-KLEDGTEFDSSLTRDQPFIFSLGTGQVIKG
JAA96089            QIGVKKRV--DNCPIKSRKGDVLHMHYTG-KLEDGTEFDSSLTRDQPFIFSLGTGQVIKG
                    .**:        *  ::   * : ::* *  * .*  :*.*     .: . :*  :**:*
```

NP_004461	WDQGLLGMCEGEKRKLVIPSELGYGERGAPPKIPGGATLVFEVELLKIE
NP_001032558	WDQGLLGMCEGEKRKLVIPSELGYGERGAPPKIPGGATLVFEVELLKIE
NP_032046	WDQGLLGMCEGEKRKLVIPSELGYGERGAPPKIPGGATLVFEVELLKIE
NP_001253744	WDQGLLGMCEGEKRKLVIPSELGYGERGAPPKIPGGATLVFEVELLKIE
NP_001127900	WDQGLLGMCEGEKRKLVIPSELGYGERGAPPKIPGGATLVFEVELLKIE
NP_001107353	WDQGLLGMCEGEKRKLVIPSELGYGDRGAPPKIPGGATLIFEVELVKIE
NP_001004677	WDQGLLGMCEGEKRKLVIPSELGYGDRGAPPKIPGGATLIFEVELLNIE
NP_001158699	WDQGLLGMCEGEKRKLVIPSELGYGDRGAPPKIPGGATLIFEVELLSIE
NP_001188070	WDQGLLGMCEGEKRKLVIPSELGYGDRGAPPKIPGGATLIFEVELLSIE
NP_001079493	WDQGLLGMCEGEKRKLVIPSELGYGDRGAPPKIPGGATLIFEVELLKIE
NP_001299353	WDQGLIGMCEGEQRKLVIPPELAYGENGSPPKIPKSATLTFHVDLVKIE
NP_010807	WDQGVAGMCVGEKRKLQIPSSLAYGERGVPGVIPPSADLVFDVELVDVK
JAO69915	WDQGLLGMCEGEKRKLVIPAELGYGDRGAPPKIPGGATLIFEVELLSIE
XP_016084356	WDQGLLGMCEGEKRKLVIPSELGYGDRGAPPKIPGGATLIFEVELLSIE
XP_016071878	WDQGLLGMCEGEKRKLVIPSELGYGERGAPPKIPGGATLVFEVELLKIE
XP_016041686	WDQGLLGMCEGEKRKLVIPSELGYGERGAPPKIPGGATLVFEVELLKIE
XP_015974039	WDQGLLGMCEGEKRKLVIPSELGYGERGAPPKIPGGATLVFEVELLKIE
XP_006980788	WDQGLLGMCEGEKRKLVIPSELGYGERGAPPKIPGGATLVFEVELLKIE
XP_015826586	WDQGLLGMCEGEKRKLVIPAELGYGDRGAPPKIPGGATLIFEVELLSIE
XP_015744729	WDQGLLGMCEGEKRKLVIPSELGYGDRGAPPKIPGGATLIFEVELLKIE
XP_015683908	WDQGLLGMCEGEKRKLVIPSELGYGDRGAPPKIPGGATLIFEVELLKIE
XP_007244563	WDQGLLGMCEGEKRKLVIPSELGYGDKGAPPKIPGGATLIFEVELLSIE
XP_006911221	WDQGLLGMCEGEKRKLVIPSELGYGERGAPPKIPGGATLVFEVELLKIE
XP_006771685	WDQGLLGMCEGEKRKLVIPSELGYGERGAPPKIPGGATLVFEVELLKIE
XP_015397493	WDQGLLGMCEGEKRKLVIPSELGYGERGAPPKIPGGATLVFEVELLKIE
XP_015333657	WDQGLLGMCEGEKRKLVIPSELGYGERGAPPKIPGGATLVFEVELLKIE
XP_005577499	WDQGLLGMCEGEKRKLVIPSELGYGERGAPPKIPGGATLVFEVELLKIE
XP_015272310	WDQGLLGMCEGEKRKLVIPSELGYGDRGAPPKIPGGATLIFEVELLKIE
XP_015248799	WDQGLLGMCEGEKRKLVIPAELGYGDRGAPPKIPGGATLIFEVELLSIE
XP_015105405	WDQGLLGMCEGEKRKLVIPSELGYGERGAPPKIPGGATLVFEVELLKIE
XP_014969226	WDQGLLGMCEGEKRKLVIPSELGYGERGAPPKIPGGATLVFEVELLKIE
XP_011957484	WDQGLLGMCEGEKRKLVIPSELGYGERGAPPKIPGGATLVFEVELLKIE
XP_014856097	WDQGLLGMCEGEKRKLVIPAELGYGDRGAPPKIPGGATLIFEVELLSIE
XP_014935343	WDQGLLGMCEGEKRKLVIPSELGYGERGAPPKIPGGATLVFEVELLKIE
XP_014748502	WDQGLLGMCEGEKRKLVIPPELGYGDRGAPPKIPGGAVLIFEVELLKIE
XP_014700413	WDQGLLGMCEGEKRKLVIPSELGYGERGAPPKIPGGATLVFEVELLKIE
XP_006149888	WDQGLLGMCEGEKRKLVIPSELGYGERGAPPKIPGGATLVFEVELLKIE
XP_005864247	WDQGLLGMCEGEKRKLVIPSELGYGERGAPPKIPGGATLVFEVELLKIE
XP_006176941	WDQGLLGMCEGEKRKLVIPSELGYGERGAPPKIPGGATLVFEVELLKIE
XP_006129809	WDQGLLGMCEGEKRKLVIPSELGYGDRGAPPKIPGKATVPPPCLVPACP
XP_006036922	WDQGLLGMCEGEKRKLVIPSELGYGDRGAPPKIPGGATLVFEVELLKIE
XP_005988896	WDQGLLGMCEGEKRKLVIPSELGYGDRGAPPKIPGGATLIFEVELLKIE
XP_005894189	WDQGLLGMCEGEKRKLVIPSELGYGERGAPPKIPGGATLVFEVELLKIE
XP_005808854	WDQGLLGMCEGEKRKLVIPAELGYGDRGAPPKIPGGATLIFEVELLSIE
XP_006106614	WDQGLLGMCEGEKRKLVIPSELGYGERGAPPKIPGGATLVFEVELLKIE
XP_008952214	WDQGLLGMCEGEKRKLVIPSELGYGERGAPPKIPGGATLVFEVELLKIE
XP_013976757	WDQGLLGMCEGEKRKLVIPSELGYGERGAPPKIPGGATLVFEVELLKIE
XP_003828606	WDQGLLGMCEGEKRKLVIPSELGYGERGAPPKIPGGATLVFEVELLKIE
XP_013873214	WDQGLLGMCEGEKRKLVIPSELGYGDRGAPPKIPGGATLIFEVELLSIE
XP_005660807	WDQGLLGMCEGEKRKLVIPSELGYGERGAPPKIPGGATLVFEVELLKIE
XP_005699908	WDQGLLGMCEGEKRKLVIPSELGYGERGAPPKIPGGATLVFEVELLKIE
XP_005736999	WDQGLLGMCEGEKRKLVIPAELGYGDRGAPPKIPGGATLIFEVELLSIE
XP_005384688	WDQGLLGMCEGEKRKLVIPSELGYGERGAPPKIPGGATLVFEVELLKIE
XP_005333562	WDQGLLGMCEGEKRKLVIPSELGYGERGAPPKIPGGATLVFEVELLKIE
XP_003452386	WDQGLLGMCEGEKRKLVIPAELGYGDRGAPPKIPGGATLIFEVELLSIE
XP_005351906	WDQGLLGMCEGEKRKLVIPSELGYGERGAPPKIPGGATLVFEVELLKIE
XP_013006176	WDQGLLGMCEGEKRKLVIPSELGYGERGAPPKIPGGATLVFEVELLKIE
JAI39202	WDQGLLGMCEGEKRRLVIPPELGYGKAGAGDKIPPDATLVFDVELEKID
XP_012975627	WDQGLLGMCEGEKRKLVIPSELGYGERGAPPKIPGGATLVFEVELLKIE
XP_010898827	WDQGLLGMCEGEKRKLVIPSELGYGDRGAPPKIPGGATLIFEVELLNIE

XP_004852587	WDQGLLGMCEGEKRKLVIPSELGYGERGAPPKIPGGATLVFEVELLKIE
XP_004770232	WDQGLLGMCEGEKRKLVIPSELGYGERGAPPKIPGGATLVFEVELLKIE
XP_012876646	WDQGLLGMCEGEKRKLVIPSELGYGERGAPPKIPGGATLVFEVELLKIE
XP_012816015	WDQGLLGMCEGEKRKLVIPSELGYGDRGAPPKIPGGATLIFEVELVKIE
XP_004656659	WDQGLLGMCEGEKRKLVIPSELGYGERGAPPKIPGGATLVFEVELLKIE
XP_004618432	WDQGLLGMCEGEKRKLVIPSELGYGERGAPPKIPGGATLVFEVELLKIE
XP_012666155	WDQGLLGMCEGEKRKLVIPSELGYGERGAPPKIPGGATLVFEVELLKIE
XP_004596785	WDQGLLGMCEGEKRKLVIPSELGYGERGAPPKIPGGATLVFEVELLKIE
XP_012581059	WDQGLLGMCEGEKRKLVIPSELGYGERGAPPKIPGGATLVFEVELLKIE
XP_012633386	WDQGLLGMCEGEKRKLVIPSELGYGERGAPPKIPGGATLVFEVELLKIE
XP_012520835	WDQGLLGMCEGEKRKLVIPSELGYGERGAPPKIPGGATLVFEVELLKIE
XP_004394068	WDQGLLGMCEGEKRKLVIPSELGYGERGAPPKIPGGATLVFEVELLKIE
XP_012408303	WDQGLLGMCEGEKRKLVIPSELGYGDRGAPPKIPGRATLVFEVELLKIE
XP_004264331	WDQGLLGMCEGEKRKLVIPSELGYGERGAPPKIPGGATLVFEVELLKIE
XP_012351963	WAQGLLGMCEGEKRKLVIPSELGYGERGAPPKIPGGATLVFEVELLKIE
XP_004643825	WDQGLLGMCEGEKRKLVIPSELGYGERGAPPKIPGGATLVFEVELLKIE
XP_004643824	WDQGLLGMCEGEKRKLVIPSELGYGERGAPPKIPGGATLVFEVELLKIE
XP_004479918	WDQGLLGMCEGEKRKLVIPSELGYGERGAPPKIPGGATLVFEVELLKIE
XP_003274202	WAQGLLGMCEGEKRKLVIPSELGYGERGAPPKIPGGATLVFEVELLKIE
XP_004479917	WDQGLLGMCEGEKRKLVIPSELGYGERGAPPKIPGGATLVFEVELLKIE
XP_012318520	WDQGLLGMCEGEKRKLVIPSELGYGERGAPPKIPGGATLVFEVELLKIE
XP_011718910	WDQGLLGMCEGEKRKLVIPSELGYGERGAPPKIPGGATLVFEVELLKIE
XP_011897294	WDQGLLGMCEGEKRKLVIPSELGYGERGAPPKIPGGATLVFEVELLKIE
XP_011848866	WDQGLLGMCEGEKRKLVIPSELGYGERGAPPKIPGGATLVFEVELLKIE
XP_011797983	WDQGLLGMCEGEKRKLVIPSELGYGERGAPPKIPGGATLVFEVELLKIE
XP_003974414	WDQGLLGMCEGEKRKLVIPADLGYGDRGAPPKIPGGATLIFEVELLSIE
XP_004068088	WDQGLLGMCEGEKRKLVIPAELGYGDRGAPPKIPGGATLIFEVELLSIE
XP_011367027	WDQGLLGMCEGEKRKLVIPSELGYGERGAPPKIPGGATLVFEVELLKIE
XP_006937524	WDQGLLGMCEGEKRKLVIPSELGYGERGAPPKIPGGATLVFEVELLKIE
XP_011219931	WDQGLLGMCEGEKRKLVIPSELGYGERGAPPKIPGGATLVFEVELLKIE
XP_010993050	WDQGLLGMCEGEKRKLVIPSELGYGERGAPPKIPGGATLVFEVELLKIE
XP_010836289	WDQGLLGMCEGEKRKLVIPSELGYGERGAPPKIPGGATLVFEVELLKIE
XP_010770745	WDQGLLGMCEGEKRKLVIPSELGYGDRGAPPKIPGGATLIFEVELLSID
XP_010752642	WDQGLLGMCEGEKRKLVIPAELGYGDRGAPPKIPGGATLIFEVELLSIE
XP_010597383	WDQGLLGMCEGEKRKLVIPSELGYGERGAPPKIPGGATLVFEVELLKIE
XP_010634398	WDQGLLGMCEGEKRKLVIPSELGYGERGAPPKIPGGATLVFEVELLKIE
XP_010346544	WDQGLLGMCEGEKRKLVIPSELGYGERGAPPKIPGGATLVFEVELLKIE
XP_010375362	WDQGLLGMCEGEKRKLVIPSELGYGERGAPPKIPGGATLVFEVELLKIE
XP_010375348	WDQGLLGMCEGEKRKLVIPSELGYGERGAPPKIPGGATLVFEVELLKIE
XP_009421628	WDQGLLGMCEGEKRKLVIPSELGYGERGAPPKIPGGATLVFEVELLKIE
XP_009244471	WDQGLLGMCEGEKRKLVIPSELGYGERGAPPKIPGGATLVFEVELLKIE
XP_003909669	WDQGLLGMCEGEKRKLVIPSELGYGERGAPPKIPGGATLVFEVELLKIE
XP_008837174	WDQGLLGMCEGEKRKLVIPSELGYGERGAPPKIPGGATLVFEVELLKIE
XP_008707188	WDQGLLGMCEGEKRKLVIPSELGYGERGAPPKIPGGATLVFEVELLKIE
XP_008565641	WDQGLLGMCEGEKRKLVIPSELGYGERGAPPKIPGGATLVFEVELLKIE
XP_008304826	WDQGLLGMCEGEKRKLVIPAELGYGDRGAPPKIPGGATLIFEVELLSIE
XP_008255497	WDQGLLGMCEGEKRKLVIPSELGYGERGAPPKIPGGAALVFQVELLKVE
XP_005305586	WDQGLLGMCEGEKRKLVIPSELGYGDRGAPPKIPGGATLIFEVELLKIE
XP_008047615	WDQGLLGMCEGEKRKLVIPSELGYGERGAPPKIPGGATLVFEVELLKIE
XP_007992347	WDQGLLGMCEGEKRKLVIPSELGYGERGAPPKIPGGATLVFEVELLKIE
XP_007943021	WDQGLLGMCEGEKRKLVIPSELGYGERGAPPKIPGGATLVFEVELLKIE
XP_007608803	WDQGLLGMCEGEKRKLVIPSELGYGERGAPPKIPGGATLVFEVELLKIE
JAC52726	WDQGLLGMCEGEKRRLVIPPELGYGKAGAGDKIPPDATLVFDVELEKID
XP_007462141	WDQGLLGMCEGEKRKLVIPSELGYGERGAPPKIPGGATLVFEVELLKIE
XP_007174388	WDQGLLGMCEGEKRKLVIPSELGYGERGAPPKIPGGATLVFEVELLKIE
XP_006050940	WDQGLLGMCEGEKRKLVIPSELGYGERGAPPKIPGGATLVFEVELLKIE
XP_007070374	WDQGLLGMCEGEKRKLVIPSELGYGDRGAPPKIPGGATLIFEVELLKIE
XP_007102658	WDQGLLGMCEGEKRKLVIPSELGYGERGAPPKIPGGATLVFEVELLKIE
XP_006901562	WDQGLLGMCEGEKRKLVIPSELGYGDRGAPPKIPGGATLVFEVELLKIE
XP_006746300	WDQGLLGMCEGEKRKLVIPSELGYGERGAPPKIPGGATLVFEVELLKIE

```
JAC05186          WEQGLLGMCEGEKRRLVIPPELGYGKSGAGDKIPPDATLVFEVECEKID
XP_005983463      WDQGLLGMCEGEKRKLVIPSELGYGERGAPPKIPGGATLVFEVELLKIE
JAB39795          WDQGLLGMCEGEKRKLVIPPELGYGDRGAPPKIPGGATLVFEVELLKIE
XP_004051480      WDQGLLGMCEGEKRKLVIPSELGYGERGAPPKIPGGATLVFEVELLKIE
AEJ84311          WAPGLLGMCEGEKRKLVIPSELGSGERGAPPKIPGGATLVFEVELLKIE
Q54SR7            WDQGVLGMCVGEKRKLIIPPSLGYGQQGAGDKIPGNSHLIFDVELIGIN
XP_014452900      WDQGLLGMCEGEKRKLVIPSELGYGDRAPCP---------LQVVQRWCS
XP_014354594      WDQGLIGMCEGEQRKLVIPPELAYGENGSPPKIPKSATLTFHVDLVKIE
JAN41653          WDQGLMGMCEGEKRKLVIPSELGYGASGAPPKIPGDATLIFNVELLKIE
XP_014287548      WDQGLMGMCEGEIRKLVIPPDLGYGKAGAPPKIPGGAVLTFDVELVKIE
XP_014211126      WEQGLIGMCVGEKRKLVIPPDLAYGSFGALPKIPPNATVIFTVELVKLV
XP_014090010      WDQGLLGMCEGEKRRLVIPPELGYGKAGAGDKIPPDSTLVFDVELERID
XP_013186102      WDQGLIGMCEGEQRKLVIPPELAYGEAGAPPKIPKSATLTFHVDLVKID
XP_004383846      WDQGLLGMCEGEKRKLVIPSELGYGERGAPPKIPGGATLVFEVELLKIE
XP_004931279      WDQGLLGMCEGEQRKLVIPPELAYGSAGAPPKIPKSATLTFHVELVKIE
XP_012254607      WEQGLMGMCEGEKRKLVIPSDLAYGSPGALPKVPPDATVIFMVELVKLV
XP_004536978      WEQGLLGMCEGEKRRLVIPPELGYGKSGAGDKIPPDATLVFEVECEKID
XP_785999         WDQGLLNMCEGEKRKLVIPSNLGYGDRGSPPKIPGGATLIFEVELIKIN
XP_011560028      WEQGLMGMCMGEQRKLVIPPELGYGQEGSPPKIPKSAILIFHVELVKIE
XP_011211190      WDQGLLGMCEGEKRRLVIPPELGYGKAGAGDKIPPDATLVFDVELEKID
KFO28453          WDQGLLGMCEGEKRKLVIPSELGYGERGAPPKIPGGATLVFEVELLKIE
XP_001600159      WEQGLMGMCVGEKRKLVIPPDLAYGSFGALPKIPPNSTVIFTVELVQLV
JAB53650          WEQGLLGMCEGEKRKLVIPSELGYGDRGAPPKIPGGATLIFEVELLKIE
KTG33021          WDQGLLGMCEGEKRKLVIPSELGYGDRGAPPKIPGGATLIFEVELLSIE
JAI11984          WDQGLLGMCEGEKRKLVIPSELGYGDRGAPPKIPGGATLIFEVELLKIE
JAA96089          WDQGLLGMCEGEKRKLVIPSELGYGDRGAPPKIPGGATLIFEVELLKIE
                  *  *: .** ** *:* **..*. *  .
```

Figure S2A

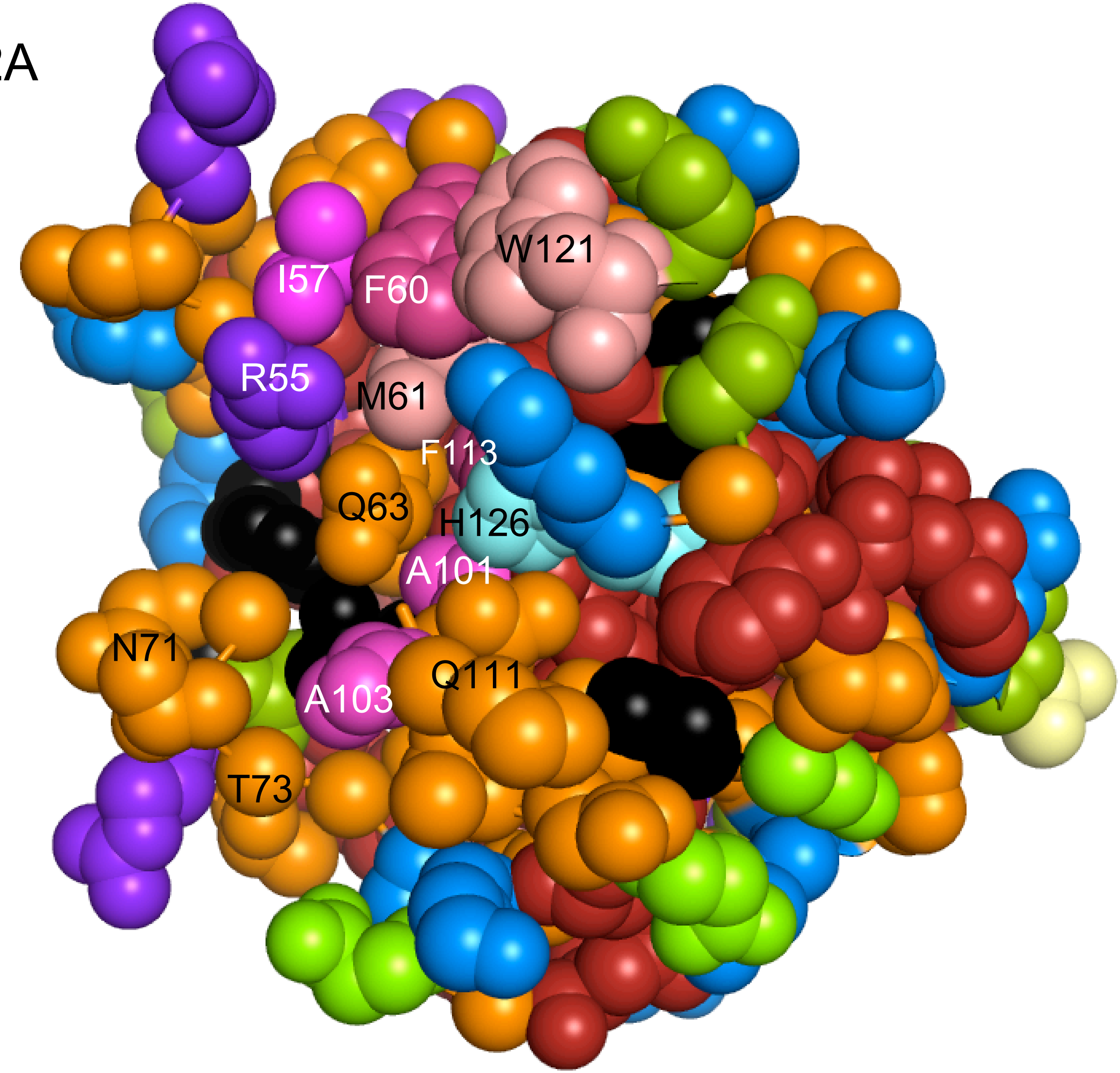

S2B

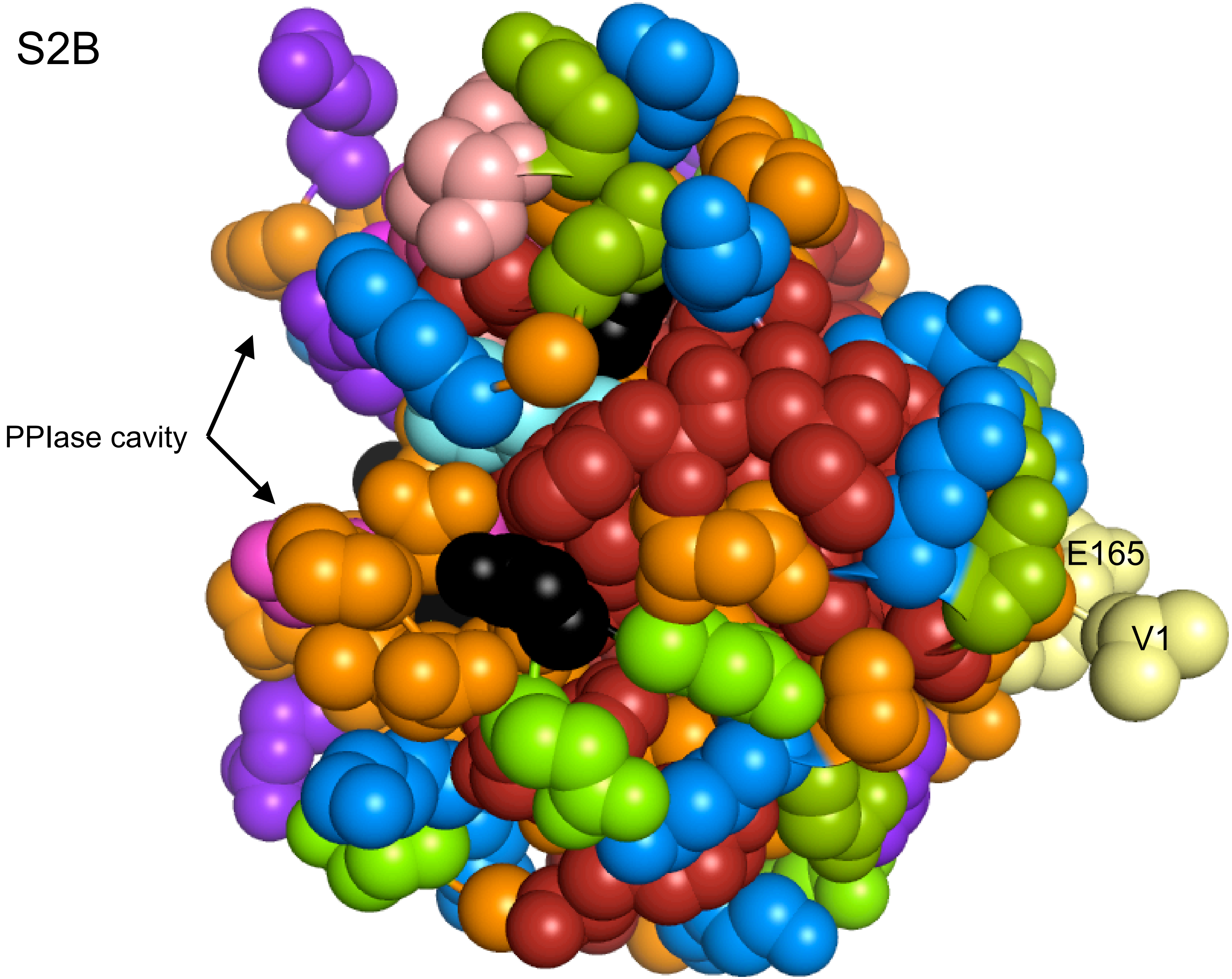

S2C

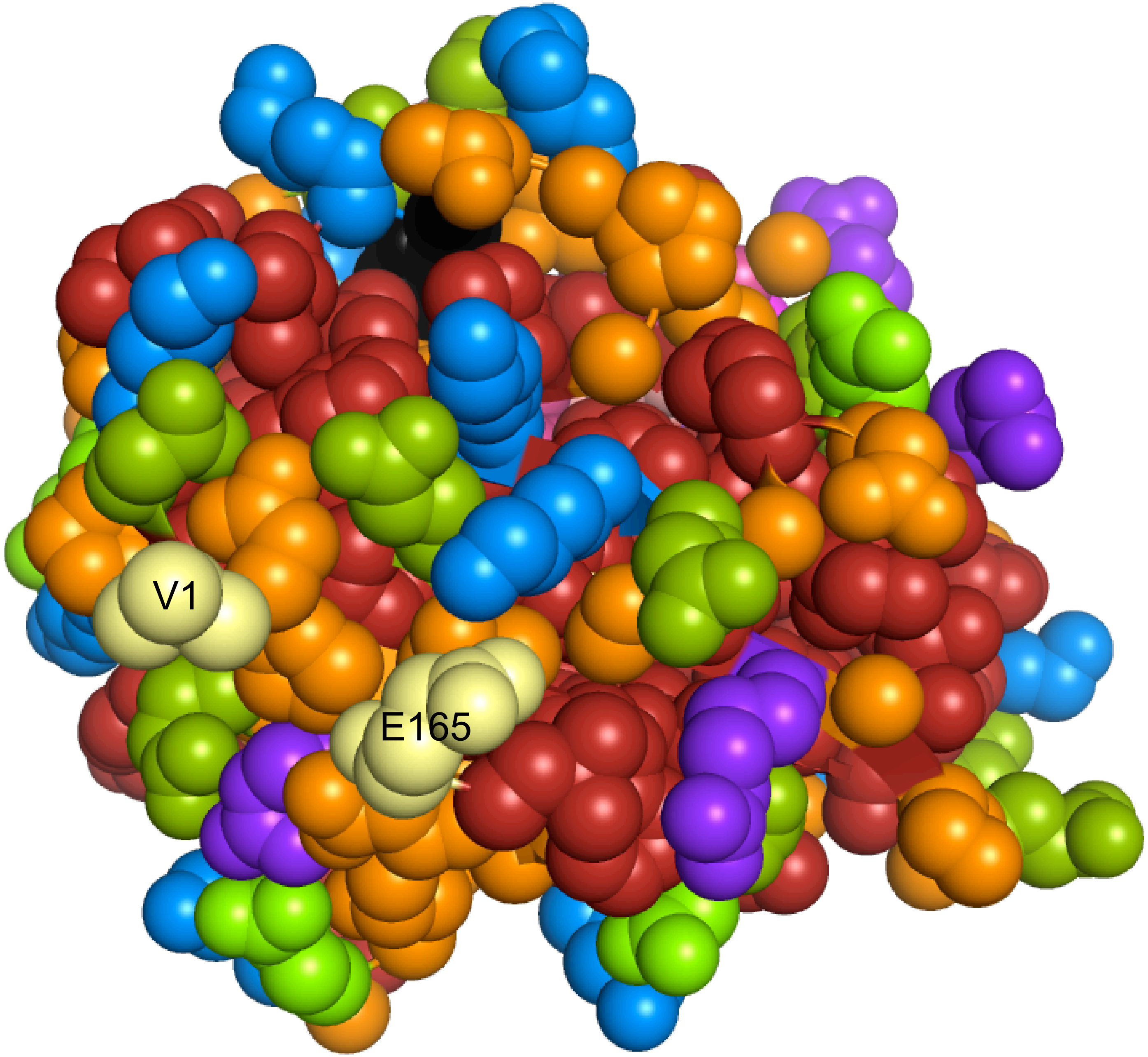

Figure S3. Alignment of nine fragments of sequences of mTOR from different species (see Fig. S3A)

```
NP_004949         -YTVPAVQGFFRSISLSRGNNLQDTLRVLTLWFDYGHWPDVNEALVEGVKAIQIDTWLQV
NP_064393         -YTVPAVQGFFRSISLSRGNNLQDTLRVLTLWFDYGHWPDVNEALVEGVKAIQIDTWLQV
NP_063971         -YTVPAVQGFFRSISLSRGNNLQDTLRVLTLWFDYGHWPDVNEALVEGVKAIQIDTWLQV
NP_001138927      MYTVPAVQGFFRSISLSRGNNLQDTLRVLTLWFDYGHWPDVNEALVEGVKAIQIDTWLQV
NP_001272677      -YTVPAVQGFFRSISLSRGNNLQDTLRVLTLWFDYGHWPDVNEALVEGVKAIQIDTWLQV
NP_001260427      ----PAVQGFFRSISLIKGNSLQDTLRLLTLWFDYGNHAEVYEALLSGMKLIEINTWLQV
NP_524891         RYAVPAVQGFFRSISLIKGNSLQDTLRLLTLWFDYGNHAEVYEALLSGMKLIEINTWLQV
AJV38810          ----PAIKGFFHSISLLETSCLQDTLRLLTLLFNFGGIKEVSQAMYEGFNLMKIENWLEV
AJS55673          -HVIPAIKGFFHSISLSESSSLQDALRLLTLWFTFGGIPEATQAMHEGFNLIQIGTWLEV
                      **::***:**** . . ***:**:*** * :*   :. :*: .*.: ::* .**:*

NP_004949         IPQLIARIDTPRPLVGRLIHQLLTDIGRYHPQALIYPLTVASKSTTTARHNAANKILKNM
NP_064393         IPQLIARIDTPRPLVGRLIHQLLTDIGRYHPQALIYPLTVASKSTTTARHNAANKILKNM
NP_063971         IPQLIARIDTPRPLVGRLIHQLLTDIGRYHPQALIYPLTVASKSTTTARHNAANKILKNM
NP_001138927      IPQLIARIDTPRPLVGRLIHQLLTDIGRYHPQALIYPLTVASKSTTTARHNAANKILKNM
NP_001272677      IPQLIARIDTPRPLVGRLIHQLLTDIGRYHPQALIYPLTVASKSTTTARHNAANKILKNM
NP_001260427      IPQLIARIDTHRQLVGQLIHQLLMDIGKNHPQALVYPLTVASKSASLARRNAAFKILDSM
NP_524891         IPQLIARIDTHRQLVGQLIHQLLMDIGKNHPQALVYPLTVASKSASLARRNAAFKILDSM
AJV38810          LPQLISRIHQPDPTVSNSLLSLLSDLGKAHPQALVYPLTVAIKSESVSRQKAALSIIEKI
AJS55673          LPQLISRIHQPNQIVSRSLLSLLSDLGKAHPQALVYPLMVAIKSESLSRQKAALSIIEKM
                  :****:**.     *.. : .** *:*: *****:*** ** ** : :*::** .*:..:

                               Rapamycin binding domain
NP_004949         CEHSNTLVQQAMMVSEELIRVAILWHEMWHEGLEEASRLYFGERNVKGMFEVLEPLHAMM
NP_064393         CEHSNTLVQQAMMVSEELIRVAILWHEMWHEGLEEASRLYFGERNVKGMFEVLEPLHAMM
NP_063971         CEHSNTLVQQAMMVSEELIRVAILWHEMWHEGLEEASRLYFGERNVKGMFEVLEPLHAMM
NP_001138927      CEHSNTLVQQAMMVSEELIRVAILWHEMWHEGLEEASRLYFGERNVKGMFEVLEPLHAMM
NP_001272677      CEHSNTLVQQAMMVSEELIRVAILWHEMWHEGLEEASRLYFGERNVKGMFEVLEPLHAMM
NP_001260427      RKHSPTLVEQAVMCSEELIRVAILWHEQWHEGLEEASRLYFGDRNVKGMFEILEPLHAML
NP_524891         RKHSPTLVEQAVMCSEELIRVAILWHEQWHEGLEEASRLYFGDRNVKGMFEILEPLHAML
AJV38810          RIHSPVLVNQAELVSHELIRVAVLWHELWYEGLEDASRQFFVEHNIEKMFSTLEPLHKHL
AJS55673          RIHSPVLVDQAELVSHELIRMAVLWHEQWYEGLDDASRQFFGEHNTEKMFAALEPLYEML
                    ** .**:** : *.****:*:**** *:***::*** :* ::* : **  ****:  :

NP_004949         ERGPQTLKETSFNQAYGRDLMEAQEWCRKYMKSGNVKDLTQAWDLYYHVFRRISKQLPQL
NP_064393         ERGPQTLKETSFNQAYGRDLMEAQEWCRKYMKSGNVKDLTQAWDLYYHVFRRISKQLPQL
NP_063971         ERGPQTLKETSFNQAYGRDLMEAQEWCRKYMKSGNVKDLTQAWDLYYHVFRRISKQLPQL
NP_001138927      ERGPQTLKETSFNQAYGRDLMEAQEWCRKYMKSGNVKDLTQAWDLYYHVFRRISKQLPQL
NP_001272677      ERGPQTLKETSFNQAYGRDLMEAQEWCRKYMKSGNVKDLTQAWDLYYHVFRRISKQLPQL
NP_001260427      ERGPQTLKETSFSQAYGRELTEAYEWSQRYKTSAVVMDLDRAWDIYYHVFQKISRQLPQL
NP_524891         ERGPQTLKETSFSQAYGRELTEAYEWSQRYKTSAVVMDLDRAWDIYYHVFQKISRQLPQL
AJV38810          GNEPQTLSEVSFQKSFGRDLNDAYEWLNNYKKSKDINNLNQAWDIYYNVFRKITRQIPQL
AJS55673          KRGPETLREISFQNSFGRDLNDAYEWLMNYKKSKDVSNLNQAWDIYYNVFRKIGKQLPQL
                   . *:** * **.:::**:* :* **  .* .*  :  :* :***:**:**::* :*:***

                                                          PI3-Kinase
NP_004949         TSLELQYVSPKLLMCRDLELAVPGTYDPN-QPIIRIQSIAPSLQVITSKQRPRKLTLMGS
NP_064393         TSLELQYVSPKLLMCRDLELAVPGTYDPN-QPIIRIQSIAPSLQVITSKQRPRKLTLMGS
NP_063971         TSLELQYVSPKLLMCRDLELAVPGTYDPN-QPIIRIQSIAPSLQVITSKQRPRKLTLMGS
NP_001138927      TSLELQYVSPKLLMCRDLELAVPGTYDPN-QPIIRIQSIAPSLQVITSKQRPRKLTLMGS
NP_001272677      TSLELQYVSPKLLMCRDLELAVPGTYDPN-QPIIRIQSIAPSLQVITSKQRPRKLTLMGS
NP_001260427      TSLELPYVSPKLMTCKDLELAVPGSYNPG-QELIRISIIKTNLQVITSKQRPRKLCIRGS
NP_524891         TSLELPYVSPKLMTCKDLELAVPGSYNPG-QELIRISIIKTNLQVITSKQRPRKLCIRGS
AJV38810          QTLDLQHVSPQLLATHDLELAVPGTYFPG-KPTIRIVKFEPLFSVISSKQRPRKFSIKGS
AJS55673          QTLELQHVSPKLLSAHDLELAVPGTRASGGKPIVKISKFEPVFSVISSKQRPRKFCIKGS
                   :*:* :***:*:  :********:  .. :  ::*  : . :.**:*******: : **
```

```
NP_004949        NGHEFVFLLKGHEDLRQDERVMQLFGLVNTLLANDPTSLRKNLSIQRYAVIPLSTNSGLI
NP_064393        NGHEFVFLLKGHEDLRQDERVMQLFGLVNTLLANDPTSLRKNLSIQRYAVIPLSTNSGLI
NP_063971        NGHEFVFLLKGHEDLRQDERVMQLFGLVNTLLANDPTSLRKNLSIQRYAVIPLSTNSGLI
NP_001138927     NGHEFVFLLKGHEDLRQDERVMQLFGLVNTLLANDPTSLRKNLSIQRYAVIPLSTNSGLI
NP_001272677     NGHEFVFLLKGHEDLRQDERVMQLFGLVNTLLANDPTSLRKNLSIQRYAVIPLSTNSGLI
NP_001260427     NGKDYMYLLKGHEDLRQDERVMQLFSLVNTLLLDDPDTFRRNLAIQRYAVIPLSTNSGLI
NP_524891        NGKDYMYLLKGHEDLRQDERVMQLFSLVNTLLLDDPDTFRRNLAIQRYAVIPLSTNSGLI
AJV38810         DGKDYKYVLKGHEDIRQDSLVMQLFGLVNTLLKNDSECFKRHLDIQQYPAIPLSPKSGLL
AJS55673         DGKDYKYVLKGHEDIRQDSLVMQLFGLVNTLLQNDAECFRRHLDIQQYPAIPLSPKSGLL
                 :*::: ::******:***. *****.****** :*.  ::::* **:*..****.:***:

NP_004949        GWVPHCDTLHALIRDYREKKKILLNIEHRIMLRMAPDYDHLTLMQKVEVFEHAVNNTAGD
NP_064393        GWVPHCDTLHALIRDYREKKKILLNIEHRIMLRMAPDYDHLTLMQKVEVFEHAVNNTAGD
NP_063971        GWVPHCDTLHALIRDYREKKKILLNIEHRIMLRMAPDYDHLTLMQKVEVFEHAVNNTAGD
NP_001138927     GWVPHCDTLHALIRDYREKKKILLNIEHRIMLRMAPDYDHLTLMQKVEVFEHAVNNTAGD
NP_001272677     GWVPHCDTLHALIRDYREKKKILLNIEHRIMLRMAPDYDHLTLMQKVEVFEHAVNNTAGD
NP_001260427     GWVPHCDTLHTLIRDYRDKKKVPLNQEHRTMLNFAPDYDHLTLMQKVEVFEHALGQTQGD
NP_524891        GWVPHCDTLHTLIRDYRDKKKVPLNQEHRTMLNFAPDYDHLTLMQKVEVFEHALGQTQGD
AJV38810         GWVPNSDTFHVLIREHRDAKKIPLNIEHWVMLQMAPDYENLTLLQKIEVFTYALDNTKGQ
AJS55673         GWVPNSDTFHVLIREHREAKKIPLNIEHWVMLQMAPDYDNLTLLQKVEVFTYALNNTEGQ
                 ****:.**:*.***::*: **: ** **  **.:****::***:**:*** :*:.:* *:

NP_004949        DLAKLLWLKSPSSEVWFDRRTNYTRSLAVMSMVGYILGLGDRHPSNLMLDRLSGKILHID
NP_064393        DLAKLLWLKSPSSEVWFDRRTNYTRSLAVMSMVGYILGLGDRHPSNLMLDRLSGKILHID
NP_063971        DLAKLLWLKSPSSEVWFDRRTNYTRSLAVMSMVGYILGLGDRHPSNLMLDRLSGKILHID
NP_001138927     DLAKLLWLKSPSSEVWFDRRTNYTRSLAVMSMVGYILGLGDRHPSNLMLDRLSGKILHID
NP_001272677     DLAKLLWLKSPSSEVWFDRRTNYTRSLAVMSMVGYILGLGDRHPSNLMLDRLSGKILHID
NP_001260427     DLAKLLWLKSPSSELWFERRNNYTRSLAVMSMVGYILGLGDRHPSNLMLDRMSGKILHID
NP_524891        DLAKLLWLKSPSSELWFERRNNYTRSLAVMSMVGYILGLGDRHPSNLMLDRMSGKILHID
AJV38810         DLYKILWLKSRSSETWLERRTTYTRSLAVMSMTGYILGLGDRHPSNLMLDRITGKVIHID
AJS55673         DLYKVLWLKSRSSETWLERRTTYTRSLAVMSMTGYILGLGDRHPSNLMLDRITGKVIHID
                 ** *:***** *** *::**..**********.******************::**::***

NP_004949        FGDCFEVAMTREKFPEKIPFRLTRMLTNAMEVTGLDGNYRITCHTVMEVLREHKDSVMAV
NP_064393        FGDCFEVAMTREKFPEKIPFRLTRMLTNAMEVTGLDGNYRTTCHTVMEVLREHKDSVMAV
NP_063971        FGDCFEVAMTREKFPEKIPFRLTRMLTNAMEVTGLDGNYRTTCHTVMEVLREHKDSVMAV
NP_001138927     FGDCFEVAMTREKFPEKIPFRLTRMLTNAMEVTGLDGNYRITCHTVMEVLREHKDSVMAV
NP_001272677     FGDCFEVAMTREKFPEKIPFRLTRMLTNAMEVTGLDGNYRITCHTVMEVLREHKDSVMAV
NP_001260427     FGDCFEVAMTREKFPEKIPFRLTRMLIKAMEVTGIEGTYRRTCESVMLVLRRNKDSLMAV
NP_524891        FGDCFEVAMTREKFPEKIPFRLTRMLIKAMEVTGIEGTYRRTCESVMLVLRRNKDSLMAV
AJV38810         FGDCFEAAILREKYPEKVPFRLTRMLTYAMEVSGIEGSFRITCENVMRVLRDNKESLMAI
AJS55673         FGDCFEAAILREKFPEKVPFRLTRMLTYAMEVSGIEGSFRITCENVMKVLRDNKDSLMAI
                 ******.*: ***:***:********  ****:*::*.:* **..** *** :*:*:**:

NP_004949        LEAFVYDPLLNWRLMDTNTKGN---------------------------------KRSRTRT
NP_064393        LEAFVYDPLLNWRLMDTNTKGN---------------------------------KRSRTRT
NP_063971        LEAFVYDPLLNWRLMDTNAKGN---------------------------------KRSRTRT
NP_001138927     LEAFVYDPLLNWRLMDTNTKGN---------------------------------KRSRTRT
NP_001272677     LEAFVYDPLLNWRLMDTNTKGN---------------------------------KRSRTRT
NP_001260427     LEAFVYDPLLNWRLLDVDKKGNDAVAGAGAPGGRGGSGMQDSLSNSVEDSLPMAKSKPYD
NP_524891        LEAFVYDPLLNWRLLDVDKKGNDAVAGAGAPGGRGGSGMQDSLSNSVEDSLPMAKSKPYD
AJV38810         LEAFALDPLIHWG-FDLP-----------------------------------------PQK
AJS55673         LEAFAFDPLINWG-FDLP-----------------------------------------TKK
                 ****. ***::*  :*                                         .
```

```
NP_004949         DSYSAG---------------------------------QSVEILDGVELGEPAHKKTG-
NP_064393         DSYSAG---------------------------------QSVEILDGVELGEPAHKKAG-
NP_063971         DSYSAG---------------------------------QSVEILDGVELGEPAHKKTG-
NP_001138927      DSYSAG---------------------------------QSVEILDGVELGEPAHKKTG-
NP_001272677      DSYSAG---------------------------------QSVEILDGVELGEPAHKKTG-
NP_001260427      PTLQQGGLHNNVADETNSKASQVIKRVKCKLTGTDFQTEKSVNEQSQVELLIQQATNNEN
NP_524891         PTLQQGGLHNNVADETNSKASQVIKRVKCKLTGTDFQTEKSVNEQSQVELLIQQATNNEN
AJV38810          LTEQTG---------------------------------IPLPLINPSELLRKG------
AJS55673          IEEETG---------------------------------IQLPVMNANELLSNG------
                     . *                                   :   .  **
```

FATC domain

```
NP_004949         ------------TTVPESIHSFIGDGLVKPEALNKKAIQIINRVRDKLTGRDFSHDDTLD
NP_064393         ------------TTVPESIHSFIGDGLVKPEALNKKAIQIINRVRDKLTGRDFSHDDTLD
NP_063971         ------------TTVPESIHSFIGDGLVKPEALNKKAIQIINRVRDKLTGRDFSHDDTLD
NP_001138927      ------------TTVPESIHSFIGYGLVKPEALNKKAIQIINRVRDKLTGRDFSHDETLD
NP_001272677      ------------TTVPESIHSFIGDGLVKPEALNKKAIQIINRVRDKLTGRDFSHDETLD
NP_001260427      LCQCYIGWCPFWTTVPESIHSFIGDGLVKPEALNKKAIQIINRVRDKLTGRDFSHDETLD
NP_524891         LCQCYIGWCPFWTTVPESIHSFIGDGLVKPEALNKKAIQIINRVRDKLTGRDFSHDETLD
AJV38810          ------------AITVEEAANMEAE--QQNETRNARAMLVLRRITDKLTGNDIKRFNELD
AJS55673          ------------AITEEEVQRVENE--HKNAIRNARAMLVLKRITDKLTGNDIRRFNDLD
                              : . *.   .      :    * :*: ::.*: *****.*: : : **

NP_004949         VPTQVELLIKQATSHENLCQCYIGWCPFW
NP_064393         VPTQVELLIKQATSHENLCQCYIGWCPFW
NP_063971         VPTQVELLIKQATSHENLCQCYIGWCPFW
NP_001138927      VPTQVELLIKQATSHENLCQCYIGWCPFW
NP_001272677      VPTQVELLIKQATSHENLCQCYLGWCPFW
NP_001260427      VPTQVELLIKQATSHENLCQCYLGWCPFW
NP_524891         VPTQVELLIKQATSHENLCQCYLGWCPFW
AJV38810          VPEQVDKLIQQATSIERLCQHYIGWCPFW
AJS55673          VPEQVDKLIQQATSVENLCQHYIGW----
                  ** **: **:**** *.*** *:**
```

Attention: RBD was indicated in deep yellow and in red vertical brackets; amino acid residues having contact (d ≤ 4.5 Å) with rapamycin are in red whereas those that contact hFKBP12a are in rose; sequences of these two segments are in black and were placed between green parantheses.

Figure S3A. Sequence attributes of fragments of sequences homologous to human mTOR (Figure S3).

```
--------------------------------------------------------------------------------------------------------------------
  1 NP_004949     2549AAs |1880-2549/Alg = 670AA || pI 7.8 ( 6.7) || HI  40.9 ( 43.2) || Mass kDa 76.9 ( 288.9) ||
>  serine/threonine-protein kinase mTOR [Homo sapiens].
--------------------------------------------------------------------------------------------------------------------
  2 NP_064393     2549AAs |1880-2549/Alg = 670AA || pI 7.8 ( 6.7) || HI  40.6 ( 43.4) || Mass kDa 76.9 ( 288.8) ||
>  serine/threonine-protein kinase mTOR [Mus musculus].
--------------------------------------------------------------------------------------------------------------------
  3 NP_063971     2549AAs |1880-2549/Alg = 670AA || pI 7.8 ( 6.7) || HI  40.6 ( 43.2) || Mass kDa 76.9 ( 288.8) ||
>  serine/threonine-protein kinase mTOR [Rattus norvegicus].
--------------------------------------------------------------------------------------------------------------------
  4 NP_001138927  2550AAs |1880-2550/Alg = 671AA || pI 8.0 ( 6.8) || HI  41.3 ( 43.5) || Mass kDa 77.1 ( 289.0) ||
>  serine/threonine-protein kinase mTOR [Ovis aries].
--------------------------------------------------------------------------------------------------------------------
  5 NP_001272677  2549AAs |1880-2549/Alg = 670AA || pI 7.8 ( 6.8) || HI  40.9 ( 43.1) || Mass kDa 76.9 ( 288.6) ||
>  serine/threonine-protein kinase mTOR [Capra hircus].
--------------------------------------------------------------------------------------------------------------------
  6 NP_001260427  2471AAs |1805-2549/Alg = 745AA || pI 7.3 ( 6.5) || HI  37.7 ( 41.4) || Mass kDa 85.1 ( 281.2) ||
>  target of rapamycin, isoform B [Drosophila melanogaster].
--------------------------------------------------------------------------------------------------------------------
  7 NP_524891     2470AAs |1800-2549/Alg = 750AA || pI 7.5 ( 6.5) || HI  37.9 ( 41.5) || Mass kDa 85.7 ( 281.0) ||
>  target of rapamycin, isoform A [Drosophila melanogaster].
--------------------------------------------------------------------------------------------------------------------
  8 AJV38810      2470AAs |1820-2470/Alg = 651AA || pI 6.8 ( 6.9) || HI  41.9 ( 44.5) || Mass kDa 75.0 ( 281.1) ||
>  Tor1p [Saccharomyces cerevisiae YJM1273].
--------------------------------------------------------------------------------------------------------------------
  9 AJS55673      2474AAs |1820-2470/Alg = 651AA || pI 6.5 ( 7.1) || HI  42.1 ( 44.7) || Mass kDa 74.7 ( 281.6) ||
>  Tor2p [Saccharomyces cerevisiae YJM1549].
```

Figure S4. Alignment of human sequences of ATR/ATM/mTOR (ATM=Telomere-length maintenance)

```
NP_001175       --------------------MGEHGLELASMIPALRELGSATPEEYNTVVQKPRQILCQFI
NP_000042       MSLVLNDLLICCRQLEHDRATERKKEVEKFKRLIRDPETIKHLDRHSDSKQGKYLNWDAV
NP_004949       -------------------------------MLGTGPAAATTAATTSSNVSVLQQFASGLK
                                                      : .     :  .  : :

NP_001175       DRILTDVNVVAVELVKKTDSQPTSVMLLDFIQHIMKSS--PLMFVNVSGSHEAKGSCIEF
NP_000042       FRFLQKYIQKETECLRIAKPNVSASTQASRQKKMQEISSLVKYFIKCANRRAPRLKCQEL
NP_004949       SRNEETRAKAAKELQHYVTMELREMSQEESTRFYDQLNHHIFELVSSSDANERKGGILAI
                 *          *  : .  :       .  :   : .     ::. :. .  :     :

NP_001175       SNWIITRLLR-----IAATPSCHLLHKKICEVICSLLFLFKSKSPAIFGVLTKELLQLFE
NP_000042       LNYIMDTVKDSSNGAIYGADCSNILLKDILSVRKYWCEISQQQWLELFSVYFRLYLKPSQ
NP_004949       ASLIGVEGGN-------------------------------ATRIGRFANYLRNLLPSND
                 . *                                           *.   :  *   :

NP_001175       DLVYLHRRNVMGHAVEWPVVMSR-FLSQLDEHMGYLQSAPLQLMSMQNLEFIEVTLLMVL
NP_000042       DVHRVLVARIIHAVTKGCCSQTDGLNSKFLDFFSKAIQCARQEKSSSGLNHILAALTIFL
NP_004949       PVVMEMASKAIG----RLAMAGDTFTAEYVEFEVKRALEWLGADRNEGRRHAAVLVLREL
                 :      . :             : ::  :.              .. ..  . :   *

NP_001175       TRIIAIVFFRRQELLLWQIGCVLLEYGSPKIKSLAISFLTELFQLGGLPAQP--------
NP_000042       KTLAVNFRIRVCELGDEILPTLLYIWTQHRLNDSLKEVIIELFQLQIYIHHPKGAKTQEK
NP_004949       AISVPTFFFQQVQPFFDNIFVAVWDPKQAIREGAVAALRACLILTTQREPKE--------
                      . ::  :     :   :    .   :.    .   *:       :

NP_001175       -----------------------------------------ASTFFSSFLELLKHLVEMD
NP_000042       GAYESTKWRSILYNLYDLLVNEISHIGSRGKYSSGFRNIAVKENLIELMADICHQVFNED
NP_004949       ---------------------------------------MQKPQWYRHTFEEAEKGFDET
                                                                  :  .: .:

NP_001175       TDQLKLYEEPLSKLIKT----------LFPFEAEAYRNIEPVYLNMLLEKLCVMFEDGVL
NP_000042       TRSLEISQSYTTTQRESSDYSVPCKRKKIELGWEVIKDHLQKSQNDFDLVPWLQIATQLI
NP_004949       LAKEKGMNRDD--RIHG----------ALLILNELVRISSMEGERLREEMEEITQQQLVH
                  . :  :       .            : :  *  :       .       :     :

NP_001175       MRLKSDLLKAALCHLLQYFLKFVPAG---------------------------YESALQV
NP_000042       SKYPASLPNCELSPLLMILSQLLPQQRHGERTPYVLRCLTEVALCQDKRSNLESSQKSDL
NP_004949       DKYCKDLMGFGTKPRHITPFTSFQAVQ--------------------------PQQSNAL
                 :   .*               .                               ..   :

NP_001175       RKVYVRNICKALLDVLGIEVDAEYLLGPLYAALKMESMEIIEEIQCQTQQENLSSNSDGI
NP_000042       LKLWNKIWCITFRGISSEQIQAEN-FGLLGAIIQGSLVEVDREFWKLFTGSACRPSCPAV
NP_004949       VGLLGYSSHQGLMGFGTSPSPAKSTLVESRCCRDLMEEKFDQVCQWVLKCRNSKNSLIQM
                  :        : ..      *:  :    .  .    :. .             .   :

NP_001175       SPKRRRLSSSLNPSKRAP----KQTEEIKHVDMNQKSILWSALKQKAESLQISLEYSGLK
NP_000042       CCLTLALTTSIVPGTVKMGIEQNMCEVNRSFSLKESIMKWLLFYQLEGDLENSTEVP---
NP_004949       TILNLLPRLAAFRPSAFTDTQYLQDTMNHVLSCVKKEKERTAAFQALGLLSVAVRSEFK-
                         :    .             : ..  :.        *    *. : .

NP_001175       NPVIEMLEGIAVVLQLTALCTVHCSHQNMNCRTFKDCQHKSKKKP----------SVVIT
NP_000042       PILHSNFPHLVLEKILVSLTMKNCKAAMNFFQSVPECEHHQKDKEELSFSEVEELFLQTT
NP_004949       VYLPRVLDIIRAALPPKDFAHKRQKAMQVDATVFTCISMLARAMG-----------PGIQ
                  :   :  :        :   . .        .   .   :

NP_001175       WMSLDFYTKVLKS----CRSLLESVQKLDLEATIDKVVKIYDALIYMQVNSSFEDHILED
NP_000042       FDKMDFLTIVRECGIEKHQSSIGFSVHQNLKESLDRCLLGLSEQLLNNYSSEITNSETLV
NP_004949       QDIKELLEPMLAVG----LSPALTAVLYDLSRQIPQLKKDIQDGLLKMLSLVLMHKPLRH
                    ::   :         *        :*.  : :     .  :    .  : .
```

```
NP_001175       LCGMLSLPWIYSHSDDGCLKLTTFAAN-LLTLSCRISDSYSPQAQSRCVFLLTLFPRRIF
NP_000042       RCSRLLVGVLGCYCYMGVIAEEEAYKS-ELFQKAKSLMQCAGESITLFKNKTNEEFRIGS
NP_004949       PG--MPKGLAHQLASPGLTTLPEASDVGSITLALRTLGSFEFEGHSLTQFVRHCADHFLN
                    :        .  *            :    :   .   :. :          :

NP_001175       LEWRTAVYNWALQSSHEVIRASCVSGFFILLQQQNSCNRVPKIL---------------I
NP_000042       LRNMMQLCTRCLSNCTKKSPNKIASGFFLRLLTSKLMNDIADICKSLASFIKKPFDRGEV
NP_004949       SEHKEIRMEAARTCSRLLTPSIHLISGHAHVVSQTAVQVVADVLS------------KLL
                 .        .   .          . .  :  ..  : :..:                :

NP_001175       DKVKDDSD--IVKKEFASILGQLVCTLHGMFYLTSSLTEPFSEHGHVDLFCRN-------
NP_000042       ESMEDDTNGNLMEVEDQSSMNLFNDYPDSSVSDANEPGESQSTIGAINPLAEEYLSKQDL
NP_004949       VVGITDPDPDIRYCVLASLDERFDAHLAQAENLQALFVALNDQVFEIRELAICTVG----
                     *.:  :      *    :                  .    :  :.

NP_001175       ----------LKATSQHECSSSQLKASVCKPFLFLLKKKIPSPVKLAFIDNLHHLCKHLD
NP_000042       LFLDMLKFLCLCVTTAQTNTVSFRAADIRRKLLMLIDSSTLEPTKSLHLHMYLMLLKELP
NP_004949       -----------RLSSMNPAFVMPFLRKMLIQILTELEHSGIGRIKEQSARMLGHLVSNAP
                             :: :         .:   :*  :. .     *         * ..

NP_001175       ------------------------FREDETDVKAVLGTLLNLMEDPDKD-----------
NP_000042       GEEYPLPMEDVLELLKPLSNVCSLYRRDQDVCKTILNHVLHVVKNLGQSNMDSENTRDAQ
NP_004949       -------------------------RLIRPYMEPILKALILKLKDPDPDPNPG-------
                                         *  .   :.:*  ::  ::: . .

NP_001175       ----------------------VRVAFSGNIKHILES----------LDSEDGFIKELFV
NP_000042       GQFLTVIGAFWHLTKERKYIFSVRMALVNCLKTLLEADPYSKWAILNVMGKDFPVNEVFT
NP_004949       ----------------------VINNVLATIGELAQVSG---------LEMRKWVDELFI
                                      *   .   :  : :                  :.*:*

NP_001175       LRMKEAYTHAQISRNNELKDTLILTTGDIGRAAKGDLVPFALLHLLHCLLSKSASVSGAA
NP_000042       QFLADNHHQVRMLAAESINRLFQDTKGDSSRLLKALPLKLQQTAFENAYLKAQEGMREMS
NP_004949       IIMDMLQDSSLLAKRQVALWTLGQLVASTGYVVEPYRKYPTLLEVLLNFLKTEQNQGTRR
                  :        :   :     :    .. .   :          .    *. . .

NP_001175       YTEIR--------------------ALVAAKSVKLQSFFSQYKKP---------ICQFLV
NP_000042       HSAENPETLDEIYNRKSVLLTLIAVVLSCSPICEKQALFALCKSVKENGLEPHLVKKVLE
NP_004949       EAIRVLG-----------------------------------------------LLGALD
                 :                                                    :   *

NP_001175       ESLHSSQMTALPNTPCQNADVRKQDVAHQREMALNTLSEIANVFDFPDLNRFLTRTLQVL
NP_000042       KVSETFGYRRLEDFMASHLDYLVLEWLNLQDTEYNLSSFPFILLNYTNIEDFYRSCYKVL
NP_004949       PYKHKVNIGMIDQSRDASAVSLSESKSSQDSSDYSTSEMLVNMGNLPLDEFYPAVSMVAL
                   ..     : :           .     .   .  .    : : .  : :      .*

NP_001175       LPDLAAKASPAASALIRTLGKQLNVNRREILINNFKYIFSHLVCSCSKDELERALHYLKN
NP_000042       IPHLVIRS---HFDEVKSIANQIQEDWKSLLTDCFPKILVNILPYFAYEGTRDSGMAQQR
NP_004949       MRIFRDQSLSHHHTMVVQAITFIFKSLGLKCVQFLPQVMPTFLNVIR--VCDGAIREFLF
                :  :  ::       :    . :  .      : :  ::  ::          :

NP_001175       ETEIELGSLLR--------------QDFQGLHNELLLRIGEHYQQVFNGLSILASFASSD
NP_000042       ETATKVYDMLKSENLLGKQIDHLFISNLPEIVVELLMTLHEPANSSASQSTDLCDFSGDL
NP_004949       QQLGMLVSFVK--------------SHIRPYMDEIVTLMREFWVMNTSIQSTIILLIEQI
                :    : .:::              ..:     *::  : *      .  : :  :  .

NP_001175       DPYQGPRDIISPELMADYLQP------KLLGILAFFN---------MQLLSSSVGIEDKK
NP_000042       DPAPNPPHFPSHVIKATFAYISNCHKTKLKSILEILSKSPDSYQKILLAICEQAAETNNV
NP_004949       VVALGGEFKLYLPQLIPHMLR-----------------------------VFMHDNSPGR
                    .            .
```

```
NP_001175          MALNSLMSLMKLMGP---KHVSSVR--------VKMMTTLRTGLRFKDDFP---------
NP_000042          YKKHRILKIYHLFVS---LLLKDIKSGLGGAWAFVLRDVIYTLIHYINQRPSCIMDVSLR
NP_004949          IVSIKLLAAIQLFGANLDDYLH-----------LLLPPIVKLFDAPEAPLPSR-------
                        ::   :*: .     :            . :   :          *

NP_001175          --ELCCRAWDCFVRCLDHACLGSLLSHVIVALLPLIHIQ------PKETAAIFHYLIIEN
NP_000042          SFSLCCDLLSQVCQTAVTYCKDALENHLHVIVGTLIPLVYEQVEVQKQVLDLLKYLVIDN
NP_004949          --KAALETVDRLTESLDFTDYASRIIHPIVRTLDQSPELR------STAMDTLSSLVFQL
                     . .    . . .        :   *  *                . .   :  *:::

NP_001175          RD--AVQDFLHEIYFLPDHPELKKIKAVLQEYRKETSESTDLQTTLQLSMKAIQHENVDV
NP_000042          KDNENLYITIKLLDPFPDHVVFKDLRITQQKIKYSRGPFSLLEEINHFLSVSVYDALPLT
NP_004949          GKKYQIFIPMVNKVLVRHRINHQRYDVLICRIVKGYTLADEEEDPLIYQHRMLRSGQGDA
                    .   :   :     . .:   :       .           :         :      .

NP_001175          RIHALTSLK--ETLYKNQEKLIKYATDS--ETVEPIISQLVTVLLKGCQDAN-----SQA
NP_000042          RLEGLKDLR--RQLELHKDQMVDIMRASQDNPQDGIMVKLVVNLLQLSKMAINHTGEKEV
NP_004949          LASGPVETGPMKKLHVSTINLQKAWGAARRVSKDDWLEWLRRLSLELLKDSS--------
                      .  .    . *     :: .    :   . :  :  *    *:  : :

NP_001175          RLLCGECLGELGAIDPGRLDFSTTETQGKDFTFVTGVEDSSFAYGLLMELTRAYLAYADN
NP_000042          LEAVGSCLGEVGPIDFSTIAIQHSKDASYTKALKLFEDKE-------LQWTFIMLTYLNN
NP_004949          SPSLRSCWALAQAYNPMARDLFNAAFVSCWSELNEDQQDE-------------LIRSIEL
                        .* .   . :     :  :   .    :    :..              :   :

NP_001175          SRAQDSAAYAIQELLSIYDCREMETNGPGHQLWRRFPEHVREILEPHLNTR-----YKSS
NP_000042          TLVEDCVKVRSAAVTCLKNILATKTGHSFWEIYKMTTDPMLAYLQPFRTSRKKFLEVPRF
NP_004949          ALTSQDIAEVTQTLLNLAEFMEHSDKGP----LPLRDDNGIVLLGERAAKCRAYAKALHY
                   : ..:        :  : :    .   .         :     *     .

NP_001175          QKSTDWSGVKKPIYLSKLGSNFAEWSASWAGYLITKVRHDLASKIFTCCSIMMKHDFKVT
NP_000042          DKENPFEGLDDINLWIPLSENHDIWIKTLTCAFLDSG--GTKCEILQLLKPMCEVKTDFC
NP_004949          KELEFQKGPTPAILESLISINNKLQQPEAAAGVLEYA-----MKHFGELEIQATWYEKLH
                   .:    .*         :. *        :  .:         : :   .       ..

NP_001175          IYLLPHILVYVLLGCNQEDQQEVYAEIMAVLKHDDQHTINTQDIAS-DLCQLSTQTVFSM
NP_000042          QTVLPYLIHDILLQDTNESWRNLLSTHVQGFFTSCLRHFSQTSRST-TPANLDSESEHFF
NP_004949          EWEDALVAYDKKMDTNKDDPELMLGRMRCLEALGEWGQLHQQCCEKWTLVNDETQAKMAR
                       . :     :  .::. . : .        .    :      .    : .:::

NP_001175          LDHLTQWARHKFQALKAEKCPHSK--SNRNKVDSMVSTVDYEDYQSVTR-----FLDLIP
NP_000042          RCCLDKKSQRTMLAVVDYMRRQKRPSSGTIFNDAFWLDLNYLEVAKVAQSCAAHFTALLY
NP_004949          MAAAAAWGLGQWDSMEEYTCMIPRDTHDGAFYRAVLALH--QDLFSLAQQCIDKARDLLD
                          .     ::        :   .     :.       :  .:::        *:

NP_001175          QDTLAVASFRSKAYTRAVMHFESFITEKKQNIQEHLG---------FLQKLYAAMHEPDG
NP_000042          AEIYADKKSMDDQEKRSLAFEEGSQSTTISSLSEKSKEETGISLQDLLLEIYRSIGEPDS
NP_004949          AELTAMAG-----ESYSRAYGAMVSCHMLSELEEVIQ--------------YKLVPERRE
                    :  *         . :  .         ..:.*                 *  : *

NP_001175          VAGVSAIRKAEPSLKEQILEHESLGLLRDATACYDRAIQLEPDQIIHYHGVVKSMLGLGQ
NP_000042          LYGCGGGKMLQPITRLRTYEHEAMWGKALVTYDLETAIPSS----TRQAGIIQALQNLGL
NP_004949          IIRQIWWERLQGCQRIVEDWQKILMVRSLVVSPHEDMRTWL-----KYASLCGKSGRLAL
                   :      .  :   :     :: :     ..   :           :  .:      *.

                                                      FAT-domain
                                          <----------------------------
NP_001175          LSTVITQVNGVHANRSEWTDELNTYRVEAAWKLSQWDLVENYLAADGKSTTWSVRLGQLL
NP_000042          CHILSVYLKGLDYENKDWCPELEELHYQAAWRNMQWDHCT-SVSKEVEGTSYHESLYNAL
NP_004949          AHKTLVLLLGVDPSR-QLDHPLPTVHPQVTYAYMKN------MWKSARKIDAFQHMQHFV
                        . : *:. .. :    *   : :.::   :       :  . .       : : :
```

```
NP_001175       LSAKKRDITAFYDSLKLVRAEQIVPLSAASFERGSYQRGYEYIVRLHMLCELEHSIKPLF
NP_000042       QSLRDREFSTFYESLKYARVKEVEEMCKRSLES--VYSLYPTLSRLQAIGELESIGELFS
NP_004949       QTMQQQAQHAIATEDQQHKQELHKLMARCFLKLG------EWQLNLQGINESTIPKVLQY
                 : :.:   ::  . :  : :    :.   ::            .*: : *

NP_001175       QHSPGDSSQEDSLNWVARLEMTQNS-YRAKEPILALRRALLSLNKRPDYN--------EM
NP_000042       RSVTHRQLSEVYIKWQKHSQLLKDSDFSFQEPIMALRTVILEILMEKEMDNSQRECIKDI
NP_004949       YSAATEHDRSWYKAWHAWAVMNFEAVLHYKHQNQARD---------------------EK
                   .     .    *     :  ::    :.   *                       :

NP_001175       VGECWLQSARVARKAGHHQTAYNALLNAGES-----RLAELYVERAKWLWSKGDVHQALI
NP_000042       LTKHLVELSILARTFKNTQLPERAIFQIKQYNSVSCGVSEWQLEEAQVFWAKKEQSLALS
NP_004949       KKLRHASGANITNATTAATTAATATT--TAS-------------------TEGSNSESEA
                      . : ::.       .  *                          :: .   :

NP_001175       VLQKGVELCFPENETPPEGKNMLIHGRAMLLVGRFMEETANFESNAIMKKYKDVTACLPE
NP_000042       ILKQMIKKLDASCAAN-NPSLKLTYTECLRVCGNWLAETCLENPAVIMQTYLEKAVEVAG
NP_004949       ESTENSPTPSPLQKKVTEDLSKTLLMYTVPAVQGFFRSISLSRGNNLQDTLRVLTLWFDY
                   :      .      :          :     :: . .  .   : ..    :  .

NP_001175       WEDG-----------------HFYLAKYYDKLMPMVTDNKMEKQGDLIRYIVLHFGRSLQ
NP_000042       NYDGESSDELRNGKMKAFLSLARFSDTQYQRIENYMKSSEFENKQALLKRAKEEVGLLRE
NP_004949       GHWP----------------------DVNEALVEGVKAIQIDTWLQVIPQLIARIDTPRP
                                             : :   :.  :::.   ::     ...

NP_001175       YGNQFIYQSMPRMLTLWLDYGTKAYEWE-KAGRSDRVQMR--------------------
NP_000042       HKIQTNRYTVKVQRELELDELALRALKE-DRKRFLCKAVENYINCLLSGEEHDMWVFRLC
NP_004949       LVGRLIHQLLTDIGRYHPQALIYPLTVASKSTTTARHN----------------------
                   :     :        :          .

                                             TEL1 PI3-family
NP_001175       ------NDLGKINKVITEHTNYLAPYQFLTAFSQLISRICHSHD---EVFVVLMEIIAKV
NP_000042       SLWLENSGVSEVNGMMKRDGMKIPTYKFLPLMYQLAARMGTKMMGGLGFHEVLNNLISRI
NP_004949       -------AANKILKNMCEHSNTLVQQAMMVSEELIRVAILWHEMWHEGLEEASRLYFGER
                         .::   : ..   :    ::     :   :         .  .    :..

NP_001175       FLAYPQQAMWMMTAVSKS------SYPMRVNRCKEILNKAIHMKKSLEKFVGDATRLTDK
NP_000042       SMDHPHHTLFIILALANANRDEFLTKPEVARRSRITKNVPKQSSQLDEDRTEAANRIICT
NP_004949       NVKGMFEVLEPLHAMMER--------------------GPQTLKETSFNQAYGRDLMEAQ
                 :    ..:  : *: :                      .   .:   . .     :

NP_001175       LLELCNKPVDGSSSTLSMSTHFKMLKKLVEEATFSEILIPLQS---VMIPTLPSILGTHA
NP_000042       IRSRRPQMVRSVEALCDAYIILANLDATQWKTQRKGINIPADQPITKLKNLEDVVVPTME
NP_004949       EWCRKYMKSGNVKDLTQAWDLYYHVFRRISKQLPQLTSLELQYVSPKLLMCRDLELAVPG
                          . .   .       :     :   .   :  :     :       : .

                                        Kinase domain
NP_001175       NHASHEPFPGHWAYIAGFDDMVEILASLQKPKKISLKGSDGKFYIMMCKPKDDLRKDCRL
NP_000042       IKVDHTGEYGNLVTIQSFKAEFRLAGGVNLPKIIDCVGSDGKERRQLVKGRDDLRQDAVM
NP_004949       TYDPNQ----PIIRIQSIAPSLQVITSKQRPRKLTLMGSNGHEFVFLLKGHEDLRQDERV
                    :         * .:   ..:  . : *: :   **:*:    : * ::***:*  :

NP_001175       MEFNSLINKCLRKDAESRRRELHIRTYAVIPLNDECGIIEWVNNTAGLRPILTKLYK---
NP_000042       QQVFQMCNTLLQRNTETRKRKLTICTYKVVPLSQRSGVLEWCTGTVPIGEFLVNNEDGAH
NP_004949       MQLFGLVNTLLANDPTSLRKNLSIQRYAVIPLSTNSGLIGWVPHCDTLHALIRDYRE---
                 :.  : *. * .:. : :::* *  * *:**. ..*:: *      :  :: .  .

NP_001175       ---EKGVYMTGKELRQCMLPKSAALSEKLKVFREFLLPRHPPIFHEWFLRTFPDPTSWYS
NP_000042       KRYRPNDFSAFQCQKKMMEVQKKSFEEKYEVFMDVCQN-FQPVFRYFCMEKFLDPAIWFE
NP_004949       KKKILLNIEHRIMLRMAPDYDHLTLMQKVEVFEHAVNNTAGDDLAKLLWLKSPSSEVWFD
                              :     .  :: :* :** .         :      .  ..  *:.
```

```
                           Catalytic loop        A-loop
NP_001175          SRSAYCRSTAVMSMVGYILGLGDRHGENILFDSLTGECVHVDFNCLFNKGE-TFEVPEIV
NP_000042          KRLAYTRSVATSSIVGYILGLGDRHVQNILINEQSAELVHIDLGVAFEQGK-ILPTPETV
NP_004949          RRTNYTRSLAVMSMVGYILGLGDRHPSNLMLDRLSGKILHIDFGDCFEVAMTREKFPEKI
                    *  * ** *. *:*********** .*::::  :.: :*:*:.  *: .      ** :

NP_001175          PFRLTHNMVNGMGPMGTEGLFRRACEVTMRLMRDQREPLMSVLKTFLHDPLVEWSKP---
NP_000042          PFRLTRDIVDGMGITGVEGVFRRCCEKTMEVMRNSQETLLTIVEVLLYDPLFDWTMNPL-
NP_004949          PFRLTRMLTNAMEVTGLDGNYRITCHTVMEVLREHKDSVMAVLEAFVYDPLLNWRLMDTN
                   *****: :.:.*   * :* :*  *. .*.::*: ::.::::::.:::***.:*

NP_001175          --------------VKGHSKAPLNETGEVVNEKAKTHVLDIEQRLQGVIK-----------
NP_000042          -------KALYLQQRPEDETELHPTLNADDQECKRNLSDIDQSFNKVAER----------
NP_004949          TKGNKRSRTRTDSYSAGQSVEILDGVELGEPAHKKTGTTVPESIHSFIGDGLVKPEALNK
                                    ... :    :  :   *     : : :: .

NP_001175          ---------TRNRVTG------LPLSIEGHVHYLIQEATDENLLCQMYLGWTPYM
NP_000042          VLMRLQEKLKGVEEG------TVLSVGGQVNLLIQQAIDPKNLSRLFPGWKAWV
NP_004949          KAIQIINRVRDKLTGRDFSHDDTLDVPTQVELLIKQATSHENLCQCYIGWCPFW
                            :.   *        *.:  :*. **::* . : *.: : ** .:
                                                 FATC
```

ATR = NP_001175 (2644 AAs);
ATM = NP_000042 (3056 AAs);
mTOR = NP_004949 (2549 AAs);

FAT domain is in deep red whereas C-terminal FATC in orange;
Blue line - Rapamycin-binding domain (RBD) in mTOR
Sequence in green – kinase domain with catalytic loop (red)
and A-loop (rose); Sequence similarity scores (IDs) for the entire sequences vary from 12 to 14 %; the IDs for RBD + Kinase + FATC vary from 22 to 25 %.

```
MTORXray           RIQSIAPSLQVITSKQRPRKLTLMGSNGHEFVFLLKGHEDLRQDERVMQLFGLVNTLLAN
S6KINas            KIRPECFELLRVLGKGGYGKVFQVRKVTGANTGKIFAMKVLKKAMIVRNAKDTAHTKAER
                   :*:. . .*  : .*    *:  : .     .  : . : *::   * :  . .:*   .

MTORXray           DPTSLRKN--LSIQRYAVIPLSTNSGLIGWVPHCDTLHALIRDYREKKKILLNIEHRIML
S6KINas            NILEEVKHPFIVDLIYAFQTGGKLYLILEYLSGGELFMQLEREGIFMEDTACFYLAEISM
                   :  .  *:  :    **. .. :: ::.  : :  * *:    :.       .* :

MTORXray           RMAPDYDHLTLMQKVEVFEHAVNNTAGDDLAKLLWLKSPSSE---VWFDRRTNYTRSLAV
S6KINas            ALGHLHQKGIIYRDLKPENIMLNHQGHVKLTDFGLCKESIHDGTVTHTFCGTIEYMAPEI
                    :.  :::  : :.::  :*: .  .*:.:   *..  :   .     *    :  :

MTORXray           MSMVGYILGLGDRHPSNLMLDRLS------GKILHIDFGDCFEVAMTREKFPEKIPFRLT
S6KINas            LMRSGHNRAVDWWSLGALMYDMLTGAPPFTGENRKKTIDKILKCKLNLPPYLTQEARDLL
                   :   *:  .:.    . ** * *:      *:  :  :.. ::  :.   :  : .  *

MTORXray           RMLTNAMEVTGLDGNYRITCHTVMEVLREHK--DSVMAVLEAFVYDPLLNWRL
S6KINas            KKLLKRNAASRLGAGPGDAGEVQAHPFFRHINWEELLARKVEPPFKPLLQS--
                   : * :   .: *...   : ..  . : .*   :.::*      :.***:
```

Table S1. Sequence attributes of several proteins that form mTORC1 and mTORC2.

```
--------------------------------------------------------------------------------------------------------------------
  1 NP_065812      1335AAs |   1-1335/Alg =1335AA || pI 6.4 ( 6.4) || HI  43.1 ( 43.1) || Mass kDa149.0 ( 149.0) ||
>  regulatory-associated protein of mTOR isoform 1 [Homo sapiens].
--------------------------------------------------------------------------------------------------------------------
  2 NP_001272368  1732AAs |   1-1732/Alg =1732AA || pI 7.5 ( 7.5) || HI  40.3 ( 40.3) || Mass kDa195.0 ( 195.0) ||
>  rapamycin-insensitive companion of mTOR isoform 2 [Homo sapiens].
--------------------------------------------------------------------------------------------------------------------
  3 NP_001272369  1385AAs |   1-1385/Alg =1385AA || pI 6.7 ( 6.7) || HI  39.1 ( 39.1) || Mass kDa155.9 ( 155.9) ||
>  rapamycin-insensitive companion of mTOR isoform 3 [Homo sapiens].
--------------------------------------------------------------------------------------------------------------------
  4 NP_689969      1708AAs |   1-1708/Alg =1708AA || pI 7.1 ( 7.1) || HI  40.4 ( 40.4) || Mass kDa192.2 ( 192.2) ||
>  rapamycin-insensitive companion of mTOR isoform 1 [Homo sapiens].
--------------------------------------------------------------------------------------------------------------------
  5 NP_071767       326AAs |   1- 326/Alg = 326AA || pI 5.4 ( 5.4) || HI  34.7 ( 34.7) || Mass kDa 35.9 (  35.9) ||
>  target of rapamycin complex subunit LST8 isoform a [Homo sapiens].
--------------------------------------------------------------------------------------------------------------------
  6 NP_001269941    308AAs |   1- 308/Alg = 308AA || pI 7.2 ( 7.2) || HI  35.7 ( 35.7) || Mass kDa 34.0 (  34.0) ||
>  DEP domain-containing mTOR-interacting protein isoform 2 [Homo
--------------------------------------------------------------------------------------------------------------------
  7 NP_079117       368AAs |   1- 368/Alg = 368AA || pI 6.3 ( 6.3) || HI  42.7 ( 42.7) || Mass kDa 40.8 (  40.8) ||
>  proline-rich protein 5-like isoform a [Homo sapiens].
--------------------------------------------------------------------------------------------------------------------
  8 NP_073620       409AAs |   1- 409/Alg = 409AA || pI 8.0 ( 8.0) || HI  31.5 ( 31.5) || Mass kDa 46.3 (  46.3) ||
>  DEP domain-containing mTOR-interacting protein isoform 1 [Homo
--------------------------------------------------------------------------------------------------------------------
  9 P42345         2549AAs |   1-2549/Alg =2549AA || pI 6.7 ( 6.7) || HI  43.2 ( 43.2) || Mass kDa288.9 ( 288.9) ||
>  RecName: Full=Serine/threonine-protein kinase mTOR; AltName:
--------------------------------------------------------------------------------------------------------------------
 10 P23443          525AAs |   1- 525/Alg = 525AA || pI 6.2 ( 6.2) || HI  26.9 ( 26.9) || Mass kDa 59.1 (  59.1) ||
>  RecName: Full=Ribosomal protein S6 kinase beta-1; Short=S6K-beta-1;
```

Table S2. Output from search(Lex_Lyser) with word =:: kinase in Human Genomic Database

#	Database-code	Length	m(kDa)	pI	HI	Chromosome	Gene	Title in database
1	NP_060370.1	199	22	11.2	24.1	1p36.33	AURKAIP1	aurora kinase A-interacting protein [Homo sapiens].
2	NP_001120701.1	199	22	11.2	24.1	1p36.33	AURKAIP1	aurora kinase A-interacting protein [Homo sapiens].
3	NP_001120702.1	199	22	11.2	24.1	1p36.33	AURKAIP1	aurora kinase A-interacting protein [Homo sapiens].
4	NP_277028.1	771	90	5.2	17.0	1p36.33	CDK11B	cyclin-dependent kinase 11B isoform 9 [Homo sapiens].
5	NP_277027.1	780	91	5.2	17.3	1p36.33	CDK11B	cyclin-dependent kinase 11B isoform 8 [Homo sapiens].
6	NP_277021.1	782	91	5.3	16.6	1p36.33	CDK11B	cyclin-dependent kinase 11B isoform 2 [Homo sapiens].
7	NP_277023.1	737	86	5.1	17.4	1p36.33	CDK11B	cyclin-dependent kinase 11B isoform 4 [Homo sapiens].
8	NP_277024.1	748	87	5.2	17.1	1p36.33	CDK11B	cyclin-dependent kinase 11B isoform 5 [Homo sapiens].
9	NP_277022.1	526	59	4.4	24.3	1p36.33	CDK11B	cyclin-dependent kinase 11B isoform 3 [Homo sapiens].
10	NP_277071.2	770	90	5.1	17.7	1p36.33	CDK11A	cyclin-dependent kinase 11A isoform 4 [Homo sapiens].
11	NP_076916.2	780	91	5.2	17.4	1p36.33	CDK11A	cyclin-dependent kinase 11A isoform 1 [Homo sapiens].
12	NP_075394.3	446	49	6.0	46.0	1p36.33	NADK	NAD kinase isoform 1 [Homo sapiens].
13	NP_001185922.1	446	49	6.0	46.0	1p36.33	NADK	NAD kinase isoform 1 [Homo sapiens].
14	NP_001185923.1	591	63	6.3	47.0	1p36.33	NADK	NAD kinase isoform 2 [Homo sapiens].
15	NP_001185924.1	414	45	5.7	45.9	1p36.33	NADK	NAD kinase isoform 3 [Homo sapiens].
16	NP_002735.3	592	68	5.4	27.5	1p36.33-p3	PRKCZ	protein kinase C zeta type isoform 1 [Homo sapiens].
17	NP_001229803.1	488	56	5.5	28.9	1p36.33-p3	PRKCZ	protein kinase C zeta type isoform 3 [Homo sapiens].
18	NP_001028753.1	409	47	4.5	31.5	1p36.33-p3	PRKCZ	protein kinase C zeta type isoform 2 [Homo sapiens].
19	NP_001028754.1	409	47	4.5	31.5	1p36.33-p3	PRKCZ	protein kinase C zeta type isoform 2 [Homo sapiens].
20	NP_060686.1	773	86	5.8	44.4	1p36.32	PANK4	pantothenate kinase 4 [Homo sapiens].
21	NP_078930.3	702	79	9.3	40.7	1p36.31	NOL9	polynucleotide 5'-hydroxyl-kinase NOL9 [Homo sapiens].
22	NP_005017.3	1044	119	6.8	39.1	1p36.2	PIK3CD	phosphatidylinositol 4,5-bisphosphate 3-kinase catalytic subunit
23	NP_004949.1	2549	289	6.7	43.2	1p36.2	MTOR	serine/threonine-protein kinase mTOR [Homo sapiens].
24	NP_004422.2	976	106	5.8	32.4	1p36	EPHA2	ephrin type-A receptor 2 precursor [Homo sapiens].
25	NP_061054.2	78	9	5.1	9.0	1p36.12	CAMK2N1	calcium/calmodulin-dependent protein kinase II inhibitor 1 [Homo
26	NP_115785.1	581	55	8.3	54.0	1p36	PINK1	serine/threonine-protein kinase PINK1, mitochondrial precursor
27	NP_065387.1	1005	108	7.7	36.8	1p36.12	EPHA8	ephrin type-A receptor 8 isoform 1 precursor [Homo sapiens].
28	NP_001006944.1	495	51	5.9	34.6	1p36.12	EPHA8	ephrin type-A receptor 8 isoform 2 precursor [Homo sapiens].
29	NP_004433.2	987	108	5.3	38.0	1p36.1-p35	EPHB2	ephrin type-B receptor 2 isoform 2 precursor [Homo sapiens].
30	NP_059145.2	986	108	5.2	38.1	1p36.1-p35	EPHB2	ephrin type-B receptor 2 isoform 1 precursor [Homo sapiens].
31	NP_076942.1	357	40	7.3	16.8	1p36.11	AUNIP	aurora kinase A and ninein-interacting protein [Homo sapiens].
32	NP_690048.1	341	39	6.4	39.0	1p36.11	PDIK1L	serine/threonine-protein kinase PDIK1L [Homo sapiens].
33	NP_001230461.1	341	39	6.4	39.0	1p36.11	PDIK1L	serine/threonine-protein kinase PDIK1L [Homo sapiens].
34	NP_001230462.1	341	39	6.4	39.0	1p36.11	PDIK1L	serine/threonine-protein kinase PDIK1L [Homo sapiens].
35	NP_006305.2	713	79	5.1	31.6	1p36.11	CNKSR1	connector enhancer of kinase suppressor of ras 1 [Homo sapiens].
36	NP_002944.2	735	83	7.7	33.5	1p	RPS6KA1	ribosomal protein S6 kinase alpha-1 isoform a [Homo sapiens].
37	NP_001006666.1	744	84	9.0	34.1	1p	RPS6KA1	ribosomal protein S6 kinase alpha-1 isoform b [Homo sapiens].
38	NP_004663.3	1288	143	6.7	41.2	1p36.11	MAP3K6	mitogen-activated protein kinase kinase kinase 6 [Homo sapiens].
39	NP_001036194.1	529	59	5.3	31.9	1p36.2-p36	FGR	tyrosine-protein kinase Fgr [Homo sapiens].
40	NP_005239.1	529	59	5.3	31.9	1p36.2-p36	FGR	tyrosine-protein kinase Fgr [Homo sapiens].
41	NP_001036212.1	529	59	5.3	31.9	1p36.2-p36	FGR	tyrosine-protein kinase Fgr [Homo sapiens].
42	NP_005347.3	509	58	5.1	31.7	1p34.3	LCK	tyrosine-protein kinase Lck precursor [Homo sapiens].
43	NP_001036236.1	509	58	5.1	31.7	1p34.3	LCK	tyrosine-protein kinase Lck precursor [Homo sapiens].
44	NP_075385.1	195	20	4.5	4.1	1p35.1	MARCKSL1	MARCKS-related protein [Homo sapiens].
45	NP_443073.1	268	30	6.3	41.0	1p35-p34	TSSK3	testis-specific serine/threonine-protein kinase 3 [Homo sapiens].
46	NP_001186128.1	224	25	6.3	35.3	1p34	AK2	adenylate kinase 2, mitochondrial isoform c [Homo sapiens].
47	NP_037543.1	232	26	8.0	36.2	1p34	AK2	adenylate kinase 2, mitochondrial isoform b [Homo sapiens].
48	NP_001616.1	239	26	7.8	36.8	1p34	AK2	adenylate kinase 2, mitochondrial isoform a [Homo sapiens].
49	NP_114406.1	435	49	7.8	33.8	1p34.3	STK40	serine/threonine-protein kinase 40 [Homo sapiens].
50	NP_005415.1	1138	123	6.5	35.5	1p34-p33	TIE1	tyrosine-protein kinase receptor Tie-1 isoform 1 precursor [Homo
51	NP_001240286.1	1093	120	6.6	34.9	1p34-p33	TIE1	tyrosine-protein kinase receptor Tie-1 isoform 2 [Homo sapiens].
52	NP_004064.2	646	72	9.4	36.8	1p34.1	PLK3	serine/threonine-protein kinase PLK3 [Homo sapiens].
53	NP_009101.2	571	64	6.6	31.3	1p32	TESK2	dual specificity testis-specific protein kinase 2 [Homo sapiens].
54	NP_055927.2	1798	196	8.1	24.7	1p34.1	MAST2	microtubule-associated serine/threonine-protein kinase 2 [Homo

55	NP_003620.3	461	54	5.6	15.2	1p34.1	PIK3R3	phosphatidylinositol 3-kinase regulatory subunit gamma [Homo
56	NP_001107644.1	461	54	5.6	15.2	1p34.1	PIK3R3	phosphatidylinositol 3-kinase regulatory subunit gamma [Homo
57	NP_001129025.1	424	47	6.4	30.7	1p33	MKNK1	MAP kinase-interacting serine/threonine-protein kinase 1 isoform 3
58	NP_003675.2	465	51	6.3	32.7	1p33	MKNK1	MAP kinase-interacting serine/threonine-protein kinase 1 isoform 1
59	NP_945324.1	347	39	6.3	30.5	1p33	MKNK1	MAP kinase-interacting serine/threonine-protein kinase 1 isoform 2
60	NP_660322.2	268	32	9.6	38.4	1p33	MOB3C	MOB kinase activator 3C isoform 1 [Homo sapiens].
61	NP_958805.1	216	26	8.9	39.8	1p33	MOB3C	MOB kinase activator 3C isoform 2 [Homo sapiens].
62	NP_057392.1	228	26	8.0	34.6	1p32	CMPK1	UMP-CMP kinase isoform a [Homo sapiens].
63	NP_001129612.1	179	20	7.6	36.3	1p32	CMPK1	UMP-CMP kinase isoform b [Homo sapiens].
64	NP_001253.1	168	18	6.1	39.3	1p32	CDKN2C	cyclin-dependent kinase 4 inhibitor C [Homo sapiens].
65	NP_523240.1	168	18	6.1	39.3	1p32	CDKN2C	cyclin-dependent kinase 4 inhibitor C [Homo sapiens].
66	NP_006243.2	552	62	7.6	33.5	1p31	PRKAA2	5'-AMP-activated protein kinase catalytic subunit alpha-2 [Homo
67	NP_001106882.1	575	63	6.0	53.6	1p32.1	FGGY	FGGY carbohydrate kinase domain-containing protein isoform a [Homo
68	NP_060761.3	551	60	5.9	53.5	1p32.1	FGGY	FGGY carbohydrate kinase domain-containing protein isoform b [Homo
69	NP_001231643.1	463	50	5.6	55.9	1p32.1	FGGY	FGGY carbohydrate kinase domain-containing protein isoform 3 [Homo
70	NP_005003.2	937	101	6.3	31.3	1p32-p31	ROR1	tyrosine-protein kinase transmembrane receptor ROR1 isoform 1
71	NP_001077061.1	393	41	5.4	27.5	1p32-p31	ROR1	tyrosine-protein kinase transmembrane receptor ROR1 isoform 2
72	NP_002218.2	1154	133	7.3	29.9	1p32.3-p31	JAK1	tyrosine-protein kinase JAK1 [Homo sapiens].
73	NP_982289.1	223	25	8.7	25.1	1p31.3	AK4	adenylate kinase isoenzyme 4, mitochondrial [Homo sapiens].
74	NP_037542.1	223	25	8.7	25.1	1p31.3	AK4	adenylate kinase isoenzyme 4, mitochondrial [Homo sapiens].
75	NP_001005353.1	223	25	8.7	25.1	1p31.3	AK4	adenylate kinase isoenzyme 4, mitochondrial [Homo sapiens].
76	NP_057062.1	835	93	6.3	42.3	1p31.1	TNNI3K	serine/threonine-protein kinase TNNI3K [Homo sapiens].
77	NP_777283.1	562	63	4.8	27.4	1p31	AK5	adenylate kinase isoenzyme 5 isoform 1 [Homo sapiens].
78	NP_036225.2	536	60	4.8	26.9	1p31	AK5	adenylate kinase isoenzyme 5 isoform 2 [Homo sapiens].
79	NP_002722.1	351	41	9.1	26.2	1p31.1	PRKACB	cAMP-dependent protein kinase catalytic subunit beta isoform 2
80	NP_997461.1	257	30	9.4	29.6	1p31.1	PRKACB	cAMP-dependent protein kinase catalytic subunit beta isoform 3
81	NP_891993.1	398	46	9.0	23.9	1p31.1	PRKACB	cAMP-dependent protein kinase catalytic subunit beta isoform 1
82	NP_001229786.1	358	41	8.8	27.4	1p31.1	PRKACB	cAMP-dependent protein kinase catalytic subunit beta isoform 4
83	NP_001229788.1	355	41	9.0	27.6	1p31.1	PRKACB	cAMP-dependent protein kinase catalytic subunit beta isoform 6
84	NP_001229787.1	339	39	9.1	27.4	1p31.1	PRKACB	cAMP-dependent protein kinase catalytic subunit beta isoform 5
85	NP_001229789.1	357	41	8.8	27.5	1p31.1	PRKACB	cAMP-dependent protein kinase catalytic subunit beta isoform 7
86	NP_001229790.1	321	37	8.3	26.8	1p31.1	PRKACB	cAMP-dependent protein kinase catalytic subunit beta isoform 8
87	NP_001229791.1	338	39	9.1	27.8	1p31.1	PRKACB	cAMP-dependent protein kinase catalytic subunit beta isoform 9
88	NP_006247.1	984	112	5.9	29.3	1p22.2	PKN2	serine/threonine-protein kinase N2 [Homo sapiens].
89	NP_078802.2	563	64	7.6	27.0	1p13.3	EPS8L3	epidermal growth factor receptor kinase substrate 8-like protein 3
90	NP_573444.2	593	67	7.9	25.6	1p13.3	EPS8L3	epidermal growth factor receptor kinase substrate 8-like protein 3
91	NP_620641.1	594	67	7.9	25.6	1p13.3	EPS8L3	epidermal growth factor receptor kinase substrate 8-like protein 3
92	NP_001136254.1	1481	163	8.0	21.9	1p12-p11.2	MAGI3	membrane-associated guanylate kinase, WW and PDZ domain-containing
93	NP_690864.2	1125	123	6.0	27.4	1p12-p11.2	MAGI3	membrane-associated guanylate kinase, WW and PDZ domain-containing
94	NP_938009.1	1210	131	8.1	39.3	1p13.2	HIPK1	homeodomain-interacting protein kinase 1 isoform 1 [Homo sapiens].
95	NP_689909.2	1075	117	7.7	41.9	1p13.2	HIPK1	homeodomain-interacting protein kinase 1 isoform 2 [Homo sapiens].
96	NP_938010.1	836	89	8.2	38.6	1p13.2	HIPK1	homeodomain-interacting protein kinase 1 isoform 3 precursor [Homo
97	NP_852003.1	816	87	8.6	38.5	1p13.2	HIPK1	homeodomain-interacting protein kinase 1 isoform 4 [Homo sapiens].
98	NP_005390.1	272	30	6.0	28.3	1q21.1	PRKAB2	5'-AMP-activated protein kinase subunit beta-2 [Homo sapiens].
99	NP_057358.2	409	46	9.1	13.7	1q21.2	PLEKHO1	pleckstrin homology domain-containing family O member 1 [Homo
100	NP_001129110.1	562	63	8.2	34.5	1q21.3	PIP5K1A	phosphatidylinositol 4-phosphate 5-kinase type-1 alpha isoform 1
101	NP_001129108.1	522	58	8.0	33.1	1q21.3	PIP5K1A	phosphatidylinositol 4-phosphate 5-kinase type-1 alpha isoform 3
102	NP_003548.1	549	61	8.4	33.9	1q21.3	PIP5K1A	phosphatidylinositol 4-phosphate 5-kinase type-1 alpha isoform 2
103	NP_001129109.1	500	56	7.3	37.4	1q21.3	PIP5K1A	phosphatidylinositol 4-phosphate 5-kinase type-1 alpha isoform 4
104	NP_002642.1	828	93	5.9	37.9	1q21	PI4KB	phosphatidylinositol 4-kinase beta isoform 1 [Homo sapiens].
105	NP_001185702.1	801	90	6.0	37.3	1q21	PI4KB	phosphatidylinositol 4-kinase beta isoform 2 [Homo sapiens].
106	NP_001185703.1	801	90	6.0	37.3	1q21	PI4KB	phosphatidylinositol 4-kinase beta isoform 2 [Homo sapiens].
107	NP_001185704.1	484	55	5.8	37.8	1q21	PI4KB	phosphatidylinositol 4-kinase beta isoform 3 [Homo sapiens].
108	NP_001817.1	79	10	9.4	17.7	1q21.2	CKS1B	cyclin-dependent kinases regulatory subunit 1 [Homo sapiens].
109	NP_872631.1	207	23	6.9	41.5	1q21-q22	EFNA4	ephrin-A4 isoform b precursor [Homo sapiens].
110	NP_872632.2	193	22	5.1	38.3	1q21-q22	EFNA4	ephrin-A4 isoform c precursor [Homo sapiens].
111	NP_005218.1	201	22	7.0	40.8	1q21-q22	EFNA4	ephrin-A4 isoform a precursor [Homo sapiens].
112	NP_004943.1	238	26	8.7	30.3	1q21-q22	EFNA3	ephrin-A3 precursor [Homo sapiens].
113	NP_004419.2	205	22	6.5	26.2	1q21-q22	EFNA1	ephrin-A1 isoform a precursor [Homo sapiens].

114	NP_872626.1	183	19	6.0	26.1	1q21-q22	EFNA1	ephrin- A1 isoform b precursor [Homo sapiens].
115	NP_003984.2	498	60	9.9	23.1	1q21	CLK2	dual specificity protein kinase CLK2 [Homo sapiens].
116	NP_000289.1	574	62	7.6	49.3	1q21	PKLR	pyruvate kinase isozymes R/L isoform 1 [Homo sapiens].
117	NP_870986.1	543	58	6.6	49.7	1q21	PKLR	pyruvate kinase isozymes R/L isoform 2 [Homo sapiens].
118	NP_001007793.1	760	84	6.3	45.1	1q21-q22	NTRK1	high affinity nerve growth factor receptor isoform 3 [Homo
119	NP_002520.2	796	87	6.2	44.2	1q21-q22	NTRK1	high affinity nerve growth factor receptor isoform 2 precursor
120	NP_001012331.1	790	87	6.2	43.9	1q21-q22	NTRK1	high affinity nerve growth factor receptor isoform 1 precursor
121	NP_001171692.1	345	39	5.2	46.1	1q23.3	UHMK1	serine/threonine-protein kinase Kist isoform 2 [Homo sapiens].
122	NP_787062.1	419	47	5.5	42.5	1q23.3	UHMK1	serine/threonine-protein kinase Kist isoform 1 [Homo sapiens].
123	NP_653225.2	344	38	5.4	45.6	1q23.3	UHMK1	serine/threonine-protein kinase Kist isoform 3 [Homo sapiens].
124	NP_006173.2	855	94	5.0	38.6	1q23.3	DDR2	discoidin domain-containing receptor 2 precursor [Homo sapiens].
125	NP_001014796.1	855	94	5.0	38.6	1q23.3	DDR2	discoidin domain-containing receptor 2 precursor [Homo sapiens].
126	NP_036606.2	261	29	6.3	36.0	1q23	UCK2	uridine-cytidine kinase 2 [Homo sapiens].
127	NP_443094.3	416	45	5.2	9.9	1q24.2	RCSD1	capZ-interacting protein [Homo sapiens].
128	NP_037462.1	376	42	6.0	42.0	1q24	NME7	nucleoside diphosphate kinase 7 isoform a [Homo sapiens].
129	NP_932076.1	340	38	6.4	43.5	1q24	NME7	nucleoside diphosphate kinase 7 isoform b [Homo sapiens].
130	NP_001161710.1	1058	116	8.2	27.8	1q25.2	ABL2	Abelson tyrosine-protein kinase 2 isoform h [Homo sapiens].
131	NP_001161708.1	1161	126	8.3	27.6	1q25.2	ABL2	Abelson tyrosine-protein kinase 2 isoform f [Homo sapiens].
132	NP_001161709.1	1079	118	7.7	27.3	1q25.2	ABL2	Abelson tyrosine-protein kinase 2 isoform g [Homo sapiens].
133	NP_009298.1	1182	128	8.0	27.2	1q25.2	ABL2	Abelson tyrosine-protein kinase 2 isoform b [Homo sapiens].
134	NP_001161711.1	1043	114	6.9	29.9	1q25.2	ABL2	Abelson tyrosine-protein kinase 2 isoform i [Homo sapiens].
135	NP_001129472.1	1064	116	6.5	29.2	1q25.2	ABL2	Abelson tyrosine-protein kinase 2 isoform d [Homo sapiens].
136	NP_005149.4	1167	127	6.8	28.9	1q25.2	ABL2	Abelson tyrosine-protein kinase 2 isoform c [Homo sapiens].
137	NP_001129473.1	542	61	6.2	29.3	1q25.2	ABL2	Abelson tyrosine-protein kinase 2 isoform e [Homo sapiens].
138	NP_598001.1	302	35	8.2	32.5	1q31.3	NEK7	serine/threonine-protein kinase Nek7 [Homo sapiens].
139	NP_060678.2	386	45	7.0	35.2	1q32.1	ETNK2	ethanolamine kinase 2 [Homo sapiens].
140	NP_002637.3	1634	185	6.9	35.7	1q32	PIK3C2B	phosphatidylinositol 4-phosphate 3-kinase C2 domain-containing
141	NP_955749.1	884	100	6.2	37.3	1q32.1	DSTYK	dual serine/threonine and tyrosine protein kinase isoform 2 [Homo
142	NP_056190.1	929	105	6.3	37.4	1q32.1	DSTYK	dual serine/threonine and tyrosine protein kinase isoform 1 [Homo
143	NP_112214.1	628	70	9.1	27.7	1q32.1	NUAK2	NUAK family SNF1-like kinase 2 [Homo sapiens].
144	NP_997668.1	504	58	9.3	29.4	1q31-q32	CDK18	cyclin-dependent kinase 18 isoform a [Homo sapiens].
145	NP_002587.2	474	54	8.8	28.7	1q31-q32	CDK18	cyclin-dependent kinase 18 isoform b [Homo sapiens].
146	NP_997667.1	474	54	8.8	28.7	1q31-q32	CDK18	cyclin-dependent kinase 18 isoform b [Homo sapiens].
147	NP_073568.2	243	27	4.8	2.1	1q32.1	NUCKS1	nuclear ubiquitous casein and cyclin-dependent kinase substrate 1
148	NP_054721.1	716	80	7.7	38.0	1q32.1	IKBKE	inhibitor of nuclear factor kappa-B kinase subunit epsilon isoform
149	NP_001180251.1	657	74	8.0	39.0	1q32.1	IKBKE	inhibitor of nuclear factor kappa-B kinase subunit epsilon isoform
150	NP_001180250.1	631	71	6.8	38.7	1q32.1	IKBKE	inhibitor of nuclear factor kappa-B kinase subunit epsilon isoform
151	NP_003573.2	588	66	9.6	29.8	1q32.1	DYRK3	dual specificity tyrosine-phosphorylation-regulated kinase 3
152	NP_001004023.1	568	64	9.5	30.6	1q32.1	DYRK3	dual specificity tyrosine-phosphorylation-regulated kinase 3
153	NP_116584.2	400	46	8.8	27.3	1q32	MAPKAPK2	MAP kinase-activated protein kinase 2 isoform 2 [Homo sapiens].
154	NP_004750.1	370	42	8.2	26.2	1q32	MAPKAPK2	MAP kinase-activated protein kinase 2 isoform 1 [Homo sapiens].
155	NP_065172.1	476	53	7.5	36.1	1q32.2	CAMK1G	calcium/calmodulin-dependent protein kinase type 1G [Homo sapiens].
156	NP_079504.2	551	64	8.5	15.8	1q32	TRAF3IP3	TRAF3-interacting JNK-activating modulator [Homo sapiens].
157	NP_001191111.1	388	45	7.2	26.8	1q32.2-q41	NEK2	serine/threonine-protein kinase Nek2 isoform 3 [Homo sapiens].
158	NP_002488.1	445	52	9.2	24.9	1q32.2-q41	NEK2	serine/threonine-protein kinase Nek2 isoform 1 [Homo sapiens].
159	NP_001191112.1	384	45	8.5	27.3	1q32.2-q41	NEK2	serine/threonine-protein kinase Nek2 isoform 2 [Homo sapiens].
160	NP_036556.2	1066	119	4.6	29.0	1q41	RPS6KC1	ribosomal protein S6 kinase delta-1 isoform a [Homo sapiens].
161	NP_001129610.1	1054	117	4.6	29.2	1q41	RPS6KC1	ribosomal protein S6 kinase delta-1 isoform b [Homo sapiens].
162	NP_061120.3	795	89	9.7	23.3	1q41	MARK1	serine/threonine-protein kinase MARK1 [Homo sapiens].
163	NP_009138.1	482	53	7.6	39.6	1q41	DUSP10	dual specificity protein phosphatase 10 isoform a [Homo sapiens].
164	NP_653330.1	140	16	9.1	37.1	1q41	DUSP10	dual specificity protein phosphatase 10 isoform b [Homo sapiens].
165	NP_653329.1	140	16	9.1	37.1	1q41	DUSP10	dual specificity protein phosphatase 10 isoform b [Homo sapiens].
166	NP_002212.3	946	102	8.4	21.7	1q42.13	ITPKB	inositol-trisphosphate 3-kinase B [Homo sapiens].
167	NP_064632.2	647	72	6.5	36.0	1q42.13	ADCK3	chaperone activity of bc1 complex-like, mitochondrial [Homo
168	NP_055641.3	1638	186	5.9	27.7	1q42.11	CDC42BPA	serine/threonine-protein kinase MRCK alpha isoform A [Homo
169	NP_003598.2	1719	196	6.0	26.6	1q42.11	CDC42BPA	serine/threonine-protein kinase MRCK alpha isoform B [Homo
170	NP_001229769.1	241	27	9.3	36.5	1q32-q41	GUK1	guanylate kinase isoform c [Homo sapiens].
171	NP_001152862.1	218	24	7.4	38.5	1q32-q41	GUK1	guanylate kinase isoform a [Homo sapiens].
172	NP_000849.1	197	22	6.1	36.5	1q32-q41	GUK1	guanylate kinase isoform b [Homo sapiens].
173	NP_001152863.1	197	22	6.1	36.5	1q32-q41	GUK1	guanylate kinase isoform b [Homo sapiens].

174	NP_001229768.1	197	22	6.1	36.5	1q32-q41	GUK1	guanylate kinase isoform b [Homo sapiens].
175	NP_115811.2	1036	114	8.8	30.9	1q42	KIAA1804	mitogen-activated protein kinase kinase kinase MLK4 [Homo sapiens].
176	NP_859029.1	465	54	6.1	28.6	1q44	AKT3	RAC-gamma serine/threonine-protein kinase isoform 2 [Homo sapiens].
177	NP_001193658.1	465	54	6.1	28.6	1q44	AKT3	RAC-gamma serine/threonine-protein kinase isoform 2 [Homo sapiens].
178	NP_005456.1	479	56	5.7	27.8	1q44	AKT3	RAC-gamma serine/threonine-protein kinase isoform 1 [Homo sapiens].
179	NP_001243407.1	366	40	7.0	44.0	2p25.2	CMPK2	UMP-CMP kinase 2, mitochondrial isoform 3 precursor [Homo sapiens].
180	NP_997198.2	449	39	5.6	37.3	2p25.2	CMPK2	UMP-CMP kinase 2, mitochondrial isoform 1 precursor [Homo sapiens].
181	NP_001243406.1	409	45	7.1	40.6	2p25.2	CMPK2	UMP-CMP kinase 2, mitochondrial isoform 2 precursor [Homo sapiens].
182	NP_065789.1	1771	197	6.2	32.4	2p24	KIDINS220	kinase D-interacting substrate of 220 kDa [Homo sapiens].
183	NP_004841.2	1388	161	5.7	18.7	2p24	ROCK2	rho-associated protein kinase 2 [Homo sapiens].
184	NP_000212.1	298	33	5.6	41.6	2p23.3	KHK	ketohexokinase isoform a [Homo sapiens].
185	NP_006479.1	298	33	5.9	39.6	2p23.3	KHK	ketohexokinase isoform b [Homo sapiens].
186	NP_001477.2	625	69	6.2	48.6	2p23	GCKR	glucokinase regulatory protein [Homo sapiens].
187	NP_004295.2	1620	175	6.6	36.0	2p23	ALK	ALK tyrosine kinase receptor precursor [Homo sapiens].
188	NP_001129124.1	510	57	8.0	27.3	2p22-p21	EIF2AK2	interferon-induced, double-stranded RNA-activated protein kinase
189	NP_001129123.1	551	62	8.5	27.4	2p22-p21	EIF2AK2	interferon-induced, double-stranded RNA-activated protein kinase
190	NP_002750.1	551	62	8.5	27.4	2p22-p21	EIF2AK2	interferon-induced, double-stranded RNA-activated protein kinase
191	NP_005804.1	890	100	6.4	35.1	2p21	PRKD3	serine/threonine-protein kinase D3 [Homo sapiens].
192	NP_001257354.1	873	99	7.7	34.6	2p22.1	MAP4K3	mitogen-activated protein kinase kinase kinase kinase 3 isoform 2
193	NP_003609.2	894	101	7.3	33.8	2p22.1	MAP4K3	mitogen-activated protein kinase kinase kinase kinase 3 isoform 1
194	NP_612379.2	493	54	8.3	37.1	2p21	PKDCC	protein kinase domain-containing protein, cytoplasmic precursor
195	NP_005391.1	737	84	6.7	33.1	2p21	PRKCE	protein kinase C epsilon type [Homo sapiens].
196	NP_001734.1	149	17	3.9	16.8	2p21	CALM2	calmodulin [Homo sapiens].
197	NP_001123952.1	508	58	9.1	26.4	2p16.1	VRK2	serine/threonine-protein kinase VRK2 isoform 1 [Homo sapiens].
198	NP_001123953.1	508	58	9.1	26.4	2p16.1	VRK2	serine/threonine-protein kinase VRK2 isoform 1 [Homo sapiens].
199	NP_006287.2	508	58	9.1	26.4	2p16.1	VRK2	serine/threonine-protein kinase VRK2 isoform 1 [Homo sapiens].
200	NP_001123955.1	396	45	9.4	26.8	2p16.1	VRK2	serine/threonine-protein kinase VRK2 isoform 3 [Homo sapiens].
201	NP_001123954.1	485	55	9.0	27.4	2p16.1	VRK2	serine/threonine-protein kinase VRK2 isoform 2 [Homo sapiens].
202	NP_055726.3	961	104	6.1	29.1	2p14	AAK1	AP2-associated protein kinase 1 [Homo sapiens].
203	NP_060691.2	216	25	6.5	34.7	2p13.1	MOB1A	MOB kinase activator 1A [Homo sapiens].
204	NP_001372.1	481	52	6.0	22.2	2p13	DOK1	docking protein 1 isoform 1 [Homo sapiens].
205	NP_001184189.1	342	37	5.1	15.2	2p13	DOK1	docking protein 1 isoform 2 [Homo sapiens].
206	NP_000180.2	917	102	5.7	41.8	2p13	HK2	hexokinase-2 [Homo sapiens].
207	NP_004827.4	1116	122	5.1	31.4	2p12	EIF2AK3	eukaryotic translation initiation factor 2-alpha kinase 3 precursor
208	NP_001070.2	619	70	7.5	31.8	2q12	ZAP70	tyrosine-protein kinase ZAP-70 isoform 1 [Homo sapiens].
209	NP_997402.1	312	36	7.5	31.7	2q12	ZAP70	tyrosine-protein kinase ZAP-70 isoform 2 [Homo sapiens].
210	NP_001229488.1	1239	142	7.1	24.6	2q11.2-q12	MAP4K4	mitogen-activated protein kinase kinase kinase kinase 4 isoform 4
211	NP_663720.1	1212	139	7.1	24.9	2q11.2-q12	MAP4K4	mitogen-activated protein kinase kinase kinase kinase 4 isoform 3
212	NP_663719.2	1273	145	7.1	24.3	2q11.2-q12	MAP4K4	mitogen-activated protein kinase kinase kinase kinase 4 isoform 2
213	NP_001229489.1	1235	141	7.1	24.6	2q11.2-q12	MAP4K4	mitogen-activated protein kinase kinase kinase kinase 4 isoform 5
214	NP_004825.3	1165	133	7.4	26.1	2q11.2-q12	MAP4K4	mitogen-activated protein kinase kinase kinase kinase 4 isoform 1
215	NP_004327.1	1085	122	6.0	31.6	2q14	BUB1	mitotic checkpoint serine/threonine-protein kinase BUB1 [Homo
216	NP_006334.2	999	108	5.4	43.3	2q14.1	MERTK	tyrosine-protein kinase Mer precursor [Homo sapiens].
217	NP_006600.3	619	70	8.0	23.7	2q14.3	MAP3K2	mitogen-activated protein kinase kinase kinase 2 [Homo sapiens].
218	NP_001018056.1	510	58	6.2	34.7	2q21.3	MAP3K19	SPS1/STE20-related protein kinase YSK4 isoform 2 [Homo sapiens].
219	NP_079328.3	1328	151	6.6	24.5	2q21.3	MAP3K19	SPS1/STE20-related protein kinase YSK4 isoform 1 [Homo sapiens].
220	NP_001104503.1	336	37	7.2	45.2	2q24.1	ACVR1C	activin receptor type-1C isoform 4 precursor [Homo sapiens].
221	NP_001104502.1	413	46	7.4	46.2	2q24.1	ACVR1C	activin receptor type-1C isoform 3 precursor [Homo sapiens].
222	NP_660302.2	493	53	7.5	45.2	2q24.1	ACVR1C	activin receptor type-1C isoform 1 precursor [Homo sapiens].
223	NP_001104501.1	443	50	7.9	43.8	2q24.1	ACVR1C	activin receptor type-1C isoform 2 [Homo sapiens].
224	NP_001096.1	509	55	7.2	37.0	2q23-q24	ACVR1	activin receptor type-1 precursor [Homo sapiens].
225	NP_001104537.1	509	55	7.2	37.0	2q23-q24	ACVR1	activin receptor type-1 precursor [Homo sapiens].
226	NP_037365.2	545	59	5.9	40.9	2q24.3	STK39	STE20/SPS1-related proline-alanine-rich protein kinase [Homo
227	NP_078898.3	847	97	7.5	47.8	2q31	FASTKD1	FAST kinase domain-containing protein 1 [Homo sapiens].
228	NP_036422.3	766	87	8.9	22.6	2q31.1	TLK1	serine/threonine-protein kinase tousled-like 1 isoform 1 [Homo
229	NP_001130026.1	718	82	8.8	23.3	2q31.1	TLK1	serine/threonine-protein kinase tousled-like 1 isoform 2 [Homo
230	NP_001130027.1	670	77	9.3	22.7	2q31.1	TLK1	serine/threonine-protein kinase tousled-like 1 isoform 3 [Homo
231	NP_002601.1	436	49	9.0	32.6	2q31.1	PDK1	pyruvate dehydrogenase kinase, isozyme 1 [Homo sapiens].
232	NP_057737.2	800	91	7.7	26.3	2q24.2	ZAK	mitogen-activated protein kinase kinase kinase MLT isoform 1 [Homo

233	NP_598407.1	455	52	5.0	34.7	2q24.2	ZAK	mitogen-activated protein kinase kinase kinase MLT isoform 2 [Homo
234	NP_003681.1	313	34	8.4	31.6	2q31.2	PRKRA	interferon-inducible double stranded RNA-dependent protein kinase
235	NP_001132989.1	302	33	8.4	34.8	2q31.2	PRKRA	interferon-inducible double stranded RNA-dependent protein kinase
236	NP_001132990.1	288	32	8.4	32.3	2q31.2	PRKRA	interferon-inducible double stranded RNA-dependent protein kinase
237	NP_543152.1	217	24	6.1	40.6	2q32.1	DUSP19	dual specificity protein phosphatase 19 isoform 1 [Homo sapiens].
238	NP_001135786.1	166	18	8.1	33.1	2q32.1	DUSP19	dual specificity protein phosphatase 19 isoform 2 [Homo sapiens].
239	NP_004217.1	372	42	5.0	37.1	2q32.3	STK17B	serine/threonine-protein kinase 17B [Homo sapiens].
240	NP_056202.2	225	26	5.4	26.2	2q33.1	MOB4	MOB-like protein phocein isoform 1 [Homo sapiens].
241	NP_001094289.1	204	24	6.5	27.9	2q33.1	MOB4	MOB-like protein phocein isoform 3 [Homo sapiens].
242	NP_955776.1	193	22	5.7	29.0	2q33.1	MOB4	MOB-like protein phocein isoform 2 [Homo sapiens].
243	NP_001191023.1	193	22	5.7	29.0	2q33.1	MOB4	MOB-like protein phocein isoform 2 [Homo sapiens].
244	NP_001155879.1	526	62	9.1	22.2	2q33	CLK1	dual specificity protein kinase CLK1 isoform 2 [Homo sapiens].
245	NP_004062.2	484	57	9.0	22.9	2q33	CLK1	dual specificity protein kinase CLK1 isoform 1 [Homo sapiens].
246	NP_061041.2	418	47	6.5	38.8	2q33.1	STRADB	STE20-related kinase adapter protein beta isoform 1 [Homo sapiens].
247	NP_001193793.1	377	42	7.9	39.8	2q33.1	STRADB	STE20-related kinase adapter protein beta isoform 2 [Homo sapiens].
248	NP_001248364.1	429	48	7.1	36.1	2q33.2	CDK15	cyclin-dependent kinase 15 isoform 1 [Homo sapiens].
249	NP_001248365.1	400	45	6.5	36.7	2q33.2	CDK15	cyclin-dependent kinase 15 isoform 2 [Homo sapiens].
250	NP_631897.1	384	44	7.1	38.5	2q33.2	CDK15	cyclin-dependent kinase 15 isoform 3 [Homo sapiens].
251	NP_055744.2	710	81	7.9	47.9	2q33.3	FASTKD2	FAST kinase domain-containing protein 2 [Homo sapiens].
252	NP_001129665.1	710	81	7.9	47.9	2q33.3	FASTKD2	FAST kinase domain-containing protein 2 [Homo sapiens].
253	NP_001129666.1	710	81	7.9	47.9	2q33.3	FASTKD2	FAST kinase domain-containing protein 2 [Homo sapiens].
254	NP_055855.2	2098	237	6.2	27.2	2q34	PIKFYVE	1-phosphatidylinositol 3-phosphate 5-kinase isoform 2 [Homo
255	NP_001171471.1	548	62	6.4	18.1	2q34	PIKFYVE	1-phosphatidylinositol 3-phosphate 5-kinase isoform 4 [Homo
256	NP_689884.1	451	50	5.7	19.1	2q34	PIKFYVE	1-phosphatidylinositol 3-phosphate 5-kinase isoform 3 [Homo
257	NP_001036064.1	1292	143	6.0	31.0	2q33.3-q34	ERBB4	receptor tyrosine-protein kinase erbB-4 isoform JM-a/CVT-2
258	NP_005226.1	1308	144	6.0	30.6	2q33.3-q34	ERBB4	receptor tyrosine-protein kinase erbB-4 isoform JM-a/CVT-1
259	NP_056505.2	1315	144	5.5	51.6	2q35	STK36	serine/threonine-protein kinase 36 isoform 1 [Homo sapiens].
260	NP_001230242.1	1294	142	5.5	51.2	2q35	STK36	serine/threonine-protein kinase 36 isoform 2 [Homo sapiens].
261	NP_059127.2	489	54	5.5	44.0	2q35	PRKAG3	5'-AMP-activated protein kinase subunit gamma-3 [Homo sapiens].
262	NP_003927.1	367	39	10.1	33.8	2q35	CDK5R2	cyclin-dependent kinase 5 activator 2 [Homo sapiens].
263	NP_001008910.1	305	35	6.4	40.0	2q35	STK16	serine/threonine-protein kinase 16 [Homo sapiens].
264	NP_005867.3	3267	354	8.4	28.2	2q35	SPEG	striated muscle preferentially expressed protein kinase isoform 1
265	NP_001166947.1	113	13	9.7	19.5	2q35	SPEG	striated muscle preferentially expressed protein kinase isoform 4
266	NP_443134.2	1099	121	5.2	41.6	2q35	STK11IP	serine/threonine-protein kinase 11-interacting protein [Homo
267	NP_004429.1	986	110	6.2	34.8	2q36.1	EPHA4	ephrin type-A receptor 4 precursor [Homo sapiens].
268	NP_065915.1	653	71	8.5	20.2	2q36.3	NYAP2	neuronal tyrosine-phosphorylated phosphoinositide-3-kinase adapter
269	NP_085126.2	1671	183	4.9	28.7	2q36	SPHKAP	A-kinase anchor protein SPHKAP isoform 2 [Homo sapiens].
270	NP_001136116.1	1700	186	4.9	28.8	2q36	SPHKAP	A-kinase anchor protein SPHKAP isoform 1 [Homo sapiens].
271	NP_690618.2	1214	135	7.3	32.4	2q37.1	DGKD	diacylglycerol kinase delta isoform 2 [Homo sapiens].
272	NP_003639.2	1170	130	7.4	33.8	2q37.1	DGKD	diacylglycerol kinase delta isoform 1 [Homo sapiens].
273	NP_110395.1	392	43	6.7	30.9	2q37.3	ILKAP	integrin-linked kinase-associated serine/threonine phosphatase 2C
274	NP_001239051.1	1288	139	4.6	41.9	2q37.3	PASK	PAS domain-containing serine/threonine-protein kinase isoform 3
275	NP_001239049.1	1323	143	4.6	42.3	2q37.3	PASK	PAS domain-containing serine/threonine-protein kinase isoform 2
276	NP_055963.2	1323	143	4.6	42.3	2q37.3	PASK	PAS domain-containing serine/threonine-protein kinase isoform 2
277	NP_001239048.1	1330	144	4.6	42.0	2q37.3	PASK	PAS domain-containing serine/threonine-protein kinase isoform 1
278	NP_001239053.1	1143	123	4.8	41.4	2q37.3	PASK	PAS domain-containing serine/threonine-protein kinase isoform 4
279	NP_006365.2	426	48	6.3	31.0	2q37.3	STK25	serine/threonine-protein kinase 25 [Homo sapiens].
280	NP_001158503.1	188	21	9.1	30.9	2q37.3	DTYMK	thymidylate kinase isoform 2 [Homo sapiens].
281	NP_036277.2	212	24	8.4	32.1	2q37.3	DTYMK	thymidylate kinase isoform 1 [Homo sapiens].
282	NP_003647.1	370	41	5.0	35.4	3p25.3	CAMK1	calcium/calmodulin-dependent protein kinase type 1 [Homo sapiens].
283	NP_955379.2	712	82	7.8	38.6	3p24.1	NEK10	serine/threonine-protein kinase Nek10 [Homo sapiens].
284	NP_208382.1	648	74	9.1	23.8	3p22.2	DCLK3	serine/threonine-protein kinase DCLK3 [Homo sapiens].
285	NP_005100.1	527	58	6.0	34.3	3p22.2	OXSR1	serine/threonine-protein kinase OSR1 [Homo sapiens].
286	NP_060356.2	1275	142	5.9	49.7	3p22.1	ULK4	serine/threonine-protein kinase ULK4 [Homo sapiens].
287	NP_005784.1	194	22	8.0	47.4	3p21	NME6	nucleoside diphosphate kinase 6 [Homo sapiens].
288	NP_004558.1	469	54	6.2	32.6	3p22-p21	PFKFB4	6-phosphofructo-2-kinase/fructose-2,6-bisphosphatase 4 [Homo
289	NP_057375.2	426	49	6.4	27.9	3p21.31	IP6K2	inositol hexakisphosphate kinase 2 isoform a [Homo sapiens].
290	NP_001005909.1	426	49	6.4	27.9	3p21.31	IP6K2	inositol hexakisphosphate kinase 2 isoform a [Homo sapiens].
291	NP_001005910.1	97	11	9.4	32.0	3p21.31	IP6K2	inositol hexakisphosphate kinase 2 isoform b [Homo sapiens].

292	NP_001005911.1	97	11	9.4	32.0	3p21.31	IP6K2	inositol hexakisphosphate kinase 2 isoform b [Homo sapiens].
293	NP_001139650.1	87	10	7.4	28.7	3p21.31	IP6K2	inositol hexakisphosphate kinase 2 isoform c [Homo sapiens].
294	NP_001139651.1	87	10	7.4	28.7	3p21.31	IP6K2	inositol hexakisphosphate kinase 2 isoform c [Homo sapiens].
295	NP_001177246.1	188	21	8.2	31.4	3p21.31	IP6K2	inositol hexakisphosphate kinase 2 isoform e [Homo sapiens].
296	NP_001177245.1	185	21	8.7	33.5	3p21.31	IP6K2	inositol hexakisphosphate kinase 2 isoform d [Homo sapiens].
297	NP_004148.1	404	46	4.8	33.4	3p21.3-p21	PRKAR2A	cAMP-dependent protein kinase type II-alpha regulatory subunit
298	NP_001229758.1	441	50	6.8	24.3	3p21.31	IP6K1	inositol hexakisphosphate kinase 1 isoform 1 [Homo sapiens].
299	NP_695005.1	441	50	6.8	24.3	3p21.31	IP6K1	inositol hexakisphosphate kinase 1 isoform 1 [Homo sapiens].
300	NP_001006115.1	276	32	8.6	27.2	3p21.31	IP6K1	inositol hexakisphosphate kinase 1 isoform 2 [Homo sapiens].
301	NP_001231866.1	1351	147	6.2	48.9	3p21.3	MST1R	macrophage-stimulating protein receptor isoform 2 precursor [Homo
302	NP_002438.2	1400	150	6.1	49.1	3p21.3	MST1R	macrophage-stimulating protein receptor isoform 1 preproprotein
302	NP_002438.2	1400	30	5.6	51.1	3p21.3	MST1R	macrophage-stimulating protein receptor isoform 1 preproprotein
302	NP_002438.2	1400	119	6.0	48.8	3p21.3	MST1R	macrophage-stimulating protein receptor isoform 1 preproprotein
303	NP_004626.1	382	43	6.9	28.0	3p21.3	MAPKAPK3	MAP kinase-activated protein kinase 3 [Homo sapiens].
304	NP_001230854.1	382	43	6.9	28.0	3p21.3	MAPKAPK3	MAP kinase-activated protein kinase 3 [Homo sapiens].
305	NP_001230855.1	382	43	6.9	28.0	3p21.3	MAPKAPK3	MAP kinase-activated protein kinase 3 [Homo sapiens].
306	NP_009215.1	349	40	6.4	24.4	3p21.1	TWF2	twinfilin-2 [Homo sapiens].
307	NP_660305.2	523	55	6.3	53.0	3p21.1	GLYCTK	glycerate kinase isoform 1 [Homo sapiens].
308	NP_001138423.1	234	25	8.6	44.9	3p21.1	GLYCTK	glycerate kinase isoform 2 [Homo sapiens].
309	NP_001180462.1	752	84	7.6	27.4	3p21.1	NEK4	serine/threonine-protein kinase Nek4 isoform 2 [Homo sapiens].
310	NP_003148.2	841	95	7.8	27.6	3p21.1	NEK4	serine/threonine-protein kinase Nek4 isoform 1 [Homo sapiens].
311	NP_006245.2	676	78	7.6	33.4	3p21.31	PRKCD	protein kinase C delta type [Homo sapiens].
312	NP_997704.1	676	78	7.6	33.4	3p21.31	PRKCD	protein kinase C delta type [Homo sapiens].
313	NP_060241.2	578	65	9.7	31.0	3p14.3	PXK	PX domain-containing protein kinase-like protein [Homo sapiens].
314	NP_001028229.1	1462	162	7.4	22.9	3p14.1	MAGI1	membrane-associated guanylate kinase, WW and PDZ domain-containing
315	NP_056335.1	1287	140	5.7	27.7	3p14.1	MAGI1	membrane-associated guanylate kinase, WW and PDZ domain-containing
316	NP_004733.2	1256	137	5.5	27.0	3p14.1	MAGI1	membrane-associated guanylate kinase, WW and PDZ domain-containing
317	NP_005224.2	983	110	6.3	34.0	3p11.2	EPHA3	ephrin type-A receptor 3 isoform a precursor [Homo sapiens].
318	NP_872585.1	539	61	5.5	29.5	3p11.2	EPHA3	ephrin type-A receptor 3 isoform b precursor [Homo sapiens].
319	NP_001073917.2	1130	127	6.5	37.1	3q11.2	EPHA6	ephrin type-A receptor 6 isoform a [Homo sapiens].
320	NP_775926.1	334	37	8.6	42.8	3q11.2	EPHA6	ephrin type-A receptor 6 isoform b [Homo sapiens].
321	NP_001139628.1	420	47	8.9	39.3	3q13.3	GSK3B	glycogen synthase kinase-3 beta isoform 2 [Homo sapiens].
322	NP_002084.2	433	48	8.9	39.0	3q13.3	GSK3B	glycogen synthase kinase-3 beta isoform 1 [Homo sapiens].
323	NP_001019831.2	2986	340	5.7	29.4	3q21.2	KALRN	kalirin isoform 1 [Homo sapiens].
324	NP_003938.1	1663	192	5.7	27.4	3q21.2	KALRN	kalirin isoform 2 [Homo sapiens].
325	NP_008995.2	1289	144	5.9	30.5	3q21.2	KALRN	kalirin isoform 3 [Homo sapiens].
326	NP_055417.1	1358	153	6.7	39.4	3q22.1	PIK3R4	phosphoinositide 3-kinase regulatory subunit 4 [Homo sapiens].
327	NP_079076.3	645	74	4.9	29.0	3q22.1	NEK11	serine/threonine-protein kinase Nek11 isoform 1 [Homo sapiens].
328	NP_001139475.1	599	69	5.2	28.9	3q22.1	NEK11	serine/threonine-protein kinase Nek11 isoform 3 [Homo sapiens].
329	NP_665917.1	470	54	5.6	30.9	3q22.1	NEK11	serine/threonine-protein kinase Nek11 isoform 2 [Homo sapiens].
330	NP_002949.2	607	68	6.9	40.9	3q22	RYK	tyrosine-protein kinase RYK isoform 2 precursor [Homo sapiens].
331	NP_001005861.1	610	68	6.9	41.6	3q22	RYK	tyrosine-protein kinase RYK isoform 1 precursor [Homo sapiens].
332	NP_004432.1	984	108	6.1	37.8	3q21-q23	EPHB1	ephrin type-B receptor 1 precursor [Homo sapiens].
333	NP_006210.1	1070	123	6.7	42.8	3q22.3	PIK3CB	phosphatidylinositol 4,5-bisphosphate 3-kinase catalytic subunit
334	NP_001242974.1	582	67	8.1	41.8	3q22.3	PIK3CB	phosphatidylinositol 4,5-bisphosphate 3-kinase catalytic subunit
335	NP_631948.1	553	62	6.2	38.7	3q24	GRK7	G protein-coupled receptor kinase 7 [Homo sapiens].
336	NP_001034636.1	529	59	6.5	45.7	3q23	GK5	putative glycerol kinase 5 [Homo sapiens].
337	NP_001175.2	2644	301	7.1	45.4	3q23	ATR	serine/threonine-protein kinase ATR [Homo sapiens].
338	NP_002731.4	596	68	5.5	26.8	3q26.3	PRKCI	protein kinase C iota type [Homo sapiens].
339	NP_001155038.1	1268	144	6.7	24.6	3q26.31	TNIK	TRAF2 and NCK-interacting protein kinase isoform 8 [Homo sapiens].
340	NP_001155037.1	1276	145	6.7	24.3	3q26.31	TNIK	TRAF2 and NCK-interacting protein kinase isoform 7 [Homo sapiens].
341	NP_001155034.1	1323	150	6.9	23.9	3q26.31	TNIK	TRAF2 and NCK-interacting protein kinase isoform 4 [Homo sapiens].
342	NP_001155033.1	1331	151	6.9	23.6	3q26.31	TNIK	TRAF2 and NCK-interacting protein kinase isoform 3 [Homo sapiens].
343	NP_001155036.1	1297	148	6.5	24.4	3q26.31	TNIK	TRAF2 and NCK-interacting protein kinase isoform 6 [Homo sapiens].
344	NP_001155035.1	1305	149	6.5	24.1	3q26.31	TNIK	TRAF2 and NCK-interacting protein kinase isoform 5 [Homo sapiens].
345	NP_001155032.1	1352	154	6.7	23.7	3q26.31	TNIK	TRAF2 and NCK-interacting protein kinase isoform 2 [Homo sapiens].
346	NP_055843.1	1360	155	6.7	23.4	3q26.31	TNIK	TRAF2 and NCK-interacting protein kinase isoform 1 [Homo sapiens].
347	NP_006209.2	1068	124	6.8	39.9	3q26.3	PIK3CA	phosphatidylinositol 4,5-bisphosphate 3-kinase catalytic subunit
348	NP_150284.1	79	9	5.2	3.8	3q27.1	CAMK2N2	calcium/calmodulin-dependent protein kinase II inhibitor 2 [Homo

349	NP_004434.2	998	107	5.7	37.2	3q21-qter	EPHB3	ephrin type-B receptor 3 precursor [Homo sapiens].
350	NP_001229246.1	759	85	6.5	22.7	3q27	MAP3K13	mitogen-activated protein kinase kinase kinase 13 isoform 2 [Homo
351	NP_004712.1	966	108	6.0	22.7	3q27	MAP3K13	mitogen-activated protein kinase kinase kinase 13 isoform 1 [Homo
352	NP_001229243.1	966	108	6.0	22.7	3q27	MAP3K13	mitogen-activated protein kinase kinase kinase 13 isoform 1 [Homo
353	NP_001074214.1	752	85	6.0	31.6	3q27.2-q27	DGKG	diacylglycerol kinase gamma isoform 3 [Homo sapiens].
354	NP_001074213.1	766	86	6.2	31.9	3q27.2-q27	DGKG	diacylglycerol kinase gamma isoform 2 [Homo sapiens].
355	NP_001337.2	791	89	6.4	31.2	3q27.2-q27	DGKG	diacylglycerol kinase gamma isoform 1 [Homo sapiens].
356	NP_001010938.1	1086	119	7.4	26.3	3q29	TNK2	activated CDC42 kinase 1 isoform 2 [Homo sapiens].
357	NP_005772.3	1038	115	6.8	26.2	3q29	TNK2	activated CDC42 kinase 1 isoform 1 [Homo sapiens].
358	NP_002568.2	524	58	5.6	36.8	3q29	PAK2	serine/threonine-protein kinase PAK 2 [Homo sapiens].
359	NP_001338.2	942	101	7.2	44.9	4p16.3	DGKQ	diacylglycerol kinase theta [Homo sapiens].
360	NP_001156685.1	808	86	5.7	34.0	4p16.3	FGFR3	fibroblast growth factor receptor 3 isoform 3 precursor [Homo
361	NP_000133.1	806	86	5.5	34.3	4p16.3	FGFR3	fibroblast growth factor receptor 3 isoform 1 precursor [Homo
362	NP_075254.1	694	74	5.5	32.0	4p16.3	FGFR3	fibroblast growth factor receptor 3 isoform 2 precursor [Homo
363	NP_892027.2	578	67	7.6	26.0	4p16.3	GRK4	G protein-coupled receptor kinase 4 isoform alpha [Homo sapiens].
364	NP_001004057.1	532	61	7.4	27.4	4p16.3	GRK4	G protein-coupled receptor kinase 4 isoform gamma [Homo sapiens].
365	NP_001004056.1	546	63	6.5	26.2	4p16.3	GRK4	G protein-coupled receptor kinase 4 isoform beta [Homo sapiens].
366	NP_775931.3	504	53	6.4	33.5	4p16.3	DOK7	protein Dok-7 isoform 1 [Homo sapiens].
367	NP_001158145.1	255	28	8.9	38.0	4p16.3	DOK7	protein Dok-7 isoform 2 [Homo sapiens].
368	NP_001243825.1	194	20	7.1	24.7	4p16.3	DOK7	protein Dok-7 isoform 3 [Homo sapiens].
369	NP_060871.1	414	48	7.5	29.5	4p16.2	STK32B	serine/threonine-protein kinase 32B [Homo sapiens].
370	NP_001092903.1	831	97	5.5	16.6	4p16.1	JAKMIP1	janus kinase and microtubule-interacting protein 1 isoform 1 [Homo
371	NP_653321.1	626	73	5.8	16.6	4p16.1	JAKMIP1	janus kinase and microtubule-interacting protein 1 isoform 2 [Homo
372	NP_060793.2	481	55	5.6	32.2	4p15.2	PI4K2B	phosphatidylinositol 4-kinase type 2-beta [Homo sapiens].
373	NP_003319.2	527	61	7.9	33.0	4p12	TXK	tyrosine-protein kinase TXK [Homo sapiens].
374	NP_003206.2	631	74	8.4	25.0	4p12	TEC	tyrosine-protein kinase Tec [Homo sapiens].
375	NP_000213.1	976	110	6.5	40.1	4q11-q12	KIT	mast/stem cell growth factor receptor Kit isoform 1 precursor [Homo
376	NP_001087241.1	972	109	6.4	40.2	4q11-q12	KIT	mast/stem cell growth factor receptor Kit isoform 2 precursor [Homo
377	NP_002244.1	1356	149	5.5	37.4	4q11-q12	KDR	vascular endothelial growth factor receptor 2 precursor [Homo
378	NP_872272.2	1015	110	6.2	35.8	4q13.1	EPHA5	ephrin type-A receptor 5 isoform b precursor [Homo sapiens].
379	NP_004430.4	1037	112	6.2	37.1	4q13.1	EPHA5	ephrin type-A receptor 5 isoform a precursor [Homo sapiens].
380	NP_775739.1	216	25	6.3	35.2	4q13.3	MOB1B	MOB kinase activator 1B isoform 2 [Homo sapiens].
381	NP_001231696.1	147	17	5.0	34.7	4q13.3	MOB1B	MOB kinase activator 1B isoform 3 [Homo sapiens].
382	NP_001231695.1	221	25	5.7	33.5	4q13.3	MOB1B	MOB kinase activator 1B isoform 1 [Homo sapiens].
383	NP_003939.1	493	56	8.2	35.5	4q21.1	CDKL2	cyclin-dependent kinase-like 2 [Homo sapiens].
384	NP_942595.1	1161	129	6.0	26.2	4q21.21	BMP2K	BMP-2-inducible protein kinase isoform a [Homo sapiens].
385	NP_060063.2	662	74	7.8	32.3	4q21.21	BMP2K	BMP-2-inducible protein kinase isoform b [Homo sapiens].
386	NP_001035292.1	311	36	8.5	56.9	4q21.21	PAQR3	progestin and adipoQ receptor family member 3 [Homo sapiens].
387	NP_149991.2	553	61	5.5	52.1	4q13	GK2	glycerol kinase 2 [Homo sapiens].
388	NP_006250.1	762	87	8.5	28.1	4q13.1-q21	PRKG2	cGMP-dependent protein kinase 2 [Homo sapiens].
389	NP_620448.1	464	53	6.3	36.2	4q22.1-q23	MAPK10	mitogen-activated protein kinase 10 isoform 2 [Homo sapiens].
390	NP_620446.1	426	48	6.4	35.9	4q22.1-q23	MAPK10	mitogen-activated protein kinase 10 isoform 3 [Homo sapiens].
391	NP_002744.1	422	49	7.5	37.7	4q22.1-q23	MAPK10	mitogen-activated protein kinase 10 isoform 1 [Homo sapiens].
392	NP_620447.1	277	32	6.1	35.4	4q22.1-q23	MAPK10	mitogen-activated protein kinase 10 isoform 4 [Homo sapiens].
393	NP_001243722.1	532	61	7.1	31.0	4q22-q24	BMPR1B	bone morphogenetic protein receptor type-1B isoform a precursor
394	NP_001194.1	502	56	7.1	29.7	4q22-q24	BMPR1B	bone morphogenetic protein receptor type-1B isoform b precursor
395	NP_001243721.1	502	56	7.1	29.7	4q22-q24	BMPR1B	bone morphogenetic protein receptor type-1B isoform b precursor
396	NP_001243723.1	502	56	7.1	29.7	4q22-q24	BMPR1B	bone morphogenetic protein receptor type-1B isoform b precursor
397	NP_001230665.1	117	13	8.6	47.0	4q23	LAMTOR3	ragulator complex protein LAMTOR3 isoform 2 [Homo sapiens].
398	NP_068805.1	124	14	7.3	46.8	4q23	LAMTOR3	ragulator complex protein LAMTOR3 isoform 1 [Homo sapiens].
399	NP_005434.4	624	71	6.4	34.1	4q24	PAPSS1	bifunctional 3'-phosphoadenosine 5'-phosphosulfate synthase 1 [Homo
400	NP_079420.3	1244	139	5.8	30.3	4q25	ALPK1	alpha-protein kinase 1 isoform 1 [Homo sapiens].
401	NP_001095876.1	1244	139	5.8	30.3	4q25	ALPK1	alpha-protein kinase 1 isoform 1 [Homo sapiens].
402	NP_001240813.1	1166	130	6.1	29.5	4q25	ALPK1	alpha-protein kinase 1 isoform 2 [Homo sapiens].
403	NP_742126.1	478	54	6.7	31.6	4q26	CAMK2D	calcium/calmodulin-dependent protein kinase type II subunit delta
404	NP_001212.2	499	56	6.8	30.9	4q26	CAMK2D	calcium/calmodulin-dependent protein kinase type II subunit delta
405	NP_742125.1	478	54	6.8	31.6	4q26	CAMK2D	calcium/calmodulin-dependent protein kinase type II subunit delta
406	NP_742113.1	478	54	6.8	31.6	4q26	CAMK2D	calcium/calmodulin-dependent protein kinase type II subunit delta
407	NP_742112.1	492	56	6.7	30.7	4q26	CAMK2D	calcium/calmodulin-dependent protein kinase type II subunit delta

408	NP_742127.1	489	55	7.5	30.9	4q26	CAMK2D	calcium/calmodulin-dependent protein kinase type II subunit delta
409	NP_055079.3	970	109	8.7	28.6	4q28	PLK4	serine/threonine-protein kinase PLK4 isoform 1 [Homo sapiens].
410	NP_001177728.1	938	105	8.5	28.9	4q28	PLK4	serine/threonine-protein kinase PLK4 isoform 2 [Homo sapiens].
411	NP_001177730.1	929	105	8.8	27.2	4q28	PLK4	serine/threonine-protein kinase PLK4 isoform 3 [Homo sapiens].
412	NP_078881.3	786	89	6.8	25.6	4q31.23	ARHGAP10	rho GTPase-activating protein 10 [Homo sapiens].
413	NP_001035351.4	783	85	8.5	31.7	4q31.3	DCLK2	serine/threonine-protein kinase DCLK2 isoform b [Homo sapiens].
414	NP_001035350.2	766	84	8.3	32.4	4q31.3	DCLK2	serine/threonine-protein kinase DCLK2 isoform a [Homo sapiens].
415	NP_001186328.1	1189	135	5.3	22.7	4q33	NEK1	serine/threonine-protein kinase Nek1 isoform 4 [Homo sapiens].
416	NP_001186329.1	1214	138	5.4	22.8	4q33	NEK1	serine/threonine-protein kinase Nek1 isoform 5 [Homo sapiens].
417	NP_001186327.1	1242	141	5.5	22.7	4q33	NEK1	serine/threonine-protein kinase Nek1 isoform 3 [Homo sapiens].
418	NP_036356.1	1258	143	5.6	22.6	4q33	NEK1	serine/threonine-protein kinase Nek1 isoform 2 [Homo sapiens].
419	NP_001186326.1	1286	146	5.7	22.5	4q33	NEK1	serine/threonine-protein kinase Nek1 isoform 1 [Homo sapiens].
420	NP_008961.1	219	24	9.9	20.1	5p15.3	TPPP	tubulin polymerization-promoting protein [Homo sapiens].
421	NP_001180384.1	732	82	6.6	26.5	5p15.31	NSUN2	tRNA (cytosine(34)-C(5))-methyltransferase isoform 2 [Homo
422	NP_060225.4	767	86	6.3	25.7	5p15.31	NSUN2	tRNA (cytosine(34)-C(5))-methyltransferase isoform 1 [Homo
423	NP_076996.2	662	76	8.2	46.1	5p15.31	FASTKD3	FAST kinase domain-containing protein 3 [Homo sapiens].
424	NP_001078880.1	442	49	8.1	31.4	5p13.2	NADKD1	NAD kinase domain-containing protein 1 isoform 1 [Homo sapiens].
425	NP_694558.1	279	32	6.3	29.7	5p13.2	NADKD1	NAD kinase domain-containing protein 1 isoform 2 [Homo sapiens].
426	NP_006242.5	559	64	8.0	30.1	5p12	PRKAA1	5'-AMP-activated protein kinase catalytic subunit alpha-1 isoform 1
427	NP_996790.3	574	66	8.2	29.8	5p12	PRKAA1	5'-AMP-activated protein kinase catalytic subunit alpha-1 isoform 2
428	NP_699192.1	436	50	8.4	28.2	5p12	NIM1	serine/threonine-protein kinase NIM1 [Homo sapiens].
429	NP_005912.1	1512	164	7.5	33.4	5q11.2	MAP3K1	mitogen-activated protein kinase kinase kinase 1 [Homo sapiens].
430	NP_001239155.1	671	77	8.2	31.3	5q12.1-q13	PLK2	serine/threonine-protein kinase PLK2 isoform 2 [Homo sapiens].
431	NP_006613.2	685	78	8.1	30.1	5q12.1-q13	PLK2	serine/threonine-protein kinase PLK2 isoform 1 [Homo sapiens].
432	NP_001158136.1	2623	284	8.7	20.2	5q12.3	MAST4	microtubule-associated serine/threonine-protein kinase 4 isoform c
433	NP_942123.1	250	26	9.0	29.6	5q12.3	MAST4	microtubule-associated serine/threonine-protein kinase 4 isoform b
434	NP_055998.1	2434	265	8.8	18.8	5q12.3	MAST4	microtubule-associated serine/threonine-protein kinase 4 isoform a
435	NP_852664.1	724	84	5.8	24.2	5q13.1	PIK3R1	phosphatidylinositol 3-kinase regulatory subunit alpha isoform 1
436	NP_852665.1	424	50	7.4	14.2	5q13.1	PIK3R1	phosphatidylinositol 3-kinase regulatory subunit alpha isoform 3
437	NP_852556.2	454	53	6.0	15.4	5q13.1	PIK3R1	phosphatidylinositol 3-kinase regulatory subunit alpha isoform 2
438	NP_001229395.1	361	43	8.1	15.5	5q13.1	PIK3R1	phosphatidylinositol 3-kinase regulatory subunit alpha isoform 4
439	NP_001790.1	346	39	8.6	40.5	5q12.1	CDK7	cyclin-dependent kinase 7 [Homo sapiens].
440	NP_001015891.1	169	20	4.5	21.3	5q11.2-q13	TAF9	adenylate kinase isoenzyme 6 isoform c [Homo sapiens].
441	NP_057367.1	172	20	4.3	25.0	5q11.2-q13	TAF9	adenylate kinase isoenzyme 6 isoform b [Homo sapiens].
442	NP_001015892.1	264	29	9.0	31.8	5q11.2-q13	TAF9	adenylate kinase isoenzyme 6 isoform a [Homo sapiens].
443	NP_003178.1	264	29	9.0	31.8	5q11.2-q13	TAF9	adenylate kinase isoenzyme 6 isoform a [Homo sapiens].
444	NP_001816.2	419	43	7.2	27.9	5q13.3	CKMT2	creatine kinase S-type, mitochondrial precursor [Homo sapiens].
445	NP_001093205.1	419	43	7.2	27.9	5q13.3	CKMT2	creatine kinase S-type, mitochondrial precursor [Homo sapiens].
446	NP_001093206.1	419	43	7.2	27.9	5q13.3	CKMT2	creatine kinase S-type, mitochondrial precursor [Homo sapiens].
447	NP_001230.1	323	38	6.8	39.0	5q13.3-q14	CCNH	cyclin-H isoform 1 [Homo sapiens].
448	NP_001186118.1	270	31	6.0	43.7	5q13.3-q14	CCNH	cyclin-H isoform 2 [Homo sapiens].
449	NP_060813.2	552	63	5.6	21.2	5q15	RIOK2	serine/threonine-protein kinase RIO2 isoform 1 [Homo sapiens].
450	NP_001153221.1	474	55	5.1	21.3	5q15	RIOK2	serine/threonine-protein kinase RIO2 isoform 2 [Homo sapiens].
451	NP_056031.2	1222	138	7.9	29.8	5q21.1	PPIP5K2	inositol hexakisphosphate and diphosphoinositol-pentakisphosphate
452	NP_001953.1	228	24	6.1	28.8	5q21	EFNA5	ephrin-A5 precursor [Homo sapiens].
453	NP_005237.2	822	95	6.7	26.6	5q21	FER	tyrosine-protein kinase Fer [Homo sapiens].
454	NP_001735.1	473	52	5.5	38.7	5q21.3	CAMK4	calcium/calmodulin-dependent protein kinase type IV [Homo sapiens].
455	NP_114417.1	367	42	7.6	30.2	5q22.2	TSSK1B	testis-specific serine/threonine-protein kinase 1 [Homo sapiens].
456	NP_001257501.1	424	49	9.4	23.1	5q23	CSNK1G3	casein kinase I isoform gamma-3 isoform 5 [Homo sapiens].
457	NP_001038188.1	455	52	9.5	23.1	5q23	CSNK1G3	casein kinase I isoform gamma-3 isoform 4 [Homo sapiens].
458	NP_004375.2	447	51	9.5	23.0	5q23	CSNK1G3	casein kinase I isoform gamma-3 isoform 1 [Homo sapiens].
459	NP_001026982.1	423	49	9.5	23.6	5q23	CSNK1G3	casein kinase I isoform gamma-3 isoform 2 [Homo sapiens].
460	NP_001257502.1	348	41	8.9	23.6	5q23	CSNK1G3	casein kinase I isoform gamma-3 isoform 6 [Homo sapiens].
461	NP_001257503.1	311	36	8.4	22.8	5q23	CSNK1G3	casein kinase I isoform gamma-3 isoform 7 [Homo sapiens].
462	NP_005331.1	126	14	6.5	37.3	5q31.2	HINT1	histidine triad nucleotide-binding protein 1 [Homo sapiens].
463	NP_064625.1	84	9	8.5	33.3	5q31.1	CDC42SE2	CDC42 small effector protein 2 [Homo sapiens].
464	NP_001033791.1	84	9	8.5	33.3	5q31.1	CDC42SE2	CDC42 small effector protein 2 [Homo sapiens].
465	NP_001107047.1	592	68	9.7	27.7	5q31	CDKL3	cyclin-dependent kinase-like 3 isoform 1 [Homo sapiens].
466	NP_057592.2	455	52	9.3	33.0	5q31	CDKL3	cyclin-dependent kinase-like 3 isoform 2 [Homo sapiens].

467	NP_003542.1	212	24	5.9	38.2	5q31	NME5	nucleoside diphosphate kinase homolog 5 [Homo sapiens].
468	NP_055886.1	814	92	6.2	31.2	5q31	ARHGAP26	rho GTPase-activating protein 26 isoform a [Homo sapiens].
469	NP_001129080.1	759	86	6.2	28.3	5q31	ARHGAP26	rho GTPase-activating protein 26 isoform b [Homo sapiens].
470	NP_001106195.1	396	46	7.0	29.0	5q32	STK32A	serine/threonine-protein kinase 32A isoform 1 [Homo sapiens].
471	NP_659438.1	166	20	5.8	30.1	5q32	STK32A	serine/threonine-protein kinase 32A isoform 2 [Homo sapiens].
472	NP_055605.2	810	95	5.8	14.4	5q32	JAKMIP2	janus kinase and microtubule-interacting protein 2 isoform 3 [Homo
473	NP_001257863.1	799	94	5.7	16.1	5q32	JAKMIP2	janus kinase and microtubule-interacting protein 2 isoform 2 [Homo
474	NP_001257870.1	820	96	5.7	15.9	5q32	JAKMIP2	janus kinase and microtubule-interacting protein 2 [Homo sapiens].
475	NP_001883.4	337	39	9.9	32.0	5q32	CSNK1A1	casein kinase I isoform alpha isoform 2 [Homo sapiens].
476	NP_001020276.1	365	42	9.9	30.1	5q32	CSNK1A1	casein kinase I isoform alpha isoform 1 [Homo sapiens].
477	NP_741960.1	478	54	6.6	33.1	5q32	CAMK2A	calcium/calmodulin-dependent protein kinase type II subunit alpha
478	NP_057065.2	489	55	7.1	32.3	5q32	CAMK2A	calcium/calmodulin-dependent protein kinase type II subunit alpha
479	NP_005537.3	620	72	7.4	32.3	5q31-q32	ITK	tyrosine-protein kinase ITK/TSK [Homo sapiens].
480	NP_078870.1	370	41	6.1	43.5	5q34	PANK3	pantothenate kinase 3 [Homo sapiens].
481	NP_005981.3	968	112	6.5	18.9	5q35.1	STK10	serine/threonine-protein kinase 10 [Homo sapiens].
482	NP_001017995.1	911	102	8.9	19.2	5q35.1	SH3PXD2B	SH3 and PX domain-containing protein 2B [Homo sapiens].
483	NP_004408.1	367	39	6.8	53.4	5q34	DUSP1	dual specificity protein phosphatase 1 [Homo sapiens].
484	NP_002106.2	923	99	5.1	53.2	5q35.2	HK3	hexokinase-3 [Homo sapiens].
485	NP_002002.3	802	86	6.4	41.4	5q35.1-qte	FGFR4	fibroblast growth factor receptor 4 isoform 1 precursor [Homo
486	NP_998812.1	802	86	6.4	41.4	5q35.1-qte	FGFR4	fibroblast growth factor receptor 4 isoform 1 precursor [Homo
487	NP_075252.2	762	81	6.3	41.3	5q35.1-qte	FGFR4	fibroblast growth factor receptor 4 isoform 2 precursor [Homo
488	NP_002073.2	589	67	8.5	28.7	5q35	GRK6	G protein-coupled receptor kinase 6 isoform B [Homo sapiens].
489	NP_001004106.1	576	66	7.9	28.5	5q35	GRK6	G protein-coupled receptor kinase 6 isoform A [Homo sapiens].
490	NP_001004105.1	560	64	8.4	29.3	5q35	GRK6	G protein-coupled receptor kinase 6 isoform C [Homo sapiens].
491	NP_001138348.1	228	25	8.2	30.3	5q35.3	DOK3	docking protein 3 isoform 3 [Homo sapiens].
492	NP_001138347.1	330	36	8.6	32.7	5q35.3	DOK3	docking protein 3 isoform 2 [Homo sapiens].
493	NP_079148.2	496	53	7.6	27.8	5q35.3	DOK3	docking protein 3 isoform 1 [Homo sapiens].
494	NP_065717.1	481	57	8.7	24.7	5q35	CLK4	dual specificity protein kinase CLK4 [Homo sapiens].
495	NP_002743.3	424	48	5.3	38.0	5q35	MAPK9	mitogen-activated protein kinase 9 isoform JNK2 alpha2 [Homo
496	NP_620709.1	424	48	5.4	37.5	5q35	MAPK9	mitogen-activated protein kinase 9 isoform JNK2 beta2 [Homo
497	NP_620707.1	382	44	6.0	40.1	5q35	MAPK9	mitogen-activated protein kinase 9 isoform JNK2 alpha1 [Homo
498	NP_620708.1	382	44	6.1	39.5	5q35	MAPK9	mitogen-activated protein kinase 9 isoform JNK2 beta1 [Homo
499	NP_001128516.1	242	27	8.2	56.6	5q35	MAPK9	mitogen-activated protein kinase 9 isoform JNK2 gamma [Homo
500	NP_891555.2	1363	150	5.8	37.6	5q35.3	FLT4	vascular endothelial growth factor receptor 3 isoform 1 precursor
501	NP_002011.2	1298	143	5.8	39.2	5q35.3	FLT4	vascular endothelial growth factor receptor 3 isoform 2 precursor
502	NP_006089.1	317	35	7.5	35.6	5q35.3	GNB2L1	guanine nucleotide-binding protein subunit beta-2-like 1 [Homo
503	NP_064570.1	184	21	8.1	35.9	6p25.3	DUSP22	dual specificity protein phosphatase 22 [Homo sapiens].
504	NP_001012418.2	388	45	5.9	30.7	6p25.2	MYLK4	myosin light chain kinase family member 4 [Homo sapiens].
505	NP_003795.2	671	76	5.9	24.9	6p25.2	RIPK1	receptor-interacting serine/threonine-protein kinase 1 [Homo
506	NP_003904.3	1007	117	10.7	16.9	6p25.2	PRPF4B	serine/threonine-protein kinase PRP4 homolog [Homo sapiens].
507	NP_113668.2	568	66	5.8	20.6	6p24.3	RIOK1	serine/threonine-protein kinase RIO1 isoform 1 [Homo sapiens].
508	NP_694550.1	327	38	6.7	23.9	6p24.3	RIOK1	serine/threonine-protein kinase RIO1 isoform 2 [Homo sapiens].
509	NP_001229314.1	583	66	10.0	25.4	6p24	MAK	serine/threonine-protein kinase MAK isoform 2 [Homo sapiens].
510	NP_005897.1	623	71	10.0	25.2	6p24	MAK	serine/threonine-protein kinase MAK isoform 1 [Homo sapiens].
511	NP_001229886.1	648	73	9.9	24.7	6p24	MAK	serine/threonine-protein kinase MAK isoform 3 [Homo sapiens].
512	NP_001189452.1	894	95	6.2	34.3	6p21.3	DDR1	epithelial discoidin domain-containing receptor 1 isoform 6
513	NP_054700.2	919	100	6.6	35.4	6p21.3	DDR1	epithelial discoidin domain-containing receptor 1 isoform 3
514	NP_054699.2	913	99	6.6	34.9	6p21.3	DDR1	epithelial discoidin domain-containing receptor 1 isoform 2
515	NP_001945.3	876	95	6.2	34.3	6p21.3	DDR1	epithelial discoidin domain-containing receptor 1 isoform 1
516	NP_001189451.1	767	84	6.8	37.3	6p21.3	DDR1	epithelial discoidin domain-containing receptor 1 isoform 5
517	NP_001189450.1	508	54	7.2	31.8	6p21.3	DDR1	epithelial discoidin domain-containing receptor 1 isoform 4
518	NP_001311.3	215	25	5.2	29.3	6p21.3	CSNK2B	casein kinase II subunit beta [Homo sapiens].
519	NP_115830.1	368	41	10.1	37.2	6p21.3	STK19	serine/threonine-protein kinase 19 isoform 2 [Homo sapiens].
520	NP_004188.1	364	40	10.3	35.7	6p21.3	STK19	serine/threonine-protein kinase 19 isoform 1 [Homo sapiens].
521	NP_473452.2	410	46	8.0	26.3	6p21.31	IP6K3	inositol hexakisphosphate kinase 3 [Homo sapiens].
522	NP_001136355.1	410	46	8.0	26.3	6p21.31	IP6K3	inositol hexakisphosphate kinase 3 [Homo sapiens].
523	NP_065855.1	444	51	5.0	6.5	6p21.3	PACSIN1	protein kinase C and casein kinase substrate in neurons protein 1
524	NP_001186512.1	444	51	5.0	6.5	6p21.3	PACSIN1	protein kinase C and casein kinase substrate in neurons protein 1
525	NP_003128.3	655	74	5.8	26.7	6p21.31	SRPK1	SRSF protein kinase 1 [Homo sapiens].

526	NP_620581.1	360	41	5.4	39.4	6p21.3-p21	MAPK14	mitogen-activated protein kinase 14 isoform 2 [Homo sapiens].
527	NP_001306.1	360	41	5.5	37.2	6p21.3-p21	MAPK14	mitogen-activated protein kinase 14 isoform 1 [Homo sapiens].
528	NP_620583.1	307	35	8.9	41.7	6p21.3-p21	MAPK14	mitogen-activated protein kinase 14 isoform 4 [Homo sapiens].
529	NP_620582.1	297	34	8.2	42.1	6p21.3-p21	MAPK14	mitogen-activated protein kinase 14 isoform 3 [Homo sapiens].
530	NP_002745.1	365	42	8.6	32.6	6p21.31	MAPK13	mitogen-activated protein kinase 13 [Homo sapiens].
531	NP_009202.1	465	54	6.7	29.7	6p21	STK38	serine/threonine-protein kinase 38 [Homo sapiens].
532	NP_000380.1	164	18	8.4	17.1	6p21.2	CDKN1A	cyclin-dependent kinase inhibitor 1 [Homo sapiens].
533	NP_510867.1	164	18	8.4	17.1	6p21.2	CDKN1A	cyclin-dependent kinase inhibitor 1 [Homo sapiens].
534	NP_001207706.1	164	18	8.4	17.1	6p21.2	CDKN1A	cyclin-dependent kinase inhibitor 1 [Homo sapiens].
535	NP_001207707.1	164	18	8.4	17.1	6p21.2	CDKN1A	cyclin-dependent kinase inhibitor 1 [Homo sapiens].
536	NP_001230115.1	404	45	6.5	35.1	6p21.2	PIM1	serine/threonine-protein kinase pim-1 isoform 1 [Homo sapiens].
537	NP_002639.1	313	36	5.7	39.9	6p21.2	PIM1	serine/threonine-protein kinase pim-1 isoform 2 [Homo sapiens].
538	NP_002812.2	1070	115	6.4	32.1	6p21.1-p12	PTK7	inactive tyrosine-protein kinase 7 isoform a precursor [Homo
539	NP_690621.1	1014	109	6.3	31.9	6p21.1-p12	PTK7	inactive tyrosine-protein kinase 7 isoform d precursor [Homo
540	NP_690619.1	1030	111	6.3	33.3	6p21.1-p12	PTK7	inactive tyrosine-protein kinase 7 isoform b precursor [Homo
541	NP_690620.1	940	100	6.4	34.1	6p21.1-p12	PTK7	inactive tyrosine-protein kinase 7 isoform c precursor [Homo
542	NP_001257327.1	1078	119	6.5	31.9	6p21.1-p12	PTK7	inactive tyrosine-protein kinase 7 isoform e [Homo sapiens].
543	NP_115927.1	1321	143	5.4	21.8	6p21.1	TTBK1	tau-tubulin kinase 1 [Homo sapiens].
544	NP_620061.2	417	45	8.7	49.9	6p12.3	PGK2	phosphoglycerate kinase 2 [Homo sapiens].
545	NP_055735.1	632	71	10.1	22.3	6p12.1	ICK	serine/threonine-protein kinase ICK [Homo sapiens].
546	NP_057597.2	632	71	10.1	22.3	6p12.1	ICK	serine/threonine-protein kinase ICK [Homo sapiens].
547	NP_001010844.1	260	29	9.2	31.5	6q14-q15	IRAK1BP1	interleukin-1 receptor-associated kinase 1-binding protein 1 [Homo
548	NP_003309.2	857	97	8.1	25.0	6q13-q21	TTK	dual specificity protein kinase TTK isoform 1 [Homo sapiens].
549	NP_001160163.1	856	97	8.1	25.0	6q13-q21	TTK	dual specificity protein kinase TTK isoform 2 [Homo sapiens].
550	NP_056340.2	1353	151	7.5	37.5	6q14.1	IBTK	inhibitor of Bruton tyrosine kinase [Homo sapiens].
551	NP_003179.1	579	64	6.2	29.4	6q15	MAP3K7	mitogen-activated protein kinase kinase kinase 7 isoform A [Homo
552	NP_663304.1	606	67	6.7	30.7	6q15	MAP3K7	mitogen-activated protein kinase kinase kinase 7 isoform B [Homo
553	NP_663306.1	491	54	6.2	32.6	6q15	MAP3K7	mitogen-activated protein kinase kinase kinase 7 isoform D [Homo
554	NP_663305.1	518	57	6.9	34.0	6q15	MAP3K7	mitogen-activated protein kinase kinase kinase 7 isoform C [Homo
555	NP_004431.1	998	112	5.5	35.8	6q16.1	EPHA7	ephrin type-A receptor 7 precursor [Homo sapiens].
556	NP_001138600.2	1911	221	4.8	29.5	6q21	AKD1	adenylate kinase domain-containing protein 1 isoform 1 [Homo
557	NP_659462.1	421	49	4.8	37.5	6q21	AKD1	adenylate kinase domain-containing protein 1 isoform 2 [Homo
558	NP_055891.1	502	57	8.7	27.9	6q21	CDK19	cyclin-dependent kinase 19 [Homo sapiens].
559	NP_694593.1	482	55	5.8	25.9	6q21	FYN	tyrosine-protein kinase Fyn isoform c [Homo sapiens].
560	NP_694592.1	534	60	5.8	27.7	6q21	FYN	tyrosine-protein kinase Fyn isoform b [Homo sapiens].
561	NP_002028.1	537	61	6.2	26.3	6q21	FYN	tyrosine-protein kinase Fyn isoform a [Homo sapiens].
562	NP_002347.5	332	32	4.3	19.3	6q22.2	MARCKS	myristoylated alanine-rich C-kinase substrate [Homo sapiens].
563	NP_002022.1	505	58	6.2	29.3	6q21-q22.3	FRK	tyrosine-protein kinase FRK [Homo sapiens].
564	NP_002935.2	2347	260	5.6	43.5	6q22	ROS1	proto-oncogene tyrosine-protein kinase ROS precursor [Homo
565	NP_001257323.1	85	9	4.6	17.6	6q22.31	PKIB	cAMP-dependent protein kinase inhibitor beta isoform 2 [Homo
566	NP_001257324.1	85	9	4.6	17.6	6q22.31	PKIB	cAMP-dependent protein kinase inhibitor beta isoform 2 [Homo
567	NP_861460.1	78	8	4.6	19.2	6q22.31	PKIB	cAMP-dependent protein kinase inhibitor beta isoform 1 [Homo
568	NP_861459.1	78	8	4.6	19.2	6q22.31	PKIB	cAMP-dependent protein kinase inhibitor beta isoform 1 [Homo
569	NP_115860.1	78	8	4.6	19.2	6q22.31	PKIB	cAMP-dependent protein kinase inhibitor beta isoform 1 [Homo
570	NP_001257322.1	78	8	4.6	19.2	6q22.31	PKIB	cAMP-dependent protein kinase inhibitor beta isoform 1 [Homo
571	NP_057461.2	348	40	5.8	31.3	6q23	AKAP7	A-kinase anchor protein 7 isoform gamma isoform gamma [Homo
572	NP_619539.1	104	11	4.7	13.5	6q23	AKAP7	A-kinase anchor protein 7 isoform gamma isoform beta [Homo
573	NP_004833.1	81	9	4.8	16.0	6q23	AKAP7	A-kinase anchor protein 7 isoform gamma isoform alpha [Homo
574	NP_001137148.1	526	60	7.9	29.1	6q23	SGK1	serine/threonine-protein kinase Sgk1 isoform 2 [Homo sapiens].
575	NP_001137149.1	459	52	8.6	29.8	6q23	SGK1	serine/threonine-protein kinase Sgk1 isoform 3 [Homo sapiens].
576	NP_001137150.1	445	51	9.0	32.6	6q23	SGK1	serine/threonine-protein kinase Sgk1 isoform 4 [Homo sapiens].
577	NP_005618.2	431	49	8.7	33.2	6q23	SGK1	serine/threonine-protein kinase Sgk1 isoform 1 [Homo sapiens].
578	NP_005914.1	1374	155	5.4	37.2	6q22.33	MAP3K5	mitogen-activated protein kinase kinase kinase 5 [Homo sapiens].
579	NP_055908.1	693	76	8.5	20.3	6q25.1	TAB2	TGF-beta-activated kinase 1 and MAP3K7-binding protein 2 [Homo
580	NP_004681.1	1130	127	8.9	20.5	6q25.1	LATS1	serine/threonine-protein kinase LATS1 isoform 1 [Homo sapiens].
581	NP_001257448.1	690	76	10.2	16.5	6q25.1	LATS1	serine/threonine-protein kinase LATS1 isoform 2 [Homo sapiens].
582	NP_112211.1	165	18	4.9	20.0	6q24.3-q25	PPP1R14C	protein phosphatase 1 regulatory subunit 14C [Homo sapiens].
583	NP_005091.2	1782	191	4.2	15.3	6q24-q25	AKAP12	A-kinase anchor protein 12 isoform 1 [Homo sapiens].
584	NP_653080.1	1684	182	4.3	15.6	6q24-q25	AKAP12	A-kinase anchor protein 12 isoform 2 [Homo sapiens].

585	NP_775786.2	555	62	8.9	27.4	6q25.2	CNKSR3	connector enhancer of kinase suppressor of ras 3 [Homo sapiens].
586	NP_005913.2	1608	182	5.9	31.0	6q26	MAP3K4	mitogen-activated protein kinase kinase kinase 4 isoform a [Homo
587	NP_006715.2	1558	177	5.8	30.7	6q26	MAP3K4	mitogen-activated protein kinase kinase kinase 4 isoform b [Homo
588	NP_001006933.1	741	84	6.2	35.2	6q27	RPS6KA2	ribosomal protein S6 kinase alpha-2 isoform b [Homo sapiens].
589	NP_066958.2	733	83	8.9	35.7	6q27	RPS6KA2	ribosomal protein S6 kinase alpha-2 isoform a [Homo sapiens].
590	NP_064608.2	584	64	6.8	30.2	7p22.3	FAM20C	extracellular serine/threonine protein kinase Fam20C precursor
591	NP_002726.1	381	43	5.4	30.7	7p22	PRKAR1B	cAMP-dependent protein kinase type I-beta regulatory subunit [Homo
592	NP_001158230.1	381	43	5.4	30.7	7p22	PRKAR1B	cAMP-dependent protein kinase type I-beta regulatory subunit [Homo
593	NP_001158231.1	381	43	5.4	30.7	7p22	PRKAR1B	cAMP-dependent protein kinase type I-beta regulatory subunit [Homo
594	NP_001158232.1	381	43	5.4	30.7	7p22	PRKAR1B	cAMP-dependent protein kinase type I-beta regulatory subunit [Homo
595	NP_001158234.1	381	43	5.4	30.7	7p22	PRKAR1B	cAMP-dependent protein kinase type I-beta regulatory subunit [Homo
596	NP_001158233.1	381	43	5.4	30.7	7p22	PRKAR1B	cAMP-dependent protein kinase type I-beta regulatory subunit [Homo
597	NP_001127807.1	629	71	5.6	32.9	7p22	EIF2AK1	eukaryotic translation initiation factor 2-alpha kinase 1 isoform b
598	NP_055228.2	630	71	5.6	33.0	7p22	EIF2AK1	eukaryotic translation initiation factor 2-alpha kinase 1 isoform a
599	NP_004071.1	804	91	7.7	32.5	7p21.2	DGKB	diacylglycerol kinase beta isoform 1 [Homo sapiens].
600	NP_663733.1	773	87	6.8	32.6	7p21.2	DGKB	diacylglycerol kinase beta isoform 2 [Homo sapiens].
601	NP_787082.1	318	35	5.9	58.8	7p21.1	PRPS1L1	ribose-phosphate pyrophosphokinase 3 [Homo sapiens].
602	NP_113602.2	1019	116	4.9	32.2	7p15.3	STK31	serine/threonine-protein kinase 31 isoform a [Homo sapiens].
603	NP_001247434.1	996	113	4.9	32.7	7p15.3	STK31	serine/threonine-protein kinase 31 isoform c [Homo sapiens].
604	NP_116562.2	996	113	4.9	32.0	7p15.3	STK31	serine/threonine-protein kinase 31 isoform b [Homo sapiens].
605	NP_001247433.1	996	113	4.9	32.0	7p15.3	STK31	serine/threonine-protein kinase 31 isoform b [Homo sapiens].
606	NP_976033.1	221	26	10.2	3.6	7p14.3	RP9	retinitis pigmentosa 9 protein [Homo sapiens].
607	NP_003709.3	1512	165	10.1	21.0	7p13	CDK13	cyclin-dependent kinase 13 isoform 1 [Homo sapiens].
608	NP_112557.2	1452	158	10.1	19.9	7p13	CDK13	cyclin-dependent kinase 13 isoform 2 [Homo sapiens].
609	NP_004751.2	414	47	4.9	31.9	7p13	STK17A	serine/threonine-protein kinase 17A [Homo sapiens].
610	NP_000153.1	465	52	5.0	34.6	7p15.3-p15	GCK	glucokinase isoform 1 [Homo sapiens].
611	NP_277042.1	466	52	4.9	36.3	7p15.3-p15	GCK	glucokinase isoform 2 [Homo sapiens].
612	NP_277043.1	464	52	5.0	34.7	7p15.3-p15	GCK	glucokinase isoform 3 [Homo sapiens].
613	NP_742081.1	449	51	7.0	35.9	7p14.3-p14	CAMK2B	calcium/calmodulin-dependent protein kinase type II subunit beta
614	NP_742077.1	517	58	6.9	32.5	7p14.3-p14	CAMK2B	calcium/calmodulin-dependent protein kinase type II subunit beta
615	NP_742080.1	479	54	6.7	34.2	7p14.3-p14	CAMK2B	calcium/calmodulin-dependent protein kinase type II subunit beta
616	NP_742078.1	503	56	7.0	33.4	7p14.3-p14	CAMK2B	calcium/calmodulin-dependent protein kinase type II subunit beta
617	NP_742076.1	518	58	6.9	33.0	7p14.3-p14	CAMK2B	calcium/calmodulin-dependent protein kinase type II subunit beta
618	NP_742079.1	492	55	7.1	35.0	7p14.3-p14	CAMK2B	calcium/calmodulin-dependent protein kinase type II subunit beta
619	NP_742075.1	542	60	6.9	34.1	7p14.3-p14	CAMK2B	calcium/calmodulin-dependent protein kinase type II subunit beta
620	NP_001211.3	666	73	6.9	32.7	7p14.3-p14	CAMK2B	calcium/calmodulin-dependent protein kinase type II subunit beta
621	NP_001248763.1	642	72	6.9	46.9	7p13	TBRG4	protein TBRG4 isoform 3 [Homo sapiens].
622	NP_112162.1	521	58	6.6	41.8	7p13	TBRG4	protein TBRG4 isoform 2 [Homo sapiens].
623	NP_954573.1	521	58	6.6	41.8	7p13	TBRG4	protein TBRG4 isoform 2 [Homo sapiens].
624	NP_004740.2	631	71	7.0	46.1	7p13	TBRG4	protein TBRG4 isoform 1 [Homo sapiens].
625	NP_005219.2	1210	132	6.1	31.2	7p12	EGFR	epidermal growth factor receptor isoform a precursor [Homo
626	NP_958441.1	705	75	6.4	33.3	7p12	EGFR	epidermal growth factor receptor isoform d precursor [Homo
627	NP_958439.1	628	67	6.5	31.6	7p12	EGFR	epidermal growth factor receptor isoform b precursor [Homo
628	NP_958440.1	405	42	6.2	30.7	7p12	EGFR	epidermal growth factor receptor isoform c precursor [Homo
629	NP_001245389.1	378	44	6.4	34.1	7p11.2	PHKG1	phosphorylase b kinase gamma catalytic chain, skeletal muscle
630	NP_006204.1	387	45	6.4	34.1	7p11.2	PHKG1	phosphorylase b kinase gamma catalytic chain, skeletal muscle
631	NP_001245388.1	419	49	6.3	31.5	7p11.2	PHKG1	phosphorylase b kinase gamma catalytic chain, skeletal muscle
632	NP_115784.1	1483	171	8.5	21.5	7q11.23	BAZ1B	tyrosine-protein kinase BAZ1B [Homo sapiens].
633	NP_002305.1	647	73	6.5	32.0	7q11.23	LIMK1	LIM domain kinase 1 isoform 1 [Homo sapiens].
634	NP_001191355.1	613	69	7.6	31.3	7q11.23	LIMK1	LIM domain kinase 1 isoform 2 [Homo sapiens].
635	NP_057170.1	313	36	5.7	39.6	7q11.23	STYXL1	serine/threonine/tyrosine-interacting-like protein 1 [Homo
636	NP_036611.2	247	28	4.7	22.7	7q11.23	YWHAG	14-3-3 protein gamma [Homo sapiens].
637	NP_036433.2	1455	159	5.9	24.3	7q21	MAGI2	membrane-associated guanylate kinase, WW and PDZ domain-containing
638	NP_036527.1	451	51	9.0	31.3	7q21-q22	CDK14	cyclin-dependent kinase 14 [Homo sapiens].
639	NP_005742.4	3907	453	4.8	16.9	7q21-q22	AKAP9	A-kinase anchor protein 9 isoform 2 [Homo sapiens].
640	NP_671714.1	3899	452	4.8	16.8	7q21-q22	AKAP9	A-kinase anchor protein 9 isoform 3 [Homo sapiens].
641	NP_001250.1	326	37	6.0	39.6	7q21-q22	CDK6	cyclin-dependent kinase 6 [Homo sapiens].
642	NP_001138778.1	326	37	6.0	39.6	7q21-q22	CDK6	cyclin-dependent kinase 6 [Homo sapiens].
643	NP_002603.1	411	46	6.2	40.1	7q21.3	PDK4	pyruvate dehydrogenase kinase, isozyme 4 [Homo sapiens].

644	NP_055731.2	1503	162	4.2	26.6	7q21.3	LMTK2	serine/threonine-protein kinase LMTK2 precursor [Homo sapiens].
645	NP_061330.2	511	57	8.9	22.9	7q22.1	BAIAP2L1	brain-specific angiogenesis inhibitor 1-associated protein 2-like
646	NP_775835.2	841	88	10.0	25.6	7q22.1	NYAP1	neuronal tyrosine-phosphorylated phosphoinositide-3-kinase adapter
647	NP_004435.3	987	107	6.5	36.4	7q22	EPHB4	ephrin type-B receptor 4 precursor [Homo sapiens].
648	NP_872634.1	699	79	5.0	26.3	7q22-q31.1	SRPK2	SRSF protein kinase 2 isoform a [Homo sapiens].
649	NP_872633.1	688	78	4.7	26.2	7q22-q31.1	SRPK2	SRSF protein kinase 2 isoform b [Homo sapiens].
650	NP_002640.2	1102	126	7.2	37.2	7q22.3	PIK3CG	phosphatidylinositol 4,5-bisphosphate 3-kinase catalytic subunit
651	NP_002727.2	418	46	4.7	38.5	7q22	PRKAR2B	cAMP-dependent protein kinase type II-beta regulatory subunit [Homo
652	NP_001120972.1	1408	155	6.8	42.7	7q31	MET	hepatocyte growth factor receptor isoform a precursor [Homo
653	NP_000236.2	1390	153	6.8	42.5	7q31	MET	hepatocyte growth factor receptor isoform b precursor [Homo
654	NP_057562.3	502	55	5.3	29.9	7q32.2	ZC3HC1	nuclear-interacting partner of ALK [Homo sapiens].
655	NP_653249.1	825	94	6.0	31.0	7q33	LRGUK	leucine-rich repeat and guanylate kinase domain-containing protein
656	NP_004708.1	1065	117	7.6	35.5	7q32.3-q33	DGKI	diacylglycerol kinase iota [Homo sapiens].
657	NP_001106710.1	1171	128	8.4	37.0	7q32-q34	HIPK2	homeodomain-interacting protein kinase 2 isoform 2 [Homo sapiens].
658	NP_073577.3	1198	131	8.3	37.6	7q32-q34	HIPK2	homeodomain-interacting protein kinase 2 isoform 1 [Homo sapiens].
659	NP_443085.2	626	69	8.8	55.1	7q34	ADCK2	uncharacterized aarF domain-containing protein kinase 2 [Homo
660	NP_004324.2	766	84	7.3	34.2	7q34	BRAF	serine/threonine-protein kinase B-raf [Homo sapiens].
661	NP_001099028.1	567	63	6.1	21.5	7q32	WEE2	wee1-like protein kinase 2 [Homo sapiens].
662	NP_005223.4	976	108	6.2	33.7	7q34	EPHA1	ephrin type-A receptor 1 precursor [Homo sapiens].
663	NP_001035947.1	194	22	5.1	37.1	7q34-q35	TPK1	thiamin pyrophosphokinase 1 isoform b [Homo sapiens].
664	NP_071890.2	243	27	4.9	47.7	7q34-q35	TPK1	thiamin pyrophosphokinase 1 isoform a [Homo sapiens].
665	NP_001157882.1	260	30	6.0	35.8	7q36	CDK5	cyclin-dependent kinase 5 isoform 2 [Homo sapiens].
666	NP_004926.1	292	33	7.5	37.3	7q36	CDK5	cyclin-dependent kinase 5 isoform 1 [Homo sapiens].
667	NP_148936.2	408	46	9.9	41.9	7q35	FASTK	fas-activated serine/threonine kinase isoform 4 [Homo sapiens].
668	NP_001245390.1	522	58	10.1	42.3	7q35	FASTK	fas-activated serine/threonine kinase isoform 5 [Homo sapiens].
669	NP_006703.1	549	61	10.2	43.0	7q35	FASTK	fas-activated serine/threonine kinase isoform 1 [Homo sapiens].
670	NP_057287.2	569	63	9.7	38.3	7q36.1	PRKAG2	5'-AMP-activated protein kinase subunit gamma-2 isoform a [Homo
671	NP_001035723.1	525	58	9.3	39.0	7q36.1	PRKAG2	5'-AMP-activated protein kinase subunit gamma-2 isoform c [Homo
672	NP_077747.1	328	38	5.9	54.3	7q36.1	PRKAG2	5'-AMP-activated protein kinase subunit gamma-2 isoform b [Homo
673	NP_001074295.1	1402	149	6.8	24.5	8p23.1	SGK223	tyrosine-protein kinase SgK223 [Homo sapiens].
674	NP_001706.2	505	58	7.8	35.4	8p23-p22	BLK	tyrosine-protein kinase Blk [Homo sapiens].
675	NP_003965.2	412	45	5.7	23.1	8p21.3	DOK2	docking protein 2 [Homo sapiens].
676	NP_775266.1	1009	116	5.9	33.7	8p21.1	PTK2B	protein-tyrosine kinase 2-beta isoform a [Homo sapiens].
677	NP_004094.3	1009	116	5.9	33.7	8p21.1	PTK2B	protein-tyrosine kinase 2-beta isoform a [Homo sapiens].
678	NP_775268.1	1009	116	5.9	33.7	8p21.1	PTK2B	protein-tyrosine kinase 2-beta isoform a [Homo sapiens].
679	NP_775267.1	967	111	5.6	34.0	8p21.1	PTK2B	protein-tyrosine kinase 2-beta isoform b [Homo sapiens].
680	NP_056069.2	1826	203	5.6	30.1	8p12	KIF13B	kinesin-like protein KIF13B [Homo sapiens].
681	NP_001385.1	394	43	7.1	44.7	8p12-p11	DUSP4	dual specificity protein phosphatase 4 isoform 1 [Homo sapiens].
682	NP_476499.1	303	33	7.9	42.2	8p12-p11	DUSP4	dual specificity protein phosphatase 4 isoform 2 [Homo sapiens].
683	NP_076930.1	211	24	9.9	42.2	8p12	DUSP26	dual specificity protein phosphatase 26 [Homo sapiens].
684	NP_001167538.1	853	93	5.8	34.7	8p12	FGFR1	fibroblast growth factor receptor 1 isoform 14 precursor [Homo
685	NP_075594.1	731	79	6.0	33.4	8p12	FGFR1	fibroblast growth factor receptor 1 isoform 4 precursor [Homo
686	NP_075593.1	733	80	6.1	33.4	8p12	FGFR1	fibroblast growth factor receptor 1 isoform 3 precursor [Homo
687	NP_001167537.1	733	80	6.1	33.4	8p12	FGFR1	fibroblast growth factor receptor 1 isoform 3 precursor [Homo
688	NP_056934.2	820	89	5.7	33.8	8p12	FGFR1	fibroblast growth factor receptor 1 isoform 2 precursor [Homo
689	NP_001167536.1	820	89	5.7	33.8	8p12	FGFR1	fibroblast growth factor receptor 1 isoform 2 precursor [Homo
690	NP_001167534.1	820	89	5.7	33.3	8p12	FGFR1	fibroblast growth factor receptor 1 isoform 10 precursor [Homo
691	NP_075598.2	822	89	5.7	33.8	8p12	FGFR1	fibroblast growth factor receptor 1 isoform 1 precursor [Homo
692	NP_001167535.1	812	88	5.7	33.6	8p12	FGFR1	fibroblast growth factor receptor 1 isoform 11 precursor [Homo
693	NP_001177649.1	754	86	5.6	29.2	8p11.2	IKBKB	inhibitor of nuclear factor kappa-B kinase subunit beta isoform 2
694	NP_001547.1	756	87	5.5	29.9	8p11.2	IKBKB	inhibitor of nuclear factor kappa-B kinase subunit beta isoform 1
695	NP_001229707.1	697	80	5.2	31.1	8p11.2	IKBKB	inhibitor of nuclear factor kappa-B kinase subunit beta isoform 5
696	NP_115613.1	350	40	5.7	34.3	8p11.21	SGK196	protein kinase-like protein SgK196 [Homo sapiens].
697	NP_001075109.1	4097	466	6.8	43.0	8q11	PRKDC	DNA-dependent protein kinase catalytic subunit isoform 2 [Homo
698	NP_008835.5	4128	469	6.7	43.0	8q11	PRKDC	DNA-dependent protein kinase catalytic subunit isoform 1 [Homo
699	NP_002341.1	512	59	6.8	31.3	8q13	LYN	tyrosine-protein kinase Lyn isoform A [Homo sapiens].
700	NP_001104567.1	491	56	6.1	32.6	8q13	LYN	tyrosine-protein kinase Lyn isoform B [Homo sapiens].
701	NP_005363.1	346	38	9.0	43.4	8q11	MOS	proto-oncogene serine/threonine-protein kinase mos [Homo sapiens].
702	NP_001191102.1	496	57	6.5	37.3	8	C8orf44-S	serine/threonine-protein kinase Sgk3 [Homo sapiens].

703	NP_037389.4	496	57	6.5	37.3	8q12	SGK3	serine/threonine-protein kinase Sgk3 isoform 1 [Homo sapiens].
704	NP_001028750.1	496	57	6.5	37.3	8q12	SGK3	serine/threonine-protein kinase Sgk3 isoform 1 [Homo sapiens].
705	NP_733827.2	464	53	6.8	35.8	8q12	SGK3	serine/threonine-protein kinase Sgk3 isoform 2 [Homo sapiens].
706	NP_006814.1	76	8	4.3	27.6	8q21.12	PKIA	cAMP-dependent protein kinase inhibitor alpha [Homo sapiens].
707	NP_862822.1	76	8	4.3	27.6	8q21.12	PKIA	cAMP-dependent protein kinase inhibitor alpha [Homo sapiens].
708	NP_149117.1	385	43	10.0	35.3	8q21.3	PSKH2	serine/threonine-protein kinase H2 [Homo sapiens].
709	NP_003812.1	540	61	6.7	31.1	8q21	RIPK2	receptor-interacting serine/threonine-protein kinase 2 [Homo
710	NP_001243241.1	519	59	5.0	29.7	8q22.2	STK3	serine/threonine-protein kinase 3 isoform 2 [Homo sapiens].
711	NP_001243242.1	380	44	4.9	22.1	8q22.2	STK3	serine/threonine-protein kinase 3 isoform 3 [Homo sapiens].
712	NP_006272.2	491	37	5.0	37.6	8q22.2	STK3	serine/threonine-protein kinase 3 isoform 1 [Homo sapiens].
712	NP_006272.2	491	20	4.9	11.8	8q22.2	STK3	serine/threonine-protein kinase 3 isoform 1 [Homo sapiens].
713	NP_003397.1	245	28	4.6	25.7	8q23.1	YWHAZ	14-3-3 protein zeta/delta [Homo sapiens].
714	NP_663723.1	245	28	4.6	25.7	8q23.1	YWHAZ	14-3-3 protein zeta/delta [Homo sapiens].
715	NP_001129171.1	245	28	4.6	25.7	8q23.1	YWHAZ	14-3-3 protein zeta/delta [Homo sapiens].
716	NP_001129172.1	245	28	4.6	25.7	8q23.1	YWHAZ	14-3-3 protein zeta/delta [Homo sapiens].
717	NP_001129173.1	245	28	4.6	25.7	8q23.1	YWHAZ	14-3-3 protein zeta/delta [Homo sapiens].
718	NP_001129174.1	245	28	4.6	25.7	8q23.1	YWHAZ	14-3-3 protein zeta/delta [Homo sapiens].
719	NP_005598.3	1074	122	6.1	31.1	8q24.3	PTK2	focal adhesion kinase 1 isoform b [Homo sapiens].
720	NP_722560.1	1052	119	6.2	30.6	8q24.3	PTK2	focal adhesion kinase 1 isoform a [Homo sapiens].
721	NP_001186578.1	1065	121	6.3	30.2	8q24.3	PTK2	focal adhesion kinase 1 isoform c [Homo sapiens].
722	NP_620590.2	544	60	9.1	36.9	8q24.3	MAPK15	mitogen-activated protein kinase 15 [Homo sapiens].
723	NP_777582.3	580	66	9.1	50.9	8q24.3	ADCK5	uncharacterized aarF domain-containing protein kinase 5 [Homo
724	NP_001186781.1	187	21	9.9	20.3	9p24.1	AK3	GTP:AMP phosphotransferase, mitochondrial isoform b [Homo sapiens].
725	NP_057366.2	227	26	9.5	27.8	9p24.1	AK3	GTP:AMP phosphotransferase, mitochondrial isoform a [Homo sapiens].
726	NP_001186782.1	157	18	9.2	19.1	9p24.1	AK3	GTP:AMP phosphotransferase, mitochondrial isoform c [Homo sapiens].
727	NP_001186784.1	157	18	9.2	19.1	9p24.1	AK3	GTP:AMP phosphotransferase, mitochondrial isoform c [Homo sapiens].
728	NP_001186785.1	157	18	9.2	19.1	9p24.1	AK3	GTP:AMP phosphotransferase, mitochondrial isoform c [Homo sapiens].
729	NP_004963.1	1132	131	6.8	32.0	9p24	JAK2	tyrosine-protein kinase JAK2 [Homo sapiens].
730	NP_001001874.1	136	15	10.2	28.7	9p24.1	TPD52L3	tumor protein D55 isoform 2 [Homo sapiens].
731	NP_001001875.1	132	15	10.2	23.5	9p24.1	TPD52L3	tumor protein D55 isoform 3 [Homo sapiens].
732	NP_277051.3	140	15	10.4	28.6	9p24.1	TPD52L3	tumor protein D55 isoform 1 [Homo sapiens].
733	NP_000068.1	156	17	5.4	34.0	9p21	CDKN2A	cyclin-dependent kinase inhibitor 2A isoform p16INK4a [Homo
734	NP_001182061.1	167	18	6.4	34.1	9p21	CDKN2A	cyclin-dependent kinase inhibitor 2A isoform p16gamma [Homo
735	NP_478102.2	132	14	12.9	28.8	9p21	CDKN2A	cyclin-dependent kinase inhibitor 2A isoform p14ARF [Homo sapiens].
736	NP_478104.2	116	12	5.8	19.8	9p21	CDKN2A	cyclin-dependent kinase inhibitor 2A isoform p12 [Homo sapiens].
737	NP_004927.2	138	15	6.1	34.1	9p21	CDKN2B	cyclin-dependent kinase 4 inhibitor B isoform 1 [Homo sapiens].
738	NP_511042.1	78	8	12.2	21.8	9p21	CDKN2B	cyclin-dependent kinase 4 inhibitor B isoform 2 [Homo sapiens].
739	NP_000450.2	1124	124	6.5	34.3	9p21	TEK	angiopoietin-1 receptor precursor [Homo sapiens].
740	NP_079037.3	216	25	8.4	41.2	9p21.2	MOB3B	MOB kinase activator 3B [Homo sapiens].
741	NP_006276.2	626	68	8.0	31.5	9p13	TESK1	dual specificity testis-specific protein kinase 1 [Homo sapiens].
742	NP_115982.1	163	16	7.5	40.4	9p13.3	HINT2	histidine triad nucleotide-binding protein 2, mitochondrial
743	NP_001177317.1	717	79	6.9	48.4	9p13.3	GNE	bifunctional UDP-N-acetylglucosamine
744	NP_001121699.1	753	83	6.5	46.5	9p13.3	GNE	bifunctional UDP-N-acetylglucosamine
745	NP_001177312.1	648	71	6.3	50.0	9p13.3	GNE	bifunctional UDP-N-acetylglucosamine
746	NP_005467.1	722	79	6.3	48.5	9p13.3	GNE	bifunctional UDP-N-acetylglucosamine
747	NP_001177313.1	612	67	6.3	48.2	9p13.3	GNE	bifunctional UDP-N-acetylglucosamine
748	NP_055606.1	651	75	8.8	28.1	9p13.2	MELK	maternal embryonic leucine zipper kinase isoform 1 [Homo sapiens].
749	NP_001243614.1	610	70	9.3	29.2	9p13.2	MELK	maternal embryonic leucine zipper kinase isoform 2 [Homo sapiens].
750	NP_001243616.1	603	69	9.0	27.9	9p13.2	MELK	maternal embryonic leucine zipper kinase isoform 3 [Homo sapiens].
751	NP_001243617.1	580	66	9.1	27.4	9p13.2	MELK	maternal embryonic leucine zipper kinase isoform 4 [Homo sapiens].
752	NP_001243618.1	619	71	9.0	26.5	9p13.2	MELK	maternal embryonic leucine zipper kinase isoform 5 [Homo sapiens].
753	NP_001243620.1	571	66	9.3	26.1	9p13.2	MELK	maternal embryonic leucine zipper kinase isoform 7 [Homo sapiens].
754	NP_001243619.1	580	67	9.1	25.9	9p13.2	MELK	maternal embryonic leucine zipper kinase isoform 6 [Homo sapiens].
755	NP_001243621.1	520	60	9.2	25.0	9p13.2	MELK	maternal embryonic leucine zipper kinase isoform 8 [Homo sapiens].
756	NP_001243622.1	457	53	9.7	22.8	9p13.2	MELK	maternal embryonic leucine zipper kinase isoform 9 [Homo sapiens].
757	NP_003549.1	540	61	6.4	32.4	9q13	PIP5K1B	phosphatidylinositol 4-phosphate 5-kinase type-1 beta isoform 2
758	NP_002723.2	351	40	8.8	28.5	9q13	PRKACG	cAMP-dependent protein kinase catalytic subunit gamma [Homo
759	NP_060132.3	2022	232	7.5	36.4	9q21.13	TRPM6	transient receptor potential cation channel subfamily M member 6
760	NP_001170781.1	2017	231	7.6	36.5	9q21.13	TRPM6	transient receptor potential cation channel subfamily M member 6

761	NP_001170782.1	2017	231	7.5	36.4	9q21.13	TRPM6	transient receptor potential cation channel subfamily M member 6
762	NP_001121075.1	175	20	4.4	30.3	9q21.13	NMRK1	nicotinamide riboside kinase 1 isoform 2 [Homo sapiens].
763	NP_060351.1	199	23	4.7	28.6	9q21.13	NMRK1	nicotinamide riboside kinase 1 isoform 1 [Homo sapiens].
764	NP_001001551.2	187	21	5.8	44.4	9q21.32	IDNK	probable gluconokinase isoform 1 [Homo sapiens].
765	NP_001243844.1	141	16	5.9	44.7	9q21.32	IDNK	probable gluconokinase isoform 3 [Homo sapiens].
766	NP_001129425.1	315	36	9.7	15.9	9q21.32	GKAP1	G kinase-anchoring protein 1 isoform b [Homo sapiens].
767	NP_079487.2	366	42	9.2	13.9	9q21.32	GKAP1	G kinase-anchoring protein 1 isoform a [Homo sapiens].
768	NP_006171.2	838	90	5.9	35.2	9q22.1	NTRK2	BDNF/NT-3 growth factors receptor isoform a precursor [Homo
769	NP_001018074.1	822	88	5.8	35.5	9q22.1	NTRK2	BDNF/NT-3 growth factors receptor isoform c precursor [Homo
770	NP_001018075.1	553	57	5.6	32.6	9q22.1	NTRK2	BDNF/NT-3 growth factors receptor isoform d precursor [Homo
771	NP_001018076.1	537	55	5.4	33.0	9q22.1	NTRK2	BDNF/NT-3 growth factors receptor isoform e precursor [Homo
772	NP_001007098.1	477	49	5.6	36.1	9q22.1	NTRK2	BDNF/NT-3 growth factors receptor isoform b precursor [Homo
773	NP_004929.2	1430	160	6.4	39.7	9q21.33	DAPK1	death-associated protein kinase 1 [Homo sapiens].
774	NP_036251.2	325	36	6.4	44.6	9q22.1	CDK20	cyclin-dependent kinase 20 isoform 2 [Homo sapiens].
775	NP_001034892.1	346	39	6.3	42.2	9q22.1	CDK20	cyclin-dependent kinase 20 isoform 3 [Homo sapiens].
776	NP_848519.1	338	38	6.6	45.3	9q22.1	CDK20	cyclin-dependent kinase 20 isoform 1 [Homo sapiens].
777	NP_001164111.1	243	27	9.7	49.0	9q22.1	CDK20	cyclin-dependent kinase 20 isoform 5 [Homo sapiens].
778	NP_001164110.1	275	31	7.2	49.5	9q22.1	CDK20	cyclin-dependent kinase 20 isoform 4 [Homo sapiens].
779	NP_001818.1	79	10	9.0	11.4	9q22	CKS2	cyclin-dependent kinases regulatory subunit 2 [Homo sapiens].
780	NP_003168.2	635	72	8.3	26.5	9q22	SYK	tyrosine-protein kinase SYK isoform 1 [Homo sapiens].
781	NP_001167638.1	635	72	8.3	26.5	9q22	SYK	tyrosine-protein kinase SYK isoform 1 [Homo sapiens].
782	NP_001128524.1	612	70	7.3	26.0	9q22	SYK	tyrosine-protein kinase SYK isoform 2 [Homo sapiens].
783	NP_001167639.1	612	70	7.3	26.0	9q22	SYK	tyrosine-protein kinase SYK isoform 2 [Homo sapiens].
784	NP_004551.2	943	101	5.9	29.8	9q22	ROR2	tyrosine-protein kinase transmembrane receptor ROR2 precursor [Homo
785	NP_006639.3	2217	234	5.4	31.9	9q22.3	WNK2	serine/threonine-protein kinase WNK2 [Homo sapiens].
786	NP_004603.1	503	54	7.1	38.4	9q22	TGFBR1	TGF-beta receptor type-1 isoform 1 precursor [Homo sapiens].
787	NP_001124388.1	426	45	7.7	37.3	9q22	TGFBR1	TGF-beta receptor type-1 isoform 2 precursor [Homo sapiens].
788	NP_003631.2	1332	150	5.6	40.5	9q31	IKBKAP	elongator complex protein 1 [Homo sapiens].
789	NP_001032370.1	379	42	4.9	16.5	9q31.3	PALM2	paralemmin-2 isoform b [Homo sapiens].
790	NP_443749.5	411	45	4.9	18.7	9q31.3	PALM2	paralemmin-2 isoform a [Homo sapiens].
791	NP_001185585.1	961	106	4.9	14.7	9q31.3	AKAP2	A-kinase anchor protein 2 isoform 3 [Homo sapiens].
792	NP_001004065.2	948	104	4.9	13.9	9q31.3	AKAP2	A-kinase anchor protein 2 isoform 1 [Homo sapiens].
793	NP_001130034.1	859	95	4.9	14.6	9q31.3	AKAP2	A-kinase anchor protein 2 isoform 2 [Homo sapiens].
794	NP_001159752.1	783	88	6.7	44.7	9q31.3-q32	MUSK	muscle, skeletal receptor tyrosine-protein kinase isoform 2 [Homo
795	NP_005583.1	869	97	6.9	46.4	9q31.3-q32	MUSK	muscle, skeletal receptor tyrosine-protein kinase isoform 1 [Homo
796	NP_001159753.1	773	86	7.4	45.9	9q31.3-q32	MUSK	muscle, skeletal receptor tyrosine-protein kinase isoform 3 [Homo
797	NP_001159639.1	331	38	8.7	36.3	9q33.3-q34	NEK6	serine/threonine-protein kinase Nek6 isoform 3 [Homo sapiens].
798	NP_001159641.1	338	38	8.1	35.5	9q33.3-q34	NEK6	serine/threonine-protein kinase Nek6 isoform 4 [Homo sapiens].
799	NP_001138473.1	347	40	8.4	34.6	9q33.3-q34	NEK6	serine/threonine-protein kinase Nek6 isoform 1 [Homo sapiens].
800	NP_001159643.1	347	40	8.4	34.6	9q33.3-q34	NEK6	serine/threonine-protein kinase Nek6 isoform 1 [Homo sapiens].
801	NP_055212.2	313	36	7.9	35.1	9q33.3-q34	NEK6	serine/threonine-protein kinase Nek6 isoform 2 [Homo sapiens].
802	NP_001159640.1	313	36	7.9	35.1	9q33.3-q34	NEK6	serine/threonine-protein kinase Nek6 isoform 2 [Homo sapiens].
803	NP_001159642.1	313	36	7.9	35.1	9q33.3-q34	NEK6	serine/threonine-protein kinase Nek6 isoform 2 [Homo sapiens].
804	NP_077022.1	486	55	8.3	30.0	9q33.3	MAPKAP1	target of rapamycin complex 2 subunit MAPKAP1 isoform 2 [Homo
805	NP_001006620.1	475	54	7.8	29.1	9q33.3	MAPKAP1	target of rapamycin complex 2 subunit MAPKAP1 isoform 3 [Homo
806	NP_001006618.1	522	59	7.3	29.9	9q33.3	MAPKAP1	target of rapamycin complex 2 subunit MAPKAP1 isoform 1 [Homo
807	NP_001006621.1	330	37	6.3	34.2	9q33.3	MAPKAP1	target of rapamycin complex 2 subunit MAPKAP1 isoform 4 [Homo
808	NP_001006622.1	330	37	6.3	34.2	9q33.3	MAPKAP1	target of rapamycin complex 2 subunit MAPKAP1 isoform 4 [Homo
809	NP_001006619.1	323	36	7.7	29.7	9q33.3	MAPKAP1	target of rapamycin complex 2 subunit MAPKAP1 isoform 5 [Homo
810	NP_001252.1	372	43	9.0	34.7	9q34.1	CDK9	cyclin-dependent kinase 9 [Homo sapiens].
811	NP_000467.1	194	22	9.0	28.9	9q34.1	AK1	adenylate kinase isoenzyme 1 [Homo sapiens].
812	NP_001128691.1	394	45	9.9	32.0	9q34.11	PIP5KL1	phosphatidylinositol 4-phosphate 5-kinase-like protein 1 isoform 1
813	NP_775763.1	191	22	9.4	27.7	9q34.11	PIP5KL1	phosphatidylinositol 4-phosphate 5-kinase-like protein 1 isoform 2
814	NP_037487.2	889	99	8.4	35.4	9q34.11	PKN3	serine/threonine-protein kinase N3 [Homo sapiens].
815	NP_055723.1	538	59	8.6	70.6	9q34.11	DOLK	dolichol kinase [Homo sapiens].
816	NP_009297.2	1149	125	9.1	24.7	9q34.1	ABL1	tyrosine-protein kinase ABL1 isoform b [Homo sapiens].
817	NP_005148.2	1130	123	8.8	24.9	9q34.1	ABL1	tyrosine-protein kinase ABL1 isoform a [Homo sapiens].
818	NP_001248379.1	268	31	6.6	28.4	9q34.13	UCK1	uridine-cytidine kinase 1 isoform c [Homo sapiens].
819	NP_001248380.1	282	32	6.4	29.4	9q34.13	UCK1	uridine-cytidine kinase 1 isoform d [Homo sapiens].

820	NP_113620.1	277	31	6.9	28.9	9q34.13	UCK1	uridine-cytidine kinase 1 isoform a [Homo sapiens].
821	NP_001129426.1	201	23	5.8	23.4	9q34.13	UCK1	uridine-cytidine kinase 1 isoform b [Homo sapiens].
822	NP_689785.1	479	55	5.7	40.9	9q34.13	AK8	adenylate kinase 8 [Homo sapiens].
823	NP_714921.3	680	76	5.1	47.6	9q34.2	C9orf96	protein kinase-like protein SgK071 [Homo sapiens].
824	NP_002618.1	784	86	7.3	44.1	10p15.3-p1	PFKP	6-phosphofructokinase type C isoform 1 [Homo sapiens].
825	NP_001229268.1	776	85	8.5	40.1	10p15.3-p1	PFKP	6-phosphofructokinase type C isoform 2 [Homo sapiens].
826	NP_001229342.1	643	74	7.8	29.9	10p15	PRKCQ	protein kinase C theta type isoform 2 [Homo sapiens].
827	NP_006248.1	706	82	7.4	30.2	10p15	PRKCQ	protein kinase C theta type isoform 1 [Homo sapiens].
828	NP_705718.1	385	43	6.8	34.5	10p13	CAMK1D	calcium/calmodulin-dependent protein kinase type 1D isoform 2 [Homo
829	NP_065130.1	357	40	6.1	30.8	10p13	CAMK1D	calcium/calmodulin-dependent protein kinase type 1D isoform 1 [Homo
830	NP_001182533.1	321	35	5.0	47.7	10p14	SEPHS1	selenide, water dikinase 1 isoform 3 [Homo sapiens].
831	NP_036379.2	392	43	5.6	50.8	10p14	SEPHS1	selenide, water dikinase 1 isoform 1 [Homo sapiens].
832	NP_001182531.1	325	35	5.9	56.0	10p14	SEPHS1	selenide, water dikinase 1 isoform 2 [Homo sapiens].
833	NP_005019.2	406	46	6.5	26.6	10p12.2	PIP4K2A	phosphatidylinositol 5-phosphate 4-kinase type-2 alpha [Homo
834	NP_001165774.1	879	97	5.6	30.4	10p12.1	MASTL	serine/threonine-protein kinase greatwall isoform 1 [Homo sapiens].
835	NP_116233.2	878	97	5.6	30.4	10p12.1	MASTL	serine/threonine-protein kinase greatwall isoform 2 [Homo sapiens].
836	NP_001165775.1	840	93	5.7	29.6	10p12.1	MASTL	serine/threonine-protein kinase greatwall isoform 3 [Homo sapiens].
837	NP_005195.2	467	53	5.5	37.5	10p11.23	MAP3K8	mitogen-activated protein kinase kinase kinase 8 [Homo sapiens].
838	NP_001231063.1	467	53	5.5	37.5	10p11.23	MAP3K8	mitogen-activated protein kinase kinase kinase 8 [Homo sapiens].
839	NP_066124.1	1114	124	6.2	35.9	10q11.2	RET	proto-oncogene tyrosine-protein kinase receptor Ret isoform a
840	NP_065681.1	1072	120	6.5	36.5	10q11.2	RET	proto-oncogene tyrosine-protein kinase receptor Ret isoform c
841	NP_620637.1	427	48	6.5	38.6	10q11.22	MAPK8	mitogen-activated protein kinase 8 isoform JNK1 alpha2 [Homo
842	NP_620635.1	427	48	6.3	39.6	10q11.22	MAPK8	mitogen-activated protein kinase 8 isoform JNK1 beta2 [Homo
843	NP_002741.1	384	44	7.5	38.5	10q11.22	MAPK8	mitogen-activated protein kinase 8 isoform JNK1 alpha1 [Homo
844	NP_620634.1	384	44	7.2	39.6	10q11.22	MAPK8	mitogen-activated protein kinase 8 isoform JNK1 beta1 [Homo
845	NP_001091982.1	671	76	5.7	36.1	10q11.2	PRKG1	cGMP-dependent protein kinase 1 isoform 1 [Homo sapiens].
846	NP_006249.1	686	78	5.2	35.1	10q11.2	PRKG1	cGMP-dependent protein kinase 1 isoform 2 [Homo sapiens].
847	NP_001777.1	297	34	8.8	35.0	10q21.1	CDK1	cyclin-dependent kinase 1 isoform 1 [Homo sapiens].
848	NP_203698.1	240	28	6.7	30.8	10q21.1	CDK1	cyclin-dependent kinase 1 isoform 2 [Homo sapiens].
849	NP_001163877.1	109	12	7.6	30.3	10q21.1	CDK1	cyclin-dependent kinase 1 isoform 4 [Homo sapiens].
850	NP_001163878.1	109	12	7.6	30.3	10q21.1	CDK1	cyclin-dependent kinase 1 isoform 4 [Homo sapiens].
851	NP_079406.3	917	103	6.9	41.9	10q22.1	HKDC1	putative hexokinase HKDC1 [Homo sapiens].
852	NP_277033.1	921	103	6.3	41.3	10q22	HK1	hexokinase-1 isoform HKI-ta/tb [Homo sapiens].
853	NP_277032.1	921	103	6.3	41.3	10q22	HK1	hexokinase-1 isoform HKI-ta/tb [Homo sapiens].
854	NP_277035.2	905	101	6.5	41.9	10q22	HK1	hexokinase-1 isoform HKI-td [Homo sapiens].
855	NP_277031.1	916	102	6.2	42.1	10q22	HK1	hexokinase-1 isoform HKI-R [Homo sapiens].
856	NP_000179.2	917	102	6.4	42.0	10q22	HK1	hexokinase-1 isoform HKI [Homo sapiens].
857	NP_001213.2	495	56	6.7	29.3	10q22	CAMK2G	calcium/calmodulin-dependent protein kinase type II subunit gamma
858	NP_751913.1	504	57	6.9	29.0	10q22	CAMK2G	calcium/calmodulin-dependent protein kinase type II subunit gamma
859	NP_751910.1	518	58	6.7	28.4	10q22	CAMK2G	calcium/calmodulin-dependent protein kinase type II subunit gamma
860	NP_751911.1	556	62	7.1	27.0	10q22	CAMK2G	calcium/calmodulin-dependent protein kinase type II subunit gamma
861	NP_001191421.1	539	60	6.9	29.7	10q22	CAMK2G	calcium/calmodulin-dependent protein kinase type II subunit gamma
862	NP_751909.1	527	59	7.5	30.2	10q22	CAMK2G	calcium/calmodulin-dependent protein kinase type II subunit gamma
863	NP_006712.2	362	41	6.2	35.6	10q22	ADK	adenosine kinase isoform b [Homo sapiens].
864	NP_001189379.1	305	34	6.3	31.8	10q22	ADK	adenosine kinase isoform d [Homo sapiens].
865	NP_001114.2	345	39	6.2	37.4	10q22	ADK	adenosine kinase isoform a [Homo sapiens].
866	NP_001189378.1	327	37	6.3	39.4	10q22	ADK	adenosine kinase isoform c [Homo sapiens].
867	NP_004320.2	532	57	7.0	38.1	10q22.3	BMPR1A	bone morphogenetic protein receptor type-1A precursor [Homo
868	NP_001015880.1	619	70	7.9	37.6	10q24	PAPSS2	bifunctional 3'-phosphoadenosine 5'-phosphosulfate synthase 2
869	NP_004661.2	614	70	7.9	36.8	10q24	PAPSS2	bifunctional 3'-phosphoadenosine 5'-phosphosulfate synthase 2
870	NP_683878.1	598	64	7.3	36.1	10q23.31	PANK1	pantothenate kinase 1 isoform alpha [Homo sapiens].
871	NP_612189.2	314	36	7.5	43.0	10q23.31	PANK1	pantothenate kinase 1 isoform gamma [Homo sapiens].
872	NP_683879.1	373	42	5.9	43.7	10q23.31	PANK1	pantothenate kinase 1 isoform beta [Homo sapiens].
873	NP_689522.2	805	90	5.1	28.8	10q24.1	PIK3AP1	phosphoinositide 3-kinase adapter protein 1 [Homo sapiens].
874	NP_060895.1	479	54	8.3	31.7	10q24	PI4K2A	phosphatidylinositol 4-kinase type 2-alpha [Homo sapiens].
875	NP_001269.3	745	85	6.3	38.5	10q24-q25	CHUK	inhibitor of nuclear factor kappa-B kinase subunit alpha [Homo
876	NP_055446.2	1105	122	8.5	18.5	10q24.33	SH3PXD2A	SH3 and PX domain-containing protein 2A [Homo sapiens].
877	NP_055535.2	1235	143	4.9	17.7	10q24.33	SLK	STE20-like serine/threonine-protein kinase [Homo sapiens].
878	NP_005299.1	590	68	8.0	25.9	10q26.11	GRK5	G protein-coupled receptor kinase 5 [Homo sapiens].

879	NP_001098991.1	844	99	5.4	19.3	10q26.3	JAKMIP3	janus kinase and microtubule-interacting protein 3 [Homo sapiens].
880	NP_775846.2	486	55	6.2	31.1	10q26.3	STK32C	serine/threonine-protein kinase 32C [Homo sapiens].
881	NP_689856.6	1749	191	5.8	34.1	10q26.3	KNDC1	protein very KIND isoform a [Homo sapiens].
882	NP_073609.2	715	81	6.4	25.6	11p15.5	EPS8L2	epidermal growth factor receptor kinase substrate 8-like protein 2
883	NP_003948.2	668	75	9.1	31.6	11p15.5	BRSK2	serine/threonine-protein kinase BRSK2 isoform 2 [Homo sapiens].
884	NP_001243556.1	736	82	9.0	30.0	11p15.5	BRSK2	serine/threonine-protein kinase BRSK2 isoform 1 [Homo sapiens].
885	NP_001243558.1	674	75	9.2	31.3	11p15.5	BRSK2	serine/threonine-protein kinase BRSK2 isoform 3 [Homo sapiens].
886	NP_001243559.1	766	85	9.1	29.4	11p15.5	BRSK2	serine/threonine-protein kinase BRSK2 isoform 4 [Homo sapiens].
887	NP_001165694.1	268	30	6.7	36.2	11p15.5	MOB2	MOB kinase activator 2 isoform 1 [Homo sapiens].
888	NP_443731.2	237	27	6.3	34.2	11p15.5	MOB2	MOB kinase activator 2 isoform 2 [Homo sapiens].
889	NP_000067.1	316	32	5.3	41.5	11p15.5	CDKN1C	cyclin-dependent kinase inhibitor 1C isoform a [Homo sapiens].
890	NP_001116102.1	305	31	5.3	42.6	11p15.5	CDKN1C	cyclin-dependent kinase inhibitor 1C isoform b [Homo sapiens].
891	NP_001116103.1	305	31	5.3	42.6	11p15.5	CDKN1C	cyclin-dependent kinase inhibitor 1C isoform b [Homo sapiens].
892	NP_659477.2	261	28	6.0	20.3	11p15.4	PRKCDBP	protein kinase C delta-binding protein [Homo sapiens].
893	NP_112168.1	514	58	6.6	27.0	11p15.3	STK33	serine/threonine-protein kinase 33 [Homo sapiens].
894	NP_003381.1	646	72	6.3	24.0	11p15.3-p1	WEE1	wee1-like protein kinase isoform 1 [Homo sapiens].
895	NP_001137448.1	432	49	7.7	26.4	11p15.3-p1	WEE1	wee1-like protein kinase isoform 2 [Homo sapiens].
896	NP_001193809.1	706	77	5.1	28.2	11p15	MRVI1	protein MRVI1 isoform e [Homo sapiens].
897	NP_569056.4	912	99	5.5	24.1	11p15	MRVI1	protein MRVI1 isoform d [Homo sapiens].
898	NP_001092049.2	904	98	5.4	25.1	11p15	MRVI1	protein MRVI1 isoform a [Homo sapiens].
899	NP_001093633.1	821	90	5.7	22.7	11p15	MRVI1	protein MRVI1 isoform b [Homo sapiens].
900	NP_001093637.1	597	66	5.2	23.6	11p15	MRVI1	protein MRVI1 isoform c [Homo sapiens].
901	NP_001193810.1	597	66	5.2	23.6	11p15	MRVI1	protein MRVI1 isoform c [Homo sapiens].
902	NP_001243615.1	391	45	8.7	30.2	11p15.3	CSNK2A1P	casein kinase 2, alpha 1 polypeptide-like [Homo sapiens].
903	NP_002636.2	1686	191	7.9	37.7	11p15.5-p1	PIK3C2A	phosphatidylinositol 4-phosphate 3-kinase C2 domain-containing
904	NP_006148.2	810	88	5.7	33.4	11p15.1	NELL1	protein kinase C-binding protein NELL1 isoform 1 precursor [Homo
905	NP_963845.1	763	83	5.9	34.3	11p15.1	NELL1	protein kinase C-binding protein NELL1 isoform 2 precursor [Homo
906	NP_005725.3	1215	134	7.1	34.7	11p13	HIPK3	homeodomain-interacting protein kinase 3 isoform 1 [Homo sapiens].
907	NP_001041665.1	1194	131	7.3	34.8	11p13	HIPK3	homeodomain-interacting protein kinase 3 isoform 2 [Homo sapiens].
908	NP_005447.1	711	78	4.7	21.7	11p11.2	MAPK8IP1	C-Jun-amino-terminal kinase-interacting protein 1 [Homo sapiens].
909	NP_963290.1	945	106	7.9	27.2	11p11.2	DGKZ	diacylglycerol kinase zeta isoform 1 [Homo sapiens].
910	NP_963291.2	933	104	7.5	28.1	11p11.2	DGKZ	diacylglycerol kinase zeta isoform 3 [Homo sapiens].
911	NP_001186195.1	934	105	7.9	27.9	11p11.2	DGKZ	diacylglycerol kinase zeta isoform 5 [Homo sapiens].
912	NP_003637.2	929	104	7.9	28.0	11p11.2	DGKZ	diacylglycerol kinase zeta isoform 2 [Homo sapiens].
913	NP_001186197.1	906	101	7.3	28.7	11p11.2	DGKZ	diacylglycerol kinase zeta isoform 7 [Homo sapiens].
914	NP_001186196.1	928	104	7.9	28.0	11p11.2	DGKZ	diacylglycerol kinase zeta isoform 6 [Homo sapiens].
915	NP_001099010.1	1117	124	9.2	29.5	11p11.2	DGKZ	diacylglycerol kinase zeta isoform 4 [Homo sapiens].
916	NP_057307.2	424	48	5.8	12.5	11p12-p11.	PACSIN3	protein kinase C and casein kinase substrate in neurons protein 3
917	NP_001171904.1	424	48	5.8	12.5	11p12-p11.	PACSIN3	protein kinase C and casein kinase substrate in neurons protein 3
918	NP_001171903.1	424	48	5.8	12.5	11p12-p11.	PACSIN3	protein kinase C and casein kinase substrate in neurons protein 3
919	NP_006822.1	425	48	6.2	44.7	11q12	CLP1	polyribonucleotide 5'-hydroxyl-kinase Clp1 isoform 1 [Homo
920	NP_001136069.1	361	41	6.1	45.4	11q12	CLP1	polyribonucleotide 5'-hydroxyl-kinase Clp1 isoform 2 [Homo
921	NP_073741.2	912	98	6.0	37.4	11q12.2	TUT1	speckle targeted PIP5K1A-regulated poly(A) polymerase [Homo
922	NP_001034558.2	788	88	10.1	21.6	11q13.1	MARK2	serine/threonine-protein kinase MARK2 isoform d [Homo sapiens].
923	NP_001156768.1	719	81	10.1	22.3	11q13.1	MARK2	serine/threonine-protein kinase MARK2 isoform e [Homo sapiens].
924	NP_004945.4	724	81	9.9	22.2	11q13.1	MARK2	serine/threonine-protein kinase MARK2 isoform c [Homo sapiens].
925	NP_001156769.1	709	79	10.0	22.4	11q13.1	MARK2	serine/threonine-protein kinase MARK2 isoform f [Homo sapiens].
926	NP_059672.2	745	83	10.0	22.6	11q13.1	MARK2	serine/threonine-protein kinase MARK2 isoform a [Homo sapiens].
927	NP_003933.1	772	86	8.2	34.7	11q11-q13	RPS6KA4	ribosomal protein S6 kinase alpha-4 isoform a [Homo sapiens].
928	NP_001006945.1	766	85	8.4	34.9	11q11-q13	RPS6KA4	ribosomal protein S6 kinase alpha-4 isoform b [Homo sapiens].
929	NP_004570.2	820	92	5.9	39.4	11q13	MAP4K2	mitogen-activated protein kinase kinase kinase kinase 2 [Homo
930	NP_059995.2	1551	172	5.9	30.9	11q13.1	CDC42BPG	serine/threonine-protein kinase MRCK gamma [Homo sapiens].
931	NP_065731.3	808	90	5.9	30.9	11q13	SCYL1	N-terminal kinase-like protein isoform A [Homo sapiens].
932	NP_001041683.1	791	88	5.9	31.2	11q13	SCYL1	N-terminal kinase-like protein isoform B [Homo sapiens].
933	NP_002410.1	847	93	8.0	28.2	11q13.1-q1	MAP3K11	mitogen-activated protein kinase kinase kinase 11 [Homo sapiens].
934	NP_001610.2	689	80	6.9	29.2	11q13.1	ADRBK1	beta-adrenergic receptor kinase 1 [Homo sapiens].
935	NP_003943.2	482	53	7.0	32.8	11q13.2	RPS6KB2	ribosomal protein S6 kinase beta-2 [Homo sapiens].
936	NP_005842.1	126	13	9.8	26.2	11q13	CDK2AP2	cyclin-dependent kinase 2-associated protein 2 [Homo sapiens].
937	NP_997634.1	439	50	6.3	35.5	11q13.2	CHKA	choline kinase alpha isoform b [Homo sapiens].

938	NP_001268.2	457	52	6.1	33.9	11q13.2	CHKA	choline kinase alpha isoform a [Homo sapiens].
939	NP_004696.2	761	88	5.5	37.8	11q13.5	PRKRIR	52 kDa repressor of the inhibitor of the protein kinase [Homo
940	NP_002567.3	545	61	5.5	33.2	11q13-q14	PAK1	serine/threonine-protein kinase PAK 1 isoform 2 [Homo sapiens].
941	NP_001122092.1	553	62	5.2	32.4	11q13-q14	PAK1	serine/threonine-protein kinase PAK 1 isoform 1 [Homo sapiens].
942	NP_000042.3	3056	351	6.4	41.1	11q22-q23	ATM	serine-protein kinase ATM [Homo sapiens].
943	NP_056006.1	926	104	5.6	27.8	11q23.1	SIK2	serine/threonine-protein kinase SIK2 [Homo sapiens].
944	NP_848605.1	765	85	6.6	43.0	11q23.2	ANKK1	ankyrin repeat and protein kinase domain-containing protein 1 [Homo
945	NP_079440.2	1263	140	6.2	27.1	11q23.3	SIK3	serine/threonine-protein kinase SIK3 [Homo sapiens].
946	NP_006167.1	78	8	8.0	25.6	11q24	NRGN	neurogranin [Homo sapiens].
947	NP_001119653.1	78	8	8.0	25.6	11q24	NRGN	neurogranin [Homo sapiens].
948	NP_001107593.1	476	54	8.2	29.6	11q24.2	CHEK1	serine/threonine-protein kinase Chk1 isoform 1 [Homo sapiens].
949	NP_001107594.1	476	54	8.2	29.6	11q24.2	CHEK1	serine/threonine-protein kinase Chk1 isoform 1 [Homo sapiens].
950	NP_001265.2	476	54	8.2	29.6	11q24.2	CHEK1	serine/threonine-protein kinase Chk1 isoform 1 [Homo sapiens].
951	NP_001231775.1	442	50	7.9	28.3	11q24.2	CHEK1	serine/threonine-protein kinase Chk1 isoform 2 [Homo sapiens].
952	NP_998820.3	2634	280	6.0	34.5	12p13.3	WNK1	serine/threonine-protein kinase WNK1 isoform 3 [Homo sapiens].
953	NP_001171914.1	2642	280	5.9	34.4	12p13.3	WNK1	serine/threonine-protein kinase WNK1 isoform 4 [Homo sapiens].
954	NP_061852.3	2382	251	5.9	36.6	12p13.3	WNK1	serine/threonine-protein kinase WNK1 isoform 1 [Homo sapiens].
955	NP_055638.2	2134	226	6.0	33.3	12p13.3	WNK1	serine/threonine-protein kinase WNK1 isoform 2 [Homo sapiens].
956	NP_003836.1	520	60	9.1	32.7	12p13.32	DYRK4	dual specificity tyrosine-phosphorylation-regulated kinase 4 [Homo
957	NP_006413.2	853	95	5.8	30.9	12p13.3	AKAP3	A-kinase anchor protein 3 [Homo sapiens].
958	NP_060893.2	422	48	7.1	48.6	12p13.2	STYK1	tyrosine-protein kinase STYK1 [Homo sapiens].
959	NP_085143.1	665	73	7.2	34.4	12p13	DUSP16	dual specificity protein phosphatase 16 [Homo sapiens].
960	NP_004055.1	198	22	6.6	7.1	12p13.1-p1	CDKN1B	cyclin-dependent kinase inhibitor 1B [Homo sapiens].
961	NP_004438.3	822	92	7.2	25.3	12p12.3	EPS8	epidermal growth factor receptor kinase substrate 8 [Homo sapiens].
962	NP_009109.3	350	38	4.8	34.3	12p12.3	STRAP	serine-threonine kinase receptor-associated protein [Homo sapiens].
963	NP_004561.3	1445	166	6.5	37.8	12p12	PIK3C2G	phosphatidylinositol 4-phosphate 3-kinase C2 domain-containing
964	NP_061108.2	452	51	6.1	41.6	12p12.1	ETNK1	ethanolamine kinase 1 isoform A [Homo sapiens].
965	NP_001034570.1	258	28	8.4	41.9	12p12.1	ETNK1	ethanolamine kinase 1 isoform B [Homo sapiens].
966	NP_055815.1	464	54	6.4	27.6	12p11.23	STK38L	serine/threonine-protein kinase 38-like [Homo sapiens].
967	NP_940980.3	2527	286	6.3	46.7	12q12	LRRK2	leucine-rich repeat serine/threonine-protein kinase 2 [Homo
968	NP_001107654.1	460	52	5.1	36.3	12q12	IRAK4	interleukin-1 receptor-associated kinase 4 isoform a [Homo
969	NP_057207.2	460	52	5.1	36.3	12q12	IRAK4	interleukin-1 receptor-associated kinase 4 isoform a [Homo
970	NP_001138728.1	336	38	4.8	33.9	12q12	IRAK4	interleukin-1 receptor-associated kinase 4 isoform b [Homo
971	NP_001138729.1	336	38	4.8	33.9	12q12	IRAK4	interleukin-1 receptor-associated kinase 4 isoform b [Homo
972	NP_001138730.1	336	38	4.8	33.9	12q12	IRAK4	interleukin-1 receptor-associated kinase 4 isoform b [Homo
973	NP_002813.3	350	40	6.5	22.9	12q12	TWF1	twinfilin-1 isoform 2 [Homo sapiens].
974	NP_001229326.1	357	41	6.3	23.2	12q12	TWF1	twinfilin-1 isoform 1 [Homo sapiens].
975	NP_001138582.1	839	94	5.6	30.3	12q12	NELL2	protein kinase C-binding protein NELL2 isoform d [Homo sapiens].
976	NP_001138579.1	866	97	5.5	30.5	12q12	NELL2	protein kinase C-binding protein NELL2 isoform a [Homo sapiens].
977	NP_006150.1	816	89	5.3	28.1	12q12	NELL2	protein kinase C-binding protein NELL2 isoform b precursor [Homo
978	NP_001138580.1	816	89	5.3	28.1	12q12	NELL2	protein kinase C-binding protein NELL2 isoform b precursor [Homo
979	NP_001138581.1	815	91	5.3	29.8	12q12	NELL2	protein kinase C-binding protein NELL2 isoform c precursor [Homo
980	NP_001160158.1	851	93	7.8	43.0	12q13.3	PFKM	6-phosphofructokinase, muscle type isoform 1 [Homo sapiens].
981	NP_000280.1	780	85	7.9	43.6	12q13.3	PFKM	6-phosphofructokinase, muscle type isoform 2 [Homo sapiens].
982	NP_001160159.1	780	85	7.9	43.6	12q13.3	PFKM	6-phosphofructokinase, muscle type isoform 2 [Homo sapiens].
983	NP_001160160.1	780	85	7.9	43.6	12q13.3	PFKM	6-phosphofructokinase, muscle type isoform 2 [Homo sapiens].
984	NP_002724.1	331	38	6.5	53.5	12q12-q14	PRKAG1	5'-AMP-activated protein kinase subunit gamma-1 isoform 1 [Homo
985	NP_001193638.1	340	39	6.7	54.7	12q12-q14	PRKAG1	5'-AMP-activated protein kinase subunit gamma-1 isoform 3 [Homo
986	NP_001193639.1	299	34	8.7	57.9	12q12-q14	PRKAG1	5'-AMP-activated protein kinase subunit gamma-1 isoform 4 [Homo
987	NP_000011.2	503	54	7.0	37.6	12q13.13	ACVRL1	serine/threonine-protein kinase receptor R3 precursor [Homo
988	NP_001070869.1	503	54	7.0	37.6	12q13.13	ACVRL1	serine/threonine-protein kinase receptor R3 precursor [Homo
989	NP_064733.3	546	59	7.8	39.4	12q13	ACVR1B	activin receptor type-1B isoform c precursor [Homo sapiens].
990	NP_004293.1	505	55	6.8	38.4	12q13	ACVR1B	activin receptor type-1B isoform a precursor [Homo sapiens].
991	NP_064732.3	453	52	7.0	36.4	12q13	ACVR1B	activin receptor type-1B isoform b [Homo sapiens].
992	NP_006292.3	859	93	6.0	26.9	12q13	MAP3K12	mitogen-activated protein kinase kinase kinase 12 isoform 2 [Homo
993	NP_001180440.1	892	96	5.9	27.4	12q13	MAP3K12	mitogen-activated protein kinase kinase kinase 12 isoform 1 [Homo
994	NP_001336.2	735	83	6.3	37.6	12q13.3	DGKA	diacylglycerol kinase alpha [Homo sapiens].
995	NP_958852.1	735	83	6.3	37.6	12q13.3	DGKA	diacylglycerol kinase alpha [Homo sapiens].
996	NP_958853.1	735	83	6.3	37.6	12q13.3	DGKA	diacylglycerol kinase alpha [Homo sapiens].

997	NP_963848.1	735	83	6.3	37.6	12q13.3	DGKA	diacylglycerol kinase alpha [Homo sapiens].
998	NP_001789.2	298	34	9.0	47.7	12q13	CDK2	cyclin-dependent kinase 2 isoform 1 [Homo sapiens].
999	NP_439892.2	264	30	9.5	41.7	12q13	CDK2	cyclin-dependent kinase 2 isoform 2 [Homo sapiens].
1000	NP_001973.2	1342	146	6.1	32.2	12q13	ERBB3	receptor tyrosine-protein kinase erbB-3 isoform 1 precursor [Homo
1001	NP_001005915.1	183	18	4.9	50.0	12q13	ERBB3	receptor tyrosine-protein kinase erbB-3 isoform s precursor [Homo
1002	NP_079055.3	421	47	6.4	30.2	12q13.3	PIP4K2C	phosphatidylinositol 5-phosphate 4-kinase type-2 gamma isoform a
1003	NP_001139730.1	421	47	6.4	30.2	12q13.3	PIP4K2C	phosphatidylinositol 5-phosphate 4-kinase type-2 gamma isoform a
1004	NP_001139732.1	373	42	7.1	32.4	12q13.3	PIP4K2C	phosphatidylinositol 5-phosphate 4-kinase type-2 gamma isoform c
1005	NP_001139731.1	403	45	6.0	31.8	12q13.3	PIP4K2C	phosphatidylinositol 5-phosphate 4-kinase type-2 gamma isoform b
1006	NP_055585.1	836	91	8.9	31.9	12q14.1	AGAP2	arf-GAP with GTPase, ANK repeat and PH domain-containing protein 2
1007	NP_001116244.1	1192	125	10.3	29.5	12q14.1	AGAP2	arf-GAP with GTPase, ANK repeat and PH domain-containing protein 2
1008	NP_000066.1	303	34	6.6	44.2	12q14	CDK4	cyclin-dependent kinase 4 [Homo sapiens].
1009	NP_037386.1	729	84	6.3	35.8	12q14.1	TBK1	serine/threonine-protein kinase TBK1 [Homo sapiens].
1010	NP_009130.2	596	68	6.2	35.1	12q14.3	IRAK3	interleukin-1 receptor-associated kinase 3 isoform a [Homo
1011	NP_001135995.1	535	61	5.9	37.0	12q14.3	IRAK3	interleukin-1 receptor-associated kinase 3 isoform b [Homo
1012	NP_006473.2	601	67	10.0	29.6	12q15	DYRK2	dual specificity tyrosine-phosphorylation-regulated kinase 2
1013	NP_003574.1	528	60	9.8	28.0	12q15	DYRK2	dual specificity tyrosine-phosphorylation-regulated kinase 2
1014	NP_073143.2	235	26	5.4	35.3	12q22-q23	DUSP6	dual specificity protein phosphatase 6 isoform b [Homo sapiens].
1015	NP_001937.2	381	42	4.6	35.2	12q22-q23	DUSP6	dual specificity protein phosphatase 6 isoform a [Homo sapiens].
1016	NP_002586.2	523	60	9.3	23.9	12q23.1	CDK17	cyclin-dependent kinase 17 isoform 1 [Homo sapiens].
1017	NP_001163935.1	523	60	9.0	25.6	12q23.1	CDK17	cyclin-dependent kinase 17 isoform 2 [Homo sapiens].
1018	NP_963906.1	350	39	9.7	25.4	12q23.1	IKBIP	inhibitor of nuclear factor kappa-B kinase-interacting protein
1019	NP_963907.1	70	7	10.6	24.3	12q23.1	IKBIP	inhibitor of nuclear factor kappa-B kinase-interacting protein
1020	NP_710154.1	377	43	4.7	15.4	12q23.1	IKBIP	inhibitor of nuclear factor kappa-B kinase-interacting protein
1021	NP_060458.3	929	104	8.2	39.6	12q23.1	SCYL2	SCY1-like protein 2 [Homo sapiens].
1022	NP_055655.1	661	74	9.0	23.0	12q23.3	NUAK1	NUAK family SNF1-like kinase 1 [Homo sapiens].
1023	NP_000422.1	396	42	6.0	48.0	12q24	MVK	mevalonate kinase [Homo sapiens].
1024	NP_001107657.1	396	42	6.0	48.0	12q24	MVK	mevalonate kinase [Homo sapiens].
1025	NP_620777.1	473	54	7.6	32.3	12q24.13	MAPKAPK5	MAP kinase-activated protein kinase 5 isoform 2 [Homo sapiens].
1026	NP_003659.2	471	54	7.3	32.7	12q24.13	MAPKAPK5	MAP kinase-activated protein kinase 5 isoform 1 [Homo sapiens].
1027	NP_775869.3	921	104	9.0	26.1	12q24.22-q	KSR2	kinase suppressor of Ras 2 [Homo sapiens].
1028	NP_002558.1	187	1	7.1	0.0	12q24.23	PEBP1	phosphatidylethanolamine-binding protein 1 preproprotein [Homo
1029	NP_057365.3	898	105	6.9	19.8	12q	TAOK3	serine/threonine-protein kinase TAO3 [Homo sapiens].
1030	NP_055180.1	196	22	4.9	20.4	12q24.23	HSPB8	heat shock protein beta-8 [Homo sapiens].
1031	NP_006244.2	270	30	5.9	25.2	12q24.1-q2	PRKAB1	5'-AMP-activated protein kinase subunit beta-1 [Homo sapiens].
1032	NP_009105.1	2027	231	6.1	25.7	12q24	CIT	citron Rho-interacting kinase isoform 2 [Homo sapiens].
1033	NP_001193928.1	2069	237	6.2	25.3	12q24	CIT	citron Rho-interacting kinase isoform 1 [Homo sapiens].
1034	NP_001257415.1	556	61	6.4	32.7	12q24.2	CAMKK2	calcium/calmodulin-dependent protein kinase kinase 2 isoform 7
1035	NP_757365.1	545	60	7.4	32.3	12q24.2	CAMKK2	calcium/calmodulin-dependent protein kinase kinase 2 isoform 3
1036	NP_006540.3	588	65	6.2	31.8	12q24.2	CAMKK2	calcium/calmodulin-dependent protein kinase kinase 2 isoform 1
1037	NP_001257414.1	588	65	6.2	31.8	12q24.2	CAMKK2	calcium/calmodulin-dependent protein kinase kinase 2 isoform 1
1038	NP_705720.1	498	55	5.9	32.9	12q24.2	CAMKK2	calcium/calmodulin-dependent protein kinase kinase 2 isoform 4
1039	NP_757380.1	541	60	5.4	32.3	12q24.2	CAMKK2	calcium/calmodulin-dependent protein kinase kinase 2 isoform 2
1040	NP_705719.2	541	60	5.4	32.3	12q24.2	CAMKK2	calcium/calmodulin-dependent protein kinase kinase 2 isoform 2
1041	NP_757364.1	490	54	6.8	33.3	12q24.2	CAMKK2	calcium/calmodulin-dependent protein kinase kinase 2 isoform 6
1042	NP_757363.1	533	59	5.9	32.6	12q24.2	CAMKK2	calcium/calmodulin-dependent protein kinase kinase 2 isoform 5
1043	NP_004633.1	115	12	9.7	24.3	12q24.31	CDK2AP1	cyclin-dependent kinase 2-associated protein 1 isoform 1 [Homo
1044	NP_001257362.1	87	10	9.5	17.2	12q24.31	CDK2AP1	cyclin-dependent kinase 2-associated protein 1 isoform 2 [Homo
1045	NP_001257363.1	87	10	9.5	17.2	12q24.31	CDK2AP1	cyclin-dependent kinase 2-associated protein 1 isoform 2 [Homo
1046	NP_003556.1	1050	113	8.8	35.8	12q24.3	ULK1	serine/threonine-protein kinase ULK1 [Homo sapiens].
1047	NP_055387.2	1088	120	8.1	25.2	13q11-q12	LATS2	serine/threonine-protein kinase LATS2 [Homo sapiens].
1048	NP_001251.1	464	53	8.7	29.7	13q12	CDK8	cyclin-dependent kinase 8 [Homo sapiens].
1049	NP_004110.2	993	113	5.4	39.4	13q12	FLT3	receptor-type tyrosine-protein kinase FLT3 [Homo sapiens].
1050	NP_002010.2	1338	148	8.4	33.3	13q12	FLT1	vascular endothelial growth factor receptor 1 isoform 1 precursor
1051	NP_001153502.1	733	79	9.4	32.4	13q12	FLT1	vascular endothelial growth factor receptor 1 isoform 3 precursor
1052	NP_001153392.1	687	75	9.8	33.1	13q12	FLT1	vascular endothelial growth factor receptor 1 isoform 2 precursor
1053	NP_001153503.1	541	58	9.7	32.8	13q12	FLT1	vascular endothelial growth factor receptor 1 isoform 4 precursor
1054	NP_001182345.1	433	48	5.4	36.3	13q13	DCLK1	serine/threonine-protein kinase DCLK1 isoform 3 [Homo sapiens].
1055	NP_004725.1	729	81	8.8	32.9	13q13	DCLK1	serine/threonine-protein kinase DCLK1 isoform 1 [Homo sapiens].

1056	NP_001182344.1	422	47	5.5	38.6	13q13	DCLK1	serine/threonine-protein kinase DCLK1 isoform 2 [Homo sapiens].
1057	NP_001182359.1	56	6	4.8	5.4	13q13	DCLK1	serine/threonine-protein kinase DCLK1 isoform 4 [Homo sapiens].
1058	NP_660204.2	337	39	9.8	32.0	13q13.3	CSNK1A1L	casein kinase I isoform alpha-like [Homo sapiens].
1059	NP_821077.1	1220	135	6.1	36.5	13q14.11	DGKH	diacylglycerol kinase eta isoform 2 [Homo sapiens].
1060	NP_001191433.1	1164	128	5.9	36.6	13q14.11	DGKH	diacylglycerol kinase eta isoform 1 [Homo sapiens].
1061	NP_690874.2	1164	128	5.9	36.6	13q14.11	DGKH	diacylglycerol kinase eta isoform 1 [Homo sapiens].
1062	NP_001191434.1	1100	122	5.9	36.7	13q14.11	DGKH	diacylglycerol kinase eta isoform 3 [Homo sapiens].
1063	NP_001191435.1	1084	121	5.8	37.2	13q14.11	DGKH	diacylglycerol kinase eta isoform 4 [Homo sapiens].
1064	NP_057332.1	1901	211	5.1	29.8	13q14.11	AKAP11	A-kinase anchor protein 11 [Homo sapiens].
1065	NP_001180407.1	491	56	7.7	32.2	13q14.2	CDADC1	cytidine and dCMP deaminase domain-containing protein 1 isoform 2
1066	NP_112173.1	514	58	7.9	30.7	13q14.2	CDADC1	cytidine and dCMP deaminase domain-containing protein 1 isoform 1
1067	XP_003118937.1	149	17	4.0	12.8	13q14.2	LOC100506	PREDICTED: serine/threonine-protein kinase Nek5-like [Homo
1068	NP_954983.1	708	81	9.0	25.0	13q14.3	NEK5	serine/threonine-protein kinase Nek5 [Homo sapiens].
1069	NP_001139571.1	489	56	6.6	31.7	13q14.13	NEK3	serine/threonine-protein kinase Nek3 isoform b [Homo sapiens].
1070	NP_689933.1	506	58	6.8	31.6	13q14.13	NEK3	serine/threonine-protein kinase Nek3 isoform a [Homo sapiens].
1071	NP_006251.1	504	58	5.8	22.2	13q32.1	DNAJC3	dnaJ homolog subfamily C member 3 precursor [Homo sapiens].
1072	NP_001027467.2	431	48	5.2	31.1	13q31.2-q3	STK24	serine/threonine-protein kinase 24 isoform b [Homo sapiens].
1073	NP_003567.2	443	49	5.4	31.2	13q31.2-q3	STK24	serine/threonine-protein kinase 24 isoform a [Homo sapiens].
1074	NP_004084.1	333	34	8.7	28.4	13q33	EFNB2	ephrin-B2 precursor [Homo sapiens].
1075	NP_001185879.1	1880	209	6.4	34.7	13q33.3	MYO16	unconventional myosin-XVI isoform 1 [Homo sapiens].
1076	NP_055826.1	1858	206	6.4	34.6	13q33.3	MYO16	unconventional myosin-XVI isoform 2 [Homo sapiens].
1077	NP_001229812.1	237	25	6.5	45.6	13q34	CARKD	ATP-dependent (S)-NAD(P)H-hydrate dehydratase isoform d [Homo
1078	NP_001229811.1	329	35	8.6	52.6	13q34	CARKD	ATP-dependent (S)-NAD(P)H-hydrate dehydratase isoform c [Homo
1079	NP_001229810.1	347	37	8.0	57.1	13q34	CARKD	ATP-dependent (S)-NAD(P)H-hydrate dehydratase isoform b [Homo
1080	NP_060680.2	390	41	9.4	49.0	13q34	CARKD	ATP-dependent (S)-NAD(P)H-hydrate dehydratase isoform a [Homo
1081	NP_000811.1	678	72	5.3	34.1	13q34	GAS6	growth arrest-specific protein 6 isoform 1 precursor [Homo
1082	NP_001137417.1	405	45	6.0	46.4	13q34	GAS6	growth arrest-specific protein 6 isoform 2 [Homo sapiens].
1083	NP_001137418.1	379	42	6.0	43.0	13q34	GAS6	growth arrest-specific protein 6 isoform 3 [Homo sapiens].
1084	NP_004554.2	640	67	6.6	35.4	14q11.2	PCK2	phosphoenolpyruvate carboxykinase [GTP], mitochondrial isoform 1
1085	NP_001018083.1	441	44	7.0	40.8	14q11.2	PCK2	phosphoenolpyruvate carboxykinase [GTP], mitochondrial isoform 2
1086	NP_001171668.1	338	38	8.8	38.5	14q12	TSSK4	testis-specific serine/threonine-protein kinase 4 isoform 1 [Homo
1087	NP_777604.2	328	37	8.7	36.9	14q12	TSSK4	testis-specific serine/threonine-protein kinase 4 isoform 2 [Homo
1088	NP_006862.2	518	57	6.1	29.5	14q11.2	RIPK3	receptor-interacting serine/threonine-protein kinase 3 [Homo
1089	NP_002733.2	912	102	6.2	38.0	14q11	PRKD1	serine/threonine-protein kinase D1 [Homo sapiens].
1090	NP_004265.3	2319	257	4.8	20.8	14q12	AKAP6	A-kinase anchor protein 6 [Homo sapiens].
1091	NP_004187.2	358	42	9.0	29.3	14q21.3	CDKL1	cyclin-dependent kinase-like 1 [Homo sapiens].
1092	NP_942089.1	846	95	7.5	36.5	14q11.2-q2	MAP4K5	mitogen-activated protein kinase kinase kinase kinase 5 [Homo
1093	NP_006566.2	846	95	7.5	36.5	14q11.2-q2	MAP4K5	mitogen-activated protein kinase kinase kinase kinase 5 [Homo
1094	NP_065972.3	2133	248	4.9	15.2	14q22.1	NIN	ninein isoform 2 [Homo sapiens].
1095	NP_057434.4	1377	160	5.0	13.4	14q22.1	NIN	ninein isoform 6 [Homo sapiens].
1096	NP_891991.1	2090	243	4.8	14.7	14q22.1	NIN	ninein isoform 5 [Homo sapiens].
1097	NP_891989.2	2046	238	4.8	14.4	14q22.1	NIN	ninein isoform 1 [Homo sapiens].
1098	NP_005183.2	212	24	6.0	36.8	14q22	CDKN3	cyclin-dependent kinase inhibitor 3 isoform 1 [Homo sapiens].
1099	NP_001124323.1	172	19	7.9	32.6	14q22	CDKN3	cyclin-dependent kinase inhibitor 3 isoform 2 [Homo sapiens].
1100	NP_653179.1	245	24	5.2	11.8	14q22.3	MAPK1IP1L	MAPK-interacting and spindle-stabilizing protein-like [Homo
1101	NP_057559.2	311	35	7.6	65.0	14q23.1	JKAMP	JNK1/MAPK8-associated membrane protein isoform 1 [Homo sapiens].
1102	NP_001092095.1	305	35	7.8	64.3	14q23.1	JKAMP	JNK1/MAPK8-associated membrane protein isoform 2 [Homo sapiens].
1103	NP_002422.1	309	36	5.7	24.9	14q23	MNAT1	CDK-activating kinase assembly factor MAT1 isoform 1 [Homo
1104	NP_001171434.1	267	31	5.5	22.8	14q23	MNAT1	CDK-activating kinase assembly factor MAT1 isoform 2 [Homo
1105	NP_006246.2	683	78	7.6	37.5	14q23.1	PRKCH	protein kinase C eta type [Homo sapiens].
1106	NP_004848.3	427	47	4.7	15.7	14q23.3	AKAP5	A-kinase anchor protein 5 [Homo sapiens].
1107	NP_149132.2	1118	123	5.5	31.6	14q24.2	MAP3K9	mitogen-activated protein kinase kinase kinase 9 [Homo sapiens].
1108	NP_149107.4	979	107	5.4	34.8	14q24.3	NEK9	serine/threonine-protein kinase Nek9 [Homo sapiens].
1109	NP_065154.2	523	60	8.6	41.9	14q24.3	ADCK1	uncharacterized aarF domain-containing protein kinase 1 isoform a
1110	NP_001136017.1	455	52	8.8	42.9	14q24.3	ADCK1	uncharacterized aarF domain-containing protein kinase 1 isoform b
1111	NP_004746.2	802	90	6.6	33.4	14q31-q32.	RPS6KA5	ribosomal protein S6 kinase alpha-5 isoform a [Homo sapiens].
1112	NP_872198.1	549	62	6.4	36.8	14q31-q32.	RPS6KA5	ribosomal protein S6 kinase alpha-5 isoform b [Homo sapiens].
1113	NP_001136066.1	314	36	5.4	37.9	14q31	ITPK1	inositol-tetrakisphosphate 1-kinase isoform b [Homo sapiens].
1114	NP_055031.2	414	46	5.7	41.1	14q31	ITPK1	inositol-tetrakisphosphate 1-kinase isoform a [Homo sapiens].

1115	NP_001136065.1	414	46	5.7	41.1	14q31	ITPK1	inositol-tetrakisphosphate 1-kinase isoform a [Homo sapiens].
1116	NP_689540.2	723	83	4.5	36.5	14q32.2	AK7	adenylate kinase 7 [Homo sapiens].
1117	NP_003375.1	396	45	9.2	25.5	14q32	VRK1	serine/threonine-protein kinase VRK1 [Homo sapiens].
1118	NP_116019.1	212	24	5.9	21.2	14q32.31	CINP	cyclin-dependent kinase 2-interacting protein isoform 2 [Homo
1119	NP_001171082.1	227	26	6.1	18.9	14q32.31	CINP	cyclin-dependent kinase 2-interacting protein isoform 1 [Homo
1120	NP_001171083.1	118	13	5.8	23.7	14q32.31	CINP	cyclin-dependent kinase 2-interacting protein isoform 3 [Homo
1121	NP_006026.3	1711	194	5.9	28.3	14q32.3	CDC42BPB	serine/threonine-protein kinase MRCK beta [Homo sapiens].
1122	NP_001122390.1	753	84	9.9	22.7	14q32.3	MARK3	MAP/microtubule affinity-regulating kinase 3 isoform a [Homo
1123	NP_001122391.1	744	83	9.8	22.8	14q32.3	MARK3	MAP/microtubule affinity-regulating kinase 3 isoform b [Homo
1124	NP_002367.4	729	81	9.9	23.3	14q32.3	MARK3	MAP/microtubule affinity-regulating kinase 3 isoform c [Homo
1125	NP_001122392.1	713	80	9.9	23.3	14q32.3	MARK3	MAP/microtubule affinity-regulating kinase 3 isoform d [Homo
1126	NP_001122393.1	659	74	10.2	19.3	14q32.3	MARK3	MAP/microtubule affinity-regulating kinase 3 isoform e [Homo
1127	NP_001814.2	381	43	5.2	33.1	14q32	CKB	creatine kinase B-type [Homo sapiens].
1128	NP_005154.2	480	56	5.7	29.2	14q32.32	AKT1	RAC-alpha serine/threonine-protein kinase [Homo sapiens].
1129	NP_001014431.1	480	56	5.7	29.2	14q32.32	AKT1	RAC-alpha serine/threonine-protein kinase [Homo sapiens].
1130	NP_001014432.1	480	56	5.7	29.2	14q32.32	AKT1	RAC-alpha serine/threonine-protein kinase [Homo sapiens].
1131	NP_001013725.2	1649	187	5.8	35.2	15q15.1	EIF2AK4	eukaryotic translation initiation factor 2-alpha kinase 4 [Homo
1132	NP_001202.4	1050	120	5.0	29.4	15q15	BUB1B	mitotic checkpoint serine/threonine-protein kinase BUB1 beta [Homo
1133	NP_064553.1	681	75	9.9	32.0	15q14	PAK6	serine/threonine-protein kinase PAK 6 [Homo sapiens].
1134	NP_001122100.1	681	75	9.9	32.0	15q14	PAK6	serine/threonine-protein kinase PAK 6 [Homo sapiens].
1135	NP_001122101.1	681	75	9.9	32.0	15q14	PAK6	serine/threonine-protein kinase PAK 6 [Homo sapiens].
1136	NP_002211.1	461	51	7.5	29.3	15q15.1	ITPKA	inositol-trisphosphate 3-kinase A [Homo sapiens].
1137	NP_001129157.1	734	77	6.0	39.1	15q15.1-q2	LTK	leukocyte tyrosine kinase receptor isoform 3 precursor [Homo
1138	NP_996844.1	803	85	6.0	39.9	15q15.1-q2	LTK	leukocyte tyrosine kinase receptor isoform 2 precursor [Homo
1139	NP_002335.2	864	90	6.1	40.1	15q15.1-q2	LTK	leukocyte tyrosine kinase receptor isoform 1 precursor [Homo
1140	NP_006284.2	890	97	5.4	43.1	15q15	TYRO3	tyrosine-protein kinase receptor TYRO3 precursor [Homo sapiens].
1141	NP_001122080.1	1514	164	6.3	33.2	15q15.1	MAPKBP1	mitogen-activated protein kinase-binding protein 1 isoform b [Homo
1142	NP_055809.2	1508	163	6.3	33.2	15q15.1	MAPKBP1	mitogen-activated protein kinase-binding protein 1 isoform a [Homo
1143	NP_001252540.1	1231	134	6.1	34.6	15q15.1	MAPKBP1	mitogen-activated protein kinase-binding protein 1 isoform c [Homo
1144	NP_775771.3	1244	137	6.5	23.0	15q15.2	TTBK2	tau-tubulin kinase 2 [Homo sapiens].
1145	NP_001177143.1	1406	156	5.0	30.4	15q15.3	PPIP5K1	inositol hexakisphosphate and diphosphoinositol-pentakisphosphate
1146	NP_001124331.1	1408	157	5.1	29.5	15q15.3	PPIP5K1	inositol hexakisphosphate and diphosphoinositol-pentakisphosphate
1147	NP_055474.3	1408	157	5.1	29.5	15q15.3	PPIP5K1	inositol hexakisphosphate and diphosphoinositol-pentakisphosphate
1148	NP_001124330.1	1433	160	5.1	29.9	15q15.3	PPIP5K1	inositol hexakisphosphate and diphosphoinositol-pentakisphosphate
1149	NP_066270.1	417	43	7.3	30.2	15q15	CKMT1B	creatine kinase U-type, mitochondrial precursor [Homo sapiens].
1150	NP_001015001.1	417	43	7.3	30.2	15q15	CKMT1A	creatine kinase U-type, mitochondrial precursor [Homo sapiens].
1151	NP_001001556.1	447	49	6.0	46.1	15q21.1-q2	GALK2	N-acetylgalactosamine kinase isoform 2 [Homo sapiens].
1152	NP_002035.1	458	50	6.2	44.3	15q21.1-q2	GALK2	N-acetylgalactosamine kinase isoform 1 [Homo sapiens].
1153	NP_060142.3	1865	213	7.7	38.4	15q21	TRPM7	transient receptor potential cation channel subfamily M member 7
1154	NP_002739.1	721	83	4.8	28.8	15q21	MAPK6	mitogen-activated protein kinase 6 [Homo sapiens].
1155	NP_055141.2	370	43	6.5	32.2	15q22.31	DAPK2	death-associated protein kinase 2 [Homo sapiens].
1156	NP_071331.2	422	49	9.3	26.8	15q22.1-q2	CSNK1G1	casein kinase I isoform gamma-1 [Homo sapiens].
1157	NP_002746.1	393	43	6.2	35.9	15q22.1-q2	MAP2K1	dual specificity mitogen-activated protein kinase kinase 1 [Homo
1158	NP_660143.1	448	50	6.0	43.8	15q23	MAP2K5	dual specificity mitogen-activated protein kinase kinase 5 isoform
1159	NP_002748.1	438	49	5.8	43.4	15q23	MAP2K5	dual specificity mitogen-activated protein kinase kinase 5 isoform
1160	NP_001193733.1	412	46	5.8	40.3	15q23	MAP2K5	dual specificity mitogen-activated protein kinase kinase 5 isoform
1161	NP_001193725.1	605	66	7.7	40.5	15q22	PKM	pyruvate kinase isozymes M1/M2 isoform c [Homo sapiens].
1162	NP_001193728.1	536	59	7.5	43.3	15q22	PKM	pyruvate kinase isozymes M1/M2 isoform f [Homo sapiens].
1163	NP_001193727.1	516	56	8.4	42.1	15q22	PKM	pyruvate kinase isozymes M1/M2 isoform e [Homo sapiens].
1164	NP_001193726.1	457	50	7.7	45.5	15q22	PKM	pyruvate kinase isozymes M1/M2 isoform d [Homo sapiens].
1165	NP_002645.3	531	58	7.7	44.1	15q22	PKM	pyruvate kinase isozymes M1/M2 isoform a [Homo sapiens].
1166	NP_872270.1	531	58	7.5	43.7	15q22	PKM	pyruvate kinase isozymes M1/M2 isoform b [Homo sapiens].
1167	NP_872271.1	531	58	7.5	43.7	15q22	PKM	pyruvate kinase isozymes M1/M2 isoform b [Homo sapiens].
1168	NP_112574.3	496	54	5.8	51.6	15q24.1	ADPGK	ADP-dependent glucokinase precursor [Homo sapiens].
1169	NP_001123500.1	638	74	10.2	23.0	15q24	CLK3	dual specificity protein kinase CLK3 isoform a [Homo sapiens].
1170	NP_003983.2	490	59	9.8	25.7	15q24	CLK3	dual specificity protein kinase CLK3 isoform b [Homo sapiens].
1171	NP_004374.1	450	51	6.6	36.2	15q24.1	CSK	tyrosine-protein kinase CSK [Homo sapiens].
1172	NP_001120662.1	450	51	6.6	36.2	15q24.1	CSK	tyrosine-protein kinase CSK [Homo sapiens].
1173	NP_001092906.1	472	53	7.0	34.3	15q24.1	ULK3	serine/threonine-protein kinase ULK3 [Homo sapiens].

1174	NP_079052.2	1746	193	6.4	21.9	15q24.3	PEAK1	pseudopodium-enriched atypical kinase 1 [Homo sapiens].
1175	NP_006374.1	187	22	4.3	32.6	15q24	CIB2	calcium and integrin-binding family member 2 [Homo sapiens].
1176	NP_001077081.1	220	25	9.3	36.4	15q25.1	AGPHD1	hydroxylysine kinase isoform 2 [Homo sapiens].
1177	NP_001013641.2	373	42	6.4	40.5	15q25.1	AGPHD1	hydroxylysine kinase isoform 1 [Homo sapiens].
1178	NP_065829.3	1907	201	7.4	22.8	15q25.2	ALPK3	alpha-protein kinase 3 [Homo sapiens].
1179	NP_009131.2	2813	308	5.0	27.3	15q24-q25	AKAP13	A-kinase anchor protein 13 isoform 2 [Homo sapiens].
1180	NP_006729.4	2817	308	4.9	27.4	15q24-q25	AKAP13	A-kinase anchor protein 13 isoform 1 [Homo sapiens].
1181	NP_001257475.1	1434	162	6.6	24.3	15q24-q25	AKAP13	A-kinase anchor protein 13 isoform 4 [Homo sapiens].
1182	NP_001230030.1	817	88	6.3	37.4	15q25	NTRK3	NT-3 growth factor receptor isoform d precursor [Homo sapiens].
1183	NP_002521.2	825	89	6.2	37.8	15q25	NTRK3	NT-3 growth factor receptor isoform b precursor [Homo sapiens].
1184	NP_001012338.1	839	91	6.1	38.2	15q25	NTRK3	NT-3 growth factor receptor isoform a precursor [Homo sapiens].
1185	NP_001007157.1	612	65	5.7	33.0	15q25	NTRK3	NT-3 growth factor receptor isoform c precursor [Homo sapiens].
1186	NP_006375.2	191	22	4.5	26.7	15q25.3-q2	CIB1	calcium and integrin-binding protein 1 [Homo sapiens].
1187	NP_001996.1	822	93	6.3	32.7	15q26.1	FES	tyrosine-protein kinase Fes/Fps isoform 1 [Homo sapiens].
1188	NP_001137256.1	752	85	6.4	30.9	15q26.1	FES	tyrosine-protein kinase Fes/Fps isoform 3 [Homo sapiens].
1189	NP_001137255.1	764	87	6.2	34.3	15q26.1	FES	tyrosine-protein kinase Fes/Fps isoform 2 [Homo sapiens].
1190	NP_001137257.1	694	79	6.3	32.4	15q26.1	FES	tyrosine-protein kinase Fes/Fps isoform 4 [Homo sapiens].
1191	NP_078928.3	2015	225	6.2	41.4	15q26.3	LRRK1	leucine-rich repeat serine/threonine-protein kinase 1 [Homo
1192	NP_005000.1	187	17	9.6	37.7	16p13.3	NME4	nucleoside diphosphate kinase, mitochondrial precursor [Homo
1193	NP_055948.2	1336	147	5.1	27.7	16p13.3	MAPK8IP3	C-Jun-amino-terminal kinase-interacting protein 3 isoform 1 [Homo
1194	NP_001035529.1	1330	147	5.2	27.7	16p13.3	MAPK8IP3	C-Jun-amino-terminal kinase-interacting protein 3 isoform 2 [Homo
1195	NP_002504.2	169	19	7.8	45.0	16q13.3	NME3	nucleoside diphosphate kinase 3 precursor [Homo sapiens].
1196	NP_001123484.1	337	37	7.9	12.2	16p13.3	SLC9A3R2	Na(+)/H(+) exchange regulatory cofactor NHE-RF2 isoform a [Homo
1197	NP_004776.3	326	36	8.2	12.6	16p13.3	SLC9A3R2	Na(+)/H(+) exchange regulatory cofactor NHE-RF2 isoform b [Homo
1198	NP_001239002.1	226	25	9.8	9.3	16p13.3	SLC9A3R2	Na(+)/H(+) exchange regulatory cofactor NHE-RF2 isoform c [Homo
1199	NP_001239005.1	215	23	10.0	9.8	16p13.3	SLC9A3R2	Na(+)/H(+) exchange regulatory cofactor NHE-RF2 isoform e [Homo
1200	NP_001239004.1	224	24	9.8	8.9	16p13.3	SLC9A3R2	Na(+)/H(+) exchange regulatory cofactor NHE-RF2 isoform d [Homo
1201	NP_002604.1	556	63	7.0	25.7	16p13.3	PDPK1	3-phosphoinositide-dependent protein kinase 1 isoform 1 [Homo
1202	NP_112558.2	429	48	6.6	25.6	16p13.3	PDPK1	3-phosphoinositide-dependent protein kinase 1 isoform 2 [Homo
1203	NP_001248745.1	454	51	5.7	28.6	16p13.3	PDPK1	3-phosphoinositide-dependent protein kinase 1 isoform 3 [Homo
1204	NP_001245379.1	430	47	5.7	37.0	16p13.3	PKMYT1	membrane-associated tyrosine- and threonine-specific
1205	NP_004194.3	499	55	6.5	35.9	16p13.3	PKMYT1	membrane-associated tyrosine- and threonine-specific
1206	NP_001245380.1	490	54	6.5	35.1	16p13.3	PKMYT1	membrane-associated tyrosine- and threonine-specific
1207	NP_872629.1	480	52	7.5	36.7	16p13.3	PKMYT1	membrane-associated tyrosine- and threonine-specific
1208	NP_055907.3	3661	411	6.0	41.3	16p12.3	SMG1	serine/threonine-protein kinase SMG1 [Homo sapiens].
1209	NP_569731.2	1133	126	10.4	19.5	16p12.2	NPIPL3	putative NPIP-like protein KIAA0220-like [Homo sapiens].
1210	NP_037434.1	725	82	5.1	20.3	16p12.2	EEF2K	eukaryotic elongation factor 2 kinase [Homo sapiens].
1211	NP_005021.2	603	68	9.1	35.5	16p12.2	PLK1	serine/threonine-protein kinase PLK1 [Homo sapiens].
1212	NP_997700.1	671	77	6.6	27.4	16p11.2	PRKCB	protein kinase C beta type isoform 1 [Homo sapiens].
1213	NP_002729.2	673	77	6.6	27.3	16p11.2	PRKCB	protein kinase C beta type isoform 2 [Homo sapiens].
1214	NP_001019572.1	424	46	9.3	38.0	16p11.2	SBK1	serine/threonine-protein kinase SBK1 [Homo sapiens].
1215	NP_004774.1	1049	119	7.1	21.2	16p11.2	TAOK2	serine/threonine-protein kinase TAO2 isoform 2 [Homo sapiens].
1216	NP_057235.2	1235	138	6.8	29.2	16p11.2	TAOK2	serine/threonine-protein kinase TAO2 isoform 1 [Homo sapiens].
1217	NP_001238972.1	1122	126	8.1	30.4	16p11.2	TAOK2	serine/threonine-protein kinase TAO2 isoform 3 [Homo sapiens].
1218	NP_001103361.1	335	38	5.8	36.4	16p11.2	MAPK3	mitogen-activated protein kinase 3 isoform 3 [Homo sapiens].
1219	NP_002737.2	379	43	6.3	34.3	16p11.2	MAPK3	mitogen-activated protein kinase 3 isoform 1 [Homo sapiens].
1220	NP_001035145.1	357	40	7.3	35.9	16p11.2	MAPK3	mitogen-activated protein kinase 3 isoform 2 [Homo sapiens].
1221	NP_036380.2	448	47	5.6	54.7	16p11.2	SEPHS2	selenide, water dikinase 2 [Homo sapiens].
1222	NP_001165903.1	374	43	6.8	33.4	16p11.2	PHKG2	phosphorylase b kinase gamma catalytic chain, testis/liver isoform
1223	NP_000285.1	406	46	5.9	32.3	16p11.2	PHKG2	phosphorylase b kinase gamma catalytic chain, testis/liver isoform
1224	NP_872299.2	819	88	5.7	28.6	16q11.2	MYLK3	myosin light chain kinase 3 [Homo sapiens].
1225	NP_000284.1	1093	125	6.5	38.0	16q12-q13	PHKB	phosphorylase b kinase regulatory subunit beta isoform a [Homo
1226	NP_001027005.1	1086	124	6.2	39.0	16q12-q13	PHKB	phosphorylase b kinase regulatory subunit beta isoform b [Homo
1227	NP_060580.2	326	37	8.0	22.7	16q21	DOK4	docking protein 4 [Homo sapiens].
1228	NP_001887.1	350	41	8.8	25.4	16q21	CSNK2A2	casein kinase II subunit alpha' [Homo sapiens].
1229	NP_001166114.1	234	28	6.4	33.8	16q22-q23.	TK2	thymidine kinase 2, mitochondrial isoform 2 precursor [Homo
1230	NP_001166115.1	240	25	8.0	30.9	16q22-q23.	TK2	thymidine kinase 2, mitochondrial isoform 3 precursor [Homo
1231	NP_001166116.1	247	25	6.2	33.6	16q22-q23.	TK2	thymidine kinase 2, mitochondrial isoform 4 precursor [Homo
1232	NP_004605.4	265	27	6.6	34.1	16q22-q23.	TK2	thymidine kinase 2, mitochondrial isoform 1 precursor [Homo

1233	NP_006733.1	424	48	10.1	30.9	16q22.1	PSKH1	serine/threonine-protein kinase H1 [Homo sapiens].
1234	NP_001135969.1	263	30	9.5	17.1	16q23.1	MLKL	mixed lineage kinase domain-like protein isoform 2 [Homo sapiens].
1235	NP_689862.1	471	54	9.0	27.2	16q23.1	MLKL	mixed lineage kinase domain-like protein isoform 1 [Homo sapiens].
1236	NP_443714.3	360	41	9.0	41.9	16q24	CDK10	cyclin-dependent kinase 10 isoform a [Homo sapiens].
1237	NP_443713.2	272	31	8.3	52.9	16q24	CDK10	cyclin-dependent kinase 10 isoform b [Homo sapiens].
1238	NP_001153839.1	289	33	8.9	48.8	16q24	CDK10	cyclin-dependent kinase 10 isoform d [Homo sapiens].
1239	NP_001092003.2	283	32	8.6	51.6	16q24	CDK10	cyclin-dependent kinase 10 isoform c [Homo sapiens].
1240	NP_006752.1	255	29	4.5	33.3	17p13.3	YWHAE	14-3-3 protein epsilon [Homo sapiens].
1241	NP_114171.2	798	88	9.4	31.1	17p13	GSG2	serine/threonine-protein kinase haspin [Homo sapiens].
1242	NP_115670.1	505	56	5.5	32.9	17p13.2	CAMKK1	calcium/calmodulin-dependent protein kinase kinase 1 isoform a
1243	NP_757343.1	505	56	5.5	32.9	17p13.2	CAMKK1	calcium/calmodulin-dependent protein kinase kinase 1 isoform a
1244	NP_757344.2	520	58	5.8	31.7	17p13.2	CAMKK1	calcium/calmodulin-dependent protein kinase kinase 1 isoform b
1245	NP_722549.2	1332	150	7.7	22.8	17p13.2	MINK1	misshapen-like kinase 1 isoform 3 [Homo sapiens].
1246	NP_733763.1	1303	147	7.1	23.2	17p13.2	MINK1	misshapen-like kinase 1 isoform 2 [Homo sapiens].
1247	NP_056531.1	1295	146	7.0	23.5	17p13.2	MINK1	misshapen-like kinase 1 isoform 1 [Homo sapiens].
1248	NP_001020108.1	1312	148	7.2	22.8	17p13.2	MINK1	misshapen-like kinase 1 isoform 4 [Homo sapiens].
1249	NP_001238831.1	666	72	9.8	40.4	17p13.1	TNK1	non-receptor tyrosine-protein kinase TNK1 isoform 1 [Homo sapiens].
1250	NP_003976.2	661	72	9.8	40.4	17p13.1	TNK1	non-receptor tyrosine-protein kinase TNK1 isoform 2 [Homo sapiens].
1251	NP_001397.1	340	33	8.5	30.0	17p13.1	EFNB3	ephrin-B3 precursor [Homo sapiens].
1252	NP_004208.2	344	39	9.6	30.2	17p13.1	AURKB	aurora kinase B isoform 1 [Homo sapiens].
1253	NP_001243763.1	303	35	9.3	30.0	17p13.1	AURKB	aurora kinase B isoform 2 [Homo sapiens].
1254	NP_001010855.1	754	84	7.4	41.8	17p13.1	PIK3R6	phosphoinositide 3-kinase regulatory subunit 6 [Homo sapiens].
1255	NP_055123.2	880	97	6.3	38.1	17p13.1	PIK3R5	phosphoinositide 3-kinase regulatory subunit 5 isoform 1 [Homo
1256	NP_001136105.1	880	97	6.3	38.1	17p13.1	PIK3R5	phosphoinositide 3-kinase regulatory subunit 5 isoform 1 [Homo
1257	NP_001238781.1	494	55	9.5	32.8	17p13.1	PIK3R5	phosphoinositide 3-kinase regulatory subunit 5 isoform 2 [Homo
1258	NP_001238780.1	494	55	9.5	32.8	17p13.1	PIK3R5	phosphoinositide 3-kinase regulatory subunit 5 isoform 2 [Homo
1259	NP_001238782.1	494	55	9.5	32.8	17p13.1	PIK3R5	phosphoinositide 3-kinase regulatory subunit 5 isoform 2 [Homo
1260	NP_001238784.1	494	55	9.5	32.8	17p13.1	PIK3R5	phosphoinositide 3-kinase regulatory subunit 5 isoform 2 [Homo
1261	NP_003001.1	399	44	8.0	28.8	17p12	MAP2K4	dual specificity mitogen-activated protein kinase kinase 4 [Homo
1262	NP_620602.2	816	88	5.5	30.6	17p11.2	MAPK7	mitogen-activated protein kinase 7 isoform 1 [Homo sapiens].
1263	NP_620603.2	816	88	5.5	30.6	17p11.2	MAPK7	mitogen-activated protein kinase 7 isoform 1 [Homo sapiens].
1264	NP_002740.2	816	88	5.5	30.6	17p11.2	MAPK7	mitogen-activated protein kinase 7 isoform 1 [Homo sapiens].
1265	NP_620601.1	677	73	5.4	28.8	17p11.2	MAPK7	mitogen-activated protein kinase 7 isoform 2 [Homo sapiens].
1266	NP_055498.3	1036	113	8.4	34.7	17p11.2	ULK2	serine/threonine-protein kinase ULK2 [Homo sapiens].
1267	NP_001136082.1	1036	113	8.4	34.7	17p11.2	ULK2	serine/threonine-protein kinase ULK2 [Homo sapiens].
1268	NP_009133.2	662	71	5.6	30.6	17q11.1	AKAP10	A-kinase anchor protein 10, mitochondrial precursor [Homo sapiens].
1269	NP_659731.1	347	39	7.2	35.4	17q11.2	MAP2K3	dual specificity mitogen-activated protein kinase kinase 3 isoform
1270	NP_002747.2	318	36	5.8	38.1	17q11.2	MAP2K3	dual specificity mitogen-activated protein kinase kinase 3 isoform
1271	NP_055053.1	762	85	8.6	27.4	17q11.2	KSR1	kinase suppressor of Ras 1 [Homo sapiens].
1272	NP_057315.3	527	58	8.0	41.6	17q11.2	NLK	serine/threonine-protein kinase NLK [Homo sapiens].
1273	NP_001167574.1	410	46	6.9	41.2	17q11.2	SGK494	uncharacterized serine/threonine-protein kinase SgK494 [Homo
1274	NP_835464.1	692	75	7.6	49.3	17q11.1	NEK8	serine/threonine-protein kinase Nek8 [Homo sapiens].
1275	NP_065842.1	1001	116	7.3	17.0	17q11.2	TAOK1	serine/threonine-protein kinase TAO1 isoform 1 [Homo sapiens].
1276	NP_079418.1	853	98	6.5	19.2	17q11.2	TAOK1	serine/threonine-protein kinase TAO1 isoform 2 [Homo sapiens].
1277	NP_054749.2	761	84	6.3	26.8	17p11.2	GIT1	ARF GTPase-activating protein GIT1 isoform 2 [Homo sapiens].
1278	NP_001078923.1	770	85	6.6	26.2	17p11.2	GIT1	ARF GTPase-activating protein GIT1 isoform 1 [Homo sapiens].
1279	NP_003876.1	307	34	9.7	37.5	17q11.2	CDK5R1	cyclin-dependent kinase 5 activator 1 [Homo sapiens].
1280	NP_008957.1	198	22	9.8	49.5	17q12	DUSP14	dual specificity protein phosphatase 14 [Homo sapiens].
1281	NP_079524.2	1183	127	9.7	19.9	17q12	SRCIN1	SRC kinase signaling inhibitor 1 [Homo sapiens].
1282	NP_003550.1	416	47	7.0	28.8	17q12	PIP4K2B	phosphatidylinositol 5-phosphate 4-kinase type-2 beta [Homo
1283	NP_057591.2	1490	164	9.8	20.6	17q12	CDK12	cyclin-dependent kinase 12 isoform 1 [Homo sapiens].
1284	NP_055898.1	1481	163	9.8	20.5	17q12	CDK12	cyclin-dependent kinase 12 isoform 2 [Homo sapiens].
1285	NP_004439.2	1255	138	5.5	36.7	17q12	ERBB2	receptor tyrosine-protein kinase erbB-2 isoform a precursor [Homo
1286	NP_001005862.1	1225	135	5.6	35.9	17q12	ERBB2	receptor tyrosine-protein kinase erbB-2 isoform b [Homo sapiens].
1287	NP_115763.2	1243	135	5.2	24.7	17q21-q22	WNK4	serine/threonine-protein kinase WNK4 [Homo sapiens].
1288	NP_079095.3	231	27	9.9	46.8	17q21.31	DCAKD	dephospho-CoA kinase domain-containing protein [Homo sapiens].
1289	NP_001122103.1	231	27	9.9	46.8	17q21.31	DCAKD	dephospho-CoA kinase domain-containing protein [Homo sapiens].
1290	NP_003945.2	947	104	7.5	30.4	17q21	MAP3K14	mitogen-activated protein kinase kinase kinase 14 [Homo sapiens].
1291	NP_055541.1	615	68	5.5	18.5	17q21.32	TBKBP1	TANK-binding kinase 1-binding protein 1 [Homo sapiens].

1292	NP_002602.2	407	46	6.1	38.6	17q21.33	PDK2	pyruvate dehydrogenase kinase, isozyme 2 isoform 1 [Homo sapiens].
1293	NP_001186829.1	199	22	8.7	37.2	17q21.33	PDK2	pyruvate dehydrogenase kinase, isozyme 2 isoform 3 [Homo sapiens].
1294	NP_001186827.1	343	39	5.5	37.9	17q21.33	PDK2	pyruvate dehydrogenase kinase, isozyme 2 isoform 2 [Homo sapiens].
1295	NP_001186828.1	343	39	5.5	37.9	17q21.33	PDK2	pyruvate dehydrogenase kinase, isozyme 2 isoform 2 [Homo sapiens].
1296	NP_003962.3	1307	145	4.9	27.1	17q21.33	SPAG9	C-Jun-amino-terminal kinase-interacting protein 4 isoform 3 [Homo
1297	NP_001123999.1	1311	145	4.9	26.9	17q21.33	SPAG9	C-Jun-amino-terminal kinase-interacting protein 4 isoform 2 [Homo
1298	NP_001124000.1	1321	146	4.9	26.8	17q21.33	SPAG9	C-Jun-amino-terminal kinase-interacting protein 4 isoform 1 [Homo
1299	NP_001238900.1	1177	129	5.2	32.3	17q21.33	SPAG9	C-Jun-amino-terminal kinase-interacting protein 4 isoform 4 [Homo
1300	NP_937818.1	177	20	5.3	39.5	17q21.3	NME1	nucleoside diphosphate kinase A isoform a [Homo sapiens].
1301	NP_000260.1	152	17	5.8	38.8	17q21.3	NME1	nucleoside diphosphate kinase A isoform b [Homo sapiens].
1302	NP_001018147.1	152	17	8.7	35.5	17q21.3	NME2	nucleoside diphosphate kinase B isoform a [Homo sapiens].
1303	NP_002503.1	152	17	8.7	35.5	17q21.3	NME2	nucleoside diphosphate kinase B isoform a [Homo sapiens].
1304	NP_001018149.1	152	17	8.7	35.5	17q21.3	NME2	nucleoside diphosphate kinase B isoform a [Homo sapiens].
1305	NP_001018148.1	152	17	8.7	35.5	17q21.3	NME2	nucleoside diphosphate kinase B isoform a [Homo sapiens].
1306	NP_001185611.1	82	10	10.1	37.8	17q21.3	NME2	nucleoside diphosphate kinase B isoform b [Homo sapiens].
1307	NP_003638.1	567	64	7.6	43.4	17q22	DGKE	diacylglycerol kinase epsilon [Homo sapiens].
1308	NP_003479.1	903	94	4.6	36.3	17q22	AKAP1	A-kinase anchor protein 1, mitochondrial precursor [Homo sapiens].
1309	NP_001229831.1	903	97	4.7	37.3	17q22	AKAP1	A-kinase anchor protein 1, mitochondrial precursor [Homo sapiens].
1310	NP_001229832.1	903	97	4.7	37.3	17q22	AKAP1	A-kinase anchor protein 1, mitochondrial precursor [Homo sapiens].
1311	NP_112562.3	1451	163	5.0	27.4	17q22	TEX14	inactive serine/threonine-protein kinase TEX14 isoform b [Homo
1312	NP_938207.2	1491	167	4.9	26.8	17q22	TEX14	inactive serine/threonine-protein kinase TEX14 isoform a [Homo
1313	NP_001188386.1	1497	168	4.9	26.9	17q22	TEX14	inactive serine/threonine-protein kinase TEX14 isoform c [Homo
1314	NP_055721.3	755	84	4.8	29.3	17q22	PPM1E	protein phosphatase 1E [Homo sapiens].
1315	NP_003152.1	525	59	6.2	26.9	17q23.1	RPS6KB1	ribosomal protein S6 kinase beta-1 [Homo sapiens].
1316	NP_006843.2	750	85	8.4	21.5	17q23	TLK2	serine/threonine-protein kinase tousled-like 2 isoform A [Homo
1317	NP_001106178.1	718	82	8.2	21.7	17q23	TLK2	serine/threonine-protein kinase tousled-like 2 isoform B [Homo
1318	NP_976226.1	657	74	9.3	21.0	17q23.3	MAP3K3	mitogen-activated protein kinase kinase kinase 3 isoform 1 [Homo
1319	NP_002392.2	626	71	9.0	20.1	17q23.3	MAP3K3	mitogen-activated protein kinase kinase kinase 3 isoform 2 [Homo
1320	NP_001003786.1	394	44	5.8	40.9	17q23.3	STRADA	STE20-related kinase adapter protein alpha isoform 2 [Homo
1321	NP_001003787.1	431	48	6.0	39.4	17q23.3	STRADA	STE20-related kinase adapter protein alpha isoform 1 [Homo
1322	NP_001003788.1	373	42	6.0	41.0	17q23.3	STRADA	STE20-related kinase adapter protein alpha isoform 4 [Homo
1323	NP_001159441.1	314	35	6.2	44.9	17q23.3	STRADA	STE20-related kinase adapter protein alpha isoform 5 [Homo
1324	NP_001159442.1	299	33	6.9	43.8	17q23.3	STRADA	STE20-related kinase adapter protein alpha isoform 6 [Homo
1325	NP_699166.2	348	39	6.3	40.8	17q23.3	STRADA	STE20-related kinase adapter protein alpha isoform 3 [Homo
1326	NP_002728.1	672	77	6.6	28.3	17q22-q23.	PRKCA	protein kinase C alpha type [Homo sapiens].
1327	NP_002725.1	381	43	5.1	28.1	17q23-q24	PRKAR1A	cAMP-dependent protein kinase type I-alpha regulatory subunit [Homo
1328	NP_997636.1	381	43	5.1	28.1	17q23-q24	PRKAR1A	cAMP-dependent protein kinase type I-alpha regulatory subunit [Homo
1329	NP_997637.1	381	43	5.1	28.1	17q23-q24	PRKAR1A	cAMP-dependent protein kinase type I-alpha regulatory subunit [Homo
1330	NP_002749.2	334	37	7.2	38.9	17q24.3	MAP2K6	dual specificity mitogen-activated protein kinase kinase 6 [Homo
1331	NP_001249.1	305	35	9.1	41.3	17q25.1	CDK3	cyclin-dependent kinase 3 [Homo sapiens].
1332	NP_892010.2	470	51	6.7	53.0	17q25.2	SPHK1	sphingosine kinase 1 isoform 2 [Homo sapiens].
1333	NP_068807.2	398	44	6.9	59.3	17q25.2	SPHK1	sphingosine kinase 1 isoform 1 [Homo sapiens].
1334	NP_001136073.1	384	43	6.7	57.3	17q25.2	SPHK1	sphingosine kinase 1 isoform 3 [Homo sapiens].
1335	NP_001136074.1	384	43	6.7	57.3	17q25.2	SPHK1	sphingosine kinase 1 isoform 3 [Homo sapiens].
1336	NP_003249.3	234	25	8.5	46.2	17q23.2-q2	TK1	thymidine kinase, cytosolic [Homo sapiens].
1337	NP_001073864.2	1374	145	4.3	32.4	17q25.3	AATK	serine/threonine-protein kinase LMTK1 isoform 1 [Homo sapiens].
1338	NP_004911.2	1271	134	4.4	30.8	17q25.3	AATK	serine/threonine-protein kinase LMTK1 isoform 2 [Homo sapiens].
1339	NP_004703.1	777	86	5.8	20.6	17q25	HGS	hepatocyte growth factor-regulated tyrosine kinase substrate [Homo
1340	NP_001884.2	415	47	10.1	27.2	17q25	CSNK1D	casein kinase I isoform delta isoform 1 [Homo sapiens].
1341	NP_620693.1	409	47	10.0	26.4	17q25	CSNK1D	casein kinase I isoform delta isoform 2 [Homo sapiens].
1342	NP_005424.1	543	61	6.3	32.0	18p11.31-p	YES1	tyrosine-protein kinase Yes [Homo sapiens].
1343	NP_005397.1	1354	158	5.6	18.6	18q11.1	ROCK1	rho-associated protein kinase 1 [Homo sapiens].
1344	NP_003822.2	519	59	5.4	26.6	18q11.2	RIOK3	serine/threonine-protein kinase RIO3 [Homo sapiens].
1345	NP_060640.2	312	36	7.3	25.6	18q12.2	RPRD1A	regulation of nuclear pre-mRNA domain-containing protein 1A [Homo
1346	NP_002638.2	887	102	6.4	32.8	18q12.3	PIK3C3	phosphatidylinositol 3-kinase catalytic subunit type 3 [Homo
1347	NP_002738.2	587	66	5.1	33.0	18q21.1	MAPK4	mitogen-activated protein kinase 4 [Homo sapiens].
1348	NP_443179.3	2170	237	5.0	23.6	18q21.31	ALPK2	alpha-protein kinase 2 [Homo sapiens].
1349	NP_002566.1	415	47	5.4	36.9	18q21.3	SERPINB2	plasminogen activator inhibitor 2 [Homo sapiens].
1350	NP_001137290.1	415	47	5.4	36.9	18q21.3	SERPINB2	plasminogen activator inhibitor 2 [Homo sapiens].

1351	NP_689934.2	331	38	8.5	22.1	18q22.2	DOK6	docking protein 6 [Homo sapiens].
1352	NP_000446.1	433	49	7.2	31.4	19p13.3	STK11	serine/threonine-protein kinase STK11 [Homo sapiens].
1353	NP_001396.2	213	19	6.3	15.9	19p13.3	EFNA2	ephrin-A2 precursor [Homo sapiens].
1354	NP_001230008.1	336	36	6.0	33.6	19p13.3	PLK5	inactive serine/threonine-protein kinase PLK5 [Homo sapiens].
1355	NP_001310.3	415	47	9.3	25.1	19p13.3	CSNK1G2	casein kinase I isoform gamma-2 [Homo sapiens].
1356	NP_060042.2	414	47	6.0	31.4	19p13.3	MKNK2	MAP kinase-interacting serine/threonine-protein kinase 2 isoform 1
1357	NP_951009.1	465	52	5.8	31.8	19p13.3	MKNK2	MAP kinase-interacting serine/threonine-protein kinase 2 isoform 2
1358	NP_570719.1	217	25	8.8	31.3	19p13.3	MOB3A	MOB kinase activator 3A [Homo sapiens].
1359	NP_001182662.1	640	70	5.2	34.2	19p13.3	PIP5K1C	phosphatidylinositol 4-phosphate 5-kinase type-1 gamma isoform 1
1360	NP_036530.1	668	73	5.0	33.1	19p13.3	PIP5K1C	phosphatidylinositol 4-phosphate 5-kinase type-1 gamma isoform 2
1361	NP_002369.2	508	57	9.5	35.0	19p13.3	MATK	megakaryocyte-associated tyrosine-protein kinase isoform b [Homo
1362	NP_647612.1	507	56	8.9	35.7	19p13.3	MATK	megakaryocyte-associated tyrosine-protein kinase isoform a [Homo
1363	NP_647611.1	466	52	8.6	37.1	19p13.3	MATK	megakaryocyte-associated tyrosine-protein kinase isoform c [Homo
1364	NP_733778.1	230	26	5.7	28.3	19p13.3	NMRK2	nicotinamide riboside kinase 2 [Homo sapiens].
1365	NP_001339.1	454	53	6.5	26.7	19p13.3	DAPK3	death-associated protein kinase 3 [Homo sapiens].
1366	NP_109587.1	400	44	6.1	34.0	19p13.3	MAP2K2	dual specificity mitogen-activated protein kinase kinase 2 [Homo
1367	NP_001013863.1	403	45	8.2	29.8	19p13.3	STAP2	signal-transducing adaptor protein 2 isoform 2 [Homo sapiens].
1368	NP_060190.2	449	50	7.7	30.1	19p13.3	STAP2	signal-transducing adaptor protein 2 isoform 1 [Homo sapiens].
1369	XP_003403529.3	118	13	8.1	32.2	19p13.3	LOC390877	PREDICTED: adenylate kinase isoenzyme 1-like [Homo sapiens].
1370	NP_660186.1	419	47	9.5	30.5	19p13.3-p1	MAP2K7	dual specificity mitogen-activated protein kinase kinase 7 [Homo
1371	NP_003322.3	1187	134	6.7	36.1	19p13.2	TYK2	non-receptor tyrosine-protein kinase TYK2 [Homo sapiens].
1372	NP_524145.1	166	18	5.7	40.4	19p13	CDKN2D	cyclin-dependent kinase 4 inhibitor D [Homo sapiens].
1373	NP_001791.1	166	18	5.7	40.4	19p13	CDKN2D	cyclin-dependent kinase 4 inhibitor D [Homo sapiens].
1374	NP_001001329.1	525	59	4.2	14.1	19p13.2	PRKCSH	glucosidase 2 subunit beta isoform 2 precursor [Homo sapiens].
1375	NP_002734.2	528	59	4.2	14.0	19p13.2	PRKCSH	glucosidase 2 subunit beta isoform 1 precursor [Homo sapiens].
1376	NP_115708.1	315	34	5.3	33.0	19p13.2	WDR83	WD repeat domain-containing protein 83 [Homo sapiens].
1377	NP_001093207.1	315	34	5.3	33.0	19p13.2	WDR83	WD repeat domain-containing protein 83 [Homo sapiens].
1378	NP_055790.1	1570	171	8.4	26.7	19p13.2	MAST1	microtubule-associated serine/threonine-protein kinase 1 [Homo
1379	NP_002721.1	351	41	9.1	25.4	19p13.1	PRKACA	cAMP-dependent protein kinase catalytic subunit alpha isoform 1
1380	NP_997401.1	343	40	9.0	25.9	19p13.1	PRKACA	cAMP-dependent protein kinase catalytic subunit alpha isoform 2
1381	NP_002732.3	942	104	6.0	32.4	19p13.12	PKN1	serine/threonine-protein kinase N1 isoform 2 [Homo sapiens].
1382	NP_998725.1	948	105	5.8	32.2	19p13.12	PKN1	serine/threonine-protein kinase N1 isoform 1 [Homo sapiens].
1383	NP_005849.1	692	76	4.9	14.0	19p13.1	AKAP8	A-kinase anchor protein 8 [Homo sapiens].
1384	NP_055186.2	646	72	4.8	14.6	19p13.12	AKAP8L	A-kinase anchor protein 8-like [Homo sapiens].
1385	NP_473454.1	187	22	4.4	28.9	19p13.12	CIB3	calcium and integrin-binding family member 3 [Homo sapiens].
1386	NP_000206.2	1124	125	6.8	42.1	19p13.1	JAK3	tyrosine-protein kinase JAK3 [Homo sapiens].
1387	NP_055831.1	1309	143	7.9	29.7	19p13.11	MAST3	microtubule-associated serine/threonine-protein kinase 3 [Homo
1388	NP_005018.1	728	82	6.1	26.1	19q13.2-q1	PIK3R2	phosphatidylinositol 3-kinase regulatory subunit beta [Homo
1389	NP_114426.1	273	30	9.4	37.4	19p13.11	TSSK6	testis-specific serine/threonine-protein kinase 6 [Homo sapiens].
1390	NP_055081.1	93	7	8.5	51.4	19q13.1	HCST	hematopoietic cell signal transducer isoform 1 precursor [Homo
1391	NP_001007470.1	92	7	8.9	52.1	19q13.1	HCST	hematopoietic cell signal transducer isoform 2 precursor [Homo
1392	NP_001230876.1	120	13	11.4	13.3	19q13.1	PPP1R14A	protein phosphatase 1 regulatory subunit 14A isoform 2 [Homo
1393	NP_150281.1	147	17	9.8	10.2	19q13.1	PPP1R14A	protein phosphatase 1 regulatory subunit 14A isoform 1 [Homo
1394	NP_001036065.1	821	90	8.1	42.5	19q13.1-q1	MAP4K1	mitogen-activated protein kinase kinase kinase kinase 1 isoform 1
1395	NP_009112.1	833	91	8.3	43.6	19q13.1-q1	MAP4K1	mitogen-activated protein kinase kinase kinase kinase 1 isoform 2
1396	NP_001014831.1	591	64	10.1	31.5	19q13.2	PAK4	serine/threonine-protein kinase PAK 4 isoform 1 [Homo sapiens].
1397	NP_005875.1	591	64	10.1	31.5	19q13.2	PAK4	serine/threonine-protein kinase PAK 4 isoform 1 [Homo sapiens].
1398	NP_001014832.1	591	64	10.1	31.5	19q13.2	PAK4	serine/threonine-protein kinase PAK 4 isoform 1 [Homo sapiens].
1399	NP_001014834.1	438	48	9.6	38.8	19q13.2	PAK4	serine/threonine-protein kinase PAK 4 isoform 2 [Homo sapiens].
1400	NP_001014835.1	438	48	9.6	38.8	19q13.2	PAK4	serine/threonine-protein kinase PAK 4 isoform 2 [Homo sapiens].
1401	NP_006474.1	589	65	9.3	29.0	19q13.2	DYRK1B	dual specificity tyrosine-phosphorylation-regulated kinase 1B
1402	NP_006475.1	601	66	9.3	28.5	19q13.2	DYRK1B	dual specificity tyrosine-phosphorylation-regulated kinase 1B
1403	NP_004705.1	629	69	9.4	28.5	19q13.2	DYRK1B	dual specificity tyrosine-phosphorylation-regulated kinase 1B
1404	NP_002437.2	954	104	6.6	31.3	19q13.2	MAP3K10	mitogen-activated protein kinase kinase kinase 10 [Homo sapiens].
1405	NP_001617.1	481	56	5.9	31.4	19q13.1-q1	AKT2	RAC-beta serine/threonine-protein kinase isoform 1 [Homo sapiens].
1406	NP_001229956.1	419	49	5.8	33.4	19q13.1-q1	AKT2	RAC-beta serine/threonine-protein kinase isoform 2 [Homo sapiens].
1407	NP_001229957.1	419	49	5.8	33.4	19q13.1-q1	AKT2	RAC-beta serine/threonine-protein kinase isoform 2 [Homo sapiens].
1408	NP_653286.2	616	69	6.2	32.0	19q13.2	HIPK4	homeodomain-interacting protein kinase 4 [Homo sapiens].
1409	NP_001136027.1	503	56	7.1	37.4	19q13.2	ADCK4	uncharacterized aarF domain-containing protein kinase 4 isoform b

1410	NP_079152.3	544	60	6.9	38.4	19q13.2	ADCK4	uncharacterized aarF domain-containing protein kinase 4 isoform a
1411	NP_079470.1	683	75	4.9	21.5	19q13.1	ITPKC	inositol-trisphosphate 3-kinase C [Homo sapiens].
1412	NP_068713.2	894	98	5.1	33.9	19q13.1	AXL	tyrosine-protein kinase receptor UFO isoform 1 precursor [Homo
1413	NP_001690.2	885	97	5.1	34.4	19q13.1	AXL	tyrosine-protein kinase receptor UFO isoform 2 precursor [Homo
1414	NP_063937.2	483	51	8.9	38.5	19q13.2	GSK3A	glycogen synthase kinase-3 alpha [Homo sapiens].
1415	NP_001005376.1	281	29	5.8	23.2	19q13	PLAUR	urokinase plasminogen activator surface receptor isoform 2
1416	NP_001005377.1	290	30	5.6	30.6	19q13	PLAUR	urokinase plasminogen activator surface receptor isoform 3
1417	NP_002650.1	335	35	6.1	29.1	19q13	PLAUR	urokinase plasminogen activator surface receptor isoform 1
1418	NP_001186796.1	752	83	10.0	24.6	19q13.3	MARK4	MAP/microtubule affinity-regulating kinase 4 isoform 1 [Homo
1419	NP_113605.2	688	75	10.2	21.5	19q13.3	MARK4	MAP/microtubule affinity-regulating kinase 4 isoform 2 [Homo
1420	NP_001815.2	381	43	6.8	28.1	19q13.32	CKM	creatine kinase M-type [Homo sapiens].
1421	NP_001075031.1	625	70	4.6	37.1	19q13.3	DMPK	myotonin-protein kinase isoform 4 [Homo sapiens].
1422	NP_001075029.1	624	69	4.8	39.4	19q13.3	DMPK	myotonin-protein kinase isoform 3 [Homo sapiens].
1423	NP_004400.4	629	69	4.8	39.7	19q13.3	DMPK	myotonin-protein kinase isoform 2 [Homo sapiens].
1424	NP_001075032.1	639	70	4.9	38.0	19q13.3	DMPK	myotonin-protein kinase isoform 1 [Homo sapiens].
1425	NP_001073349.1	878	97	6.4	37.5	19q13.3	PRKD2	serine/threonine-protein kinase D2 isoform A [Homo sapiens].
1426	NP_057541.2	878	97	6.4	37.5	19q13.3	PRKD2	serine/threonine-protein kinase D2 isoform A [Homo sapiens].
1427	NP_001073350.1	878	97	6.4	37.5	19q13.3	PRKD2	serine/threonine-protein kinase D2 isoform A [Homo sapiens].
1428	NP_001073351.1	721	80	6.9	34.4	19q13.3	PRKD2	serine/threonine-protein kinase D2 isoform B [Homo sapiens].
1429	NP_001073903.1	1489	157	4.7	27.0	19q13.33	LMTK3	serine/threonine-protein kinase LMTK3 [Homo sapiens].
1430	NP_064511.2	654	69	6.5	46.2	19q13.2	SPHK2	sphingosine kinase 2 isoform a [Homo sapiens].
1431	NP_001191088.1	654	69	6.5	46.2	19q13.2	SPHK2	sphingosine kinase 2 isoform a [Homo sapiens].
1432	NP_001191087.1	595	63	6.1	46.9	19q13.2	SPHK2	sphingosine kinase 2 isoform b [Homo sapiens].
1433	NP_001191089.1	618	65	6.7	48.2	19q13.2	SPHK2	sphingosine kinase 2 isoform c [Homo sapiens].
1434	NP_001230805.1	448	47	5.2	51.8	19q13.2	SPHK2	sphingosine kinase 2 isoform d [Homo sapiens].
1435	NP_001191431.1	235	24	7.6	35.9	19q13.3	FLT3LG	fms-related tyrosine kinase 3 ligand precursor [Homo sapiens].
1436	NP_001191432.1	235	24	7.6	35.9	19q13.3	FLT3LG	fms-related tyrosine kinase 3 ligand precursor [Homo sapiens].
1437	NP_001450.2	235	24	7.6	35.9	19q13.3	FLT3LG	fms-related tyrosine kinase 3 ligand precursor [Homo sapiens].
1438	NP_068379.1	592	65	5.6	20.4	19q13.3	TSKS	testis-specific serine kinase substrate [Homo sapiens].
1439	NP_009185.2	521	57	8.5	39.9	19q13.3-q1	PNKP	bifunctional polynucleotide phosphatase/kinase [Homo sapiens].
1440	NP_001020949.1	424	48	9.2	31.1	19q13	VRK3	inactive serine/threonine-protein kinase VRK3 isoform 2 [Homo
1441	NP_057524.3	474	53	9.3	28.7	19q13	VRK3	inactive serine/threonine-protein kinase VRK3 isoform 1 [Homo
1442	NP_002730.1	697	78	7.2	32.4	19q13.4	PRKCG	protein kinase C gamma type [Homo sapiens].
1443	NP_573441.2	723	80	5.7	22.4	19q13.42	EPS8L1	epidermal growth factor receptor kinase substrate 8-like protein 1
1444	NP_060199.3	596	66	5.9	18.5	19q13.42	EPS8L1	epidermal growth factor receptor kinase substrate 8-like protein 1
1445	NP_115806.1	778	85	9.7	26.3	19q13.4	BRSK1	serine/threonine-protein kinase BRSK1 [Homo sapiens].
1446	NP_001094871.2	348	38	5.2	44.8	19q13.42	SBK2	serine/threonine-protein kinase SBK2 [Homo sapiens].
1447	NP_001186753.1	359	38	4.6	47.1	19q13.42	SGK110	putative uncharacterized serine/threonine-protein kinase SgK110
1448	NP_001015879.1	290	34	9.0	32.4	19q13.43	AURKC	aurora kinase C isoform 2 [Homo sapiens].
1449	NP_001015878.1	309	36	9.0	32.7	19q13.43	AURKC	aurora kinase C isoform 1 [Homo sapiens].
1450	NP_003151.2	275	32	9.0	34.2	19q13.43	AURKC	aurora kinase C isoform 3 [Homo sapiens].
1451	NP_808227.1	391	45	7.6	30.4	20p13	CSNK2A1	casein kinase II subunit alpha isoform a [Homo sapiens].
1452	NP_001886.1	391	45	7.6	30.4	20p13	CSNK2A1	casein kinase II subunit alpha isoform a [Homo sapiens].
1453	NP_808228.1	255	29	6.2	33.7	20p13	CSNK2A1	casein kinase II subunit alpha isoform b [Homo sapiens].
1454	NP_001186715.1	97	11	7.1	28.9	20p13	FKBP1A	peptidyl-prolyl cis-trans isomerase FKBP1A isoform b [Homo
1455	NP_000792.1	108	12	8.6	25.9	20p13	FKBP1A	peptidyl-prolyl cis-trans isomerase FKBP1A isoform a [Homo
1456	NP_463460.1	108	12	8.6	25.9	20p13	FKBP1A	peptidyl-prolyl cis-trans isomerase FKBP1A isoform a [Homo
1457	NP_543026.2	534	58	10.1	33.0	20p13	STK35	serine/threonine-protein kinase 35 [Homo sapiens].
1458	NP_068598.1	764	87	8.0	44.5	20p13	FASTKD5	FAST kinase domain-containing protein 5 [Homo sapiens].
1459	NP_705902.2	570	47	7.4	40.7	20p13	PANK2	pantothenate kinase 2, mitochondrial isoform 1 preproprotein [Homo
1460	NP_079236.3	279	31	5.7	47.0	20p13	PANK2	pantothenate kinase 2, mitochondrial isoform 2 [Homo sapiens].
1461	NP_705904.1	279	31	5.7	47.0	20p13	PANK2	pantothenate kinase 2, mitochondrial isoform 2 [Homo sapiens].
1462	NP_065074.1	719	81	8.0	25.5	20p12	PAK7	serine/threonine-protein kinase PAK 7 [Homo sapiens].
1463	NP_817127.1	719	81	8.0	25.5	20p12	PAK7	serine/threonine-protein kinase PAK 7 [Homo sapiens].
1464	NP_149109.1	596	65	6.6	27.7	20q13.31	MYLK2	myosin light chain kinase 2, skeletal/cardiac muscle [Homo
1465	NP_002101.2	526	60	6.3	30.0	20q11-q12	HCK	tyrosine-protein kinase HCK isoform a [Homo sapiens].
1466	NP_001165601.1	525	60	6.3	29.9	20q11-q12	HCK	tyrosine-protein kinase HCK isoform c [Homo sapiens].
1467	NP_001165603.1	506	57	6.4	31.4	20q11-q12	HCK	tyrosine-protein kinase HCK isoform e [Homo sapiens].
1468	NP_001165600.1	505	57	6.4	31.3	20q11-q12	HCK	tyrosine-protein kinase HCK isoform b [Homo sapiens].

1469	NP_001165604.1	505	57	6.4	31.3	20q11-q12	HCK	tyrosine-protein kinase HCK isoform b [Homo sapiens].
1470	NP_001165602.1	504	57	6.4	31.2	20q11-q12	HCK	tyrosine-protein kinase HCK isoform d [Homo sapiens].
1471	NP_005408.1	536	60	7.2	28.2	20q12-q13	SRC	proto-oncogene tyrosine-protein kinase Src [Homo sapiens].
1472	NP_938033.1	536	60	7.2	28.2	20q12-q13	SRC	proto-oncogene tyrosine-protein kinase Src [Homo sapiens].
1473	NP_057360.2	427	48	7.2	34.0	20q13.2	SGK2	serine/threonine-protein kinase Sgk2 isoform beta [Homo sapiens].
1474	NP_001186193.1	367	41	6.3	36.2	20q13.2	SGK2	serine/threonine-protein kinase Sgk2 isoform alpha [Homo sapiens].
1475	NP_733794.1	367	41	6.3	36.2	20q13.2	SGK2	serine/threonine-protein kinase Sgk2 isoform alpha [Homo sapiens].
1476	NP_861520.1	76	8	3.9	13.2	20q12-q13.	PKIG	cAMP-dependent protein kinase inhibitor gamma [Homo sapiens].
1477	NP_861521.1	76	8	3.9	13.2	20q12-q13.	PKIG	cAMP-dependent protein kinase inhibitor gamma [Homo sapiens].
1478	NP_008997.1	76	8	3.9	13.2	20q12-q13.	PKIG	cAMP-dependent protein kinase inhibitor gamma [Homo sapiens].
1479	NP_003395.1	246	28	4.6	24.0	20q13.1	YWHAB	14-3-3 protein beta/alpha [Homo sapiens].
1480	NP_647539.1	246	28	4.6	24.0	20q13.1	YWHAB	14-3-3 protein beta/alpha [Homo sapiens].
1481	NP_006273.1	487	56	4.8	31.6	20q11.2-q1	STK4	serine/threonine-protein kinase 4 [Homo sapiens].
1482	NP_898869.1	1135	125	6.0	19.6	20q13.12	ZMYND8	protein kinase C-binding protein 1 isoform c [Homo sapiens].
1483	NP_036540.3	1160	128	6.0	19.5	20q13.12	ZMYND8	protein kinase C-binding protein 1 isoform b [Homo sapiens].
1484	NP_898868.1	1188	132	6.2	19.1	20q13.12	ZMYND8	protein kinase C-binding protein 1 isoform a [Homo sapiens].
1485	NP_060901.2	306	35	8.9	24.5	20q13.2	DOK5	docking protein 5 [Homo sapiens].
1486	NP_003591.2	403	46	9.7	24.1	20q13	AURKA	aurora kinase A [Homo sapiens].
1487	NP_940835.1	403	46	9.7	24.1	20q13	AURKA	aurora kinase A [Homo sapiens].
1488	NP_940836.1	403	46	9.7	24.1	20q13	AURKA	aurora kinase A [Homo sapiens].
1489	NP_940837.1	403	46	9.7	24.1	20q13	AURKA	aurora kinase A [Homo sapiens].
1490	NP_940838.1	403	46	9.7	24.1	20q13	AURKA	aurora kinase A [Homo sapiens].
1491	NP_940839.1	403	46	9.7	24.1	20q13	AURKA	aurora kinase A [Homo sapiens].
1492	NP_005966.1	451	52	6.6	33.7	20q13.3	PTK6	protein-tyrosine kinase 6 Isoform 1 [Homo sapiens].
1493	NP_001243287.1	134	14	5.2	24.6	20q13.3	PTK6	protein-tyrosine kinase 6 Isoform 2 [Homo sapiens].
1494	NP_543013.1	488	55	8.1	37.5	20q13.33	SRMS	tyrosine-protein kinase Srms [Homo sapiens].
1495	NP_055401.1	714	80	9.4	29.1	21q22.1	HUNK	hormonally up-regulated neu tumor-associated kinase [Homo sapiens].
1496	NP_005797.1	323	32	9.5	43.3	21q22.11	OLIG2	oligodendrocyte transcription factor 2 [Homo sapiens].
1497	NP_001387.2	763	86	8.9	24.0	21q22.13	DYRK1A	dual specificity tyrosine-phosphorylation-regulated kinase 1A
1498	NP_569120.1	754	85	9.0	23.2	21q22.13	DYRK1A	dual specificity tyrosine-phosphorylation-regulated kinase 1A
1499	NP_569122.1	529	60	9.4	31.2	21q22.13	DYRK1A	dual specificity tyrosine-phosphorylation-regulated kinase 1A
1500	NP_567824.1	584	66	9.3	31.0	21q22.13	DYRK1A	dual specificity tyrosine-phosphorylation-regulated kinase 1A
1501	NP_065690.2	784	86	6.6	37.5	21q22.3	RIPK4	receptor-interacting serine/threonine-protein kinase 4 [Homo
1502	NP_775490.2	783	85	6.8	40.2	21q22.3	SIK1	serine/threonine-protein kinase SIK1 [Homo sapiens].
1503	NP_002617.3	780	85	7.2	46.8	21q22.3	PFKL	6-phosphofructokinase, liver type [Homo sapiens].
1504	NP_443732.3	358	41	9.0	29.1	22q11.21	TSSK2	testis-specific serine/threonine-protein kinase 2 [Homo sapiens].
1505	NP_477352.3	2102	237	6.6	44.5	22q11.21	PI4KA	phosphatidylinositol 4-kinase alpha isoform 1 [Homo sapiens].
1506	NP_002641.1	854	97	6.9	40.3	22q11.21	PI4KA	phosphatidylinositol 4-kinase alpha isoform 2 [Homo sapiens].
1507	NP_002736.3	360	41	6.5	35.3	22q11.21	MAPK1	mitogen-activated protein kinase 1 [Homo sapiens].
1508	NP_620407.1	360	41	6.5	35.3	22q11.21	MAPK1	mitogen-activated protein kinase 1 [Homo sapiens].
1509	NP_055449.1	454	50	4.9	37.9	22q11.22	PPM1F	protein phosphatase 1F [Homo sapiens].
1510	NP_005151.2	688	80	7.4	26.3	22q12.1	ADRBK2	beta-adrenergic receptor kinase 2 [Homo sapiens].
1511	NP_665861.1	514	58	5.7	28.8	22q12.1	CHEK2	serine/threonine-protein kinase Chk2 isoform b [Homo sapiens].
1512	NP_009125.1	543	61	5.6	28.0	22q12.1	CHEK2	serine/threonine-protein kinase Chk2 isoform a [Homo sapiens].
1513	NP_001005735.1	586	65	5.8	27.8	22q12.1	CHEK2	serine/threonine-protein kinase Chk2 isoform c [Homo sapiens].
1514	NP_001244316.1	322	36	6.7	37.0	22q12.1	CHEK2	serine/threonine-protein kinase Chk2 isoform d [Homo sapiens].
1515	NP_005560.1	638	72	6.8	34.3	22q12.2	LIMK2	LIM domain kinase 2 isoform 2a [Homo sapiens].
1516	NP_057952.1	617	70	7.1	35.8	22q12.2	LIMK2	LIM domain kinase 2 isoform 2b [Homo sapiens].
1517	NP_001026971.1	686	78	6.6	33.2	22q12.2	LIMK2	LIM domain kinase 2 isoform 1 [Homo sapiens].
1518	NP_114439.1	641	69	8.0	39.3	22q12.2	PATZ1	POZ-, AT hook-, and zinc finger-containing protein 1 long A isoform
1519	NP_055138.2	687	74	8.2	39.0	22q12.2	PATZ1	POZ-, AT hook-, and zinc finger-containing protein 1 long C isoform
1520	NP_114441.1	537	58	8.5	40.2	22q12.2	PATZ1	POZ-, AT hook-, and zinc finger-containing protein 1 long B isoform
1521	NP_114440.1	537	58	8.4	43.4	22q12.2	PATZ1	POZ-, AT hook-, and zinc finger-containing protein 1 short isoform
1522	NP_001034672.1	415	47	5.0	34.5	22q13.1	PICK1	PRKCA-binding protein [Homo sapiens].
1523	NP_036539.1	415	47	5.0	34.5	22q13.1	PICK1	PRKCA-binding protein [Homo sapiens].
1524	NP_001034673.1	415	47	5.0	34.5	22q13.1	PICK1	PRKCA-binding protein [Homo sapiens].
1525	NP_001885.1	416	47	10.0	25.2	22q13.1	CSNK1E	casein kinase I isoform epsilon [Homo sapiens].
1526	NP_689407.1	416	47	10.0	25.2	22q13.1	CSNK1E	casein kinase I isoform epsilon [Homo sapiens].
1527	NP_705717.1	462	50	5.3	37.4	22q13.1	TAB1	TGF-beta-activated kinase 1 and MAP3K7-binding protein 1 isoform

1528	NP_006107.1	504	55	5.2	32.5	22q13.1	TAB1	TGF-beta-activated kinase 1 and MAP3K7-binding protein 1 isoform
1529	NP_001171900.1	445	51	5.1	10.8	22q13.2-q1	PACSIN2	protein kinase C and casein kinase substrate in neurons protein 2
1530	NP_009160.2	486	56	4.9	9.9	22q13.2-q1	PACSIN2	protein kinase C and casein kinase substrate in neurons protein 2
1531	NP_001171899.1	486	56	4.9	9.9	22q13.2-q1	PACSIN2	protein kinase C and casein kinase substrate in neurons protein 2
1532	NP_073603.2	537	60	8.1	42.5	22q13.31	CERK	ceramide kinase [Homo sapiens].
1533	NP_001001852.2	326	36	5.7	48.2	22q13	PIM3	serine/threonine-protein kinase pim-3 [Homo sapiens].
1534	NP_002960.2	367	42	6.0	32.4	22q13.33	MAPK12	mitogen-activated protein kinase 12 [Homo sapiens].
1535	NP_002742.3	364	41	5.5	36.3	22q13.33	MAPK11	mitogen-activated protein kinase 11 [Homo sapiens].
1536	NP_005189.2	395	45	5.2	35.9	22q13.33	CHKB	choline/ethanolamine kinase [Homo sapiens].
1537	NP_036456.1	824	88	4.2	27.2	22q13.33	MAPK8IP2	C-Jun-amino-terminal kinase-interacting protein 2 isoform 1 [Homo
1538	NP_057515.1	797	85	4.2	29.1	22q13.33	MAPK8IP2	C-Jun-amino-terminal kinase-interacting protein 2 isoform 2 [Homo
1539	NP_005079.2	695	81	10.2	21.7	Xp22.33 an	AKAP17A	A-kinase anchor protein 17A isoform 1 [Homo sapiens].
1540	NP_005035.1	358	41	6.4	34.9	Xp22.3	PRKX	cAMP-dependent protein kinase catalytic subunit PRKX [Homo
1541	NP_001034180.1	321	35	6.0	58.3	Xp22.2	PRPS2	ribose-phosphate pyrophosphokinase 2 isoform 1 [Homo sapiens].
1542	NP_002756.1	318	35	6.2	58.8	Xp22.2	PRPS2	ribose-phosphate pyrophosphokinase 2 isoform 2 [Homo sapiens].
1543	NP_975010.1	675	78	8.5	25.8	Xp22.2	BMX	cytoplasmic tyrosine-protein kinase BMX [Homo sapiens].
1544	NP_001712.1	675	78	8.5	25.8	Xp22.2	BMX	cytoplasmic tyrosine-protein kinase BMX [Homo sapiens].
1545	NP_003150.1	1030	116	9.9	17.9	Xp22	CDKL5	cyclin-dependent kinase-like 5 [Homo sapiens].
1546	NP_001032420.1	1030	116	9.9	17.9	Xp22	CDKL5	cyclin-dependent kinase-like 5 [Homo sapiens].
1547	NP_000283.1	1235	138	6.0	41.9	Xp22.2-p22	PHKA2	phosphorylase b kinase regulatory subunit alpha, liver isoform
1548	NP_001001671.3	1313	147	5.3	33.4	Xp22.12	MAP3K15	mitogen-activated protein kinase kinase kinase 15 [Homo sapiens].
1549	NP_114098.1	665	73	6.2	21.1	Xp22.1-p21	SH3KBP1	SH3 domain-containing kinase-binding protein 1 isoform a [Homo
1550	NP_001019837.1	628	69	7.4	21.2	Xp22.1-p21	SH3KBP1	SH3 domain-containing kinase-binding protein 1 isoform b [Homo
1551	NP_001171889.1	427	47	9.3	20.6	Xp22.1-p21	SH3KBP1	SH3 domain-containing kinase-binding protein 1 isoform c [Homo
1552	NP_004577.1	740	84	6.4	35.0	Xp22.2-p22	RPS6KA3	ribosomal protein S6 kinase alpha-3 [Homo sapiens].
1553	NP_055742.2	1034	118	6.3	22.4	Xp22.12	CNKSR2	connector enhancer of kinase suppressor of ras 2 isoform 1 [Homo
1554	NP_001162118.1	1004	114	6.2	22.9	Xp22.12	CNKSR2	connector enhancer of kinase suppressor of ras 2 isoform 2 [Homo
1555	NP_001162119.1	898	102	6.6	22.0	Xp22.12	CNKSR2	connector enhancer of kinase suppressor of ras 2 isoform 3 [Homo
1556	NP_001162120.1	849	96	6.2	20.8	Xp22.12	CNKSR2	connector enhancer of kinase suppressor of ras 2 isoform 4 [Homo
1557	NP_001135858.1	415	48	8.9	32.5	Xp22.11	PDK3	pyruvate dehydrogenase kinase, isozyme 3 isoform 1 [Homo sapiens].
1558	NP_005382.1	406	47	8.6	33.3	Xp22.11	PDK3	pyruvate dehydrogenase kinase, isozyme 3 isoform 2 [Homo sapiens].
1559	NP_001191948.1	559	61	6.1	49.6	Xp21.3	GK	glycerol kinase isoform d [Homo sapiens].
1560	NP_976325.1	530	58	6.1	47.5	Xp21.3	GK	glycerol kinase isoform a [Homo sapiens].
1561	NP_001121599.1	553	61	5.9	49.7	Xp21.3	GK	glycerol kinase isoform c [Homo sapiens].
1562	NP_000158.1	524	57	5.9	47.7	Xp21.3	GK	glycerol kinase isoform b [Homo sapiens].
1563	NP_690000.2	712	79	8.4	16.3	Xp21.2	TAB3	TGF-beta-activated kinase 1 and MAP3K7-binding protein 3 [Homo
1564	NP_001119527.1	897	102	5.9	33.9	Xp11.4	CASK	peripheral plasma membrane protein CASK isoform 3 [Homo sapiens].
1565	NP_001119526.1	898	102	5.9	34.1	Xp11.4	CASK	peripheral plasma membrane protein CASK isoform 2 [Homo sapiens].
1566	NP_003679.2	921	105	6.0	33.2	Xp11.4	CASK	peripheral plasma membrane protein CASK isoform 1 [Homo sapiens].
1567	NP_148978.2	502	56	7.0	25.9	Xp11	CDK16	cyclin-dependent kinase 16 isoform 2 [Homo sapiens].
1568	NP_001163931.1	570	63	8.1	28.2	Xp11	CDK16	cyclin-dependent kinase 16 isoform 3 [Homo sapiens].
1569	NP_006192.1	496	56	7.4	26.2	Xp11	CDK16	cyclin-dependent kinase 16 isoform 1 [Homo sapiens].
1570	NP_001243125.1	609	68	9.3	33.5	Xp11.4-p11	ARAF	serine/threonine-protein kinase A-Raf isoform 2 [Homo sapiens].
1571	NP_001645.1	606	68	9.2	33.7	Xp11.4-p11	ARAF	serine/threonine-protein kinase A-Raf isoform 1 [Homo sapiens].
1572	NP_001243126.1	186	21	8.5	34.4	Xp11.4-p11	ARAF	serine/threonine-protein kinase A-Raf isoform 3 [Homo sapiens].
1573	NP_001107595.1	428	45	6.5	37.9	Xp11.2	ELK1	ETS domain-containing protein Elk-1 isoform a [Homo sapiens].
1574	NP_005220.2	428	45	6.5	37.9	Xp11.2	ELK1	ETS domain-containing protein Elk-1 isoform a [Homo sapiens].
1575	NP_001244097.1	95	11	9.8	18.9	Xp11.2	ELK1	ETS domain-containing protein Elk-1 isoform b [Homo sapiens].
1576	NP_006866.2	311	34	5.5	41.5	Xp11.23	PIM2	serine/threonine-protein kinase pim-2 [Homo sapiens].
1577	NP_003877.2	854	94	6.5	27.6	Xp11.2	AKAP4	A-kinase anchor protein 4 isoform 1 [Homo sapiens].
1578	NP_647450.1	845	93	6.7	27.7	Xp11.2	AKAP4	A-kinase anchor protein 4 isoform 2 [Homo sapiens].
1579	NP_001013764.1	1271	142	5.3	31.3	Xp11.22	DGKK	diacylglycerol kinase kappa [Homo sapiens].
1580	NP_001002838.1	1743	192	5.4	25.0	Xp11.22	WNK3	serine/threonine-protein kinase WNK3 isoform 2 [Homo sapiens].
1581	NP_065973.2	1800	198	5.7	24.7	Xp11.22	WNK3	serine/threonine-protein kinase WNK3 isoform 1 [Homo sapiens].
1582	NP_004420.1	346	35	8.6	27.9	Xq12	EFNB1	ephrin-B1 precursor [Homo sapiens].
1583	NP_001165907.1	1151	129	5.6	42.6	Xq12-q13	PHKA1	phosphorylase b kinase regulatory subunit alpha, skeletal muscle
1584	NP_001116142.1	1210	136	5.8	42.0	Xq12-q13	PHKA1	phosphorylase b kinase regulatory subunit alpha, skeletal muscle
1585	NP_002628.2	1223	137	5.8	41.6	Xq12-q13	PHKA1	phosphorylase b kinase regulatory subunit alpha, skeletal muscle
1586	NP_000282.1	417	45	8.1	47.5	Xq13.3	PGK1	phosphoglycerate kinase 1 [Homo sapiens].

1587	NP_055311.1	745	84	5.9	38.1	Xq21	RPS6KA6	ribosomal protein S6 kinase alpha-6 [Homo sapiens].
1588	NP_000052.1	659	76	7.6	28.2	Xq21.33-q2	BTK	tyrosine-protein kinase BTK [Homo sapiens].
1589	NP_002755.1	318	35	6.5	56.6	Xq21.32-q2	PRPS1	ribose-phosphate pyrophosphokinase 1 isoform 1 [Homo sapiens].
1590	NP_001191331.1	114	12	6.2	65.8	Xq21.32-q2	PRPS1	ribose-phosphate pyrophosphokinase 1 isoform 2 [Homo sapiens].
1591	NP_001121640.1	580	65	5.3	30.3	Xq23	PAK3	serine/threonine-protein kinase PAK 3 isoform b [Homo sapiens].
1592	NP_001121644.1	565	63	5.3	31.0	Xq23	PAK3	serine/threonine-protein kinase PAK 3 isoform c [Homo sapiens].
1593	NP_001121645.1	559	62	5.2	30.9	Xq23	PAK3	serine/threonine-protein kinase PAK 3 isoform d [Homo sapiens].
1594	NP_002569.1	544	61	5.3	31.8	Xq23	PAK3	serine/threonine-protein kinase PAK 3 isoform a [Homo sapiens].
1595	NP_001121638.1	544	61	5.3	31.8	Xq23	PAK3	serine/threonine-protein kinase PAK 3 isoform a [Homo sapiens].
1596	NP_001121639.1	544	61	5.3	31.8	Xq23	PAK3	serine/threonine-protein kinase PAK 3 isoform a [Homo sapiens].
1597	NP_848928.1	197	23	6.3	31.0	Xq24	AKAP14	A-kinase anchor protein 14 isoform a [Homo sapiens].
1598	NP_001008534.1	137	16	4.8	22.6	Xq24	AKAP14	A-kinase anchor protein 14 isoform b [Homo sapiens].
1599	NP_001008535.1	89	10	4.3	15.7	Xq24	AKAP14	A-kinase anchor protein 14 isoform c [Homo sapiens].
1600	NP_057626.2	416	47	5.0	33.7	Xq26.2	MST4	serine/threonine-protein kinase MST4 isoform 1 [Homo sapiens].
1601	NP_001035917.1	354	40	5.0	32.2	Xq26.2	MST4	serine/threonine-protein kinase MST4 isoform 3 [Homo sapiens].
1602	NP_001035918.1	339	38	5.7	36.0	Xq26.2	MST4	serine/threonine-protein kinase MST4 isoform 2 [Homo sapiens].
1603	NP_001386.1	384	42	5.7	39.8	Xq28	DUSP9	dual specificity protein phosphatase 9 [Homo sapiens].
1604	NP_001034671.3	426	47	6.8	33.8	Xq28	PNCK	calcium/calmodulin-dependent protein kinase type 1B isoform a [Homo
1605	NP_001129212.1	360	40	6.8	36.7	Xq28	PNCK	calcium/calmodulin-dependent protein kinase type 1B isoform b [Homo
1606	NP_055185.2	567	62	6.9	34.2	Xq28	SRPK3	SRSF protein kinase 3 isoform 1 [Homo sapiens].
1607	NP_001164231.1	566	62	6.9	34.3	Xq28	SRPK3	SRSF protein kinase 3 isoform 2 [Homo sapiens].
1608	NP_001164232.1	533	59	6.6	36.6	Xq28	SRPK3	SRSF protein kinase 3 isoform 3 [Homo sapiens].
1609	NP_001020414.1	633	68	6.0	35.1	Xq28	IRAK1	interleukin-1 receptor-associated kinase 1 isoform 3 [Homo
1610	NP_001020413.1	682	73	6.2	35.5	Xq28	IRAK1	interleukin-1 receptor-associated kinase 1 isoform 2 [Homo
1611	NP_001560.2	712	77	6.2	36.0	Xq28	IRAK1	interleukin-1 receptor-associated kinase 1 isoform 1 [Homo
1612	NP_001093326.2	487	56	6.3	17.9	Xq28	IKBKG	NF-kappa-B essential modulator isoform b [Homo sapiens].
1613	NP_003630.1	419	48	5.5	14.1	Xq28	IKBKG	NF-kappa-B essential modulator isoform a [Homo sapiens].
1614	NP_001093327.1	419	48	5.5	14.1	Xq28	IKBKG	NF-kappa-B essential modulator isoform a [Homo sapiens].
1615	NP_001138727.1	320	37	5.8	13.4	Xq28	IKBKG	NF-kappa-B essential modulator isoform c [Homo sapiens].
1616	XP_003960221.1	270	30	6.0	28.5	1	LOC101060	PREDICTED: 5'-AMP-activated protein kinase subunit beta-2-like
1617	XP_003960220.1	272	30	6.0	28.3	1	LOC101060	PREDICTED: 5'-AMP-activated protein kinase subunit beta-2-like
1618	XP_003960222.1	190	21	6.5	37.4	1	LOC101060	PREDICTED: 5'-AMP-activated protein kinase subunit beta-2-like
1619	XP_003960585.1	149	17	4.0	12.8	11q13.1	LOC100506	PREDICTED: serine/threonine-protein kinase Nek5-like [Homo
1620	NP_002489.1	506	58	6.8	31.6	13q14.13	NEK3	serine/threonine-protein kinase Nek3 isoform a [Homo sapiens].
1621	XP_003960635.1	178	21	8.9	31.5	14q32	CKMT1B	PREDICTED: creatine kinase U-type, mitochondrial isoform 1 [Homo
1622	XP_003960636.1	113	13	9.3	43.4	14q32	CKMT1B	PREDICTED: creatine kinase U-type, mitochondrial isoform 2 [Homo
1623	XP_003960698.1	191	21	9.1	12.0	14q32	LOC101060	PREDICTED: serine/threonine-protein kinase SMG1-like [Homo
1624	XP_003960679.1	839	93	7.0	53.3	14q32	LOC101060	PREDICTED: serine/threonine-protein kinase SMG1-like [Homo
1625	XP_003960701.1	227	25	9.6	22.0	14q32	LOC101060	PREDICTED: serine/threonine-protein kinase SMG1-like [Homo
1626	XP_003960702.1	411	45	6.5	58.6	14q32	LOC101060	PREDICTED: serine/threonine-protein kinase SMG1-like [Homo
1627	XP_003960802.1	118	13	8.1	32.2	14q32	LOC390877	PREDICTED: adenylate kinase isoenzyme 1-like [Homo sapiens].
1628	XP_003960871.1	398	45	5.8	38.4	19q13.3	MAPK11	PREDICTED: mitogen-activated protein kinase 11 [Homo sapiens].
1629	XP_003119578.1	223	25	8.7	25.1	11q24.2	LOC100507	PREDICTED: adenylate kinase isoenzyme 4, mitochondrial-like [Homo
1630	XP_003960919.1	184	21	9.1	39.7	11q24.2	LOC101060	PREDICTED: nucleoside diphosphate kinase B-like [Homo sapiens].
1631	XP_003119604.1	149	17	4.0	12.8	11q24.2	LOC100506	PREDICTED: serine/threonine-protein kinase Nek5-like [Homo
1632	XP_003960932.1	471	52	7.8	35.2	11q24.2	LOC101060	PREDICTED: 3-phosphoinositide-dependent protein kinase 1-like [Homo
1633	XP_003403749.3	118	13	8.1	32.2	11q24.2	LOC390877	PREDICTED: adenylate kinase isoenzyme 1-like [Homo sapiens].
1634	XP_003846682.1	354	39	7.6	40.4	21p11.1	LOC100996	PREDICTED: phosphatidylinositol 4-kinase alpha-like [Homo sapiens].
1635	XP_003846692.1	357	41	5.4	34.7	21p11.1	MAPK12	PREDICTED: mitogen-activated protein kinase 12 [Homo sapiens].
1636	XP_003846795.1	142	16	4.9	28.2	10	LOC100996	PREDICTED: dual specificity mitogen-activated protein kinase kinase

Additional entries:

1	NP_001229838.1	236	26	8.8	21.2	1p34.3	RSPO1	R-spondin-1 isoform 2 [Homo sapiens].
2	NP_001229839.1	200	22	9.5	29.0	1p34.3	RSPO1	R-spondin-1 isoform 3 precursor [Homo sapiens].
3	NP_001033722.1	263	29	9.3	24.0	1p34.3	RSPO1	R-spondin-1 isoform 1 precursor [Homo sapiens].
4	NP_001229837.1	263	29	9.3	24.0	1p34.3	RSPO1	R-spondin-1 isoform 1 precursor [Homo sapiens].
5	NP_001092909.1	1008	106	6.3	40.4	1p34.3	EPHA10	ephrin type-A receptor 10 isoform 3 precursor [Homo sapiens].

6	NP_892021.1	582	66	4.9	12.9	1p22.2	ZNF326	DBIRD complex subunit ZNF326 isoform 1 [Homo sapiens].
7	NP_001157945.1	605	70	6.2	14.0	1p22.1	FNBP1L	formin-binding protein 1-like isoform 3 [Homo sapiens].
8	NP_060207.2	547	64	5.8	12.1	1p22.1	FNBP1L	formin-binding protein 1-like isoform 2 [Homo sapiens].
9	NP_001020119.1	551	64	5.8	12.0	1p22.1	FNBP1L	formin-binding protein 1-like isoform 1 [Homo sapiens].
10	NP_004806.3	1261	142	6.3	30.2	1p22.1	ARHGAP29	rho GTPase-activating protein 29 [Homo sapiens].
11	NP_006104.4	847	98	6.6	29.4	1p13.3	VAV3	guanine nucleotide exchange factor VAV3 isoform 1 [Homo sapiens].
12	NP_000692.2	1023	113	5.2	49.6	1p21	ATP1A1	sodium/potassium-transporting ATPase subunit alpha-1 isoform a
13	NP_001153705.1	1023	113	5.2	49.6	1p21	ATP1A1	sodium/potassium-transporting ATPase subunit alpha-1 isoform c
14	NP_001138428.1	92	11	10.0	21.7	1q21.3	RIIAD1	RIIa domain-containing protein 1 [Homo sapiens].
15	NP_056656.2	1181	131	8.7	26.1	1q21.3	ADAR	double-stranded RNA-specific adenosine deaminase isoform c [Homo
16	NP_056655.2	1200	133	8.5	25.8	1q21.3	ADAR	double-stranded RNA-specific adenosine deaminase isoform b [Homo
17	NP_001102.2	1226	136	8.6	26.1	1q21.3	ADAR	double-stranded RNA-specific adenosine deaminase isoform a [Homo
18	NP_001180424.1	931	104	8.7	27.8	1q21.3	ADAR	double-stranded RNA-specific adenosine deaminase isoform d [Homo
19	NP_001020278.1	931	104	8.7	27.8	1q21.3	ADAR	double-stranded RNA-specific adenosine deaminase isoform d [Homo
20	NP_001155856.1	985	111	6.9	27.1	1q21-q22	ARHGEF2	rho guanine nucleotide exchange factor 2 isoform 2 [Homo sapiens].
21	NP_001155855.1	986	112	6.9	27.2	1q21-q22	ARHGEF2	rho guanine nucleotide exchange factor 2 isoform 1 [Homo sapiens].
22	NP_004714.2	958	108	6.4	27.8	1q21-q22	ARHGEF2	rho guanine nucleotide exchange factor 2 isoform 3 [Homo sapiens].
23	NP_055030.1	1297	141	6.1	40.5	1q21-q23	INSRR	insulin receptor-related protein precursor [Homo sapiens].
24	NP_055030.1	1297	79	6.1	40.6	1q21-q23	INSRR	insulin receptor-related protein precursor [Homo sapiens].
25	NP_055030.1	1297	61	5.7	40.7	1q21-q23	INSRR	insulin receptor-related protein precursor [Homo sapiens].
26	NP_000693.1	1020	112	5.3	48.1	1q23.2	ATP1A2	sodium/potassium-transporting ATPase subunit alpha-2 proprotein
27	NP_065156.5	688	77	4.7	42.2	1q24.2	SCYL3	protein-associating with the carboxyl-terminal domain of ezrin
28	NP_851607.2	742	83	4.8	41.9	1q24.2	SCYL3	protein-associating with the carboxyl-terminal domain of ezrin
29	NP_055679.1	409	46	6.4	43.5	1q25	FAM20B	glycosaminoglycan xylosylkinase [Homo sapiens].
30	NP_066956.1	741	84	6.2	32.5	1q25	RNASEL	2-5A-dependent ribonuclease [Homo sapiens].
31	NP_001348.2	1270	141	6.4	36.9	1q25	DHX9	ATP-dependent RNA helicase A [Homo sapiens].
32	NP_872604.1	418	47	9.4	26.6	1q32.1	RASSF5	ras association domain-containing protein 5 isoform A [Homo
33	NP_872605.1	336	37	9.9	25.0	1q32.1	RASSF5	ras association domain-containing protein 5 isoform B [Homo
34	NP_071411.1	322	34	4.8	53.7	2p23.3	RBKS	ribokinase [Homo sapiens].
35	NP_733772.1	690	78	6.3	36.1	2p25.1-p24	RASGRP3	ras guanyl-releasing protein 3 isoform 1 [Homo sapiens].
36	NP_001132960.1	690	78	6.3	36.1	2p25.1-p24	RASGRP3	ras guanyl-releasing protein 3 isoform 1 [Homo sapiens].
37	NP_056191.1	689	78	6.3	36.1	2p25.1-p24	RASGRP3	ras guanyl-releasing protein 3 isoform 2 [Homo sapiens].
38	NP_550438.1	277	28	6.3	36.6	2p13	DGUOK	deoxyguanosine kinase, mitochondrial isoform a [Homo sapiens].
39	NP_149035.1	550	61	6.1	34.4	2p13.1	RTKN	rhotekin isoform b [Homo sapiens].
40	NP_001015056.1	513	57	6.4	34.7	2p13.1	RTKN	rhotekin isoform c [Homo sapiens].
41	NP_490595.1	730	81	9.2	26.0	2q21.3	CCNT2	cyclin-T2 isoform b [Homo sapiens].
42	NP_001232.1	663	74	9.5	26.2	2q21.3	CCNT2	cyclin-T2 isoform a [Homo sapiens].
43	NP_001607.1	513	56	5.5	39.1	2q22.3	ACVR2A	activin receptor type-2A precursor [Homo sapiens].
44	NP_001020372.2	433	50	6.2	32.1	2q31.1	CHN1	N-chimaerin isoform 2 [Homo sapiens].
45	NP_001813.1	459	53	6.5	30.7	2q31.1	CHN1	N-chimaerin isoform 1 [Homo sapiens].
46	NP_001193531.1	334	38	8.2	37.1	2q31.1	CHN1	N-chimaerin isoform 3 [Homo sapiens].
47	NP_001025483.1	419	47	8.0	39.4	2q31.3	CERKL	ceramide kinase-like protein isoform 3 [Homo sapiens].
48	NP_001153749.1	514	58	7.6	41.6	2q31.3	CERKL	ceramide kinase-like protein isoform 7 [Homo sapiens].
49	NP_001025484.1	463	52	8.7	37.8	2q31.3	CERKL	ceramide kinase-like protein isoform 4 [Homo sapiens].
50	NP_963842.1	532	60	7.9	41.2	2q31.3	CERKL	ceramide kinase-like protein isoform 1 [Homo sapiens].
51	NP_001025482.1	558	63	8.2	40.9	2q31.3	CERKL	ceramide kinase-like protein isoform 2 [Homo sapiens].
52	NP_001195.2	1038	112	5.7	26.5	2q33-q34	BMPR2	bone morphogenetic protein receptor type-2 precursor [Homo
53	NP_056328.2	420	42	4.6	34.3	3p25.3	CRELD1	cysteine-rich with EGF-like domain protein 1 isoform 2 precursor
54	NP_001070883.1	420	42	4.6	34.3	3p25.3	CRELD1	cysteine-rich with EGF-like domain protein 1 isoform 2 precursor
55	NP_002871.1	648	73	9.5	34.4	3p25	RAF1	RAF proto-oncogene serine/threonine-protein kinase [Homo sapiens].
56	NP_001020018.1	592	65	5.3	31.1	3p22	TGFBR2	TGF-beta receptor type-2 isoform A precursor [Homo sapiens].
57	NP_003233.4	567	62	5.3	31.6	3p22	TGFBR2	TGF-beta receptor type-2 isoform B precursor [Homo sapiens].
58	NP_003140.1	402	45	8.5	21.4	3p22.3	STAC	SH3 and cysteine-rich domain-containing protein [Homo sapiens].
59	NP_001097.2	512	56	5.4	33.4	3p22	ACVR2B	activin receptor type-2B precursor [Homo sapiens].
60	NP_009113.3	340	39	9.1	24.7	3p21.3	RASSF1	ras association domain-containing protein 1 isoform A [Homo
61	NP_733832.1	344	39	9.1	24.4	3p21.3	RASSF1	ras association domain-containing protein 1 isoform D [Homo
62	NP_001007566.1	400	43	4.8	17.5	3q12.2	TFG	protein TFG isoform 1 [Homo sapiens].
63	NP_006061.2	400	43	4.8	17.5	3q12.2	TFG	protein TFG isoform 1 [Homo sapiens].
64	NP_001182407.1	400	43	4.8	17.5	3q12.2	TFG	protein TFG isoform 1 [Homo sapiens].

65	NP_001182408.1	396	43	4.9	17.7	3q12.2	TFG	protein TFG isoform 2 [Homo sapiens].
66	NP_444256.3	1794	197	6.1	27.0	3q21	MYLK	myosin light chain kinase, smooth muscle isoform 3B [Homo sapiens].
67	NP_444254.3	1845	203	5.8	26.7	3q21	MYLK	myosin light chain kinase, smooth muscle isoform 2 [Homo sapiens].
68	NP_444255.3	1863	205	6.1	27.1	3q21	MYLK	myosin light chain kinase, smooth muscle isoform 3A [Homo sapiens].
69	NP_444253.3	1914	211	5.8	26.8	3q21	MYLK	myosin light chain kinase, smooth muscle isoform 1 [Homo sapiens].
70	NP_060048.2	212	24	5.4	48.1	3q21.1	ROPN1	ropporin-1A [Homo sapiens].
71	NP_001012337.1	212	24	4.9	52.4	3q21.2	ROPN1B	ropporin-1B [Homo sapiens].
72	NP_006497.2	849	97	6.8	33.7	3q22-q23	RASA2	ras GTPase-activating protein 2 [Homo sapiens].
73	NP_003694.1	857	98	7.3	26.8	4p16.3	NOP14	nucleolar protein 14 [Homo sapiens].
74	NP_000779.1	260	31	5.0	26.2	4q13.3-q21	DCK	deoxycytidine kinase [Homo sapiens].
75	NP_079350.5	4012	441	5.2	35.6	4q21.21	FRAS1	extracellular matrix protein FRAS1 isoform 1 precursor [Homo
76	NP_001159605.1	1976	214	5.4	34.4	4q21.21	FRAS1	extracellular matrix protein FRAS1 isoform 2 precursor [Homo
77	NP_542416.1	2490	278	6.0	30.0	4q21.3	PTPN13	tyrosine-protein phosphatase non-receptor type 13 isoform 4 [Homo
78	NP_542414.1	2485	277	6.0	30.0	4q21.3	PTPN13	tyrosine-protein phosphatase non-receptor type 13 isoform 1 [Homo
79	NP_006255.1	2466	275	6.0	30.1	4q21.3	PTPN13	tyrosine-protein phosphatase non-receptor type 13 isoform 2 [Homo
80	NP_542415.1	2294	256	5.8	30.3	4q21.3	PTPN13	tyrosine-protein phosphatase non-receptor type 13 isoform 3 [Homo
81	NP_001228.1	432	49	6.1	35.0	4q27	CCNA2	cyclin-A2 [Homo sapiens].
82	NP_640336.1	576	64	9.3	36.3	4q27	ADAD1	adenosine deaminase domain-containing protein 1 isoform 1 [Homo
83	NP_001152757.1	565	63	9.1	36.3	4q27	ADAD1	adenosine deaminase domain-containing protein 1 isoform 2 [Homo
84	NP_001152767.1	558	62	9.3	36.9	4q27	ADAD1	adenosine deaminase domain-containing protein 1 isoform 3 [Homo
85	NP_776297.2	866	97	5.7	50.8	4q33	CLCN3	H(+)/Cl(-) exchange transporter 3 isoform e [Homo sapiens].
86	NP_001820.2	818	91	5.8	53.3	4q33	CLCN3	H(+)/Cl(-) exchange transporter 3 isoform b [Homo sapiens].
87	NP_001230301.1	791	88	6.0	52.1	4q33	CLCN3	H(+)/Cl(-) exchange transporter 3 isoform a [Homo sapiens].
88	NP_001230303.1	791	88	6.6	53.4	4q33	CLCN3	H(+)/Cl(-) exchange transporter 3 isoform c [Homo sapiens].
89	NP_114122.2	230	26	7.6	30.0	5p15.2	ROPN1L	ropporin-1-like protein [Homo sapiens].
90	NP_001188395.1	230	26	7.6	30.0	5p15.2	ROPN1L	ropporin-1-like protein [Homo sapiens].
91	NP_001093882.1	1337	155	7.9	24.3	5p13.3	DROSHA	ribonuclease 3 isoform 2 [Homo sapiens].
92	NP_037367.3	1374	159	7.7	24.5	5p13.3	DROSHA	ribonuclease 3 isoform 1 [Homo sapiens].
93	NP_001073948.2	1731	195	5.7	28.9	5q13.2	ARHGEF28	rho guanine nucleotide exchange factor 28 isoform 1 [Homo sapiens].
94	NP_001171164.1	1705	192	5.6	28.9	5q13.2	ARHGEF28	rho guanine nucleotide exchange factor 28 isoform 2 [Homo sapiens].
95	NP_001231293.1	1392	157	6.0	25.4	5q13.2	ARHGEF28	rho guanine nucleotide exchange factor 28 isoform 3 [Homo sapiens].
96	NP_004559.4	376	43	5.0	39.1	6p25	SERPINB6	serpin B6 [Homo sapiens].
97	NP_001182220.1	376	43	5.0	39.1	6p25	SERPINB6	serpin B6 [Homo sapiens].
98	NP_001243647.1	783	79	4.3	47.0	6q14.2	SNAP91	clathrin coat assembly protein AP180 isoform e [Homo sapiens].
99	NP_116173.2	272	31	9.7	21.0	6q22.33	RSPO3	R-spondin-3 precursor [Homo sapiens].
100	NP_004058.1	468	54	7.1	30.8	7p15.3	CHN2	beta-chimaerin isoform 2 [Homo sapiens].
101	NP_001035025.1	332	38	7.9	40.7	7p15.3	CHN2	beta-chimaerin isoform 1 [Homo sapiens].
102	NP_057700.3	588	67	4.8	33.0	7p14.1	NME8	thioredoxin domain-containing protein 3 [Homo sapiens].
103	NP_001231509.1	3859	438	8.1	45.9	7q21.2-q22	TRRAP	transformation/transcription domain-associated protein isoform 1
104	NP_003487.1	3830	434	8.1	46.0	7q21.2-q22	TRRAP	transformation/transcription domain-associated protein isoform 2
105	NP_055706.1	181	21	9.4	4.4	7q22.1	PDAP1	28 kDa heat- and acid-stable phosphoprotein [Homo sapiens].
106	NP_001009571.2	1255	143	5.7	33.9	7q31.3	CADPS2	calcium-dependent secretion activator 2 isoform b [Homo sapiens].
107	NP_060424.9	1296	148	5.8	34.5	7q31.3	CADPS2	calcium-dependent secretion activator 2 isoform a [Homo sapiens].
108	NP_001161412.1	1300	148	5.7	34.4	7q31.3	CADPS2	calcium-dependent secretion activator 2 isoform c [Homo sapiens].
109	NP_060708.1	422	43	6.7	36.1	7q34	AGK	acylglycerol kinase, mitochondrial precursor [Homo sapiens].
110	NP_004436.2	1021	111	6.2	39.9	7q33-q35	EPHB6	ephrin type-B receptor 6 precursor [Homo sapiens].
111	NP_060962.2	322	36	4.8	35.7	8p21.2	PBK	lymphokine-activated killer T-cell-originated protein kinase [Homo
112	NP_001157853.1	538	59	10.0	23.2	8q21.11	STAU2	double-stranded RNA-binding protein Staufen homolog 2 isoform b
113	NP_001157852.1	570	63	10.0	23.2	8q21.11	STAU2	double-stranded RNA-binding protein Staufen homolog 2 isoform a
114	NP_001157854.1	504	55	9.8	25.2	8q21.11	STAU2	double-stranded RNA-binding protein Staufen homolog 2 isoform c
115	NP_001157855.1	398	43	9.9	24.1	8q21.11	STAU2	double-stranded RNA-binding protein Staufen homolog 2 isoform d
116	NP_055208.2	479	53	10.1	23.6	8q21.11	STAU2	double-stranded RNA-binding protein Staufen homolog 2 isoform e
117	NP_001157856.1	479	53	10.1	23.6	8q21.11	STAU2	double-stranded RNA-binding protein Staufen homolog 2 isoform e
118	NP_001157857.1	473	52	10.1	22.8	8q21.11	STAU2	double-stranded RNA-binding protein Staufen homolog 2 isoform f
119	NP_443156.2	670	74	7.2	33.3	8q24.3	RHPN1	rhophilin-1 [Homo sapiens].
120	NP_006368.3	1591	181	5.6	28.0	9p13.3	UNC13B	protein unc-13 homolog B [Homo sapiens].
121	NP_004808.2	1190	134	7.0	20.2	9q13-q21	TJP2	tight junction protein ZO-2 isoform 1 [Homo sapiens].
122	NP_963923.1	1043	118	6.5	22.2	9q13-q21	TJP2	tight junction protein ZO-2 isoform 2 [Homo sapiens].
123	NP_001164101.1	993	112	7.1	24.7	9q13-q21	TJP2	tight junction protein ZO-2 isoform 6 [Homo sapiens].

124	NP_001163887.1	1221	137	8.1	21.3	9q13-q21	TJP2	tight junction protein ZO-2 isoform 3 [Homo sapiens].
125	NP_001163886.1	1157	130	7.3	21.0	9q13-q21	TJP2	tight junction protein ZO-2 isoform 4 [Homo sapiens].
126	NP_001163885.1	1020	115	6.3	22.5	9q13-q21	TJP2	tight junction protein ZO-2 isoform 5 [Homo sapiens].
127	NP_001177411.1	1860	194	5.4	23.9	9q21.3	PCSK5	proprotein convertase subtilisin/kexin type 5 isoform 1
128	NP_006191.2	913	89	6.1	26.7	9q21.3	PCSK5	proprotein convertase subtilisin/kexin type 5 isoform 2
129	NP_001177874.1	416	46	5.7	33.4	9q33-q34	TRAF1	TNF receptor-associated factor 1 isoform a [Homo sapiens].
130	NP_005649.1	416	46	5.7	33.4	9q33-q34	TRAF1	TNF receptor-associated factor 1 isoform a [Homo sapiens].
131	NP_001177876.1	294	33	6.2	36.1	9q33-q34	TRAF1	TNF receptor-associated factor 1 isoform b [Homo sapiens].
132	NP_060857.2	672	74	8.9	32.7	9q33.3	STRBP	spermatid perinuclear RNA-binding protein isoform 1 [Homo sapiens].
133	NP_001164608.1	658	72	8.8	33.1	9q33.3	STRBP	spermatid perinuclear RNA-binding protein isoform 2 [Homo sapiens].
134	NP_003362.2	839	97	6.5	29.9	9q34.1	VAV2	guanine nucleotide exchange factor VAV2 isoform 2 [Homo sapiens].
135	NP_001127870.1	878	101	6.7	30.6	9q34.1	VAV2	guanine nucleotide exchange factor VAV2 isoform 1 [Homo sapiens].
136	NP_066961.2	501	56	7.3	38.1	9q34	TRAF2	TNF receptor-associated factor 2 [Homo sapiens].
137	NP_061172.1	739	81	10.6	33.4	10p15.3	ADARB2	double-stranded RNA-specific editase B2 [Homo sapiens].
138	NP_079114.3	743	83	6.7	45.1	10p12.1	THNSL1	threonine synthase-like 1 [Homo sapiens].
139	NP_001018081.3	1309	144	6.3	37.1	10q11.22	FRMPD2	FERM and PDZ domain-containing protein 2 isoform 3 [Homo sapiens].
140	NP_660350.2	609	69	7.7	30.9	10q21.2	RTKN2	rhotekin-2 [Homo sapiens].
141	NP_002649.1	431	18	8.5	17.2	10q24	PLAU	urokinase-type plasminogen activator isoform 1 preproprotein [Homo
142	NP_002649.1	431	3	10.2	0.0	10q24	PLAU	urokinase-type plasminogen activator isoform 1 preproprotein [Homo
143	NP_002649.1	431	28	7.7	33.6	10q24	PLAU	urokinase-type plasminogen activator isoform 1 preproprotein [Homo
144	NP_001138503.1	414	47	8.3	27.8	10q24	PLAU	urokinase-type plasminogen activator isoform 2 [Homo sapiens].
145	NP_004738.3	1919	214	7.1	23.8	10q23	DLG5	disks large homolog 5 [Homo sapiens].
146	NP_001017423.1	793	87	6.7	47.7	10q24.3	ALDH18A1	delta-1-pyrroline-5-carboxylate synthase isoform 2 [Homo sapiens].
147	NP_776152.1	1154	129	5.7	32.1	10q26.12	PDZD8	PDZ domain-containing protein 8 [Homo sapiens].
148	NP_006532.2	335	37	5.2	36.1	10q26	GLRX3	glutaredoxin-3 [Homo sapiens].
149	NP_001186797.1	335	37	5.2	36.1	10q26	GLRX3	glutaredoxin-3 [Homo sapiens].
150	NP_055159.2	437	49	11.1	35.9	11p13	FJX1	four-jointed box protein 1 precursor [Homo sapiens].
151	NP_001092140.1	609	69	7.6	37.6	11q13	RASGRP2	RAS guanyl-releasing protein 2 [Homo sapiens].
152	NP_001092141.1	609	69	7.6	37.6	11q13	RASGRP2	RAS guanyl-releasing protein 2 [Homo sapiens].
153	NP_722541.1	609	69	7.6	37.6	11q13	RASGRP2	RAS guanyl-releasing protein 2 [Homo sapiens].
154	NP_689645.2	874	99	7.9	28.0	11q22.1	ARHGAP42	rho GTPase-activating protein 42 [Homo sapiens].
155	NP_001231.2	726	81	9.0	24.2	12q13.11	CCNT1	cyclin-T1 [Homo sapiens].
156	NP_037409.2	632	71	9.1	30.5	12q13.12	RACGAP1	rac GTPase-activating protein 1 [Homo sapiens].
157	NP_001119575.1	632	71	9.1	30.5	12q13.12	RACGAP1	rac GTPase-activating protein 1 [Homo sapiens].
158	NP_001119576.1	632	71	9.1	30.5	12q13.12	RACGAP1	rac GTPase-activating protein 1 [Homo sapiens].
159	NP_056134.2	1419	154	8.2	29.6	12q13.13	TENC1	tensin-like C1 domain-containing phosphatase isoform 1 [Homo
160	NP_736610.2	1409	153	8.2	29.2	12q13.13	TENC1	tensin-like C1 domain-containing phosphatase isoform 2 [Homo
161	NP_065434.1	573	63	5.5	33.0	12q13	AMHR2	anti-Muellerian hormone type-2 receptor isoform 1 precursor [Homo
162	NP_599150.1	366	39	6.1	38.3	12q12-q13	TARBP2	RISC-loading complex subunit TARBP2 isoform a [Homo sapiens].
163	NP_599151.2	345	37	7.1	37.1	12q12-q13	TARBP2	RISC-loading complex subunit TARBP2 isoform b [Homo sapiens].
164	NP_004169.3	345	37	7.1	37.1	12q12-q13	TARBP2	RISC-loading complex subunit TARBP2 isoform b [Homo sapiens].
165	NP_659501.1	364	42	6.5	16.5	12q13.3	STAC3	SH3 and cysteine-rich domain-containing protein 3 [Homo sapiens].
166	NP_002920.1	563	64	5.5	34.8	13q34	GRK1	rhodopsin kinase [Homo sapiens].
167	NP_031394.2	834	96	6.8	33.6	13q34	RASA3	ras GTPase-activating protein 3 [Homo sapiens].
168	NP_663777.1	568	64	7.8	29.6	14q32.32	TRAF3	TNF receptor-associated factor 3 isoform 1 [Homo sapiens].
169	NP_003291.2	568	64	7.8	29.6	14q32.32	TRAF3	TNF receptor-associated factor 3 isoform 1 [Homo sapiens].
170	NP_783297.2	1668	187	6.3	16.5	15q13	TJP1	tight junction protein ZO-1 isoform b [Homo sapiens].
171	NP_003248.3	1748	195	6.2	16.1	15q13	TJP1	tight junction protein ZO-1 isoform a [Homo sapiens].
172	NP_001122074.1	762	87	7.6	34.1	15q14	RASGRP1	RAS guanyl-releasing protein 1 isoform b [Homo sapiens].
173	NP_005730.2	797	90	7.8	34.0	15q14	RASGRP1	RAS guanyl-releasing protein 1 isoform a [Homo sapiens].
174	NP_001074003.1	2214	251	5.6	25.7	15q21.3	UNC13C	protein unc-13 homolog C [Homo sapiens].
175	NP_008832.2	2548	293	9.1	28.3	15q22-q23	MYO9A	unconventional myosin-IXa [Homo sapiens].
176	NP_002560.1	794	74	5.1	33.8	15q26.1	FURIN	furin preproprotein [Homo sapiens].
177	NP_000866.1	1367	81	6.3	34.9	15q26.3	IGF1R	insulin-like growth factor 1 receptor precursor [Homo sapiens].
178	NP_000866.1	1367	71	5.0	32.2	15q26.3	IGF1R	insulin-like growth factor 1 receptor precursor [Homo sapiens].
179	NP_612192.1	956	89	6.5	30.6	15q26.3	PCSK6	proprotein convertase subtilisin/kexin type 6 isoform b
180	NP_002561.1	969	90	6.5	30.9	15q26.3	PCSK6	proprotein convertase subtilisin/kexin type 6 isoform a
181	NP_612194.1	962	89	6.7	30.6	15q26.3	PCSK6	proprotein convertase subtilisin/kexin type 6 isoform h
182	NP_612193.1	975	91	6.7	30.9	15q26.3	PCSK6	proprotein convertase subtilisin/kexin type 6 isoform g

183	NP_150296.3	974	107	6.8	38.2	16p12.2	ERN2	serine/threonine-protein kinase/endoribonuclease IRE2 [Homo
184	NP_005872.2	412	43	8.1	33.5	16p11.2	BCKDK	[3-methyl-2-oxobutanoate dehydrogenase [lipoamide]] kinase,
185	NP_001116429.1	365	41	9.1	34.5	16p11.2	BCKDK	[3-methyl-2-oxobutanoate dehydrogenase [lipoamide]] kinase,
186	NP_001180453.1	1233	133	6.0	32.8	16q22.1	FAM65A	protein FAM65A isoform 4 [Homo sapiens].
187	NP_001180451.1	1223	132	5.8	33.1	16q22.1	FAM65A	protein FAM65A isoform 2 [Homo sapiens].
188	NP_078795.2	1219	132	5.8	32.9	16q22.1	FAM65A	protein FAM65A isoform 1 [Homo sapiens].
189	NP_060273.1	493	55	6.7	41.4	16q22.1	DUS2L	tRNA-dihydrouridine(20) synthase [NAD(P)+]-like [Homo sapiens].
190	NP_631913.3	665	71	8.6	47.5	16q24.1	ADAD2	adenosine deaminase domain-containing protein 2 isoform 1 [Homo
191	NP_001138872.1	583	62	8.9	47.2	16q24.1	ADAD2	adenosine deaminase domain-containing protein 2 isoform 2 [Homo
192	NP_115827.1	714	80	7.4	28.9	16q24	SPIRE2	protein spire homolog 2 [Homo sapiens].
193	NP_997397.1	512	59	6.1	33.4	16q24.3	DEF8	differentially expressed in FDCP 8 homolog isoform 1 [Homo
194	NP_001229747.1	451	52	5.8	33.0	16q24.3	DEF8	differentially expressed in FDCP 8 homolog isoform 5 [Homo
195	NP_001229749.1	451	52	5.8	33.0	16q24.3	DEF8	differentially expressed in FDCP 8 homolog isoform 5 [Homo
196	NP_001229748.1	434	50	6.0	34.1	16q24.3	DEF8	differentially expressed in FDCP 8 homolog isoform 6 [Homo
197	NP_001229745.1	441	51	5.9	32.0	16q24.3	DEF8	differentially expressed in FDCP 8 homolog isoform 3 [Homo
198	NP_001229746.1	391	45	6.3	37.1	16q24.3	DEF8	differentially expressed in FDCP 8 homolog isoform 4 [Homo
199	NP_001028723.1	457	50	4.4	26.9	17p11.2	TOM1L2	TOM1-like protein 2 isoform 1 [Homo sapiens].
200	NP_945344.1	411	45	7.0	25.8	17q12	STAC2	SH3 and cysteine-rich domain-containing protein 2 [Homo sapiens].
201	NP_001035997.2	593	65	7.4	45.9	17q12-q21	COASY	bifunctional coenzyme A synthase isoform c [Homo sapiens].
202	NP_079509.5	564	62	6.5	46.3	17q12-q21	COASY	bifunctional coenzyme A synthase isoform a precursor [Homo
203	NP_001035994.1	564	62	6.5	46.3	17q12-q21	COASY	bifunctional coenzyme A synthase isoform a precursor [Homo
204	NP_114068.1	966	107	4.9	31.0	17q21.31	NBR1	next to BRCA1 gene 1 protein [Homo sapiens].
205	NP_005890.2	966	107	4.9	31.0	17q21.31	NBR1	next to BRCA1 gene 1 protein [Homo sapiens].
206	NP_114064.1	966	107	4.9	31.0	17q21.31	NBR1	next to BRCA1 gene 1 protein [Homo sapiens].
207	NP_001018146.1	267	30	9.3	39.7	17q21.3	NME1-NME2	NME1-NME2 protein [Homo sapiens].
208	NP_001424.3	977	110	6.0	33.7	17q24.2	ERN1	serine/threonine-protein kinase/endoribonuclease IRE1 precursor
209	NP_060035.2	541	58	7.5	41.1	17q24.2	FAM20A	protein FAM20A isoform a precursor [Homo sapiens].
210	NP_001230675.1	403	46	6.2	45.9	17q24.2	FAM20A	protein FAM20A isoform b [Homo sapiens].
211	NP_001166175.1	592	63	5.5	40.5	17q25.1	GGA3	ADP-ribosylation factor-binding protein GGA3 isoform 2 [Homo
212	NP_001166174.1	651	70	5.8	38.1	17q25.1	GGA3	ADP-ribosylation factor-binding protein GGA3 isoform 1 [Homo
213	NP_059345.1	552	61	9.2	21.0	17q25	BAIAP2	brain-specific angiogenesis inhibitor 1-associated protein 2
214	NP_001138360.1	534	59	9.4	21.7	17q25	BAIAP2	brain-specific angiogenesis inhibitor 1-associated protein 2
215	NP_006331.1	520	57	9.1	22.7	17q25	BAIAP2	brain-specific angiogenesis inhibitor 1-associated protein 2
216	NP_059344.1	521	57	9.2	23.0	17q25	BAIAP2	brain-specific angiogenesis inhibitor 1-associated protein 2
217	NP_064533.3	742	84	8.6	23.2	18p11.21	SPIRE1	protein spire homolog 1 isoform b [Homo sapiens].
218	NP_001122098.1	756	86	8.6	23.8	18p11.21	SPIRE1	protein spire homolog 1 isoform a [Homo sapiens].
219	NP_001122099.1	622	72	8.8	21.7	18p11.21	SPIRE1	protein spire homolog 1 isoform c [Homo sapiens].
220	NP_005015.1	397	45	5.8	31.7	18q21.3	SERPINB10	serpin B10 [Homo sapiens].
221	NP_277048.2	290	31	9.3	41.0	19p13.3	TPGS1	tubulin polyglutamylase complex subunit 1 [Homo sapiens].
222	NP_036424.2	1136	1	3.7	11.1	19p13.3	HMHA1	minor histocompatibility protein HA-1 isoform 1 [Homo sapiens].
223	NP_060043.2	755	70	6.4	37.7	19p13.3	PCSK4	proprotein convertase subtilisin/kexin type 4 precursor [Homo
224	NP_001254490.1	928	102	6.3	24.0	19p13.3	TJP3	tight junction protein ZO-3 isoform 2 [Homo sapiens].
225	NP_001254489.1	919	101	6.3	23.8	19p13.3	TJP3	tight junction protein ZO-3 isoform 1 [Homo sapiens].
226	NP_004231.1	545	63	5.1	14.7	19p13.3	TRIP10	cdc42-interacting protein 4 [Homo sapiens].
227	NP_005419.2	845	98	6.2	25.8	19p13.2	VAV1	proto-oncogene vav isoform 1 [Homo sapiens].
228	NP_001245135.1	823	96	6.0	26.4	19p13.2	VAV1	proto-oncogene vav isoform 2 [Homo sapiens].
229	NP_001245136.1	813	94	6.3	27.1	19p13.2	VAV1	proto-oncogene vav isoform 3 [Homo sapiens].
230	NP_001073285.1	1370	83	5.9	33.2	19p13.3-p1	INSR	insulin receptor isoform Short preproprotein [Homo sapiens].
231	NP_001073285.1	1370	70	5.3	33.1	19p13.3-p1	INSR	insulin receptor isoform Short preproprotein [Homo sapiens].
232	NP_008996.1	378	44	5.0	14.3	19p13.2	CDC37	hsp90 co-chaperone Cdc37 [Homo sapiens].
233	NP_060090.2	898	96	9.1	23.2	19p13.2	ILF3	interleukin enhancer-binding factor 3 isoform d [Homo sapiens].
234	NP_036350.2	894	95	9.0	23.4	19p13.2	ILF3	interleukin enhancer-binding factor 3 isoform a [Homo sapiens].
235	NP_703194.1	690	75	8.3	30.6	19p13.2	ILF3	interleukin enhancer-binding factor 3 isoform c [Homo sapiens].
236	NP_001131145.1	706	77	7.8	29.6	19p13.2	ILF3	interleukin enhancer-binding factor 3 isoform e [Homo sapiens].
237	NP_004507.2	702	76	7.6	29.9	19p13.2	ILF3	interleukin enhancer-binding factor 3 isoform b [Homo sapiens].
238	NP_004136.2	2157	243	8.9	29.1	19p13.1	MYO9B	unconventional myosin-IXb isoform 1 [Homo sapiens].
239	NP_001123537.1	2022	229	8.6	29.1	19p13.1	MYO9B	unconventional myosin-IXb isoform 2 [Homo sapiens].
240	NP_001073890.2	1703	193	5.1	28.3	19p13.11	UNC13A	protein unc-13 homolog A [Homo sapiens].
241	NP_057657.2	970	107	5.4	24.6	19p13.11	GMIP	GEM-interacting protein [Homo sapiens].
